# Reconstructor: a COBRApy compatible tool for automated genome-scale metabolic network reconstruction with parsimonious flux-based gap-filling

**DOI:** 10.1093/bioinformatics/btad367

**Published:** 2023-06-05

**Authors:** Matthew L Jenior, Emma M Glass, Jason A Papin

**Affiliations:** Department of Biomedical Engineering, University of Virginia, Charlottesville, Virginia, United States; Department of Biomedical Engineering, University of Virginia, Charlottesville, Virginia, United States; Department of Biomedical Engineering, University of Virginia, Charlottesville, Virginia, United States; Department of Medicine, Division of Infectious Diseases & International Health, University of Virginia, Charlottesville, Virginia, United States; Department of Biochemistry & Molecular Genetics, University of Virginia, Charlottesville, Virginia, United States

## Abstract

**Motivation:**

Genome-scale metabolic network reconstructions (GENREs) are valuable for understanding cellular metabolism *in silico*. Several tools exist for automatic GENRE generation. However, these tools frequently (i) do not readily integrate with some of the widely-used suites of packaged methods available for network analysis, (ii) lack effective network curation tools, (iii) are not sufficiently user-friendly, and (iv) often produce low-quality draft reconstructions.

**Results:**

Here, we present Reconstructor, a user-friendly, COBRApy-compatible tool that produces high-quality draft reconstructions with reaction and metabolite naming conventions that are consistent with the ModelSEED biochemistry database and includes a gap-filling technique based on the principles of parsimony. Reconstructor can generate SBML GENREs from three input types: annotated protein .fasta sequences (Type 1 input), a BLASTp output (Type 2), or an existing SBML GENRE that can be further gap-filled (Type 3). While Reconstructor can be used to create GENREs of any species, we demonstrate the utility of Reconstructor with bacterial reconstructions. We demonstrate how Reconstructor readily generates high-quality GENRES that capture strain, species, and higher taxonomic differences in functional metabolism of bacteria and are useful for further biological discovery.

**Availability and implementation:**

The Reconstructor Python package is freely available for download. Complete installation and usage instructions and benchmarking data are available at http://github.com/emmamglass/reconstructor.

## 1 Introduction

Genome-scale metabolic network reconstructions (GENREs) are valuable tools for understanding the link between the genotype and phenotype of an organism. GENREs can enable greater understanding of the effects of genetic and environmental perturbation on cellular function and can help identify novel drug targets, among many other applications (Haggart *et al.* 2011, Kim *et al.* 2012, Gu *et al.* 2019).

The generation of GENREs can be an incredibly laborious and complex process, requiring the integration of data from multiple sources (Thiele and Palsson 2010). The creation of a GENRE begins with the annotated genome sequence to predict reactions to include in the draft GENRE, and then further model curation steps are performed to gap-fill missing reactions. While GENREs can be generated and curated manually, methods for the automated creation of GENREs have emerged (Mendoza *et al.* 2019).

**Figure 1.**
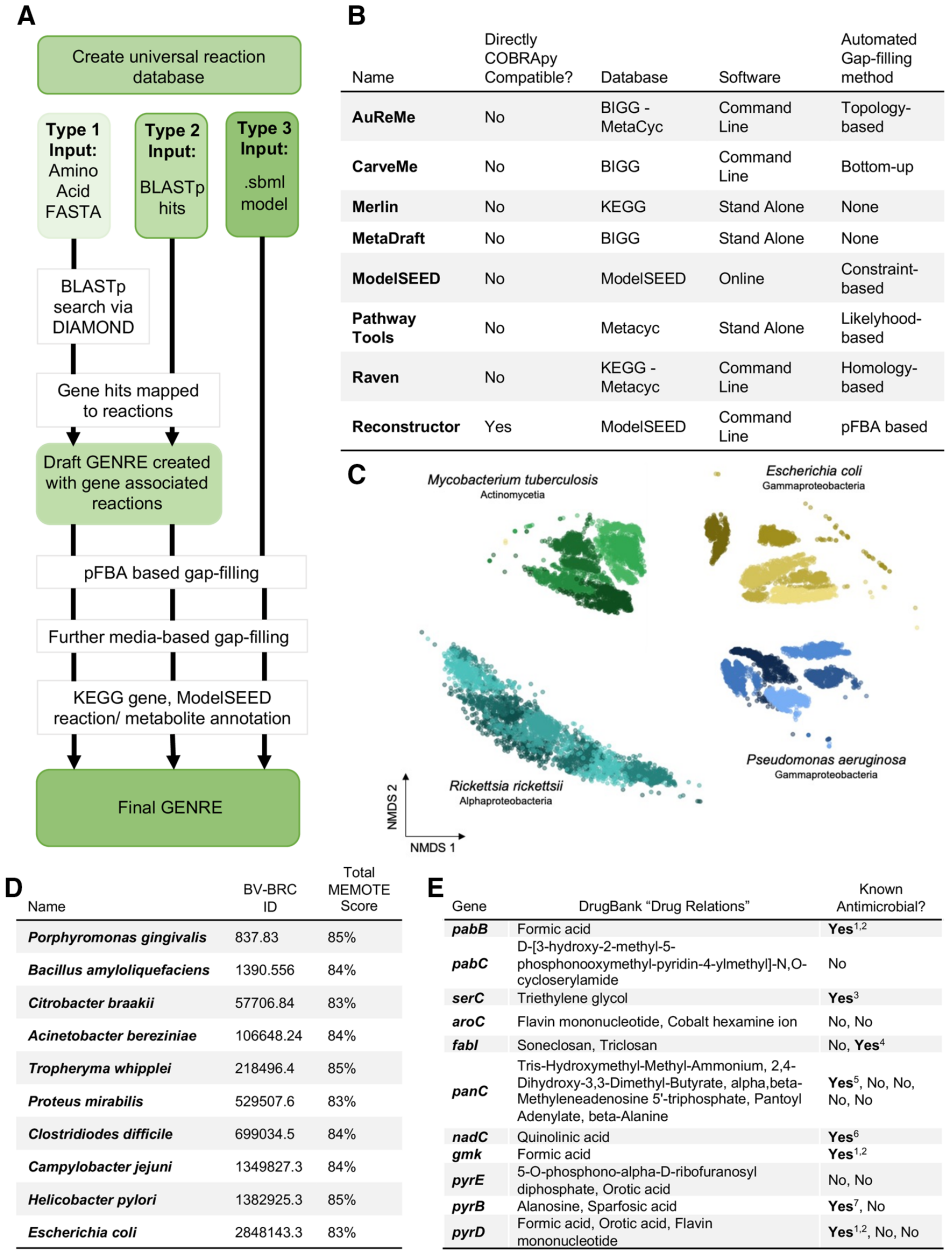
Reconstructor overview. (A) Flowchart detailing the functionality of the Reconstructor tool. (B) Comparison of other widely used GENRE construction tools including Reconstructor, adapted from Mendoza *et al.* (2019). (C) Flux sampling was performed on 20 bacterial reconstructions generated with Reconstructor (five strains for each of four species). Each dot represents a sampled flux distribution. Five hundred sampled flux distributions were captured for each reconstruction. Samples were dimensionally reduced using non-metric multidimensional scaling and plotted on a 2D plane for visualization. (D) GENREs were created via Reconstructor for each of the 10 bacterial species listed, genome sequences were downloaded from the BV-BRC (Davis *et al.* 2020), BV-BRC IDs for each species are listed. (E) Essential genes for a generated *Pseudomonas aeruginosa* (strain NCGM2.S1) reconstruction, existing drugs that target those essential genes according to DrugBank, and whether the identified drugs are known antimicrobials. ^1^Ricke *et al.* (2020), ^2^Alzahrani *et al.* (2022), ^3^Chrószcz *et al.* (2022), ^4^Kondratenko *et al.* (2020), ^5^Narui *et al.* (2009), ^6^Murthy *et al.* (1945), and ^7^Yazdankhah *et al.* (2006).

Several platforms exist for automated GENRE creation, including ModelSEED (Seaver *et al.* 2021), CarveMe (Machado *et al.* 2018), and among others (Dias *et al.* 2015, Aite *et al.* 2018; Olivier 2018, Wang *et al.* 2018, Karp et al. 2021) (Fig. 1B). However, the reconstructions generated by these tools typically require additional compatibility modules for integration with COBRApy (Ebrahim *et al.* 2013), and subsequent manual or automated curation (King *et al.* 2015, Moretti *et al.* 2016, Mundy *et al.* 2017, Camborda *et al.* 2022, Saadat *et al.* 2022, Schneider *et al.* 2022; see Supplementary).

Here, we introduce Reconstructor, an automated GENRE creation tool that generates COBRApy-compatible reconstructions in the ModelSEED namespace. Additionally, we include a two-step gap-filling technique based on parsimonious flux balance analysis (pFBA) (Lewis *et al.* 2010), a more biologically tractable gap-filling technique than other techniques based exclusively on gene–protein-reaction mapping. pFBA is motivated by the possible notion that high metabolic flux has high enzyme turn-over and that the synthesis of enzymes is energetically costly. Consequently, the model will minimize overall flux (and thus costly high enzyme turn-over), but still maximize for a given objective function (Lewis *et al.* 2010). Using a pFBA approach to gap-filling ensures that we account for all reactions with genetic evidence while generating a reconstruction that is consistent with these principles of parsimony. We also demonstrate how metabolic network models derived from Reconstructor can be used to generate experimentally testable hypotheses.

## 2 Results

### 2.1 Universal reaction database construction

A universal database of metabolic reactions was adapted from the ModelSEED database, removing all reactions that are unbalanced (see Supplementary). From this modified ModelSEED database, we generated reaction and metabolite dictionaries, missing exchange reactions were identified and corrected, and the biomass function was updated. The result was a universal database that contains a reaction collection from which the genome-informed model can select reactions for inclusion and gap-filling. The user can also curate their own universal database to use with Reconstructor by altering the ModelSEED reaction and metabolite dictionaries. The ability to readily curate this existing universal database or to make use of any other user-provided universal database in the same name-space is a key feature of Reconstructor.

### 2.2 Input data formats and draft GENRE scaffold extraction

Reconstructor automates the build of a GENRE from three different types of user-defined inputs. Type 1 requires inputs of an annotated genome sequence in the form of an amino acid FASTA file. It is important to note that the user must annotate the genome beforehand, as genome DNA files are currently not supported as inputs in Reconstructor. Type 2 requires an input of BLASTp hits, bypassing the BLASTp search step. Type 3 requires an existing GENRE in SBML format in the same namespace and with the same construction (e.g. definition of intracellular/extracellular compartments) as Reconstructor network reconstructions, and further pFBA gap-filling is performed (as described in Supplementary). Additionally, for input Types 1 and 2, the user can define their own media conditions for a given GENRE by providing metabolite names in their defined media condition (further discussed in Supplementary).

The GENRE creation process is described below from the starting point of a Type 1 input. The amino acid FASTA file is aligned to the KEGG database by performing a BLASTp search with the DIAMOND sequence aligner tool (Buchfink *et al.* 2015). Then, the KEGG gene hits are processed and translated into ModelSEED reactions. These reactions and associated gene names are used to create a draft GENRE based solely on gene-associated reactions. Additionally, reactions are added to the draft GENRE based on media conditions.

### 2.3 Parsimonious flux balance analysis-based approach to gap-filling draft GENREs

Several gap-filling methods exist (Pan and Reed 2018), many of which use parsimony as a guiding principle in which a minimum number of reactions are added to satisfy criteria like growth in defined media (Prigent *et al.* 2017, King *et al.* 2018, Zimmermann *et al.* 2021). In Reconstructor, we introduce a two-step gap-filling process based on (i) parsimonious flux principles and (ii) user-defined media conditions. Our gap-filling technique works by minimizing the flux through an optimal reaction set (all gene-associated reactions and a set of non-gene associated reactions that minimizes flux), rather than minimizing the number of reactions added to the network (see Supplementary). After the optimal reaction set is chosen, reactions are added to the GENRE.

### 2.4 Component annotation and final GENRE output

The final gap-filled GENRE is then annotated with KEGG (Kanehisa *et al.* 2016) gene IDs, ModelSEED metabolites, and reaction names. Finally, basic model statistics are reported including the number of genes, reactions, and metabolites in the draft and final GENREs, how many reactions were the result of gap-filling, and the final biomass objective flux so the user can ensure the gap-filling process was successful. Finally, the model is saved in SBML format, the current community standard (Hucka *et al.* 2003).

### 2.5 COBRApy compatibility

Current widely-used GENRE creation tools, ModelSEED and CarveMe, both require additional modules to be used in conjunction with COBRApy (Moretti *et al.* 2016, Mundy *et al.* 2017). Reconstructor GENREs are directly compatible with COBRApy; they can be generated via command line and easily imported into Python, or generated directly in a Python script using the reconstruct() function to easily and immediately take advantage of the powerful COBRApy analysis toolbox. Reconstructor’s direct COBRApy compatibility allows users to streamline GENRE analysis pipelines, potentially accelerating GENRE-based discovery and hypothesis generation.

### 2.6 Reconstructor utility

We demonstrate the utility of Reconstructor GENREs by addressing three key aspects: (i) quality of reconstructions for a range of bacteria with different levels of literature investigation, (ii) ability of GENREs to capture strain-level differences, and (iii) ability to quickly generate testable biological hypotheses.

While Reconstructor could be used for any annotated amino acid .fasta file, we demonstrate here the utility of Reconstructor with bacterial reconstructions. We generated a total of 10 GENREs representing unique bacterial strains for analysis and benchmarking through the metabolic model testing suite (MEMOTE) (Lieven *et al.* 2020). We selected a diverse set of bacterial species to ensure we can generate high-quality reconstructions for both well studied/annotated species like *Clostridium difficile* and lesser-known species like *Tropheryma whipplei.* MEMOTE scores and SBML reconstructions for each of the 10 species (Fig. 1C) are available at http://github.com/emmamglass/reconstructor. The overall MEMOTE scores for the reconstructions ranged from 83% to 85% (Fig. 1D). MEMOTE score comparisons between similar ModelSEED and CarveMe reconstructions are discussed in Supplementary.

While the benchmarking quality of Reconstructor GENREs is high, we wanted to ensure that Reconstructor creates GENREs that are capable of capturing strain-, species-, and class-level variation in metabolic functionality. To address this question, we further generated reconstructions for five distinct strains of each of four bacterial species, *Pseudomonas aeruginosa, Mycobacterium tuberculosis, Escherichia coli*, and *Rickettsia rickettsii*, for a total of 20 reconstructions. Through flux balance analysis and visualization with nonmetric multidimensional scaling, we show that the Reconstructor network reconstructions are able to capture functional metabolic differences in strain, species, and class, as evidenced by distinct clustering of flux samples (Fig. 1C) (see Supplementary).

Since we determined that Reconstructor GENREs are able to capture differences in metabolic functionality with significant detail, we wanted to demonstrate the utility of Reconstructor GENREs for generating testable biological hypotheses rapidly through integration with COBRApy. We generated a metabolic network reconstruction of a *Pseudomonas aeruginosa* strain, NCGM2.S1, that has not been previously created. Because of Reconstructor’s direct COBRApy compatibility, we were able to apply COBRApy tools to run a gene essentiality analysis. We then mapped these essential genes to targets of existing drugs in DrugBank (Wishart *et al.* 2018). We determined that 7 identified drugs are known inhibitors of microbial growth, while 13 other drugs had not been tested previously (Fig. 1E). These untested drugs represent new hypotheses that can readily be tested experimentally (see Supplementary).

## 3 Conclusion

Reconstructor automatically creates and curates COBRApy-compatible, genome-scale metabolic network reconstructions in the ModelSEED namespace and uses a pFBA based gap-filling technique (Fig. 1A) that is more consistent with parsimony principles in metabolic modeling than conventional gap-filling techniques (Jenior *et al.* 2020). Direct COBRApy compatibility enables the user to import GENREs directly into Python for further downstream analysis via the robust COBRApy toolbox. Reconstructor generates high-quality GENREs as evidenced through MEMOTE benchmarking, captures class-, species-, and even strain-level differences in functional metabolism and can be used for rapid experimental hypothesis generation.

## Supplementary Material

btad367_Supplementary_Data

## Data Availability

The data presented in this article are available at http://github.com/emmamglass/reconstructor and in the online supplementary material.
