# Supplementary material for: Reconstructor: a COBRApy compatible tool for automated genome-scale metabolic network reconstruction with parsimonious flux-based gap-filling
Source: Bioinformatics. 2023 Jun 5;39(6):btad367. doi: 10.1093/bioinformatics/btad367 (PMC10275916; doi:10.1093/bioinformatics/btad367)
Supplement: btad367_Supplementary_Data [file btad367_supplementary_data.docx]

**Existing automated GENRE creation tools and COBRApy compatibility/ curation modules**

Several platforms exist for automated GENRE creation, including ModelSEED (Seaver *et al.*, 2021) and CarveMe (Machado *et al.*, 2018), among others (Chevallier *et al.*, 2018; Dias *et al.*, 2015; Olivier, 2018; Karp *et al.*; Wang *et al.*, 2018). However, additional compatibility modules are necessary to use CarveMe- and ModelSEED-generated GENREs with the COBRApy analysis toolbox (Moretti *et al.*, 2016; Mundy *et al.*, 2017a). Integration of GENREs with the COBRApy toolbox (Ebrahim *et al.*, 2013) is critical for the analysis of GENREs and *in-silico* hypothesis testing with this rapidly developing suite of modeling tools. The COBRApy toolbox can be used for a variety of analyses including to understand functional metabolic states through flux balance analysis (cobra.sampling.sample()) and determining genes that are essential for growth (cobra.flux_analysis.variability.find_essential_genes()).

Additionally, there are many existing Python packages related to GENRE construction, curation, and optimization. Some examples include: Mackinac; Python package that generates a ModelSEED GENRE in Python by interfacing with the ModelSEED API (Mundy *et al.*, 2017b), CobraMod; a GENRE curation/extension package that interfaces with Escher for pathway visualization (Camborda *et al.*, 2022; King *et al.*, 2015), moped; which can generate automated draft GENREs using the MetaCyc database in Python but does not present a novel gap-filling method (Saadat *et al.*, 2022), and StrainDesign; a Python package that can be used for optimal strain design (Schneider *et al.*, 2022). However, there is not an existing tool that can generate a curated GENRE that has a two-step gap-filling technique based on parsimonius flux principles and defined media conditions, that can be used as either a command-line program or used in a Python script to directly interface with COBRApy.

**pFBA based gap-filler**

The goal of the pFBA gap-filler is to minimize total flux through the reaction network. The gap-filling process is broken into two distinct steps: 1) initial gap-filling based on gene-associated reactions, and 2) secondary gap-filling based on defined media conditions.

*Step 1 gap-filling: draft GENRE creation*

First, the gene-associated reactions are removed from the universal reaction database. This step ensures that these reactions are not included twice in the draft Reconstructor GENRE. Then, we define the constraints (upper and lower bounds) for the pFBA gap-filler based on a fraction of total flux through the biomass function. The default value for the lower and upper bounds of the total flux fraction are 0.01 and 0.5, respectively. These values can be changed by the user in the –max_frac and –min_frac arguments. After setting the constraints, we can begin to construct a new objective function for the pFBA based gap-filler. Ultimately, this objective function will be the sum of fluxes and corresponding linear coefficients for gene associated reactions and all other reactions in the universal reaction database. This objective function will be minimized. The first step in constructing the pFBA-based objective function is a linear coefficient assignment to all reactions. We first assign all gene-associated reaction fluxes a linear coefficient of zero to ensure that gene-associated reactions are selected by the gap-filler and added to the draft GENRE, since the objective function is being minimized. Next, we assign all non-gene associated reactions (those in the universal database) are assigned a linear coefficient of 1. All reactions and corresponding linear coefficients are added to the pFBA objective function and their sum is minimized. The solution to this optimization problem returns a set of reactions that includes all gene-associated reactions and an optimal set of reactions from the universal reaction database that minimizes the total flux through the reaction network. Finally, the solution set is further pruned to remove all reactions that have an absolute value of flux less than 1x10^-6^. The resulting reaction set is added to the Reconstructor GENRE.

*Step 2 gap-filling: final GENRE creation*

A second round of gap-filling is performed based on defined media conditions. First, all exchange reactions in a given media condition are turned ‘on’ by changing their flux bounds from zero to -1000 (lower bound) and  -0.1 (upper bound), and are added to the draft GENRE created in step 1 gap-filling. For this second iteration of pFBA gap-filling based on media conditions, all reactions that exist in the draft GENRE from step 1 are removed from the universal database. Next, we define further constraints on the pFBA-based objective function used for gap-filling. We define the lower bound for the pFBA-based objective as a fraction (0.5, the max_frac default input) of the total biomass flux of the draft GENRE. The new upper bound constraint is defined as the total biomass flux through the draft GENRE. Next, we assign linear coefficients. All reactions that were included in the draft GENRE are assigned a linear coefficient of zero, which ensures they will be included in the final Reconstructor GENRE. All other reactions in the universal database are assigned linear coefficients of 1. Then, as in step 1 gap-filling, all reactions and their corresponding linear coefficients are added to the pFBA objective function, and the sum of the products of reaction fluxes and linear coefficients is minimized. The solution to this optimization problem returns a reaction set that includes all gene-associated reactions, all exchange reactions defined by the media conditions, all reactions that were included in the draft GENRE from gap-filling in step 1, and a new set of reactions from the universal reaction database that minimizes total flux through the reaction network. Finally, the solution set is further pruned to remove reactions that have an absolute value of flux less than 1x10^-6^. The resulting reaction set is added to the final Reconstructor GENRE.

**MEMOTE score comparison for 10 representative reconstructions**

We compared our 10 representative reconstructions to comparable reconstructions created using ModelSEED and CarveMe. We selected a diverse subset of bacterial species to use for Reconstructor benchmarking to ensure we could generate high quality reconstructions for both well studied/annotated species like *C. difficile* and lesser-known species like *T. whipplei.*

ModelSEED representative models were created using the same genome sequence as Reconstructor models. CarveMe models are from the same species (or genus if no equivalent species) as the representative Reconstructor models, but different strains. These GENRES were taken from the CarveMe repository (<https://github.com/cdanielmachado/carveme>) and used for model quality comparison.

Figure S1 A shows the breakdown of the scores that make up the consistency category. Consistency is an important measure of the quality of metabolic network reconstruction. The stoichiometric consistency, mass balance, charge balance, and metabolic connectivity of all Reconstructor models were 100% due to the curation of the universal database to remove unbalanced reactions. The unbounded flux in default medium was on par with ModelSEED and CarveMe scores.

We achieved a stoichiometric consistency score of 100% for all models due to the curation of the universal reaction database. We used ModelSEED reactions in the universal database, but discovered that 4,913 reactions out of the 52,793 reactions in the modelSEED database were unbalanced. We have included the reaction IDs reaction definitions of these unbalanced reactions in this supplement. After removing the unbalanced reactions from the universal reaction database, we no longer had any unconserved metabolites, resulting in a stoichiometric consistency score of 100%.

Figure S1 B shows that Reconstructor MEMOTE scores across all MEMOTE categories are higher than comparable ModelSEED and CarveMe reconstructions, including consistency. We also included uniform metabolite annotations and reaction annotations in the ModelSEED namespace as well as KEGG gene annotations. These additional annotations result in correspondingly higher scores. We also included thorough SBO term annotations.

**Figure S1**. MEMOTE Comparison between Reconstructor, ModelSEED, and CarveMe reconstructions. MEMOTE scores are averaged across 10 reconstructions. A) Consistency sub-category comparison, B) General MEMOTE score sub-category comparison, C) Overall MEMOTE score comparison

Figure S1 C shows the overall total MEMOTE score (averaged across 10 reconstructions) for Reconstructor network reconstructions is higher than the ModelSEED and CarveMe for comparable reconstructions. Because of our high stoichiometric consistency score and well annotated metabolites, reactions, genes, and SBO terms, our reconstructions achieve high MEMOTE scores compared to comparable ModelSEED and CarveMeOverall, the high Reconstructor MEMOTE scores could be attributed to the pFBA gap-filling approach rather than minimization of the number of included reactions (like ModelSEED uses), the well-curated reaction database, or through annotation.

**Reconstructor reconstructions capture class-, species-, and strain-level variations in functional metabolism**

We generated reconstructions of 5 strains each of *M. tuberculosis, R. rickettsii, E. coli, and P. aeruginosa,* for a total of 20 reconstructions*.* We then performed flux sampling using gapsplit() (Keaty *et al.*, 2020) to generate 500 flux samples per reconstruction, representative of the distribution of functional metabolic phenotypes of a given strain. We then used NMDS for dimensionality reduction of flux samples and for visualization in two dimensions (Figure 1C). This analysis shows distinct clusters within a species for each strain, suggesting that Reconstructor is able to capture strain-specific metabolic functional differences. Additionally, broader clusters based on species are apparent suggesting that Reconstructor can capture species-specific functional metabolic differences. Furthermore, the clusters of Gammaproteobacteria species are linearly separable from the clusters of Actinomycetia and Alphaproteobacteria suggesting that Reconstructor is also capable of capturing higher taxonomic differences in functional metabolism.

**Essential gene analysis for hypothesis generation**

GENREs can be used for a wide array of applications. Here we show one application of GENREs created from Reconstructor that are directly integrated with COBRApy for fast experimentally-testable hypothesis generation.

We generated a reconstruction of *Pseudomonas aeruginosa* from a genome sequence obtained from BV-BRC. The genome sequence used here was from the *Pseudomonas aeruginosa* NCGM2.S1 strain. We then determined genes that are essential for biomass production (growth) using the cobra.flux_analysis.variablility.find_essential_genes() function. The list of essential genes that were determined through this screening are below in Table S1. This table was adapted for Figure 1E. We then converted the essential genes to KEGG orthologs and their corresponding gene name. Then, we used the gene names as target search queries in DrugBank to determine if there are any existing drugs that are associated with the gene names. While some genes had existing drugs relations, others did not. Drugs that were related to the essential genes were then used in a literature search to see if previous studies determined that these drugs do indeed have antimicrobial properties against *Pseudomonas aeruginosa* or related bacterial species.

Some of our identified drugs that are related to the essential genes in our *Pseudomonas aeruginosa* reconstruction have been previously determined to have antimicrobial properties. This result suggests that reconstructions built with Reconstructor are capable of generating predictions that have already been verified in previous studies (cited in Figure 1E of main manuscript). However, some of the identified drugs have not been shown to have antimicrobial properties previously. This result suggests that Reconstructor is also capable of generating testable hypotheses for the discovery of novel antimicrobial therapies.

| **Essential Gene** | **KEGG Ortholog** | **Gene Name** | **DrugBank “Drug Relations”** | **Known Antimicrobial?** |
| --- | --- | --- | --- | --- |
| mmd:GYY | K00196 | cooF | None | N/A |
| paf:PAM18_3288 | K01665 | pabB | Formic acid | Yes |
| pau:PA14_25710 | K02619 | pabC | **D-[3-hydroxy-2-methyl-5-phosphonooxymethyl-pyridin-4-ylmethyl]-N,O-cycloserylamide** | **No** |
| pae:PA0551 | K03472 | epd | None | N/A |
| paf:PAM18_1805 | K00831 | serC | Triethylene glycol | Yes |
| pae:PA1681 | K01736 | aroC | Flavin mononucleotide, Cobalt hexamine ion | No, No |
| pnc:NCGM2_2268 | K03473 | pdxB | None | N/A |
| pnc:NCGM2_6085 | K13038 | coaC | None | N/A |
| pae:PA4044 | K01662 | dxs | None | N/A |
| pae:PA1806 | K00208 | fabI | Soneclosan, Triclosan | No, Yes |
| pnc:NCGM2_0809 | K01918 | panC | **Tris-Hydroxymethyl-Methyl-Ammonium, 2,4-Dihydroxy-3,3-Dimethyl-Butyrate, alpha,beta-Methyleneadenosine 5'-triphosphate, Pantoyl Adenylate, beta-Alanine** | **Yes, No, No, No, No** |
| pnc:NCGM2_1075 | K00767 | nadC | Quinolinic acid | Yes |
| pnc:NCGM2_6101 | K00942 | gmk | Formic acid | Yes |
| pae:PA5331 | K00762 | pyrE | **5-O-phosphono-alpha-D-ribofuranosyl diphosphate, Orotic acid** | **No, No** |
| pae:PA0402 | K00609 | pyrB | Alanosine, Sparfosic acid | Yes, No |
| dka:DKAM_0474 | K17828 | pyrD | Formic acid, Orotic acid, Flavin mononucleotide | Yes, No, No |
| wsu:WS1004 | K00392 | sir | None | N/A |
| pae:PA2023 | K00963 | galU | None | N/A |

**Table S1.** Essential gene analysis for *Pseudomonas aeruginosa* reconstruction

**GENRE construction on different media formulations**

To ensure Reconstructor generates high quality GENREs regardless of the user-defined media formulation we generated *C. difficile* reconstructions on five media formulations. These include three media formulations that are built into Reconstructor (complete, minimal, and rich), and two *C. difficile* specific media formulations (minimal defined *C. difficile* media, enriched defined *C. difficile* media). All resulting GENREs had an overall MEMOTE score of ~84%. This result shows that Reconstructor is able to generate high quality reconstructions regardless of media formulation.

We have included the media formulations on the Reconstructor github (<https://github.com/emmamglass/reconstructor>). Furthermore, we have included the resulting reconstructions on our GitHub (<https://github.com/emmamglass/reconstructor/tree/main/C_difficile_media>), as well as the arguments used to create the reconstructions.

**Unbalanced ModelSEED database reactions**

Below is the list of 4,913 ModelSEED reactions that we determine were unbalanced, and subsequently removed from our universal reaction database.

rxn00073_c: cpd00003_c + cpd00067_c + 2.0 cpd00423_c <=> cpd00004_c + 2.0 cpd03424_c

rxn00081_c: cpd00005_c + 2.0 cpd03424_c <=> cpd00006_c + cpd00067_c + 2.0 cpd00423_c

rxn00110_c: cpd00004_c + cpd00007_c + cpd03612_c <=> cpd00003_c + cpd00013_c + cpd00049_c

rxn00111_c: cpd00005_c + cpd00007_c + cpd03612_c <=> cpd00006_c + cpd00013_c + cpd00049_c

rxn00115_c: cpd00011_c + cpd00013_c <=> cpd00150_c

rxn00277_c: cpd00033_c <=> cpd00150_c

rxn00296_c: cpd00037_c <=> cpd00273_c

rxn00349_c: cpd00041_c <=> cpd03077_c

rxn00496_c: cpd00066_c <-- cpd01647_c

rxn00664_c: cpd00085_c --> cpd02333_c

rxn00665_c: cpd00085_c <=> cpd03376_c

rxn00666_c: cpd00085_c <-- cpd03377_c

rxn00667_c: cpd00086_c + 6.0 cpd01675_c <=> 7.0 cpd00010_c + 6.0 cpd00011_c + cpd02075_c

rxn00681_c: cpd00040_c + cpd00086_c <-- cpd01709_c

rxn00689_c: cpd00002_c + cpd00023_c + cpd00087_c <=> cpd00008_c + cpd00009_c + cpd00067_c + cpd06227_c

rxn00732_c: cpd00093_c --> cpd03465_c

rxn00733_c: cpd00093_c <=> cpd03466_c

rxn00759_c: cpd00001_c + cpd01713_c <=> cpd00013_c + cpd00020_c + cpd00067_c

rxn00760_c: cpd00013_c + cpd00020_c + cpd00067_c <=> cpd00001_c + cpd01714_c

rxn00761_c: cpd00004_c + cpd00007_c + cpd01574_c --> cpd00003_c + cpd00011_c + cpd00077_c

rxn00820_c: cpd00075_c + cpd00109_c <=> cpd00110_c + cpd00209_c

rxn00828_c: cpd00112_c <=> cpd00273_c

rxn00969_c: cpd00001_c + cpd01582_c <=> cpd00067_c + cpd00136_c

rxn01040_c: cpd00153_c --> cpd00456_c

rxn01047_c: cpd00156_c --> cpd00287_c

rxn01120_c: cpd00175_c <=> cpd00273_c

rxn01198_c: cpd00197_c --> cpd01020_c

rxn01235_c: cpd00210_c <-- cpd01345_c

rxn01266_c: cpd00218_c --> cpd01876_c

rxn01272_c: cpd01997_c --> cpd00220_c

rxn01342_c: cpd00237_c --> cpd03141_c

rxn01400_c: cpd00263_c --> cpd03608_c + cpd03738_c

rxn01432_c: cpd00134_c + cpd00273_c <=> cpd00010_c + cpd02157_c

rxn01433_c: cpd00273_c <=> cpd02319_c

rxn01470_c: cpd00283_c <-- cpd03616_c

rxn01526_c: cpd00301_c <-- cpd00776_c

rxn01533_c: cpd00304_c <=> cpd03512_c

rxn01534_c: cpd00304_c <=> cpd03513_c

rxn01591_c: 2.0 cpd00004_c + 2.0 cpd00067_c + cpd00070_c + cpd00327_c <-- 2.0 cpd00003_c + cpd00011_c + cpd01393_c

rxn01592_c: 2.0 cpd00005_c + 2.0 cpd00067_c + cpd00070_c + cpd00327_c <-- 2.0 cpd00006_c + cpd00011_c + cpd01393_c

rxn01659_c: cpd00350_c --> cpd00012_c + cpd03624_c

rxn01736_c: cpd00048_c + cpd00381_c <=> cpd00084_c + cpd03456_c

rxn01766_c: 2.0 cpd00005_c + 2.0 cpd00007_c + 2.0 cpd00067_c + cpd00398_c --> 2.0 cpd00006_c + cpd03507_c

rxn01796_c: cpd00047_c + cpd00410_c <=> cpd00055_c + cpd00110_c

rxn01897_c: cpd00001_c + cpd02521_c <=> cpd00013_c + cpd00067_c + cpd00461_c

rxn01915_c: cpd00009_c + cpd02110_c <-- cpd00300_c + cpd00475_c

rxn01958_c: cpd00495_c --> cpd03596_c

rxn01963_c: cpd00020_c + cpd00102_c <-- cpd02636_c

rxn01992_c: cpd00002_c + cpd00067_c + cpd01297_c <=> cpd00008_c + cpd00517_c

rxn02001_c: cpd00521_c <-- cpd03171_c

rxn02025_c: cpd00007_c + cpd00538_c --> cpd02466_c

rxn02027_c: cpd00054_c <-- cpd00547_c

rxn02047_c: cpd00051_c + cpd00550_c --> cpd00064_c + cpd01190_c

rxn02152_c: cpd00006_c + cpd00150_c + cpd00635_c <=> cpd00005_c + cpd03424_c

rxn02162_c: cpd00002_c + cpd00644_c <-- cpd00008_c + cpd00834_c

rxn02240_c: 2.0 cpd00005_c + 2.0 cpd00007_c + 2.0 cpd00067_c + cpd00717_c --> 2.0 cpd00006_c + cpd01858_c

rxn02252_c: cpd00728_c --> cpd01557_c

rxn02256_c: cpd00173_c + cpd00736_c <=> cpd00012_c + cpd01125_c

rxn02368_c: cpd00004_c + cpd00007_c + cpd00067_c + cpd01972_c --> cpd00003_c + cpd00854_c

rxn02422_c: cpd00038_c + cpd00889_c <=> cpd00031_c + cpd02137_c

rxn02485_c: cpd00939_c --> cpd02140_c

rxn02553_c: cpd00001_c + cpd00003_c + cpd01105_c <-- cpd00004_c + 3.0 cpd00067_c + cpd01056_c

rxn02572_c: cpd01079_c <=> cpd03387_c

rxn02573_c: cpd01079_c <=> cpd03396_c

rxn02605_c: cpd01155_c --> cpd03691_c

rxn02626_c: cpd01072_c <=> cpd03684_c

rxn02627_c: cpd01196_c <=> cpd03685_c

rxn02657_c: cpd01280_c <=> cpd03623_c

rxn02664_c: cpd01294_c --> cpd03640_c

rxn02692_c: cpd01370_c --> cpd03622_c

rxn02698_c: cpd01389_c <=> cpd00011_c + cpd01390_c

rxn02714_c: cpd01430_c <=> cpd03513_c

rxn02723_c: cpd00170_c + cpd00326_c --> cpd00010_c + cpd02223_c

rxn02731_c: cpd01468_c <-- cpd00287_c

rxn02736_c: cpd01481_c + cpd02450_c <=> cpd00014_c + cpd02577_c

rxn02757_c: cpd01547_c <-- cpd03369_c

rxn02777_c: cpd00244_c + cpd01620_c <=> cpd03425_c

rxn02781_c: cpd01625_c <-- cpd03461_c

rxn02799_c: 2.0 cpd00005_c + cpd00007_c + 2.0 cpd00067_c + cpd01685_c <=> 2.0 cpd00006_c + cpd00099_c + cpd02174_c

rxn02813_c: cpd01415_c + cpd01714_c <=> cpd00067_c + cpd02336_c

rxn02824_c: cpd00134_c + cpd01752_c <=> cpd00010_c + cpd02165_c

rxn02846_c: cpd01807_c <-- cpd03378_c

rxn02859_c: 2.0 cpd00001_c + 2.0 cpd00007_c + cpd01866_c <-- 2.0 cpd00025_c + cpd03472_c + cpd03473_c

rxn02870_c: cpd00001_c + cpd03822_c <=> cpd00067_c + cpd01892_c

rxn02918_c: cpd00026_c + cpd02086_c <=> cpd00067_c + cpd00091_c + cpd02831_c

rxn02919_c: cpd00001_c + cpd02831_c <=> cpd00067_c + cpd00089_c + cpd02086_c

rxn02953_c: cpd00002_c + cpd00084_c + cpd00834_c --> cpd00008_c + cpd00009_c + cpd00067_c + cpd02666_c

rxn02954_c: cpd00052_c + cpd00084_c + cpd00834_c --> cpd00012_c + cpd00046_c + cpd02666_c

rxn02955_c: cpd00052_c + cpd00084_c + cpd00834_c --> cpd00009_c + cpd00067_c + cpd00096_c + cpd02666_c

rxn02963_c: cpd02233_c --> cpd03381_c

rxn02964_c: cpd02233_c <-- cpd03382_c

rxn02965_c: cpd02024_c --> cpd03385_c

rxn02966_c: cpd02233_c --> cpd03386_c

rxn02968_c: cpd02252_c --> cpd03498_c

rxn02984_c: cpd02311_c --> cpd00013_c + cpd00047_c + cpd03303_c

rxn02987_c: cpd00001_c + cpd02849_c <-- cpd00029_c + cpd02316_c

rxn03006_c: 4.0 cpd00001_c + 3.0 cpd00006_c + cpd00011_c + cpd00877_c <-- 3.0 cpd00007_c + cpd03041_c

rxn03028_c: 2.0 cpd00001_c + 4.0 cpd00002_c + cpd00137_c + 2.0 cpd02456_c <-- 4.0 cpd00008_c + cpd03294_c

rxn03036_c: cpd00001_c + cpd02896_c <=> cpd00067_c + cpd00122_c + cpd02486_c

rxn03081_c: cpd00364_c + cpd02656_c --> cpd00415_c + cpd03568_c

rxn03083_c: cpd00025_c + cpd02802_c --> 4.0 cpd00001_c + cpd01483_c + cpd02669_c

rxn03103_c: cpd02753_c <-- cpd00067_c + cpd02705_c

rxn03118_c: cpd02805_c <-- cpd03603_c

rxn03121_c: cpd00001_c + cpd02903_c <=> cpd00054_c + cpd02808_c

rxn03128_c: cpd00086_c + cpd03164_c <-- cpd00010_c + cpd02830_c

rxn03163_c: cpd02949_c --> cpd03539_c

rxn03172_c: 2.0 cpd00001_c + cpd02978_c <-- 2.0 cpd00009_c + cpd03520_c

rxn03189_c: cpd03038_c --> cpd03040_c

rxn03190_c: 2.0 cpd00011_c + cpd03221_c --> 6.0 cpd00005_c + 6.0 cpd00007_c + 6.0 cpd00067_c

rxn03191_c: cpd00005_c + cpd03039_c <=> cpd00006_c + cpd03040_c

rxn03225_c: cpd03092_c <-- cpd00011_c + cpd03437_c

rxn03237_c: 3.0 cpd00001_c + cpd03111_c <=> 3.0 cpd00009_c + cpd03112_c

rxn03262_c: cpd03157_c <=> cpd03683_c

rxn03266_c: cpd03163_c <=> cpd03406_c

rxn03280_c: cpd01449_c + cpd03214_c <=> cpd00012_c + cpd00254_c + cpd03436_c

rxn03285_c: cpd03229_c --> cpd03249_c

rxn03348_c: cpd00004_c + cpd00430_c --> cpd00003_c + cpd03641_c

rxn03349_c: cpd00001_c + cpd00006_c + cpd00430_c --> cpd00005_c + cpd03642_c

rxn03350_c: cpd00882_c <-- cpd03343_c

rxn03357_c: cpd03346_c <=> cpd03463_c

rxn03370_c: cpd03382_c <=> cpd03383_c

rxn03377_c: cpd03389_c <=> cpd03404_c

rxn03386_c: cpd00002_c + cpd00038_c + cpd03422_c + cpd03423_c <=> cpd00008_c + cpd00012_c + 2.0 cpd00067_c + cpd00126_c + cpd03424_c

rxn03399_c: cpd02494_c + cpd03452_c <=> cpd03453_c

rxn03401_c: cpd03466_c <=> cpd03467_c

rxn03404_c: cpd03480_c --> cpd03714_c

rxn03417_c: cpd03514_c <=> cpd03516_c

rxn03418_c: cpd03515_c <=> cpd03516_c

rxn03420_c: cpd00047_c + cpd03518_c <-- cpd03519_c

rxn03449_c: cpd03616_c --> cpd03617_c

rxn03450_c: cpd03617_c --> cpd03618_c

rxn03451_c: cpd03626_c --> cpd03627_c

rxn03475_c: cpd03682_c <=> cpd03683_c

rxn03476_c: cpd03683_c <=> cpd03685_c

rxn03480_c: cpd03692_c <-- cpd03694_c

rxn03515_c: 2.0 cpd00005_c + cpd00022_c + 2.0 cpd00067_c + cpd00070_c <=> 2.0 cpd00006_c + 2.0 cpd00010_c + cpd00011_c + cpd00487_c

rxn03520_c: cpd02024_c --> cpd03873_c

rxn03521_c: cpd03873_c --> cpd03874_c

rxn03522_c: cpd03874_c <=> cpd03875_c

rxn03539_c: 2.0 cpd00001_c + 2.0 cpd00002_c + 2.0 cpd00053_c + cpd03832_c <=> 2.0 cpd00009_c + cpd03913_c

rxn03543_c: cpd00001_c + 2.0 cpd00011_c + 2.0 cpd00036_c + cpd01995_c <-- 2.0 cpd00024_c + cpd03972_c

rxn03579_c: 2.0 cpd00042_c + cpd04005_c <=> cpd00111_c + cpd00415_c

rxn03582_c: cpd00007_c + cpd02075_c --> cpd04039_c

rxn03645_c: cpd00004_c + cpd03058_c --> cpd00006_c + cpd03267_c

rxn03647_c: cpd04051_c --> cpd00011_c + cpd04052_c

rxn03648_c: cpd01420_c --> cpd05499_c

rxn03656_c: cpd00004_c + cpd03770_c <=> cpd00003_c + cpd01498_c

rxn03664_c: cpd00001_c + cpd00099_c + cpd04049_c <=> cpd04050_c + cpd08208_c

rxn03672_c: cpd00001_c + cpd04018_c <=> cpd00191_c

rxn03673_c: cpd00001_c + cpd04019_c <=> cpd00191_c

rxn03689_c: cpd04377_c <-- cpd04378_c

rxn03692_c: cpd02254_c <-- cpd02705_c

rxn03761_c: cpd04033_c --> cpd04032_c

rxn03767_c: cpd04063_c --> cpd04064_c

rxn03769_c: cpd04055_c <=> cpd01582_c

rxn03770_c: cpd04045_c --> cpd01972_c

rxn03771_c: cpd04050_c --> cpd01972_c

rxn03776_c: cpd04044_c <-- cpd04047_c

rxn03777_c: cpd04050_c <-- cpd04051_c

rxn03784_c: cpd00005_c + cpd00007_c + cpd00015_c + cpd00067_c + cpd04358_c <-- cpd00001_c + cpd00006_c + cpd01413_c

rxn03803_c: cpd00064_c + cpd00825_c --> cpd00051_c + cpd00950_c

rxn03810_c: cpd01313_c <-- cpd04056_c

rxn03811_c: cpd04020_c <-- cpd04037_c

rxn03812_c: cpd04021_c <-- cpd04022_c

rxn03813_c: cpd04022_c <-- cpd04023_c

rxn03834_c: 2.0 cpd00011_c + 2.0 cpd04861_c <-- cpd00007_c + 2.0 cpd04862_c

rxn03844_c: cpd00001_c + cpd03959_c <-- cpd03960_c

rxn03857_c: cpd00001_c + cpd03964_c <-- cpd03965_c

rxn03900_c: cpd00002_c + cpd01352_c --> cpd00008_c + cpd00067_c + cpd00286_c

rxn03937_c: cpd01034_c --> cpd01042_c

rxn03940_c: cpd04323_c <-- cpd04324_c

rxn03945_c: cpd04328_c <-- cpd04329_c

rxn03965_c: 2.0 cpd00007_c + 4.0 cpd00067_c + cpd00182_c --> 2.0 cpd00025_c + cpd08340_c

rxn03967_c: cpd00007_c + 4.0 cpd00067_c + cpd08339_c --> cpd00025_c + cpd08340_c

rxn03987_c: cpd00007_c + cpd00067_c <-- cpd01054_c

rxn04053_c: 2.0 cpd00001_c + 2.0 cpd00002_c + 2.0 cpd00053_c + cpd03421_c <=> 2.0 cpd00023_c + cpd03914_c

rxn04055_c: cpd00007_c + 4.0 cpd00067_c + 2.0 cpd01365_c --> 2.0 cpd00001_c + 2.0 cpd08442_c

rxn04072_c: cpd00229_c <-- cpd00263_c

rxn04075_c: cpd00003_c + cpd08276_c --> cpd00004_c + cpd00007_c + cpd00067_c + cpd02726_c

rxn04091_c: cpd04440_c <=> cpd04442_c

rxn04098_c: cpd08487_c <=> cpd08488_c

rxn04102_c: cpd08453_c <=> cpd08488_c

rxn04103_c: cpd06139_c <=> cpd08453_c

rxn04115_c: cpd06139_c <=> cpd08616_c

rxn04116_c: cpd04514_c <-- cpd08617_c

rxn04117_c: cpd08617_c <-- cpd06225_c

rxn04118_c: cpd04512_c <-- cpd08618_c

rxn04119_c: cpd08618_c <-- cpd08619_c

rxn04120_c: cpd04512_c <-- cpd08620_c

rxn04121_c: cpd08620_c <-- cpd06316_c

rxn04122_c: cpd08620_c <-- cpd06285_c

rxn04123_c: cpd08620_c <-- cpd06143_c

rxn04125_c: cpd08621_c --> cpd06223_c

rxn04128_c: cpd08483_c --> cpd08484_c

rxn04129_c: cpd08484_c <=> cpd06661_c

rxn04138_c: cpd03077_c <=> cpd00013_c + cpd08627_c

rxn04145_c: cpd03630_c <=> cpd03631_c

rxn04146_c: cpd03630_c <=> cpd03632_c

rxn04163_c: cpd08632_c <=> cpd08648_c

rxn04186_c: cpd08700_c <=> cpd08688_c

rxn04187_c: cpd08700_c <=> cpd08689_c

rxn04188_c: cpd08700_c <=> cpd08690_c

rxn04189_c: cpd08688_c <=> cpd03628_c

rxn04190_c: cpd08689_c <=> cpd03628_c

rxn04191_c: cpd08695_c --> cpd08697_c

rxn04192_c: cpd08696_c --> cpd08697_c

rxn04193_c: cpd08698_c <-- cpd04580_c

rxn04194_c: cpd00289_c <-- cpd08691_c

rxn04197_c: cpd08656_c <=> cpd08657_c

rxn04199_c: cpd08659_c <=> cpd00011_c + cpd08661_c

rxn04200_c: cpd08660_c <=> cpd00011_c + cpd08662_c

rxn04201_c: cpd03633_c <=> cpd08703_c

rxn04202_c: cpd08655_c <=> cpd03634_c

rxn04203_c: cpd08658_c <=> cpd03635_c

rxn04204_c: cpd08659_c <=> cpd01389_c

rxn04205_c: cpd08661_c <=> cpd01390_c

rxn04206_c: cpd08655_c <=> cpd08656_c

rxn04207_c: cpd08658_c <=> cpd08657_c

rxn04212_c: cpd08651_c <=> cpd08656_c

rxn04213_c: cpd08703_c <=> cpd03634_c

rxn04214_c: cpd03633_c <=> cpd08651_c

rxn04220_c: cpd08669_c <=> cpd01172_c

rxn04221_c: cpd08669_c <=> cpd08654_c

rxn04223_c: cpd08664_c <=> cpd08665_c

rxn04224_c: cpd08676_c --> cpd08680_c

rxn04225_c: cpd08677_c --> cpd08681_c

rxn04226_c: cpd08678_c --> cpd08682_c

rxn04230_c: cpd08685_c --> cpd03627_c

rxn04231_c: cpd08684_c --> cpd08686_c

rxn04232_c: cpd08683_c --> cpd08687_c

rxn04252_c: cpd08252_c --> cpd08256_c

rxn04253_c: cpd08266_c --> cpd08268_c

rxn04254_c: cpd08255_c --> cpd08257_c

rxn04255_c: cpd08269_c --> cpd08272_c

rxn04256_c: cpd08256_c --> cpd08258_c

rxn04257_c: cpd08257_c --> cpd08260_c

rxn04258_c: cpd08268_c --> cpd08271_c

rxn04259_c: cpd08272_c --> cpd08274_c

rxn04263_c: cpd08273_c --> cpd08275_c

rxn04272_c: cpd00002_c + cpd00010_c + cpd08741_c <-- cpd00009_c + cpd00018_c + cpd08743_c

rxn04273_c: cpd00002_c + cpd00010_c + cpd08740_c <-- cpd00009_c + cpd00018_c + cpd08742_c

rxn04283_c: cpd00283_c <-- cpd08247_c

rxn04287_c: cpd08706_c <=> cpd08707_c

rxn04304_c: cpd08729_c <-- cpd08716_c

rxn04305_c: cpd08727_c <-- cpd08715_c

rxn04306_c: cpd00522_c <-- cpd08724_c

rxn04309_c: 2.0 cpd00070_c + cpd00086_c + 5.0 cpd01675_c <-- 8.0 cpd00010_c + 7.0 cpd00011_c + cpd08797_c

rxn04310_c: cpd00005_c + cpd00007_c + cpd08798_c --> cpd00001_c + cpd00006_c + cpd08799_c

rxn04314_c: cpd00022_c + 6.0 cpd01675_c <=> 7.0 cpd00010_c + 6.0 cpd00011_c + cpd08786_c

rxn04319_c: cpd00070_c + cpd00086_c + cpd01675_c <-- cpd00010_c + cpd00011_c + cpd08790_c

rxn04320_c: cpd00070_c + cpd00086_c + cpd01675_c <-- cpd00010_c + cpd00011_c + cpd08794_c

rxn04341_c: 7.0 cpd00070_c + cpd00760_c + 5.0 cpd01675_c <=> 13.0 cpd00010_c + 12.0 cpd00011_c + cpd08766_c

rxn04342_c: 7.0 cpd00070_c + cpd00760_c + 5.0 cpd01675_c <=> 13.0 cpd00010_c + 12.0 cpd00011_c + cpd08774_c

rxn04343_c: 7.0 cpd00070_c + cpd00481_c + 5.0 cpd01675_c <=> 13.0 cpd00010_c + 12.0 cpd00011_c + cpd08750_c

rxn04344_c: 7.0 cpd00070_c + cpd00481_c + 5.0 cpd01675_c <=> 13.0 cpd00010_c + 12.0 cpd00011_c + cpd08758_c

rxn04364_c: cpd08782_c --> cpd08783_c

rxn04365_c: cpd08782_c --> cpd08784_c

rxn04366_c: cpd08782_c <=> cpd08785_c

rxn04399_c: cpd05511_c <-- cpd08878_c

rxn04400_c: cpd05546_c <-- cpd08879_c

rxn04401_c: cpd05511_c --> cpd05546_c

rxn04402_c: cpd08878_c --> cpd08879_c

rxn04403_c: cpd05511_c <-- cpd08923_c

rxn04404_c: cpd08920_c <-- cpd08921_c

rxn04410_c: cpd06654_c --> cpd03971_c

rxn04412_c: 2.0 cpd00005_c + 2.0 cpd00007_c + 2.0 cpd00067_c + cpd01071_c --> 2.0 cpd00006_c + cpd06996_c

rxn04414_c: 2.0 cpd00005_c + 2.0 cpd00007_c + 2.0 cpd00067_c + cpd01047_c <=> 2.0 cpd00006_c + cpd07081_c

rxn04420_c: cpd00582_c <-- cpd01193_c

rxn04422_c: cpd00192_c <-- cpd05556_c

rxn04433_c: cpd03332_c --> cpd07830_c

rxn04434_c: cpd01722_c --> cpd08986_c

rxn04435_c: cpd02595_c --> cpd00634_c

rxn04436_c: cpd01890_c --> cpd07322_c

rxn04438_c: cpd00321_c --> cpd08983_c

rxn04439_c: cpd08989_c <-- cpd08990_c

rxn04440_c: cpd08990_c <-- cpd08991_c

rxn04441_c: cpd08991_c --> cpd08992_c

rxn04450_c: cpd09025_c --> cpd09026_c

rxn04452_c: cpd08835_c --> cpd01271_c

rxn04460_c: cpd08906_c <=> cpd05481_c

rxn04461_c: cpd03504_c <=> cpd05558_c

rxn04467_c: cpd00033_c <=> cpd00067_c + cpd03077_c

rxn04469_c: cpd08665_c <=> cpd01172_c

rxn04470_c: cpd08662_c <=> cpd00640_c

rxn04474_c: 3.0 cpd00070_c <-- cpd00001_c + cpd09095_c

rxn04486_c: cpd09150_c <-- cpd09151_c

rxn04487_c: cpd09151_c --> cpd09153_c

rxn04488_c: cpd09150_c --> cpd09152_c

rxn04489_c: cpd09152_c <-- cpd09153_c

rxn04490_c: cpd09153_c --> cpd09154_c

rxn04491_c: cpd09149_c --> cpd09155_c

rxn04492_c: cpd09149_c --> cpd09156_c

rxn04493_c: cpd09149_c --> cpd09157_c

rxn04496_c: cpd09157_c --> cpd09160_c

rxn04498_c: cpd09157_c --> cpd09163_c

rxn04504_c: cpd09143_c <=> cpd09191_c

rxn04505_c: cpd09191_c <=> cpd09148_c

rxn04506_c: cpd09143_c <-- cpd09144_c

rxn04507_c: cpd09144_c <-- cpd09145_c

rxn04508_c: cpd09145_c <=> cpd09146_c

rxn04509_c: cpd09146_c <=> cpd09147_c

rxn04511_c: cpd09172_c --> cpd09173_c

rxn04515_c: cpd09176_c --> cpd09177_c

rxn04517_c: cpd09178_c --> cpd09179_c

rxn04519_c: cpd09180_c <-- cpd09181_c

rxn04520_c: cpd09181_c --> cpd09182_c

rxn04522_c: cpd01310_c --> cpd01151_c

rxn04523_c: cpd09180_c --> cpd09183_c

rxn04525_c: cpd09184_c <-- cpd01310_c

rxn04528_c: cpd09187_c <=> cpd09188_c

rxn04529_c: cpd09188_c --> cpd04092_c

rxn04530_c: cpd09188_c --> cpd00067_c + cpd09189_c

rxn04532_c: cpd09120_c <=> cpd09121_c

rxn04533_c: cpd09121_c <-- cpd09122_c

rxn04534_c: cpd09122_c <-- cpd09123_c

rxn04535_c: cpd09123_c <-- cpd09124_c

rxn04536_c: cpd09124_c --> cpd04173_c

rxn04538_c: cpd09120_c <-- cpd09126_c

rxn04540_c: cpd09120_c --> cpd09134_c

rxn04541_c: cpd09121_c <-- cpd09128_c

rxn04544_c: cpd09122_c --> cpd09132_c

rxn04545_c: cpd09122_c <-- cpd09131_c

rxn04546_c: cpd09124_c --> cpd09133_c

rxn04551_c: cpd00017_c --> cpd00019_c + cpd00067_c

rxn04552_c: cpd09139_c + 2.0 cpd09196_c <=> 2.0 cpd00297_c + cpd09141_c

rxn04553_c: cpd09141_c <=> cpd09142_c

rxn04554_c: cpd09190_c <=> cpd09202_c

rxn04555_c: cpd03802_c --> cpd09192_c

rxn04556_c: cpd09192_c --> cpd03687_c

rxn04557_c: 2.0 cpd00322_c + cpd03687_c <=> cpd03686_c

rxn04558_c: cpd00153_c + cpd03688_c <=> cpd01014_c

rxn04562_c: cpd00002_c + cpd00010_c + cpd09200_c <=> cpd00018_c + cpd09201_c

rxn04563_c: cpd03688_c <-- cpd03689_c

rxn04566_c: cpd00544_c --> cpd09203_c

rxn04567_c: cpd01404_c --> cpd09204_c

rxn04568_c: cpd01404_c <=> cpd07736_c

rxn04569_c: cpd00554_c --> cpd03934_c

rxn04570_c: cpd03691_c <-- cpd01070_c

rxn04573_c: cpd00323_c <-- cpd03695_c

rxn04574_c: cpd03690_c <-- cpd09205_c

rxn04576_c: cpd00333_c <=> cpd01014_c

rxn04581_c: cpd09207_c <-- cpd09210_c

rxn04582_c: cpd09210_c --> cpd09209_c

rxn04594_c: cpd00218_c <-- cpd07015_c

rxn04595_c: cpd03343_c --> cpd08304_c

rxn04596_c: cpd00333_c --> cpd09252_c

rxn04605_c: cpd00430_c --> cpd00489_c

rxn04606_c: cpd00430_c --> cpd03320_c

rxn04613_c: cpd08919_c --> cpd09271_c

rxn04637_c: cpd01047_c <=> cpd01038_c

rxn04638_c: cpd01038_c --> cpd09259_c

rxn04640_c: cpd01047_c <=> cpd01180_c

rxn04652_c: cpd00475_c --> cpd00847_c

rxn04655_c: cpd02243_c <-- cpd09317_c

rxn04656_c: cpd00705_c --> cpd09349_c

rxn04664_c: cpd00067_c + cpd03998_c <-- cpd04367_c

rxn04665_c: cpd00099_c + cpd04367_c <=> cpd04309_c

rxn04679_c: cpd00007_c + cpd09429_c --> cpd03092_c

rxn04689_c: cpd09476_c <-- cpd09477_c

rxn04690_c: cpd09476_c --> cpd09478_c

rxn04709_c: cpd09814_c --> cpd09815_c

rxn04711_c: cpd09804_c --> cpd02045_c

rxn04738_c: cpd09802_c --> cpd09803_c

rxn04747_c: cpd09805_c --> cpd09806_c

rxn04764_c: cpd09439_c --> cpd09440_c

rxn04768_c: cpd03624_c <-- cpd09442_c

rxn04775_c: cpd00001_c + cpd05640_c <=> cpd05639_c

rxn04798_c: cpd10419_c --> cpd09026_c

rxn04799_c: cpd09026_c <=> cpd01024_c + cpd10420_c

rxn04801_c: cpd00067_c + cpd10420_c <-- cpd10422_c

rxn04802_c: cpd10421_c <-- cpd10422_c

rxn04804_c: cpd10423_c --> cpd10425_c

rxn04805_c: cpd10425_c <-- cpd08835_c

rxn04812_c: cpd10488_c <-- cpd10493_c

rxn04813_c: cpd10490_c <-- cpd10494_c

rxn04814_c: cpd10490_c --> cpd10496_c

rxn04815_c: cpd10489_c <-- cpd10495_c

rxn04825_c: cpd08524_c --> cpd10482_c

rxn04835_c: cpd09767_c <=> cpd10485_c

rxn04837_c: cpd10486_c <-- cpd10487_c

rxn04897_c: cpd00042_c + cpd10562_c <=> cpd10560_c

rxn04899_c: cpd00001_c + cpd00099_c + cpd10556_c <=> cpd00067_c + cpd04135_c

rxn04906_c: cpd10564_c --> cpd08027_c

rxn04917_c: cpd10568_c --> cpd10569_c

rxn04918_c: cpd10568_c <=> cpd10570_c

rxn04921_c: cpd10572_c <-- cpd10573_c

rxn04922_c: cpd10571_c <=> cpd10574_c

rxn04926_c: cpd00067_c + cpd08028_c <-- cpd08027_c

rxn04929_c: cpd00059_c --> cpd10596_c

rxn04946_c: cpd00003_c + cpd01453_c <=> cpd00004_c + cpd00067_c + cpd00170_c

rxn05017_c: 11.0 cpd00005_c + cpd00017_c + cpd00022_c + 10.0 cpd00067_c + 8.0 cpd00070_c <-- cpd11211_c

rxn05019_c: cpd00005_c + cpd00022_c + cpd00067_c + 3.0 cpd00070_c <-- 4.0 cpd00010_c + 3.0 cpd00011_c + cpd01728_c

rxn05020_c: cpd00005_c + cpd00067_c + 3.0 cpd00070_c + cpd00192_c <-- 4.0 cpd00010_c + 3.0 cpd00011_c + cpd05556_c

rxn05027_c: cpd00026_c + cpd11219_c <=> cpd00014_c + cpd11220_c

rxn05075_c: cpd00017_c + cpd00593_c --> cpd00019_c + cpd01970_c

rxn05076_c: cpd00026_c + cpd00593_c --> cpd00014_c + cpd02520_c

rxn05077_c: cpd00026_c + cpd00570_c --> cpd00014_c + cpd02485_c

rxn05079_c: cpd00026_c + cpd01060_c --> cpd00014_c + cpd11240_c

rxn05083_c: cpd01052_c --> cpd00570_c

rxn05121_c: cpd11313_c <=> cpd11312_c

rxn05133_c: cpd11411_c --> cpd11412_c

rxn05134_c: cpd11412_c <-- cpd02876_c

rxn05139_c: cpd00192_c --> cpd00270_c

rxn05254_c: 0.1 cpd00134_c + 0.03 cpd00327_c + 0.03 cpd01695_c + 0.01 cpd11424_c + 0.07 cpd11432_c + 0.17 cpd11434_c + 0.01 cpd11435_c + 0.2 cpd11437_c + 0.34 cpd11439_c + 0.05 cpd11441_c <=> cpd00010_c + 0.01 cpd11422_c

rxn05260_c: cpd00037_c + 45.0 cpd00402_c + cpd00861_c <=> cpd00014_c + 45.0 cpd00046_c + 46.0 cpd00067_c + cpd00091_c + cpd11442_c

rxn05262_c: 45.0 cpd00001_c + 45.0 cpd00002_c + cpd00037_c + 45.0 cpd00117_c + 45.0 cpd00402_c + cpd00861_c <=> 45.0 cpd00012_c + cpd00014_c + 45.0 cpd00018_c + 45.0 cpd00046_c + 91.0 cpd00067_c + cpd00091_c + cpd11443_c

rxn05263_c: 30.0 cpd00001_c + 30.0 cpd00026_c + 30.0 cpd00175_c <=> 30.0 cpd00014_c + 60.0 cpd00067_c + 30.0 cpd00091_c + cpd11459_c

rxn05267_c: cpd00003_c + cpd02295_c + cpd02590_c <=> cpd00004_c + cpd00011_c + cpd00012_c + cpd00067_c + cpd11425_c

rxn05268_c: cpd00080_c + 0.01 cpd11427_c <=> cpd00046_c + cpd00067_c + 0.01 cpd11454_c

rxn05269_c: cpd00080_c + 0.1 cpd00134_c + 0.03 cpd00327_c + 0.03 cpd01695_c + 0.07 cpd11432_c + 0.17 cpd11434_c + 0.01 cpd11435_c + 0.2 cpd11437_c + 0.34 cpd11439_c + 0.05 cpd11441_c <=> cpd00010_c + 0.84 cpd00067_c + 0.01 cpd11424_c

rxn05270_c: cpd00001_c + 0.01 cpd11422_c <=> cpd00009_c + 0.01 cpd11423_c

rxn05271_c: cpd00001_c + 0.01 cpd11454_c <=> cpd00009_c + 0.01 cpd11453_c

rxn05272_c: cpd00052_c + cpd00067_c + 0.01 cpd11422_c <=> cpd00012_c + 0.01 cpd11427_c

rxn05273_c: 0.01 cpd11455_c <=> cpd00011_c + 0.01 cpd11456_c

rxn05281_c: cpd00054_c + 0.01 cpd11427_c <=> cpd00046_c + cpd00067_c + 0.01 cpd11455_c

rxn05282_c: 2.0 cpd00026_c + 0.01 cpd11423_c <=> 2.0 cpd00014_c + 2.0 cpd00067_c + 0.01 cpd11428_c

rxn05283_c: cpd00026_c + 0.01 cpd11423_c <=> cpd00014_c + cpd00067_c + 0.01 cpd11450_c

rxn05284_c: 3.0 cpd00026_c + 0.01 cpd11423_c <=> 3.0 cpd00014_c + 3.0 cpd00067_c + 0.01 cpd11458_c

rxn05294_c: 0.884 cpd00115_c + 0.6692 cpd00241_c + 0.6684 cpd00356_c + 0.8807 cpd00357_c <=> 3.1023 cpd00012_c + cpd11461_c

rxn05296_c: 0.4928 cpd00023_c + 0.7723 cpd00033_c + 0.5051 cpd00035_c + 0.6114 cpd00039_c + 0.2801 cpd00041_c + 0.3653 cpd00051_c + 0.4928 cpd00053_c + 0.4091 cpd00054_c + 0.2145 cpd00060_c + 0.1028 cpd00065_c + 0.3329 cpd00066_c + 0.2097 cpd00069_c + 0.1073 cpd00084_c + 0.6555 cpd00107_c + 0.1546 cpd00119_c + 0.3041 cpd00129_c + 0.2801 cpd00132_c + 0.5807 cpd00156_c + 0.3526 cpd00161_c + 0.5107 cpd00322_c <=> cpd11463_c

rxn05320_c: cpd00002_c + cpd00104_c + cpd00242_c <=> cpd00001_c + cpd00008_c + cpd00009_c + cpd11415_c

rxn05630_c: cpd00067_e <=> cpd00061_c + cpd00067_c

rxn05739_c: cpd00080_c + cpd11606_c <=> cpd00095_c + cpd11451_c

rxn05740_c: cpd00009_c + cpd00155_c <=> cpd00089_c + cpd15302_c

rxn05747_c: cpd00004_c + cpd00067_c + cpd11606_c <=> cpd00003_c + cpd11451_c

rxn05748_c: cpd00067_e + cpd00209_e + cpd11451_e <=> cpd00001_c + cpd00075_c + cpd11606_c

rxn05749_c: cpd00982_c + cpd11606_c <=> cpd00015_c + cpd11451_c

rxn05757_c: cpd00001_c + cpd11772_c <=> cpd12039_c

rxn05761_c: cpd00007_c + 2.0 cpd11650_c <=> 2.0 cpd00001_c + 2.0 cpd11709_c

rxn05776_c: cpd00001_c <-- cpd00129_c

rxn05777_c: cpd00002_c + cpd00013_c <=> cpd00008_c + 2.0 cpd00067_c + cpd12010_c

rxn05783_c: cpd11463_c <=> cpd11770_c

rxn05784_c: cpd11463_c <=> cpd12003_c

rxn05785_c: cpd11463_c <=> cpd12036_c

rxn05786_c: cpd11463_c <=> cpd12133_c

rxn05787_c: cpd11463_c <=> cpd12227_c

rxn05788_c: cpd11463_c <=> cpd12256_c

rxn05789_c: cpd11463_c <=> cpd12668_c

rxn05790_c: cpd11463_c <=> cpd12704_c

rxn05793_c: cpd00023_c <=> cpd12759_c

rxn05795_c: 2.0 cpd00001_c + 2.0 cpd00418_c + cpd11609_c <=> 2.0 cpd00075_c + cpd11610_c

rxn05796_c: cpd00004_c + cpd00067_c + cpd11609_c <=> cpd00003_c + cpd11610_c

rxn05797_c: cpd00005_c + cpd00067_c + cpd11609_c <=> cpd00006_c + cpd11610_c

rxn05798_c: cpd00001_c + 2.0 cpd00008_c + 2.0 cpd00009_c + cpd00013_c + cpd11609_c <=> 2.0 cpd00002_c + cpd00165_c + cpd11610_c

rxn05799_c: cpd00001_c + cpd00013_c + cpd11609_c <=> cpd00165_c + cpd11610_c

rxn05800_c: cpd00026_c <-- cpd00014_c

rxn05801_c: 2.0 cpd00418_c + cpd11610_c <=> cpd00001_c + cpd00659_c + cpd11609_c

rxn05802_c: 3.0 cpd00001_c + cpd01281_c + cpd11609_c <=> 3.0 cpd00081_c + cpd11610_c

rxn05803_c: cpd00001_c + cpd00204_c + cpd11609_c <=> cpd00011_c + cpd11610_c

rxn05804_c: cpd00221_c + cpd11609_c <=> cpd00020_c + cpd11610_c

rxn05805_c: cpd02041_c + cpd11609_c <=> cpd00024_c + cpd11610_c

rxn05806_c: cpd00027_c + cpd11609_c <=> cpd00170_c + cpd11610_c

rxn05807_c: cpd00001_c --> cpd00027_c

rxn05808_c: 3.0 cpd00007_c + cpd00028_c + 3.0 cpd11610_c <=> 3.0 cpd00001_c + cpd00204_c + cpd00389_c + cpd10515_c + 3.0 cpd11609_c

rxn05812_c: cpd00001_c + cpd00071_c + cpd11609_c <=> cpd00029_c + cpd11610_c

rxn05815_c: cpd00130_c + cpd11609_c <=> cpd00032_c + cpd11610_c

rxn05816_c: cpd00115_c <-- cpd00012_c

rxn05817_c: cpd00241_c <-- cpd00012_c

rxn05818_c: cpd00356_c <-- cpd00012_c

rxn05819_c: cpd00357_c <-- cpd00012_c

rxn05820_c: cpd00514_c <=> cpd00012_c

rxn05821_c: cpd00017_c + cpd11461_c <=> cpd00019_c + cpd12223_c

rxn05822_c: cpd00002_c --> cpd00012_c + cpd00018_c

rxn05823_c: cpd00003_c --> cpd00018_c + cpd00355_c

rxn05825_c: cpd00003_c + cpd11611_c <=> cpd00004_c + cpd00067_c + cpd11722_c

rxn05826_c: cpd00006_c + cpd11611_c <=> cpd00005_c + cpd00067_c + cpd11707_c

rxn05827_c: cpd00006_c + cpd11611_c <=> cpd00005_c + cpd00067_c + cpd11813_c

rxn05828_c: cpd00007_c + cpd11611_c <=> cpd00025_c + cpd11722_c

rxn05830_c: cpd00002_c + cpd00010_c + cpd11653_c <=> cpd00012_c + cpd00018_c + cpd11611_c

rxn05832_c: cpd11609_c + cpd11611_c <=> cpd11610_c + cpd11707_c

rxn05836_c: cpd00036_c + cpd11609_c <=> cpd00106_c + cpd11610_c

rxn05837_c: cpd00037_c + cpd11463_c <=> cpd00014_c + cpd12531_c

rxn05840_c: cpd00002_c <-- cpd00012_c

rxn05842_c: cpd00009_c --> cpd00008_c

rxn05843_c: cpd00009_c --> cpd00014_c

rxn05844_c: cpd00009_c --> cpd00031_c

rxn05845_c: cpd00009_c --> cpd00096_c

rxn05846_c: cpd00038_c <-- cpd00012_c

rxn05847_c: cpd00052_c <-- cpd00012_c

rxn05848_c: cpd00062_c <-- cpd00012_c

rxn05849_c: cpd00173_c <=> cpd00012_c

rxn05850_c: cpd00039_c --> cpd00323_c

rxn05851_c: cpd00139_c + cpd11609_c <=> cpd00040_c + cpd11610_c

rxn05852_c: cpd00043_c <-- cpd00014_c

rxn05857_c: cpd00001_c + cpd11609_c + cpd11616_c <=> cpd00049_c + cpd11610_c

rxn05861_c: cpd00017_c + cpd11615_c <=> cpd00019_c + cpd12321_c

rxn05862_c: cpd00017_c + cpd11615_c <=> cpd00019_c + cpd12481_c

rxn05863_c: cpd00017_c + cpd11615_c <=> cpd00019_c + cpd12482_c

rxn05864_c: cpd00017_c + cpd11615_c <=> cpd00019_c + cpd12483_c

rxn05865_c: cpd00017_c + cpd11615_c <=> cpd00019_c + cpd12484_c

rxn05866_c: cpd00017_c + cpd11615_c <=> cpd00019_c + cpd12485_c

rxn05867_c: cpd00017_c + cpd11615_c <=> cpd00019_c + cpd12486_c

rxn05868_c: cpd00017_c + cpd11615_c <=> cpd00019_c + cpd12598_c

rxn05869_c: cpd00001_c + cpd00771_c + cpd11609_c <=> cpd00023_c + cpd00055_c + cpd11610_c

rxn05870_c: cpd00001_c + cpd00183_c + cpd11609_c <=> cpd00033_c + cpd00055_c + cpd11610_c

rxn05884_c: cpd00195_c + cpd11609_c <=> cpd11610_c + cpd11616_c

rxn05886_c: cpd00024_c + cpd00064_c <-- cpd00023_c + cpd02651_c

rxn05888_c: cpd00044_c + cpd00067_c + cpd11610_c <=> cpd00008_c + cpd00081_c + cpd11609_c

rxn05896_c: cpd00075_c + cpd11609_c <=> cpd00209_c + cpd11610_c

rxn05898_c: cpd00076_c + cpd11609_c <=> cpd03412_c + cpd11610_c

rxn05900_c: cpd00080_c + cpd11609_c <=> cpd00095_c + cpd11610_c

rxn05901_c: cpd00067_c + cpd00080_c + cpd11611_c <=> cpd00010_c + cpd00517_c

rxn05903_c: cpd00018_c + cpd00081_c + cpd11609_c <=> cpd00193_c + cpd11610_c

rxn05904_c: 3.0 cpd00001_c + cpd00239_c + cpd11609_c <=> cpd00081_c + cpd11610_c

rxn05905_c: cpd00082_c + cpd11609_c <=> cpd00234_c + cpd11610_c

rxn05906_c: cpd00001_c --> cpd00082_c

rxn05907_c: cpd00083_c <-- cpd00031_c

rxn05911_c: cpd00001_c + cpd00092_c + cpd11609_c <=> cpd00605_c + cpd11610_c

rxn05912_c: cpd00001_c + cpd11619_c <=> cpd00009_c + cpd11662_c

rxn05913_c: cpd00001_c + cpd11713_c <=> cpd00009_c + 2.0 cpd00067_c + cpd11619_c

rxn05920_c: cpd00052_c + cpd11662_c <=> cpd00067_c + cpd00096_c + cpd11619_c

rxn05921_c: cpd00098_c + cpd11609_c <=> cpd00447_c + cpd11610_c

rxn05925_c: cpd00100_c + cpd11609_c <=> cpd00157_c + cpd11610_c

rxn05929_c: cpd00001_c --> cpd00108_c

rxn05931_c: cpd00112_c <=> cpd12619_c

rxn05933_c: cpd00113_c + cpd11615_c <=> cpd00012_c + cpd12546_c

rxn05936_c: cpd00120_c + cpd11609_c <=> cpd00650_c + cpd11610_c

rxn05941_c: cpd00001_c --> cpd00122_c

rxn05943_c: cpd00017_c + cpd11622_c <=> cpd00019_c + cpd00239_c

rxn05947_c: cpd00129_c + cpd11609_c <=> cpd02431_c + cpd11610_c

rxn05956_c: cpd00134_c + cpd11609_c <=> cpd03126_c + cpd11610_c

rxn05959_c: cpd00001_c + cpd00136_c + cpd11609_c <=> cpd00007_c + cpd00484_c + cpd11610_c

rxn05960_c: cpd00007_c + cpd01658_c + cpd11610_c <=> cpd00001_c + cpd00055_c + cpd00136_c + cpd11609_c

rxn05967_c: cpd00001_c + cpd11624_c <=> cpd00188_c + cpd12849_c

rxn05972_c: cpd00001_c + cpd12504_c <=> cpd00138_c + cpd11619_c

rxn05973_c: cpd00001_c --> cpd00138_c

rxn05980_c: cpd00001_c + cpd03077_c + cpd11609_c <=> cpd00013_c + cpd11610_c + cpd11625_c

rxn05985_c: cpd00004_c + cpd00007_c + cpd00049_c <=> cpd00003_c + cpd12347_c

rxn05986_c: cpd00005_c + cpd00007_c + cpd00049_c <=> cpd00006_c + cpd12347_c

rxn05987_c: cpd00049_c + cpd00100_c <=> cpd11677_c

rxn05991_c: cpd00001_c + cpd01526_c + cpd11609_c <=> cpd00013_c + cpd00143_c + cpd11610_c

rxn05992_c: cpd00144_c + cpd11609_c <=> cpd00014_c + cpd12241_c

rxn05996_c: cpd00002_c + cpd00487_c + cpd11493_c <=> cpd00012_c + cpd00018_c + cpd00067_c + cpd11628_c

rxn05998_c: cpd00067_c + cpd12754_c <=> cpd00150_c + cpd11616_c

rxn05999_c: cpd00001_c + cpd00151_c + cpd11609_c <=> cpd11610_c + cpd12692_c

rxn06000_c: cpd00154_c <-- cpd00001_c

rxn06001_c: cpd00158_c + cpd11609_c <=> cpd00801_c + cpd11610_c

rxn06006_c: cpd00001_c + cpd00003_c + cpd11631_c <=> cpd00004_c + cpd00067_c + cpd11694_c

rxn06009_c: cpd00001_c + cpd00007_c + cpd11631_c <=> cpd00025_c + cpd11694_c

rxn06010_c: cpd00001_c + cpd00006_c + cpd00012_c + cpd00018_c + cpd11631_c <=> cpd00002_c + cpd00005_c + cpd00067_c + cpd11694_c

rxn06014_c: cpd00007_c + cpd00456_c + cpd11610_c <=> cpd00001_c + cpd00168_c + cpd11609_c

rxn06017_c: cpd00001_c + cpd00756_c + cpd11609_c <=> cpd00055_c + cpd00183_c + cpd11610_c

rxn06019_c: cpd00186_c + cpd11608_c <=> cpd12155_c

rxn06021_c: 2.0 cpd00007_c + cpd00188_c + cpd11610_c <=> cpd00001_c + cpd00336_c + cpd11609_c

rxn06024_c: cpd00017_c + cpd11635_c <=> cpd00019_c + cpd12614_c

rxn06026_c: cpd00212_c + cpd11609_c <=> cpd00234_c + cpd11610_c

rxn06027_c: cpd00588_c + cpd11609_c <=> cpd00212_c + cpd11610_c

rxn06031_c: cpd00200_c + cpd00213_c <=> cpd00011_c + cpd14699_c

rxn06034_c: cpd00222_c + cpd11609_c <=> cpd00480_c + cpd11610_c

rxn06036_c: cpd00001_c --> cpd00224_c

rxn06039_c: cpd00078_c + cpd11926_c <=> cpd00036_c + cpd11637_c

rxn06042_c: cpd01519_c + cpd12055_c <=> cpd00010_c + cpd11427_c

rxn06048_c: cpd00007_c + cpd00232_c + cpd11610_c <=> cpd00001_c + cpd02155_c + cpd11609_c

rxn06049_c: cpd00009_c --> cpd00089_c

rxn06050_c: cpd00076_c <-- cpd00082_c

rxn06051_c: cpd11735_c <=> cpd12869_c

rxn06052_c: cpd00007_c + cpd00237_c + cpd11610_c <=> cpd00001_c + cpd02850_c + cpd11609_c

rxn06053_c: cpd00511_c + cpd11609_c <=> cpd00237_c + cpd11610_c

rxn06054_c: cpd02365_c + cpd11609_c <=> cpd00237_c + cpd11610_c

rxn06057_c: cpd00001_c + cpd11609_c + cpd11660_c <=> cpd00013_c + cpd11610_c + cpd11616_c

rxn06059_c: cpd00282_c + cpd11609_c <=> cpd00247_c + cpd11610_c

rxn06061_c: cpd00001_c + cpd00264_c + cpd11609_c <=> cpd00434_c + cpd00726_c + cpd11610_c

rxn06063_c: cpd00276_c <-- cpd00001_c

rxn06064_c: cpd00001_c --> cpd00280_c

rxn06069_c: cpd00001_c + 2.0 cpd00003_c + cpd11650_c <=> 2.0 cpd00004_c + 2.0 cpd00067_c + cpd11653_c

rxn06073_c: cpd00283_c <=> cpd03746_c

rxn06083_c: cpd00001_c + cpd12513_c <=> cpd00023_c + cpd11653_c

rxn06084_c: cpd00001_c + cpd12456_c <=> cpd00162_c + cpd11653_c

rxn06093_c: cpd00001_c <=> cpd11594_c

rxn06095_c: cpd11735_c <=> cpd11657_c

rxn06097_c: cpd00001_c + cpd11657_c <=> cpd00179_c + cpd11976_c

rxn06101_c: cpd00044_c + cpd12108_c <=> cpd00045_c + cpd11659_c

rxn06102_c: cpd00007_c + cpd11610_c + cpd11661_c <=> cpd00001_c + cpd11609_c + cpd12123_c

rxn06103_c: cpd00007_c + cpd11610_c + cpd11661_c <=> cpd00001_c + cpd11609_c + cpd12296_c

rxn06104_c: cpd00134_c + cpd11662_c <=> cpd00010_c + cpd12128_c

rxn06111_c: cpd15291_c <=> 2.0 cpd00067_c + cpd11669_c

rxn06113_c: cpd00019_c + cpd11669_c <=> cpd00017_c + cpd03442_c

rxn06116_c: cpd00175_c + cpd11671_c <=> cpd00014_c + cpd12535_c

rxn06117_c: cpd00002_c <-- cpd00008_c

rxn06118_c: cpd00027_c --> cpd00079_c

rxn06119_c: cpd11713_c <=> cpd11619_c

rxn06120_c: cpd00190_c --> cpd00863_c

rxn06121_c: cpd00203_c <-- cpd00169_c

rxn06127_c: cpd00323_c + cpd11609_c <=> cpd00352_c + cpd11610_c

rxn06128_c: cpd00007_c + cpd00325_c + cpd11610_c <=> cpd00001_c + cpd00866_c + cpd11609_c

rxn06129_c: cpd00007_c + cpd00325_c + cpd11610_c <=> cpd00001_c + cpd01936_c + cpd11609_c

rxn06130_c: cpd00007_c + cpd00325_c + cpd11610_c <=> cpd00001_c + cpd02047_c + cpd11609_c

rxn06131_c: cpd00007_c + cpd00325_c + cpd11610_c <=> cpd00001_c + cpd02348_c + cpd11609_c

rxn06132_c: cpd03260_c + cpd11609_c <=> cpd00325_c + cpd11610_c

rxn06137_c: cpd00329_c + cpd11609_c <=> cpd01596_c + cpd11610_c

rxn06140_c: cpd00010_c + 2.0 cpd00067_c + cpd11422_c <=> cpd00517_c + cpd11611_c

rxn06146_c: 2.0 cpd00067_c + cpd00336_c + cpd11609_c <=> cpd00488_c + cpd11610_c

rxn06150_c: cpd00350_c <=> cpd12823_c

rxn06151_c: cpd00001_e + cpd11683_e <=> cpd00029_c + cpd11741_c

rxn06152_c: cpd00001_c <=> cpd02217_c

rxn06153_c: cpd00037_c <-- cpd00014_c

rxn06156_c: cpd00001_c --> cpd01532_c

rxn06158_c: cpd00001_e + cpd11601_e <=> cpd00116_c + cpd11686_c

rxn06161_c: cpd00007_c + cpd00369_c + cpd11610_c <=> cpd00001_c + cpd01712_c + cpd11609_c

rxn06164_c: cpd00387_c + cpd11463_c <=> cpd00008_c + cpd00839_c

rxn06165_c: cpd00387_c <-- cpd00008_c

rxn06167_c: 2.0 cpd00001_c + 2.0 cpd00002_c + cpd00401_c + cpd11610_c <=> 2.0 cpd00008_c + 2.0 cpd00009_c + cpd03763_c + cpd11609_c

rxn06169_c: cpd00404_c <=> cpd11930_c

rxn06172_c: cpd00413_c + cpd11609_c <=> cpd00011_c + cpd00650_c + cpd11610_c

rxn06173_c: cpd00004_c + cpd00067_c + cpd00418_c <=> cpd00001_c + cpd00003_c + cpd00659_c

rxn06174_c: cpd02435_c + cpd11609_c <=> cpd00420_c + cpd11610_c

rxn06175_c: cpd03139_c + cpd11609_c <=> cpd00420_c + cpd11610_c

rxn06187_c: cpd00460_c + cpd11610_c <=> cpd03048_c + cpd11609_c

rxn06189_c: cpd00001_c + cpd03161_c + cpd11609_c <=> cpd00013_c + cpd00464_c + cpd11610_c

rxn06191_c: cpd00080_c + cpd11707_c <=> cpd00010_c + cpd00517_c

rxn06192_c: cpd00006_c + cpd00010_c + cpd11709_c <=> cpd00005_c + cpd12196_c

rxn06203_c: cpd01806_c + cpd11609_c <=> cpd00474_c + cpd11610_c

rxn06204_c: cpd01883_c + cpd11609_c <=> cpd00474_c + cpd11610_c

rxn06215_c: cpd00001_c + cpd12493_c <=> cpd00363_c + cpd00487_c

rxn06223_c: cpd11423_c + cpd11707_c <=> cpd00010_c + cpd11677_c

rxn06232_c: cpd00001_c + cpd02749_c <=> cpd00507_c + cpd11616_c

rxn06233_c: cpd00006_c + cpd00517_c <=> cpd00005_c + 2.0 cpd00067_c + cpd12300_c

rxn06235_c: cpd00517_c + cpd11707_c <=> cpd00010_c + cpd11422_c

rxn06236_c: cpd00003_c + cpd11836_c <=> cpd00004_c + cpd00067_c + cpd11726_c

rxn06237_c: cpd00006_c + cpd11836_c <=> cpd00005_c + cpd00067_c + cpd11726_c

rxn06238_c: cpd11492_c + cpd11628_c <=> cpd00011_c + cpd11493_c + cpd11726_c

rxn06241_c: cpd00794_c + cpd03729_c <=> cpd00523_c

rxn06246_c: cpd00001_c + cpd00528_c + cpd11609_c <=> cpd00659_c + cpd11610_c

rxn06254_c: cpd00001_c + 2.0 cpd00067_c + cpd11737_c <=> cpd11247_c

rxn06257_c: cpd12049_c <=> cpd00001_c + cpd00542_c

rxn06259_c: cpd00542_c <=> cpd12754_c

rxn06260_c: cpd00001_c <=> cpd12811_c

rxn06261_c: cpd00007_c + cpd03271_c + cpd11610_c <=> cpd00001_c + cpd00546_c + cpd11609_c

rxn06264_c: cpd00024_c + cpd00549_c <=> cpd02074_c + cpd11673_c

rxn06265_c: cpd00001_c + cpd11609_c + cpd14871_c <=> cpd00777_c + cpd11610_c

rxn06266_c: cpd00007_c + cpd00559_c + cpd11610_c <=> cpd00001_c + cpd00776_c + cpd11609_c

rxn06271_c: cpd00001_c --> cpd00158_c

rxn06272_c: cpd00001_c --> cpd00190_c

rxn06273_c: cpd00031_c --> cpd00315_c

rxn06275_c: cpd11750_c <=> cpd01430_c

rxn06279_c: cpd00017_c + cpd11615_c <=> cpd00019_c + cpd12571_c

rxn06280_c: cpd00002_c + cpd00069_c + cpd11751_c <=> cpd00012_c + cpd00018_c + cpd00067_c + cpd12194_c

rxn06283_c: cpd00610_c <=> cpd00067_c + cpd12155_c

rxn06286_c: cpd00612_c + cpd11609_c <=> cpd03330_c + cpd11610_c

rxn06291_c: cpd00001_c + cpd00629_c + cpd11609_c <=> cpd02334_c + cpd11610_c

rxn06294_c: cpd00001_c + cpd00735_c + cpd11609_c <=> cpd00011_c + cpd00643_c + cpd11610_c

rxn06300_c: cpd00002_c + cpd00035_c + cpd11906_c <=> cpd00012_c + cpd00018_c + cpd00067_c + cpd11770_c

rxn06303_c: cpd00001_c --> cpd00009_c

rxn06307_c: cpd00691_c <-- cpd00014_c

rxn06309_c: cpd00268_c + cpd00701_c <=> cpd00081_c + cpd00239_c + cpd02322_c

rxn06310_c: cpd00007_c + cpd00702_c + cpd11610_c <=> cpd00001_c + cpd02447_c + cpd11609_c

rxn06311_c: cpd00047_c + cpd00702_c <-- cpd12693_c

rxn06316_c: cpd00001_c + cpd11792_c <=> cpd11976_c

rxn06318_c: cpd00719_c <-- cpd00031_c

rxn06323_c: cpd00354_c + cpd11794_c <=> cpd00009_c + cpd00067_c + cpd11898_c

rxn06325_c: cpd00002_c + cpd11799_c <=> cpd00008_c + cpd00067_c + cpd12412_c

rxn06326_c: cpd00001_c + cpd12412_c <=> cpd00009_c + cpd11799_c

rxn06327_c: cpd00744_c + cpd11609_c <=> cpd00828_c + cpd11610_c

rxn06334_c: cpd00760_c + cpd11609_c <=> cpd02125_c + cpd11610_c

rxn06335_c: cpd00449_c + cpd00760_c <=> cpd00010_c + cpd00067_c + cpd14703_c

rxn06337_c: cpd00766_c + cpd11609_c <=> cpd00335_c + cpd11610_c

rxn06338_c: cpd00026_c + cpd00067_c + cpd11804_c <=> cpd00014_c + cpd12391_c

rxn06339_c: cpd00774_c + cpd11609_c <=> 6.0 cpd00067_c + cpd01624_c + cpd11610_c

rxn06342_c: cpd00007_c + cpd01645_c + cpd11610_c <=> cpd00001_c + cpd00778_c + cpd11609_c

rxn06343_c: cpd02066_c + cpd11609_c <=> cpd00784_c + cpd11610_c

rxn06345_c: cpd00007_c + cpd00024_c + cpd11811_c <=> cpd00011_c + cpd00036_c + cpd12539_c

rxn06346_c: cpd00007_c + cpd00024_c + cpd11811_c <=> cpd00011_c + cpd00036_c + cpd12540_c

rxn06350_c: cpd00820_c + cpd11609_c <=> cpd03853_c + cpd11610_c

rxn06352_c: cpd00827_c + cpd11609_c <=> 2.0 cpd00067_c + cpd01228_c + cpd11610_c

rxn06358_c: cpd00851_c + cpd11609_c <=> cpd02625_c + cpd11610_c

rxn06361_c: cpd00007_c + cpd00866_c + cpd11610_c <=> cpd00001_c + cpd03268_c + cpd11609_c

rxn06369_c: cpd00007_c + cpd11610_c + cpd12458_c <=> 2.0 cpd00001_c + cpd11609_c + cpd11825_c

rxn06374_c: cpd00001_c + cpd02822_c <=> cpd00908_c + cpd11616_c

rxn06380_c: cpd00007_c + cpd01182_c + cpd11610_c <=> cpd00917_c + cpd11609_c

rxn06395_c: cpd00007_c + cpd01401_c + cpd11610_c <=> cpd00001_c + cpd03477_c + cpd11609_c

rxn06404_c: cpd00001_c + cpd00067_c + cpd12213_c <=> cpd00013_c + cpd00020_c + cpd11849_c

rxn06410_c: cpd00022_c + cpd11866_c <=> cpd00010_c + cpd11990_c

rxn06411_c: cpd00044_c <-- cpd00045_c

rxn06414_c: cpd00001_c + cpd11875_c <=> cpd00018_c + cpd11794_c

rxn06415_c: cpd01053_c + cpd11610_c <=> cpd00001_c + cpd01054_c + cpd11609_c

rxn06419_c: cpd00001_c + cpd01078_c + cpd11609_c <=> cpd03387_c + cpd11610_c

rxn06420_c: cpd01608_c + cpd11610_c <=> cpd00035_c + cpd01078_c + cpd11609_c

rxn06421_c: cpd03389_c + cpd11610_c <=> cpd00035_c + cpd01078_c + cpd11609_c

rxn06422_c: cpd01078_c + cpd11621_c <=> cpd03387_c + cpd11620_c

rxn06431_c: cpd00001_c + cpd00823_c + cpd11904_c <=> cpd00228_c + cpd03477_c

rxn06432_c: cpd00002_c + cpd00051_c + cpd11907_c <=> cpd00012_c + cpd00018_c + cpd00067_c + cpd12036_c

rxn06433_c: cpd00002_c + cpd00041_c + cpd11908_c <=> cpd00012_c + cpd00018_c + cpd12829_c

rxn06434_c: cpd00002_c + cpd00132_c + cpd11908_c <=> cpd00012_c + cpd00018_c + cpd00067_c + cpd12313_c

rxn06435_c: cpd00002_c + cpd00084_c + cpd11910_c <=> cpd00012_c + cpd00018_c + cpd00067_c + cpd12255_c

rxn06436_c: cpd00002_c + cpd00023_c + cpd11911_c <=> cpd00012_c + cpd00018_c + cpd12828_c

rxn06437_c: cpd00002_c + cpd00053_c + cpd11911_c <=> cpd00012_c + cpd00018_c + cpd00067_c + cpd12060_c

rxn06438_c: cpd00002_c + cpd00033_c + cpd11913_c <=> cpd00012_c + cpd00018_c + cpd00067_c + cpd12100_c

rxn06439_c: cpd00002_c + cpd00119_c + cpd11914_c <=> cpd00012_c + cpd00018_c + cpd00067_c + cpd12228_c

rxn06440_c: cpd00002_c + cpd00322_c + cpd11915_c <=> cpd00012_c + cpd00018_c + cpd00067_c + cpd12256_c

rxn06441_c: cpd00002_c + cpd00107_c + cpd11916_c <=> cpd00012_c + cpd00018_c + cpd00067_c + cpd12003_c

rxn06442_c: cpd00002_c + cpd00039_c + cpd11917_c <=> cpd00012_c + cpd00018_c + cpd01326_c

rxn06443_c: cpd00002_c + cpd00060_c + cpd11918_c <=> cpd00012_c + cpd00018_c + cpd00067_c + cpd12105_c

rxn06444_c: cpd00002_c + cpd00066_c + cpd11919_c <=> cpd00012_c + cpd00018_c + cpd00067_c + cpd12335_c

rxn06445_c: cpd00002_c + cpd00129_c + cpd11920_c <=> cpd00012_c + cpd00018_c + 2.0 cpd00067_c + cpd12164_c

rxn06446_c: cpd00002_c + cpd00054_c + cpd11921_c <=> cpd00012_c + cpd00018_c + 2.0 cpd00067_c + cpd12132_c

rxn06447_c: cpd00002_c + cpd00161_c + cpd11922_c <=> cpd00012_c + cpd00018_c + 2.0 cpd00067_c + cpd12229_c

rxn06448_c: cpd00002_c + cpd00065_c + cpd11923_c <=> cpd00012_c + cpd00018_c + cpd00067_c + cpd12336_c

rxn06449_c: cpd00002_c + cpd00156_c + cpd11924_c <=> cpd00012_c + cpd00018_c + cpd00067_c + cpd12133_c

rxn06450_c: cpd01170_c + cpd11609_c <=> 2.0 cpd00067_c + cpd03223_c + cpd11610_c

rxn06457_c: cpd00001_c + cpd03766_c + cpd11609_c <=> cpd01182_c + cpd11610_c

rxn06460_c: cpd11949_c <=> cpd12721_c

rxn06462_c: cpd00001_c + cpd11950_c <=> cpd12349_c

rxn06463_c: cpd03254_c + cpd11609_c <=> cpd01228_c + cpd11610_c

rxn06464_c: cpd01243_c + cpd11609_c <=> cpd03037_c + cpd11610_c

rxn06466_c: cpd01259_c + cpd11610_c <=> cpd00067_c + cpd00534_c + cpd01621_c + cpd11609_c

rxn06471_c: cpd01291_c + cpd11609_c <=> cpd01884_c + cpd11610_c

rxn06475_c: cpd01297_c + cpd11707_c <=> cpd00010_c + cpd11423_c

rxn06477_c: cpd01306_c <=> cpd03221_c

rxn06482_c: cpd00007_c + cpd01342_c + cpd11610_c <=> cpd00001_c + cpd03058_c + cpd11609_c

rxn06483_c: cpd00007_c + cpd01342_c + cpd11610_c <=> cpd00001_c + cpd03266_c + cpd11609_c

rxn06484_c: cpd00017_c + cpd11981_c <=> cpd00019_c + cpd00067_c + cpd12632_c

rxn06486_c: cpd01358_c + cpd11609_c <=> cpd01447_c + cpd11610_c

rxn06491_c: cpd11463_c + cpd12003_c <=> cpd11615_c + cpd12104_c

rxn06492_c: cpd00007_c + cpd01398_c + cpd11610_c <=> 2.0 cpd00001_c + cpd01942_c + cpd11609_c

rxn06497_c: cpd01420_c + cpd11609_c <=> cpd03217_c + cpd11610_c

rxn06498_c: cpd03220_c + cpd11609_c <=> cpd00067_c + cpd01420_c + cpd11610_c

rxn06501_c: cpd01426_c + cpd11609_c <=> cpd03680_c + cpd11610_c

rxn06502_c: cpd01431_c + cpd11707_c <=> cpd00010_c + cpd11423_c

rxn06505_c: cpd00007_c + cpd03272_c + cpd11610_c <=> cpd00001_c + cpd01450_c + cpd11609_c

rxn06506_c: cpd03256_c + cpd11609_c <=> cpd01450_c + cpd11610_c

rxn06511_c: cpd11463_c + cpd12036_c <=> cpd11615_c + cpd11710_c

rxn06512_c: cpd00001_c + cpd12692_c --> cpd00073_c + cpd01468_c

rxn06514_c: cpd12288_c <=> cpd00040_c + cpd12041_c

rxn06519_c: cpd00115_c + cpd12058_c <=> cpd09118_c + cpd11461_c

rxn06520_c: cpd00008_c + cpd00009_c + cpd00023_c + cpd12060_c <=> cpd00002_c + cpd00053_c + cpd12828_c

rxn06529_c: cpd00113_c + cpd12460_c <=> cpd00012_c + cpd12077_c

rxn06533_c: cpd00083_c <-- cpd00126_c

rxn06534_c: cpd00644_c + cpd11609_c <=> cpd03534_c + cpd11610_c

rxn06535_c: cpd00163_c <-- cpd00014_c

rxn06536_c: cpd00007_c + cpd03275_c --> cpd01342_c + cpd01585_c

rxn06543_c: cpd00001_c + cpd12278_c <=> cpd00067_c + cpd11971_c + cpd12127_c

rxn06565_c: cpd00026_c + cpd12183_c <=> cpd00014_c + cpd12563_c

rxn06566_c: cpd00402_c <-- cpd00046_c

rxn06568_c: cpd00007_c + cpd00233_c + cpd12184_c <=> cpd00001_c + cpd00231_c + cpd12365_c

rxn06572_c: cpd00044_c + cpd12193_c <=> cpd00045_c + cpd12454_c

rxn06580_c: cpd01856_c + cpd11609_c <=> cpd01940_c + cpd11610_c

rxn06583_c: cpd00584_c <-- cpd00046_c

rxn06585_c: cpd01882_c + cpd11609_c <=> cpd01966_c + cpd11610_c

rxn06586_c: cpd00449_c + cpd01882_c <=> cpd00010_c + cpd00067_c + cpd14699_c

rxn06590_c: cpd11670_c <=> 3.0 cpd00067_c + cpd11678_c

rxn06591_c: cpd00005_c + cpd00067_c + cpd12227_c <=> cpd00006_c + cpd02345_c + cpd11912_c

rxn06593_c: cpd01927_c + cpd11609_c <=> cpd00011_c + cpd03328_c + cpd11610_c + cpd11640_c

rxn06609_c: cpd02043_c + cpd11609_c <=> cpd02259_c + cpd11610_c

rxn06612_c: cpd00003_c <-- cpd00067_c + cpd00133_c

rxn06613_c: cpd02083_c + cpd11609_c <=> 6.0 cpd00067_c + cpd03418_c + cpd11610_c

rxn06618_c: cpd00076_c <-- cpd00027_c

rxn06619_c: cpd00008_c + cpd00009_c + cpd00023_c + cpd12313_c <=> cpd00002_c + cpd00053_c + cpd12829_c

rxn06620_c: cpd12235_c + cpd12494_c <=> cpd11713_c + cpd13386_c

rxn06621_c: cpd00213_c + cpd00508_c <=> cpd00011_c + cpd14703_c

rxn06623_c: cpd11624_c + cpd11662_c <=> cpd02601_c + cpd11967_c

rxn06627_c: cpd00002_c + cpd00023_c <-- cpd00008_c + cpd00009_c

rxn06628_c: cpd06227_c <=> cpd00087_c + cpd12797_c

rxn06629_c: cpd02234_c <=> cpd12836_c

rxn06632_c: cpd00001_c + cpd12494_c <=> cpd00067_c + cpd11619_c + cpd12357_c

rxn06644_c: cpd11650_c + cpd12300_c <=> cpd12257_c + cpd12375_c

rxn06646_c: cpd02374_c + cpd11463_c <=> cpd00001_c + cpd12738_c

rxn06651_c: cpd00006_c + cpd11722_c <=> cpd00005_c + cpd00067_c + cpd12566_c

rxn06654_c: cpd00007_c + cpd02395_c + cpd11610_c <=> cpd00001_c + cpd02540_c + cpd11609_c

rxn06659_c: cpd00472_c <-- cpd00014_c

rxn06667_c: cpd00007_c + cpd02471_c + cpd11610_c <=> cpd00001_c + cpd02706_c + cpd11609_c

rxn06668_c: cpd00001_c + cpd12593_c <=> cpd02481_c + cpd11674_c

rxn06669_c: cpd00002_c + cpd00117_c + cpd12441_c <=> cpd00008_c + cpd00009_c + cpd00067_c + cpd12549_c

rxn06676_c: cpd00007_c + cpd02527_c + cpd11610_c <=> cpd00001_c + cpd02681_c + cpd11609_c

rxn06693_c: cpd00007_c + cpd11610_c + cpd13045_c <=> cpd00001_c + cpd11609_c + cpd12588_c

rxn06697_c: cpd00001_c + cpd02945_c <=> cpd02044_c + cpd12525_c

rxn06703_c: cpd02647_c + cpd11628_c <=> cpd11493_c + cpd12371_c

rxn06707_c: cpd00022_c + cpd12551_c <=> cpd00010_c + cpd12561_c

rxn06714_c: cpd12572_c <=> cpd12802_c

rxn06718_c: cpd03495_c <-- cpd02229_c

rxn06730_c: cpd00007_c + cpd11610_c + cpd12554_c <=> 2.0 cpd00001_c + cpd11609_c + cpd12606_c

rxn06740_c: cpd00112_c + cpd12605_c <=> cpd00046_c + cpd00067_c + cpd02982_c

rxn06746_c: cpd02961_c + cpd11609_c <=> cpd03522_c + cpd11610_c

rxn06747_c: cpd02965_c <=> cpd12833_c

rxn06762_c: cpd03037_c + cpd11610_c <=> cpd03222_c + cpd11609_c

rxn06767_c: cpd03086_c <-- cpd03087_c

rxn06779_c: cpd03138_c + cpd11609_c <=> cpd03141_c + cpd11610_c

rxn06781_c: cpd00002_c + 2.0 cpd00067_c + cpd03163_c + cpd11918_c <=> cpd00012_c + cpd00018_c + cpd12704_c

rxn06790_c: cpd00007_c + cpd03215_c + cpd11610_c <=> 2.0 cpd00001_c + cpd03216_c + cpd11609_c

rxn06791_c: cpd03215_c + cpd11609_c <=> cpd03217_c + cpd11610_c

rxn06792_c: cpd00007_c + cpd03216_c + cpd11610_c <=> 2.0 cpd00001_c + cpd03217_c + cpd11609_c

rxn06793_c: cpd00067_c + cpd03216_c + cpd11609_c <=> cpd03219_c + cpd11610_c

rxn06794_c: cpd03219_c + cpd11609_c <=> cpd03220_c + cpd11610_c + cpd11640_c

rxn06795_c: cpd03239_c + cpd11610_c <=> cpd03240_c + cpd11609_c

rxn06796_c: cpd03258_c + cpd11609_c <=> cpd03270_c + cpd11610_c

rxn06797_c: cpd00001_c + cpd03271_c + cpd11609_c <=> cpd00007_c + cpd03272_c + cpd11610_c

rxn06800_c: cpd00048_c + cpd12732_c <=> cpd00042_c + cpd03456_c

rxn06803_c: cpd03557_c + cpd11628_c <=> cpd11456_c + cpd11493_c

rxn06806_c: cpd03293_c + cpd11608_c <=> cpd12848_c

rxn06807_c: cpd03301_c <=> cpd03305_c

rxn06810_c: cpd00024_c + cpd03362_c <=> cpd03363_c + cpd11612_c

rxn06813_c: cpd12746_c <=> cpd12747_c

rxn06815_c: cpd03389_c + cpd11622_c <=> cpd00239_c + cpd03390_c

rxn06816_c: cpd03389_c <=> cpd03403_c

rxn06821_c: cpd11610_c + cpd11640_c + cpd12753_c <=> cpd11609_c + cpd12756_c

rxn06826_c: cpd03414_c + cpd11610_c <=> 6.0 cpd00067_c + cpd03415_c + cpd11609_c

rxn06827_c: cpd03416_c + cpd11609_c <=> 6.0 cpd00067_c + cpd03417_c + cpd11610_c

rxn06829_c: cpd03427_c + cpd11609_c <=> cpd03508_c + cpd11610_c

rxn06830_c: cpd12780_c <=> cpd12785_c

rxn06832_c: cpd00017_c + cpd11425_c <=> cpd00019_c + cpd12844_c

rxn06836_c: cpd03488_c + 5.0 cpd12100_c <=> 5.0 cpd00067_c + cpd03489_c + 5.0 cpd11913_c

rxn06837_c: cpd03492_c + 5.0 cpd12100_c <=> 5.0 cpd00067_c + cpd03493_c + 5.0 cpd11913_c

rxn06838_c: cpd03496_c + 5.0 cpd12100_c <=> 5.0 cpd00067_c + cpd03497_c + 5.0 cpd11913_c

rxn06839_c: cpd03427_c + cpd11609_c <=> 2.0 cpd00067_c + cpd03509_c + cpd11610_c

rxn06841_c: cpd03550_c + cpd11609_c <=> cpd03551_c + cpd11610_c

rxn06842_c: cpd03552_c + cpd11609_c <=> cpd03553_c + cpd11610_c

rxn06844_c: cpd00010_c + cpd03567_c <-- cpd00022_c + cpd03737_c

rxn06847_c: cpd00022_c + cpd12810_c <=> cpd00011_c + cpd12809_c

rxn06851_c: cpd03604_c + cpd11609_c <=> cpd03605_c + cpd11610_c

rxn06858_c: cpd00001_c + cpd12836_c <=> cpd00067_c + cpd02767_c + cpd12837_c

rxn06863_c: cpd03477_c + cpd12845_c <=> cpd02111_c + cpd12846_c

rxn06866_c: cpd00001_c + cpd03764_c + cpd11609_c <=> cpd03765_c + cpd11610_c

rxn06867_c: cpd00001_c + cpd03836_c + cpd11609_c <=> cpd03765_c + cpd11610_c

rxn06868_c: cpd12861_c + cpd12862_c <=> cpd00010_c + cpd12863_c

rxn06873_c: cpd00001_c + cpd03837_c + cpd11609_c <=> cpd03838_c + cpd11610_c

rxn06876_c: cpd00005_c + cpd00022_e + cpd00067_e + cpd00070_e <=> cpd00006_c + cpd00010_c + cpd00011_c + cpd00487_c

rxn06877_c: cpd00005_c + cpd01675_e + cpd11611_e <=> cpd00006_c + cpd00010_c + cpd00011_c + cpd12444_c

rxn06878_c: cpd00004_c + cpd00005_c + cpd00022_e + cpd00067_e + cpd00070_e <=> cpd00003_c + cpd00006_c + cpd00010_c + cpd00011_c + cpd12196_c

rxn06879_c: cpd00472_c <=> cpd00014_c + cpd11601_c

rxn06881_c: 5.0 cpd00027_c + cpd11735_c <=> cpd00001_c + 5.0 cpd00179_c

rxn06884_c: cpd03424_c + cpd03871_c <=> 3.0 cpd00067_c + cpd02024_c + cpd12878_c

rxn06885_c: cpd02024_c + cpd11610_c <=> cpd03870_c + cpd11609_c

rxn06891_c: 2.0 cpd00067_c + cpd04041_c + cpd11610_c <=> cpd04042_c + cpd11609_c

rxn06892_c: cpd00007_c + cpd02815_c + cpd11610_c <=> cpd04053_c + cpd11609_c

rxn06896_c: cpd01892_c + cpd11609_c <=> cpd00001_c + cpd00401_c + cpd11610_c

rxn06899_c: cpd00037_e + cpd00144_e <-- cpd00014_c + cpd11691_c

rxn06918_c: cpd00006_c + cpd04032_c <=> cpd00005_c + cpd00067_c + cpd04031_c

rxn06930_c: cpd00002_c + cpd00003_c + cpd00010_c + cpd00015_c + cpd00882_c --> cpd00004_c + cpd00012_c + cpd00018_c + cpd00022_c + cpd00067_c + cpd00599_c + cpd00982_c

rxn06931_c: cpd00011_c <-- cpd04863_c

rxn06936_c: cpd00002_c + cpd00041_c + cpd11909_c <=> cpd00012_c + cpd00018_c + cpd00067_c + cpd12226_c

rxn06937_c: cpd00002_c + cpd00023_c + cpd11912_c <=> cpd00012_c + cpd00018_c + cpd00067_c + cpd12227_c

rxn06938_c: cpd00413_c + cpd11609_c <=> cpd01599_c + cpd11610_c

rxn06939_c: cpd06716_c + cpd11609_c <=> cpd06717_c + cpd11610_c

rxn06941_c: cpd00113_c + cpd02557_c --> cpd00012_c + cpd02590_c

rxn06942_c: cpd00017_c + cpd03449_c <=> cpd00019_c + cpd11669_c

rxn06943_c: cpd02295_c + cpd02557_c <=> cpd00011_c + cpd00012_c + cpd11425_c

rxn06944_c: cpd06715_c + cpd11609_c <=> cpd06703_c + cpd11610_c

rxn06945_c: cpd00001_c --> cpd12840_c

rxn06946_c: cpd00127_c + cpd03729_c <=> cpd01773_c + cpd11855_c

rxn06949_c: cpd04322_c --> cpd00012_c + cpd04323_c

rxn06951_c: cpd00033_c + 2.0 cpd11609_c <=> cpd00011_c + cpd00150_c + 2.0 cpd11610_c

rxn06952_c: cpd00001_c + cpd02526_c + cpd11609_c <=> cpd00128_c + cpd04535_c + cpd11610_c

rxn06955_c: cpd00007_c + cpd01122_c + cpd11610_c <=> cpd00001_c + cpd04503_c + cpd11609_c

rxn06957_c: cpd00007_c + cpd04550_c + cpd11610_c <=> cpd00001_c + cpd04551_c + cpd11609_c

rxn06960_c: cpd00001_c + cpd04383_c + cpd11609_c <=> cpd08204_c + cpd11610_c

rxn06961_c: cpd01048_c + cpd13049_c <=> cpd04098_c + cpd13050_c

rxn06962_c: 2.0 cpd00042_c + cpd04505_c <=> cpd00111_c + cpd04506_c

rxn06964_c: cpd04098_c + cpd11609_c <=> cpd01048_c + cpd11610_c

rxn06965_c: cpd00099_c + cpd04167_c + cpd11609_c <=> cpd04166_c + cpd11610_c

rxn06973_c: cpd00001_c <=> cpd13379_c

rxn06974_c: cpd00001_c <=> cpd13381_c

rxn06988_c: cpd06139_c <=> cpd08488_c

rxn06997_c: cpd00001_c --> cpd00029_c

rxn06998_c: cpd00134_c --> cpd00010_c

rxn06999_c: cpd11456_c <=> cpd11423_c

rxn07000_c: cpd00001_c --> cpd00724_c

rxn07001_c: cpd00001_c --> cpd00832_c

rxn07002_c: cpd00037_c + cpd11619_c <=> cpd00091_c

rxn07003_c: cpd12235_c <=> cpd11713_c

rxn07005_c: 4.0 cpd00001_c --> 4.0 cpd00138_c

rxn07006_c: 2.0 cpd00001_c --> 2.0 cpd00138_c

rxn07009_c: cpd00232_c <-- cpd00001_c

rxn07011_c: cpd00001_c --> cpd01112_c

rxn07013_c: cpd00009_c --> cpd00082_c + cpd00089_c

rxn07015_c: cpd00009_c --> cpd00027_c + cpd00501_c

rxn07018_c: cpd00009_c --> cpd00079_c + cpd00501_c

rxn07023_c: cpd00009_c --> cpd00027_c + cpd00089_c

rxn07024_c: cpd00009_c --> cpd00122_c + cpd00348_c

rxn07026_c: 2.0 cpd00089_c <-- 2.0 cpd00009_c

rxn07031_c: cpd02560_c <-- cpd01904_c

rxn07034_c: cpd00001_c --> 2.0 cpd00027_c

rxn07037_c: cpd00001_c --> cpd00027_c + cpd00082_c

rxn07039_c: cpd00001_c --> cpd00027_c + cpd00108_c

rxn07040_c: cpd00001_c --> 2.0 cpd00190_c

rxn07041_c: cpd00001_c --> cpd00108_c + cpd00121_c

rxn07043_c: cpd00001_c --> cpd00709_c

rxn07045_c: cpd00001_c --> cpd00079_c + cpd00082_c

rxn07047_c: cpd00001_c --> cpd00190_c + cpd00818_c

rxn07049_c: cpd00001_c --> cpd00027_c + cpd00079_c

rxn07051_c: cpd00001_c --> cpd00751_c + cpd02274_c

rxn07054_c: cpd03077_c <=> cpd00013_c + cpd11625_c

rxn07055_c: cpd00001_c + cpd00007_c + cpd11731_c <=> cpd00013_c + cpd00025_c + cpd11616_c

rxn07060_c: cpd00001_c --> cpd00054_c

rxn07061_c: cpd00001_c --> 2.0 cpd00122_c

rxn07062_c: cpd00001_c --> cpd00108_c + cpd00138_c

rxn07063_c: cpd00001_c --> cpd00164_c + cpd02372_c

rxn07064_c: cpd00001_c --> cpd00190_c + cpd03193_c

rxn07066_c: cpd00001_c <=> cpd00167_c

rxn07070_c: cpd03491_c --> cpd02229_c

rxn07075_c: cpd12508_c <=> cpd11712_c

rxn07078_c: cpd00001_c + cpd00002_c <-- cpd00009_c

rxn07086_c: cpd11609_c <=> cpd11610_c

rxn07088_c: cpd00001_c --> cpd00280_c + cpd02508_c

rxn07089_c: cpd00001_c <-- cpd00116_c

rxn07090_c: cpd00022_c <-- cpd00010_c

rxn07093_c: cpd00001_c --> cpd00048_c

rxn07094_c: cpd02546_c <-- cpd00046_c

rxn07095_c: 2.0 cpd00003_c <-- cpd00004_c + cpd00067_c

rxn07097_c: cpd00007_c + cpd08692_c + cpd11610_c <=> cpd00001_c + cpd08693_c + cpd11609_c

rxn07099_c: cpd00001_c + cpd11668_c <=> cpd00067_c + cpd12884_c

rxn07103_c: cpd08731_c + cpd11609_c <=> cpd08732_c + cpd11610_c

rxn07104_c: cpd01220_c + cpd11610_c <=> cpd08252_c + cpd11609_c

rxn07105_c: cpd03749_c + cpd11610_c <=> cpd00001_c + cpd08735_c + cpd11609_c

rxn07106_c: cpd00007_c + cpd08735_c + cpd11609_c <=> 2.0 cpd00001_c + cpd08736_c + cpd11610_c

rxn07107_c: cpd00001_c + cpd08736_c + cpd11609_c <=> cpd08737_c + cpd11610_c

rxn07109_c: cpd00007_c + cpd08908_c + cpd11610_c <=> 2.0 cpd00001_c + cpd00167_c + cpd11609_c

rxn07114_c: cpd08920_c <-- cpd08922_c

rxn07117_c: cpd00582_c <-- cpd07389_c

rxn07119_c: cpd01927_c + cpd11609_c <=> cpd00011_c + cpd02667_c + cpd11610_c + cpd11640_c

rxn07121_c: cpd01651_c <-- cpd03377_c

rxn07123_c: cpd00001_c <=> cpd11422_c

rxn07124_c: cpd00868_c --> cpd00011_c + cpd02043_c

rxn07125_c: cpd00022_c + 7.0 cpd00070_c <=> 7.0 cpd00010_c + 7.0 cpd00011_c + cpd13411_c

rxn07126_c: 7.0 cpd00070_c + cpd00086_c <=> 7.0 cpd00010_c + 7.0 cpd00011_c + cpd13412_c

rxn07127_c: 7.0 cpd00070_c + cpd00120_c <=> 7.0 cpd00010_c + 7.0 cpd00011_c + cpd13413_c

rxn07131_c: 8.0 cpd00070_c + cpd04029_c <=> 8.0 cpd00010_c + 8.0 cpd00011_c + cpd13430_c

rxn07133_c: cpd00022_c + 9.0 cpd00070_c <=> 9.0 cpd00010_c + 9.0 cpd00011_c + cpd13417_c

rxn07134_c: 9.0 cpd00070_c + cpd00086_c <=> 9.0 cpd00010_c + 9.0 cpd00011_c + cpd13418_c

rxn07135_c: 9.0 cpd00070_c + cpd00120_c <=> 9.0 cpd00010_c + 9.0 cpd00011_c + cpd13419_c

rxn07144_c: cpd09119_c <-- cpd09125_c

rxn07145_c: cpd09120_c <-- cpd09127_c

rxn07146_c: cpd09121_c <-- cpd09129_c

rxn07147_c: cpd09122_c <-- cpd09130_c

rxn07150_c: cpd00002_c + cpd00129_c <=> cpd00012_c + cpd00018_c + cpd13438_c

rxn07154_c: cpd00002_c + cpd00069_c <=> cpd00012_c + cpd00018_c + cpd13432_c

rxn07158_c: cpd13435_c <=> cpd13436_c

rxn07159_c: cpd13436_c <=> cpd09211_c

rxn07161_c: cpd13433_c <=> cpd13431_c

rxn07162_c: cpd13431_c <=> cpd09210_c

rxn07163_c: cpd13434_c <=> cpd13437_c

rxn07164_c: cpd13437_c <=> cpd09212_c

rxn07165_c: cpd09212_c --> cpd09213_c

rxn07166_c: cpd09223_c + 2.0 cpd13441_c <=> cpd03029_c

rxn07167_c: cpd09216_c + cpd13441_c <=> cpd08823_c

rxn07170_c: cpd04367_c <-- cpd04309_c

rxn07171_c: cpd09315_c --> cpd00036_c + cpd04135_c

rxn07175_c: cpd09817_c --> cpd09814_c

rxn07176_c: cpd03218_c --> cpd05508_c

rxn07179_c: cpd00007_c + cpd01122_c + cpd11610_c <=> 2.0 cpd00001_c + cpd03849_c + cpd11609_c

rxn07180_c: cpd00001_c + cpd11624_c <=> cpd01122_c + cpd02601_c

rxn07182_c: cpd00001_c + cpd10556_c <-- cpd04135_c

rxn07183_c: cpd00001_c + cpd10562_c <-- cpd10559_c

rxn07186_c: cpd00480_c + cpd11609_c <=> cpd01793_c + cpd11610_c

rxn07187_c: cpd00263_c + cpd11609_c <=> cpd03604_c + cpd11610_c

rxn07188_c: cpd11609_c + cpd14473_c <=> cpd11610_c + cpd11625_c

rxn07194_c: cpd00006_c + cpd11611_c <=> cpd00005_c + cpd00067_c + cpd11722_c

rxn07195_c: cpd11176_c + cpd11609_c <=> cpd00365_c + cpd11610_c

rxn07196_c: cpd00001_c + cpd11609_c + cpd11673_c <=> cpd00013_c + cpd11610_c + cpd11625_c

rxn07197_c: cpd00001_c + cpd00210_c + cpd11609_c <=> cpd00013_c + cpd00460_c + cpd11610_c

rxn07198_c: cpd03177_c + cpd11609_c <=> cpd00528_c + cpd11610_c

rxn07201_c: cpd11609_c + cpd11640_c <=> cpd11610_c

rxn07205_c: cpd00007_c + cpd00160_c + cpd11610_c <=> cpd00001_c + cpd11199_c + cpd11609_c

rxn07206_c: cpd00001_c + cpd01042_c + cpd11609_c <=> cpd00484_c + cpd11610_c

rxn07207_c: cpd00013_c + cpd00196_c + cpd11420_c <=> cpd00009_c + cpd00033_c + cpd11421_c

rxn07208_c: cpd00187_c + cpd00196_c + cpd11420_c <=> cpd00009_c + cpd00183_c + cpd11421_c

rxn07209_c: cpd00196_c + cpd00441_c + cpd11420_c <=> cpd00009_c + cpd00540_c + cpd11421_c

rxn07210_c: cpd00001_c + cpd03387_c + cpd11609_c <=> cpd03396_c + cpd11610_c

rxn07212_c: cpd00017_c + cpd11635_c <=> cpd00019_c + cpd12480_c

rxn07213_c: cpd00017_c + cpd11635_c <=> cpd00019_c + cpd12478_c

rxn07214_c: cpd00017_c + cpd11635_c <=> cpd00019_c + cpd12479_c

rxn07216_c: cpd00022_c + cpd12551_c <=> cpd00010_c + cpd13333_c

rxn07220_c: cpd00067_c + cpd00070_c + cpd12430_c <=> cpd00010_c + cpd14483_c

rxn07224_c: cpd14484_c <=> cpd00354_c

rxn07226_c: cpd00241_c + cpd12058_c <=> cpd09118_c + cpd11461_c

rxn07227_c: cpd00009_c <=> cpd00354_c

rxn07228_c: cpd00044_c + 2.0 cpd00067_c + cpd11678_c <=> cpd00045_c + cpd14488_c

rxn07234_c: cpd11718_c <=> cpd00001_c + cpd12657_c

rxn07236_c: cpd00003_c + cpd00057_c <=> cpd00004_c + cpd00067_c + cpd11616_c

rxn07237_c: cpd00003_c + cpd00057_c <=> cpd00004_c + cpd00067_c + cpd11733_c

rxn07238_c: cpd00006_c + cpd00057_c <=> cpd00005_c + cpd00067_c + cpd11616_c

rxn07253_c: cpd00001_c + cpd11262_c + cpd11609_c <=> cpd11263_c + cpd11610_c

rxn07275_c: cpd00007_c + cpd11610_c + cpd14526_c <=> cpd00001_c + cpd11609_c + cpd14527_c

rxn07299_c: cpd00007_c + cpd11610_c + cpd14523_c <=> cpd00001_c + cpd11609_c + cpd14524_c

rxn07302_c: cpd00007_c + cpd11610_c + cpd14529_c <=> cpd00001_c + cpd11609_c + cpd14530_c

rxn07303_c: cpd14530_c --> cpd05719_c

rxn07313_c: cpd05727_c <=> cpd01451_c

rxn07316_c: cpd08360_c <=> cpd14518_c

rxn07321_c: cpd08359_c <=> cpd14514_c

rxn07327_c: cpd03035_c --> cpd03221_c

rxn07339_c: cpd09851_c <-- cpd01628_c

rxn07340_c: cpd00005_c + cpd00011_c + cpd00067_c + cpd03038_c --> cpd00001_c + cpd00006_c + cpd14544_c

rxn07361_c: cpd03218_c --> cpd05497_c

rxn07362_c: cpd05497_c --> cpd05508_c

rxn07364_c: cpd14623_c <-- cpd14626_c

rxn07375_c: cpd14640_c --> cpd14643_c

rxn07376_c: cpd14643_c <=> cpd14646_c

rxn07377_c: cpd14590_c --> cpd14592_c

rxn07378_c: cpd14592_c <=> cpd14595_c

rxn07379_c: cpd03220_c --> cpd14648_c

rxn07380_c: cpd14648_c --> cpd14657_c

rxn07381_c: cpd14648_c --> cpd14661_c

rxn07383_c: cpd03220_c --> cpd14662_c

rxn07384_c: cpd14662_c <-- cpd14663_c

rxn07385_c: cpd14663_c --> cpd14665_c

rxn07386_c: cpd03220_c --> cpd14667_c

rxn07387_c: cpd14667_c --> cpd14668_c

rxn07388_c: cpd14668_c --> cpd14669_c

rxn07389_c: cpd01420_c --> cpd05498_c

rxn07390_c: cpd05498_c --> cpd03637_c

rxn07392_c: cpd05499_c --> cpd14689_c

rxn07394_c: cpd05499_c --> cpd05490_c

rxn07395_c: cpd05498_c --> cpd14689_c

rxn07397_c: cpd14689_c --> cpd14691_c

rxn07399_c: cpd05490_c --> cpd14691_c

rxn07400_c: cpd14689_c --> cpd14692_c

rxn07402_c: cpd03637_c --> cpd14692_c

rxn07403_c: cpd14691_c --> cpd05487_c

rxn07404_c: cpd14692_c --> cpd05487_c

rxn07409_c: cpd05498_c <=> cpd14706_c

rxn07410_c: cpd14706_c <=> cpd14707_c

rxn07411_c: cpd03637_c <=> cpd14707_c

rxn07412_c: cpd14707_c <=> cpd14708_c

rxn07416_c: cpd05498_c <-- cpd14677_c

rxn07417_c: cpd05498_c <-- cpd14678_c

rxn07418_c: cpd05498_c <-- cpd14679_c

rxn07419_c: cpd14677_c <=> cpd14680_c

rxn07420_c: cpd14678_c <=> cpd14681_c

rxn07421_c: cpd14679_c <=> cpd14682_c

rxn07422_c: cpd03637_c <-- cpd14680_c

rxn07423_c: cpd03637_c <-- cpd14681_c

rxn07424_c: cpd03637_c <-- cpd14682_c

rxn07425_c: cpd14680_c <-- cpd14683_c

rxn07426_c: cpd14681_c <-- cpd14687_c

rxn07427_c: cpd14682_c <-- cpd14688_c

rxn07433_c: cpd00213_c + cpd14698_c <=> cpd00056_c + cpd00067_c + cpd14699_c

rxn07435_c: cpd00213_c + cpd14702_c <=> cpd00056_c + cpd00067_c + cpd14703_c

rxn07436_c: 2.0 cpd00006_c + cpd01029_c <=> 2.0 cpd00005_c + 3.0 cpd00067_c + cpd14718_c

rxn07467_c: cpd00350_c <-- cpd14865_c

rxn07469_c: cpd14866_c --> cpd14867_c

rxn07470_c: cpd14867_c --> cpd14868_c

rxn07471_c: cpd14868_c <-- cpd14869_c

rxn07499_c: cpd00001_c + cpd14917_c --> cpd00020_c + cpd14918_c

rxn07504_c: cpd14933_c --> cpd14934_c

rxn07507_c: cpd14929_c <-- cpd14930_c

rxn07508_c: cpd14923_c <-- cpd14927_c

rxn07509_c: cpd14932_c --> cpd00599_c

rxn07510_c: cpd14931_c <-- cpd14932_c

rxn07511_c: cpd00007_c + cpd14919_c <=> cpd00001_c + cpd00127_c

rxn07531_c: cpd10013_c <-- cpd10235_c

rxn07536_c: cpd11233_c <-- cpd14909_c

rxn07540_c: cpd07089_c --> cpd07094_c

rxn07546_c: cpd14941_c <-- cpd07395_c

rxn07547_c: cpd07395_c <-- cpd00639_c

rxn07549_c: cpd01868_c <=> cpd14907_c

rxn07556_c: cpd14943_c <-- cpd14942_c

rxn07557_c: cpd14942_c <-- cpd07389_c

rxn07558_c: cpd01100_c <=> cpd04758_c

rxn07560_c: cpd00005_c + cpd00007_c + cpd00067_c + cpd07408_c --> cpd00001_c + cpd00006_c + cpd14906_c

rxn07561_c: cpd00005_c + cpd00067_c + cpd14906_c --> cpd00006_c + cpd14944_c

rxn07563_c: cpd14946_c --> cpd14947_c

rxn07564_c: cpd14947_c <-- cpd07402_c

rxn07565_c: cpd00005_c + cpd00067_c + cpd14906_c --> cpd00006_c + cpd14945_c

rxn07569_c: cpd14948_c <-- cpd07424_c

rxn07570_c: cpd07424_c <-- cpd07388_c

rxn07577_c: cpd00005_c + cpd00067_c + cpd14938_c <=> cpd00006_c + cpd14939_c

rxn07578_c: cpd14939_c <=> cpd00001_c + cpd14940_c

rxn07579_c: cpd00004_c + cpd00067_c + cpd14940_c <=> cpd00003_c + cpd12458_c

rxn07582_c: 2.0 cpd00017_c + 2.0 cpd00074_c + cpd11470_c <=> 2.0 cpd00060_c + 2.0 cpd03091_c + cpd14956_c

rxn07583_c: cpd14956_c + cpd14957_c <=> cpd00067_c + cpd11493_c + cpd14954_c

rxn07588_c: cpd00017_c + cpd08373_c <=> cpd00019_c + cpd00067_c + cpd14961_c

rxn07589_c: cpd00017_c + cpd14961_c <=> cpd00011_c + cpd00019_c + cpd08375_c

rxn07597_c: cpd02440_c <-- cpd14963_c

rxn07598_c: cpd14963_c --> cpd14964_c

rxn07599_c: cpd14964_c <=> cpd14965_c

rxn07610_c: cpd04863_c --> cpd00077_c

rxn07613_c: cpd00001_c --> cpd03888_c

rxn07614_c: cpd00001_c --> cpd00164_c

rxn07616_c: cpd00001_c --> cpd14981_c

rxn07617_c: cpd00604_c --> cpd00881_c

rxn07625_c: cpd14601_c --> cpd14986_c

rxn07629_c: cpd15001_c <=> cpd06706_c

rxn07632_c: cpd03637_c --> cpd14998_c

rxn07633_c: cpd14998_c --> cpd15000_c

rxn07634_c: cpd14986_c --> cpd14987_c

rxn07635_c: cpd03217_c --> cpd14990_c

rxn07636_c: cpd14990_c --> cpd14991_c

rxn07637_c: cpd03217_c --> cpd14993_c

rxn07638_c: cpd14993_c --> cpd14994_c

rxn07643_c: cpd03220_c --> cpd14663_c

rxn07649_c: cpd00001_c + cpd11624_c <=> cpd02601_c + cpd03850_c

rxn07650_c: cpd00001_c + cpd11624_c <=> cpd02603_c + cpd03850_c

rxn07651_c: cpd00007_c + cpd03850_c + cpd11610_c <=> 2.0 cpd00001_c + cpd11609_c + cpd15016_c

rxn07652_c: cpd14591_c --> cpd15063_c

rxn07690_c: cpd05527_c --> cpd15008_c

rxn07691_c: cpd15008_c <-- cpd15009_c

rxn07694_c: cpd15031_c --> cpd15010_c

rxn07695_c: cpd15010_c <-- cpd15011_c

rxn07696_c: cpd15011_c <-- cpd15012_c

rxn07697_c: cpd15010_c <-- cpd15012_c

rxn07698_c: cpd03502_c <=> cpd15014_c

rxn07699_c: cpd15014_c --> cpd05546_c

rxn07705_c: cpd15019_c --> cpd15018_c

rxn07706_c: cpd00289_c <-- cpd03205_c

rxn07714_c: cpd15070_c --> cpd05548_c

rxn07715_c: cpd15030_c --> cpd05548_c

rxn07723_c: cpd00007_c + cpd11610_c + cpd14883_c <=> 2.0 cpd00001_c + cpd11609_c + cpd14884_c

rxn07730_c: cpd09576_c --> cpd15081_c

rxn07731_c: cpd15081_c <-- cpd15082_c

rxn07733_c: cpd09576_c --> cpd15074_c

rxn07735_c: cpd09576_c --> cpd15072_c

rxn07736_c: cpd00001_c + cpd11609_c + cpd14871_c <=> cpd01946_c + cpd11610_c

rxn07738_c: cpd00007_c + cpd02185_c --> cpd14873_c

rxn07772_c: cpd15078_c --> cpd00040_c + cpd15079_c + cpd15080_c

rxn07784_c: cpd05556_c --> cpd05485_c

rxn07794_c: cpd15122_c <-- cpd15123_c

rxn07796_c: cpd03969_c --> cpd15113_c

rxn07797_c: cpd11204_c --> cpd15114_c

rxn07801_c: cpd05483_c --> cpd05484_c

rxn07802_c: cpd03344_c <-- cpd06648_c

rxn07810_c: cpd15098_c <=> cpd15119_c

rxn07813_c: cpd00604_c --> cpd15129_c

rxn07815_c: cpd06681_c --> cpd06698_c

rxn07816_c: cpd00398_c --> cpd01176_c

rxn07817_c: cpd15102_c <=> cpd15106_c

rxn07818_c: cpd06718_c --> cpd15124_c

rxn07819_c: cpd06719_c <-- cpd15125_c

rxn07820_c: cpd15103_c <=> cpd15106_c

rxn07821_c: cpd11204_c <-- cpd15111_c

rxn07823_c: cpd15101_c <=> cpd15104_c

rxn07824_c: cpd15102_c <=> cpd15105_c

rxn07825_c: cpd15105_c <-- cpd15106_c

rxn07827_c: cpd15099_c <=> cpd15118_c

rxn07828_c: cpd15099_c <=> cpd15117_c

rxn07829_c: cpd15098_c <=> cpd15118_c

rxn07830_c: cpd15106_c --> cpd15107_c

rxn07831_c: cpd15107_c <-- cpd01042_c

rxn07839_c: cpd15109_c <=> cpd15102_c

rxn07866_c: cpd12546_c <=> cpd15171_c

rxn07867_c: cpd15171_c <=> cpd15145_c

rxn07878_c: cpd11609_c + cpd15168_c <=> cpd01317_c + cpd11610_c

rxn07891_c: cpd05487_c <=> cpd15190_c

rxn07910_c: cpd15186_c <=> cpd00077_c

rxn07911_c: cpd15185_c <=> cpd15176_c

rxn08024_c: cpd11735_c <=> cpd01329_c

rxn08076_c: cpd00001_c + cpd15398_c <=> cpd15387_c + cpd15396_c

rxn08077_c: cpd00001_c + cpd15398_c <=> cpd00117_c + cpd15397_c

rxn08078_c: cpd00001_c + 2.0 cpd00067_c + cpd15398_c <=> cpd00122_c + cpd15401_c

rxn08144_c: cpd10516_c + cpd15411_c <=> cpd03294_c

rxn08145_c: cpd00982_c + 2.0 cpd03294_c <=> cpd00015_c + 2.0 cpd00067_c + 2.0 cpd10515_c + 2.0 cpd15411_c

rxn08146_c: cpd01270_c + 2.0 cpd03294_c <=> cpd00050_c + 2.0 cpd00067_c + 2.0 cpd10515_c + 2.0 cpd15411_c

rxn08147_c: cpd00739_c + 2.0 cpd03294_c <=> 2.0 cpd00067_c + cpd00220_c + 2.0 cpd10515_c + 2.0 cpd15411_c

rxn08257_c: cpd00266_c + cpd15441_c <-- cpd08305_c + cpd15440_c

rxn08269_c: cpd00002_c + cpd00010_c + cpd08305_c --> cpd00008_c + cpd00009_c + cpd15441_c

rxn08407_c: 4.0 cpd00067_c + cpd15432_c + cpd15458_c <-- cpd02229_c + cpd15457_c

rxn08480_c: cpd15460_c <=> cpd10516_c + cpd15332_c

rxn08484_c: cpd10516_c + cpd15461_c <=> cpd12843_c

rxn08485_c: cpd00982_c + 2.0 cpd12843_c <=> cpd00015_c + 2.0 cpd00067_c + 2.0 cpd10515_c + 2.0 cpd15461_c

rxn08486_c: cpd01270_c + 2.0 cpd12843_c <=> cpd00050_c + 2.0 cpd00067_c + 2.0 cpd10515_c + 2.0 cpd15461_c

rxn08487_c: cpd00739_c + 2.0 cpd12843_c <=> 2.0 cpd00067_c + cpd00220_c + 2.0 cpd10515_c + 2.0 cpd15461_c

rxn08494_c: cpd10516_c + cpd15462_c <=> 5.0 cpd00067_c + cpd03724_c

rxn08495_c: 7.0 cpd00067_c + cpd00982_c + 2.0 cpd03724_c <=> cpd00015_c + 2.0 cpd10515_c + 2.0 cpd15462_c

rxn08496_c: 7.0 cpd00067_c + cpd01270_c + 2.0 cpd03724_c <=> cpd00050_c + 2.0 cpd10515_c + 2.0 cpd15462_c

rxn08497_c: 7.0 cpd00067_c + cpd00739_c + 2.0 cpd03724_c <=> cpd00220_c + 2.0 cpd10515_c + 2.0 cpd15462_c

rxn08600_c: <=> cpd00031_c

rxn08605_c: cpd00155_c <=> cpd15413_c

rxn08611_c: cpd00009_c + cpd15413_c <=> cpd00089_c

rxn08615_c: cpd00387_c + cpd15302_c <=> cpd00008_c + cpd00155_c

rxn08680_c: <=> cpd00038_c

rxn08772_c: cpd15432_c + cpd15576_c <=> 2.0 cpd00067_c + cpd00286_c + cpd15392_c

rxn08790_c: 3.0 cpd00067_c + cpd00214_c + cpd03587_c <-- cpd00001_c + cpd15483_c

rxn08808_c: cpd00001_c + cpd00067_c + cpd12547_c <=> cpd00908_c + cpd01080_c

rxn08809_c: cpd00001_c + cpd12547_c <=> cpd00067_c + cpd00908_c + cpd15269_c

rxn08887_c: cpd15511_c <=> cpd00117_c + cpd15514_c

rxn08888_c: cpd15511_c <=> cpd00731_c + cpd15513_c

rxn08889_c: cpd15512_c <=> 2.0 cpd00117_c + cpd15515_c

rxn08895_c: cpd00001_c + cpd15506_c <=> cpd15505_c

rxn08896_c: cpd00001_c + cpd15503_c <=> cpd15504_c

rxn08897_c: cpd00001_c + cpd15514_c <=> cpd15510_c

rxn08931_c: cpd00001_c + cpd15502_c <=> cpd15501_c

rxn08932_c: cpd00001_c + cpd15513_c <=> cpd15509_c

rxn08937_c: cpd15505_c <=> 2.0 cpd15398_c

rxn08938_c: cpd15504_c <=> cpd15397_c + cpd15398_c

rxn08939_c: cpd15501_c <=> 2.0 cpd15397_c

rxn08940_c: 2.0 cpd00067_c + cpd15507_c <=> cpd15398_c + cpd15506_c

rxn08953_c: cpd02546_c + cpd03002_c --> cpd00046_c + cpd00067_c + cpd03585_c

rxn09021_c: cpd15432_c + cpd15519_c <-- cpd02229_c + cpd15518_c

rxn09156_c: cpd00001_c + cpd15533_c <=> 3.0 cpd00067_c + cpd01080_c + cpd12547_c

rxn09157_c: cpd00001_c + cpd15534_c <=> cpd00067_c + cpd12547_c + cpd15269_c

rxn09249_c: cpd00002_c + cpd00054_c + cpd15573_c <=> cpd00012_c + cpd00018_c + cpd15565_c

rxn09356_c: cpd00201_c <-- cpd00067_c + cpd00087_c

rxn09364_c: cpd00286_c + cpd15577_c <=> cpd00014_c + cpd15575_c

rxn09397_c: cpd00026_c <=> cpd00014_c + cpd11735_c

rxn09398_c: cpd00026_c + cpd15302_c <=> cpd00014_c + cpd00155_c

rxn09399_c: cpd11735_c <=> cpd00155_c

rxn09400_c: cpd00026_c <=> cpd00014_c + cpd11791_c

rxn09401_c: cpd00001_c + cpd11683_c <=> cpd00029_c + cpd00067_c + cpd11741_c

rxn09490_c: cpd00175_c <=> cpd00014_c + cpd11683_c

rxn09491_c: 0.27 cpd00134_c + 0.05 cpd00327_c + 0.06 cpd01260_c + 0.1 cpd01695_c + 0.02 cpd03128_c + 0.01 cpd15192_c + 0.17 cpd15238_c + 0.09 cpd15272_c + 0.24 cpd15274_c <=> cpd00010_c + 0.01 cpd11677_c

rxn09507_c: cpd00083_c + cpd12789_c <=> cpd00031_c + cpd15250_c

rxn09508_c: cpd00083_c + cpd15252_c <=> cpd00031_c + cpd12791_c

rxn09511_c: cpd12407_c <=> cpd00067_c + cpd11619_c + cpd11685_c

rxn09535_c: cpd00017_c + 0.01 cpd12514_c <=> cpd00019_c + 0.01 cpd11624_c

rxn09536_c: cpd00121_c + 0.01 cpd11427_c <=> cpd00046_c + 0.01 cpd11822_c

rxn09537_c: cpd00002_c + 0.01 cpd11822_c <=> cpd00008_c + 0.01 cpd15288_c

rxn09538_c: cpd00002_c + 0.01 cpd11822_c <=> cpd00008_c + 0.01 cpd15287_c

rxn09539_c: cpd00002_c + 0.01 cpd15288_c <=> cpd00008_c + 0.01 cpd12582_c

rxn09540_c: cpd00001_c + 0.01 cpd12582_c <=> cpd00916_c + 0.01 cpd15192_c

rxn09541_c: cpd00002_c + 0.01 cpd15287_c <=> cpd00008_c + cpd00067_c + 0.01 cpd15284_c

rxn09542_c: cpd00001_c + 0.01 cpd11454_c <=> cpd00009_c + 0.01 cpd11652_c

rxn09543_c: cpd00017_c + 0.01 cpd11456_c <=> cpd00019_c + 0.01 cpd11829_c

rxn09544_c: cpd00002_c + 0.01 cpd15276_c <=> cpd00008_c + 0.01 cpd15229_c

rxn09545_c: cpd00017_c + 0.01 cpd11829_c <=> cpd00019_c + 0.01 cpd12514_c

rxn09546_c: cpd00001_c + 0.01 cpd15229_c <=> cpd00009_c + cpd00067_c + 0.01 cpd15276_c

rxn09547_c: cpd00095_c + 0.27 cpd00134_c + 0.05 cpd00327_c + 0.06 cpd01260_c + 0.1 cpd01695_c + 0.02 cpd03128_c + 0.17 cpd15238_c + 0.09 cpd15272_c + 0.24 cpd15274_c <=> cpd00010_c + 0.01 cpd15193_c

rxn09548_c: cpd00080_c + 0.27 cpd00134_c + 0.05 cpd00327_c + 0.06 cpd01260_c + 0.1 cpd01695_c + 0.02 cpd03128_c + 0.17 cpd15238_c + 0.09 cpd15272_c + 0.24 cpd15274_c <=> cpd00010_c + 0.01 cpd00517_c

rxn09549_c: cpd00444_c + 0.01 cpd15192_c <=> cpd00046_c + 0.01 cpd11456_c

rxn09550_c: cpd00052_c + 0.01 cpd15276_c <=> cpd00012_c + 0.01 cpd11427_c

rxn09551_c: cpd00001_c + 0.01 cpd15276_c <=> cpd00009_c + 0.01 cpd15192_c

rxn09552_c: cpd00256_c + 0.01 cpd15192_c <=> cpd00046_c + 0.01 cpd11624_c

rxn09553_c: 0.01 cpd11427_c + 0.01 cpd11652_c <=> cpd00046_c + 0.01 cpd12801_c

rxn09555_c: 0.27 cpd00134_c + 0.05 cpd00327_c + 0.01 cpd00517_c + 0.06 cpd01260_c + 0.1 cpd01695_c + 0.02 cpd03128_c + 0.17 cpd15238_c + 0.09 cpd15272_c + 0.24 cpd15274_c <=> cpd00010_c + 0.01 cpd15276_c

rxn09556_c: cpd00005_c + 0.01 cpd15193_c <=> cpd00006_c + 0.97 cpd00067_c + 0.01 cpd00517_c

rxn09574_c: cpd00136_c + cpd02557_c <=> cpd00012_c + cpd15208_c

rxn09582_c: cpd00083_c + 0.01 cpd15248_c <=> cpd00031_c + cpd00067_c + 0.01 cpd15264_c

rxn09583_c: cpd00083_c + 0.01 cpd15247_c <=> cpd00031_c + cpd00067_c + 0.01 cpd15263_c

rxn09584_c: cpd00083_c + 0.01 cpd15246_c <=> cpd00031_c + cpd00067_c + 0.01 cpd15262_c

rxn09585_c: cpd00083_c + 0.01 cpd15245_c <=> cpd00031_c + cpd00067_c + 0.01 cpd15261_c

rxn09586_c: cpd00083_c + 0.01 cpd15244_c <=> cpd00031_c + cpd00067_c + 0.01 cpd15260_c

rxn09587_c: cpd00083_c + 0.01 cpd15243_c <=> cpd00031_c + cpd00067_c + 0.01 cpd15259_c

rxn09588_c: 0.01 cpd11822_c + 0.01 cpd15264_c <=> 0.01 cpd15192_c + 0.01 cpd15258_c

rxn09589_c: 0.01 cpd11822_c + 0.01 cpd15263_c <=> 0.01 cpd15192_c + 0.01 cpd15257_c

rxn09590_c: 0.01 cpd11822_c + 0.01 cpd15262_c <=> 0.01 cpd15192_c + 0.01 cpd15256_c

rxn09591_c: 0.01 cpd11822_c + 0.01 cpd15261_c <=> 0.01 cpd15192_c + 0.01 cpd15255_c

rxn09592_c: 0.01 cpd11822_c + 0.01 cpd15260_c <=> 0.01 cpd15192_c + 0.01 cpd15254_c

rxn09593_c: 0.01 cpd11822_c + 0.01 cpd15259_c <=> 0.01 cpd15192_c + 0.01 cpd15253_c

rxn09594_c: 0.01 cpd11822_c + cpd15227_c <=> 0.01 cpd15192_c + 0.01 cpd15248_c

rxn09595_c: 0.01 cpd11822_c + cpd15226_c <=> 0.01 cpd15192_c + 0.01 cpd15247_c

rxn09596_c: 0.01 cpd11822_c + cpd15225_c <=> 0.01 cpd15192_c + 0.01 cpd15246_c

rxn09597_c: 0.01 cpd11822_c + cpd15224_c <=> 0.01 cpd15192_c + 0.01 cpd15245_c

rxn09598_c: 0.01 cpd11822_c + cpd15221_c <=> 0.01 cpd15192_c + 0.01 cpd15244_c

rxn09599_c: 0.01 cpd11822_c + cpd15220_c <=> 0.01 cpd15192_c + 0.01 cpd15243_c

rxn09611_c: cpd00001_c + cpd11791_c <=> cpd00027_c

rxn09705_c: cpd00067_e <=> cpd00031_c + cpd00067_c

rxn09778_c: cpd00137_e <=> cpd00061_c + cpd00137_c

rxn09780_c: cpd00067_e <=> cpd00002_c + cpd00008_c + cpd00067_c

rxn09815_c: <=> cpd00031_c

rxn09863_c: cpd00067_e <=> cpd00002_c + cpd00008_c + cpd00067_c

rxn09864_c: cpd00018_e + cpd00067_e <=> cpd00002_c + cpd00018_c + cpd00067_c

rxn09886_c: cpd00722_c + cpd01078_c <=> cpd00029_c + cpd00067_c + cpd01608_c

rxn09983_c: cpd00001_c + cpd00656_c <-- cpd00108_c

rxn09984_c: cpd00001_c + cpd02271_c <=> cpd00067_c + cpd00108_c

rxn09988_c: 3.0 cpd00001_c + cpd12115_c <=> 3.0 cpd00224_c

rxn09989_c: 5.0 cpd00001_c + cpd11594_c <=> 6.0 cpd00027_c

rxn09995_c: cpd00001_c + cpd12085_c <=> cpd00154_c

rxn10002_c: 3.0 cpd00007_c + cpd00028_c <=> 2.0 cpd00067_c + cpd15607_c

rxn10018_c: cpd00002_c + 0.01 cpd11423_c <=> cpd00008_c + cpd00067_c + 0.01 cpd11422_c

rxn10044_c: cpd00067_e + 2.0 cpd00109_e + cpd11451_e <=> 3.0 cpd00067_c + 2.0 cpd00110_c + cpd11606_c

rxn10045_c: 0.5 cpd00007_e + 2.0 cpd00067_e + cpd11451_e <=> cpd00001_c + 2.0 cpd00067_c + cpd11606_c

rxn10046_c: 0.5 cpd00007_e + 4.0 cpd00067_e + cpd11451_e <=> cpd00001_c + 4.0 cpd00067_c + cpd11606_c

rxn10063_c: cpd00001_c + cpd00067_c + cpd11668_c <=> cpd00047_c + cpd01896_c

rxn10079_c: cpd00001_c + cpd11624_c <=> cpd00067_c + 0.36 cpd00214_c + 0.02 cpd03847_c + 0.07 cpd15237_c + 0.5 cpd15269_c + 0.05 cpd15298_c + cpd15646_c

rxn10080_c: cpd00001_c + cpd15655_c <=> cpd00067_c + 0.36 cpd00214_c + 0.02 cpd03847_c + 0.07 cpd15237_c + 0.5 cpd15269_c + 0.05 cpd15298_c + cpd15648_c

rxn10081_c: cpd00001_c + cpd15653_c <=> cpd00067_c + 0.36 cpd00214_c + 0.02 cpd03847_c + 0.07 cpd15237_c + 0.5 cpd15269_c + 0.05 cpd15298_c + cpd15647_c

rxn10082_c: cpd00001_c + cpd15648_c <=> cpd00067_c + 0.36 cpd00214_c + cpd02090_c + 0.02 cpd03847_c + 0.07 cpd15237_c + 0.5 cpd15269_c + 0.05 cpd15298_c

rxn10083_c: cpd00001_c + cpd15647_c <=> cpd00067_c + 0.36 cpd00214_c + cpd00908_c + 0.02 cpd03847_c + 0.07 cpd15237_c + 0.5 cpd15269_c + 0.05 cpd15298_c

rxn10084_c: cpd00001_c + cpd15646_c <=> cpd00067_c + 0.36 cpd00214_c + cpd00507_c + 0.02 cpd03847_c + 0.07 cpd15237_c + 0.5 cpd15269_c + 0.05 cpd15298_c

rxn10109_c: cpd00080_c + 0.04 cpd11466_c + cpd11825_c + 0.14 cpd15239_c + 0.72 cpd15277_c + 0.1 cpd15294_c <=> cpd11422_c + 2.0 cpd11493_c

rxn10348_c: cpd11735_c <=> cpd15495_c

rxn10401_c: 2.0 cpd00001_c + cpd11594_c <=> 2.0 cpd00027_c

rxn10403_c: 0.1 cpd00134_c + 0.03 cpd00327_c + 0.03 cpd01695_c + 0.01 cpd11424_c + 0.07 cpd15801_c + 0.17 cpd15802_c + 0.01 cpd15803_c + 0.2 cpd15804_c + 0.34 cpd15805_c + 0.05 cpd15806_c <=> cpd00010_c + 0.01 cpd11422_c

rxn10410_c: cpd00080_c + 0.1 cpd00134_c + 0.03 cpd00327_c + 0.03 cpd01695_c + 0.07 cpd15801_c + 0.17 cpd15802_c + 0.01 cpd15803_c + 0.2 cpd15804_c + 0.34 cpd15805_c + 0.05 cpd15806_c <=> cpd00010_c + 0.84 cpd00067_c + 0.01 cpd11424_c

rxn10513_c: 1.8 cpd00067_e + cpd15867_e + cpd15897_e <=> 1.8 cpd00067_c + cpd15866_c + cpd15898_c

rxn10521_c: cpd00011_c + cpd00067_c + 2.0 cpd15877_c + cpd15892_c <=> cpd00001_c + 2.0 cpd15876_c + cpd15879_c

rxn10522_c: cpd00067_c + cpd15879_c + cpd15884_c <=> cpd15878_c + cpd15892_c

rxn10533_c: cpd00001_c + cpd00155_c <=> cpd00027_c + cpd15302_c

rxn10536_c: cpd00002_c + cpd00023_c + cpd11911_c <=> cpd00012_c + cpd00018_c

rxn10624_c: cpd00002_c + cpd00084_c + cpd00834_c --> cpd00012_c + cpd00018_c + cpd02666_c

rxn10628_c: cpd15912_c + cpd16000_c <=> cpd11493_c + cpd16004_c

rxn10631_c: cpd00017_c + cpd15973_c <=> cpd00019_c + cpd00067_c + cpd15974_c

rxn10632_c: cpd00001_c + cpd00017_c + cpd15964_c <=> cpd00019_c + cpd00067_c + cpd15979_c

rxn10633_c: cpd00001_c + cpd11822_c <=> cpd00067_c + cpd00867_c + cpd15309_c

rxn10635_c: cpd15991_c <=> cpd16031_c

rxn10636_c: 30.0 cpd00005_c + 47.0 cpd00067_c + 16.0 cpd11492_c + cpd15240_c <=> 17.0 cpd00001_c + 30.0 cpd00006_c + 16.0 cpd00011_c + 15.0 cpd11493_c + cpd15964_c

rxn10637_c: 2.0 cpd00001_c + cpd15937_c + cpd15967_c <=> cpd00010_c + cpd00011_c + cpd00018_c + 2.0 cpd00067_c + cpd15968_c

rxn10639_c: cpd00011_c + cpd15241_c <=> cpd00001_c + cpd15937_c

rxn10640_c: cpd00017_c + cpd15964_c <=> cpd00019_c + cpd00067_c + cpd15965_c

rxn10642_c: cpd15968_c <=> cpd16031_c

rxn10646_c: 2.0 cpd00001_c + cpd15937_c + cpd15986_c <=> cpd00010_c + cpd00011_c + cpd00018_c + 2.0 cpd00067_c + cpd15991_c

rxn10648_c: cpd15990_c <=> cpd16031_c

rxn10650_c: 2.0 cpd00001_c + cpd15937_c + cpd15981_c <=> cpd00010_c + cpd00011_c + cpd00018_c + 2.0 cpd00067_c + cpd15982_c

rxn10652_c: cpd15982_c <=> cpd16031_c

rxn10654_c: cpd00018_c + cpd00067_c + cpd15975_c <=> cpd11493_c + cpd15976_c

rxn10657_c: 2.0 cpd00001_c + cpd15937_c + cpd15989_c <=> cpd00010_c + cpd00011_c + cpd00018_c + 2.0 cpd00067_c + cpd15990_c

rxn10659_c: cpd16001_c <=> cpd16031_c

rxn10669_c: 2.0 cpd00001_c + cpd15937_c + cpd15976_c <=> cpd00010_c + cpd00011_c + cpd00018_c + 2.0 cpd00067_c + cpd16001_c

rxn10673_c: cpd00794_c + cpd16041_c + cpd16042_c <=> 2.0 cpd00001_c + cpd16049_c

rxn10675_c: cpd00052_c + cpd00067_c + cpd16004_c <=> cpd00012_c + cpd15936_c

rxn10680_c: cpd00794_c + 2.0 cpd01080_c + cpd16037_c <=> 3.0 cpd00001_c + cpd16052_c

rxn10710_c: cpd00002_c + 2.0 cpd00006_c + 3.5 cpd00007_c + 2.0 cpd00039_c + cpd00054_c + cpd00599_c + cpd00797_c + cpd01335_c <=> 5.0 cpd00001_c + 2.0 cpd00005_c + cpd00008_c + cpd00009_c + cpd00010_c + cpd00011_c + 2.0 cpd00067_c + cpd15940_c

rxn10712_c: 2.0 cpd00005_c + 3.0 cpd00067_c + cpd00519_c + cpd15950_c <=> cpd00001_c + 2.0 cpd00006_c + cpd00010_c + cpd00011_c + cpd16037_c

rxn10724_c: cpd00794_c + cpd16041_c <=> 2.0 cpd00001_c + cpd16046_c

rxn10726_c: cpd00794_c + cpd16041_c + cpd16042_c <=> 2.0 cpd00001_c + cpd16045_c

rxn10729_c: cpd00001_c + cpd15936_c <=> cpd00046_c + 2.0 cpd00067_c + cpd16004_c

rxn10730_c: cpd00002_c + 2.0 cpd00007_c + 2.0 cpd00039_c + cpd00054_c + cpd00599_c + cpd00797_c + cpd15274_c <=> 5.0 cpd00001_c + cpd00008_c + cpd00009_c + cpd00010_c + cpd00011_c + 2.0 cpd00067_c + cpd15972_c

rxn10736_c: cpd00794_c + cpd01080_c + 4.0 cpd16037_c <=> 5.0 cpd00001_c + cpd16006_c

rxn10737_c: cpd00121_c + cpd15419_c <=> cpd00046_c + cpd00067_c + cpd11822_c

rxn10739_c: 2.0 cpd00005_c + 3.0 cpd00067_c + cpd00519_c + cpd16037_c <=> cpd00001_c + 2.0 cpd00006_c + cpd00010_c + cpd00011_c + cpd16036_c

rxn10749_c: cpd00794_c + cpd16041_c <=> 2.0 cpd00001_c + cpd16048_c

rxn10758_c: 2.0 cpd00067_c + cpd00794_c + cpd15968_c + cpd16001_c <=> 2.0 cpd00001_c + cpd16041_c

rxn10765_c: cpd00794_c + cpd16041_c <=> 2.0 cpd00001_c + cpd16044_c

rxn10773_c: cpd00002_c + cpd00186_c + cpd16057_c <=> cpd00008_c + cpd00009_c + cpd00067_c + cpd16058_c

rxn10780_c: cpd00002_c + cpd00035_c + cpd16059_c <=> cpd00008_c + cpd00009_c + cpd00067_c + cpd16057_c

rxn10789_c: cpd03426_c + cpd15953_c <=> 4.0 cpd00067_c + cpd00557_c + cpd15972_c

rxn10790_c: cpd01476_c + cpd15953_c <=> cpd00028_c + 4.0 cpd00067_c + cpd15972_c

rxn10794_c: cpd00111_c + 2.0 cpd15996_c <=> 2.0 cpd00042_c + cpd15999_c

rxn10798_c: cpd15953_c <=> 2.0 cpd00067_c + cpd10515_c + cpd15972_c

rxn10808_c: cpd15952_c <=> cpd10516_c + cpd15940_c

rxn10814_c: cpd00006_c + 2.0 cpd15996_c <=> cpd00005_c + cpd00067_c + cpd15999_c

rxn10846_c: cpd02295_c + cpd02557_c <=> cpd00011_c + cpd00012_c + cpd00067_c + cpd15913_c

rxn10937_c: cpd00005_c + cpd11466_c + cpd11492_c <=> cpd00006_c + cpd00011_c + cpd00067_c + cpd11481_c + cpd11493_c

rxn10947_c: cpd00007_e + 5.98 cpd00067_e + 4.0 cpd00110_e <=> 1.99 cpd00001_c + 2.0 cpd00067_c + 4.0 cpd00109_c + 0.005 cpd00532_c

rxn11026_c: cpd00001_c + 2.0 cpd00002_c + cpd00011_c + cpd00053_c --> 2.0 cpd00008_c + cpd00009_c + cpd00023_c + cpd00146_c

rxn11051_c: cpd00001_c + cpd00193_c + cpd11421_c <=> cpd00018_c + cpd00081_c + cpd11420_c

rxn11062_c: cpd12370_c <=> cpd11493_c

rxn11081_c: cpd00067_e + cpd01018_e <-- cpd00367_c

rxn11089_c: cpd00009_c + cpd00412_c <-- cpd00151_c + cpd00509_c

rxn11092_c: cpd00002_c + cpd00101_c --> cpd00008_c + cpd00103_c

rxn11111_c: cpd00009_c + cpd00121_c <-- cpd00226_c + cpd00475_c

rxn11161_c: 3.0 cpd00004_c + 3.0 cpd00067_c + cpd00075_c --> cpd00001_c + 3.0 cpd00003_c + cpd00013_c

rxn11175_c: cpd00001_c + cpd00007_c + cpd00009_c + cpd00020_c <=> cpd00011_c + cpd00025_c + cpd00196_c

rxn11180_c: cpd03456_c <=> cpd00084_c

rxn11189_c: cpd00300_c --> cpd00011_c + cpd01092_c

rxn11197_c: cpd00001_c + cpd11493_c <=> cpd00834_c

rxn11198_c: cpd00010_c <=> cpd00045_c + cpd11493_c

rxn11199_c: cpd00002_c + cpd00018_c <-- cpd00008_c

rxn11204_c: cpd00065_c + cpd00869_c --> cpd00060_c + cpd00868_c

rxn11214_c: cpd00002_c + cpd00041_c + cpd00053_c --> cpd00012_c + cpd00018_c + cpd00023_c + cpd00132_c

rxn11219_c: cpd00007_c + cpd00668_c --> cpd00011_c + cpd00551_c

rxn11220_c: cpd00858_c <-- cpd02431_c

rxn11221_c: cpd00002_c + cpd00216_e <-- cpd00008_c + cpd00009_c + cpd00098_c

rxn11223_c: cpd00005_c + cpd00067_c + cpd02120_c <-- cpd00006_c + cpd00352_c

rxn11224_c: cpd00009_c + cpd00277_c --> cpd00207_c + cpd00475_c

rxn11225_c: cpd00009_c + cpd03279_c --> cpd00226_c + cpd00475_c

rxn11226_c: cpd00012_c --> 2.0 cpd00009_c

rxn11231_c: cpd00022_c + cpd11493_c <=> cpd00010_c + cpd11628_c

rxn11232_c: 2.0 cpd00001_c + cpd00038_c <-- cpd00047_c + cpd02978_c

rxn11251_c: cpd00002_c + cpd00035_c + cpd00773_c --> cpd00001_c + cpd00008_c + cpd00009_c + cpd00890_c

rxn11252_c: cpd00002_c + cpd00186_c + cpd00890_c --> cpd00001_c + cpd00008_c + cpd00009_c + cpd00525_c

rxn11253_c: cpd00002_c + cpd00039_c + cpd00525_c --> cpd00001_c + cpd00008_c + cpd00009_c + cpd02861_c

rxn11254_c: cpd00002_c + cpd00731_c + cpd02861_c <=> cpd00001_c + cpd00008_c + cpd00009_c + cpd02943_c

rxn11257_c: cpd00004_c + cpd00007_c + cpd00067_c --> cpd00001_c + cpd00003_c

rxn11259_c: cpd00009_c + cpd00654_c --> cpd00307_c + cpd00475_c

rxn11260_c: cpd00009_c + cpd00184_c --> cpd00151_c + cpd00475_c

rxn11269_c: cpd00061_c + cpd00599_e <=> cpd00020_c + cpd03698_c

rxn11279_c: cpd02720_c + cpd02882_c <-- cpd00009_c + 2.0 cpd02656_c

rxn11283_c: cpd00013_c + cpd00216_c <-- cpd00020_c + cpd00093_c

rxn11285_c: cpd00009_c + cpd00412_c --> cpd00092_c + cpd00475_c

rxn11292_c: cpd00001_c + cpd12085_c <=> 2.0 cpd00154_c

rxn11294_c: cpd00078_c + cpd00352_c --> cpd00010_c + cpd02724_c

rxn11296_c: 337.2 cpd00002_c + 17.7 cpd00206_c + 32.3 cpd00294_c + 17.7 cpd00296_c + 32.3 cpd00298_c <=> 337.2 cpd00008_c + 337.2 cpd00009_c + 100.0 cpd11461_c

rxn11297_c: 240.0 cpd00002_c + 26.2 cpd00018_c + 20.0 cpd00046_c + 21.6 cpd00091_c + 32.2 cpd00126_c <=> 240.0 cpd00008_c + 240.0 cpd00009_c + 100.0 cpd11613_c

rxn11303_c: 12.0 cpd00005_c + 12.0 cpd00067_c + 6.0 cpd11492_c + cpd11628_c <=> 6.0 cpd00001_c + 12.0 cpd00006_c + 6.0 cpd00011_c + cpd11466_c + 6.0 cpd11493_c

rxn11305_c: cpd00002_c + 8.0 cpd00003_c + 8.0 cpd00010_c + 8.0 cpd00015_c + cpd00214_c --> 8.0 cpd00004_c + cpd00012_c + cpd00018_c + 8.0 cpd00022_c + 8.0 cpd00067_c + 8.0 cpd00982_c

rxn11306_c: 14.0 cpd00005_c + 14.0 cpd00067_c + 7.0 cpd11492_c + cpd11628_c <=> 7.0 cpd00001_c + 14.0 cpd00006_c + 7.0 cpd00011_c + cpd11476_c + 7.0 cpd11493_c

rxn11307_c: 13.0 cpd00005_c + 13.0 cpd00067_c + 7.0 cpd11492_c + cpd11628_c <=> 7.0 cpd00001_c + 13.0 cpd00006_c + 7.0 cpd00011_c + 7.0 cpd11493_c + cpd16235_c

rxn11308_c: cpd00002_c + 9.0 cpd00003_c + 9.0 cpd00010_c + 9.0 cpd00015_c + cpd01080_c --> 9.0 cpd00004_c + cpd00012_c + cpd00018_c + 9.0 cpd00022_c + 9.0 cpd00067_c + 9.0 cpd00982_c

rxn11309_c: 16.0 cpd00005_c + 16.0 cpd00067_c + 8.0 cpd11492_c + cpd11628_c <=> 8.0 cpd00001_c + 16.0 cpd00006_c + 8.0 cpd00011_c + 8.0 cpd11493_c + cpd12458_c

rxn11310_c: 15.0 cpd00005_c + 15.0 cpd00067_c + 8.0 cpd11492_c + cpd11628_c <=> 8.0 cpd00001_c + 15.0 cpd00006_c + 8.0 cpd00011_c + 8.0 cpd11493_c + cpd11825_c

rxn11328_c: cpd00489_e <-- cpd15608_c

rxn11356_c: cpd00002_c + cpd00038_c + cpd00048_c --> cpd00009_c + cpd00012_c + cpd00031_c + cpd00193_c

rxn11359_c: cpd16001_c + cpd16246_c <=> cpd00067_c + cpd16247_c

rxn11360_c: cpd15991_c + cpd16246_c <=> cpd16248_c

rxn11366_c: cpd00523_c + cpd16249_c <=> cpd16246_c + cpd16273_c

rxn11369_c: cpd16271_c <=> cpd00009_c + cpd16252_c

rxn11370_c: cpd16272_c <=> cpd00009_c + cpd16253_c

rxn11371_c: cpd16273_c <=> cpd00009_c + cpd16254_c

rxn11372_c: cpd16274_c <=> cpd00009_c + cpd16255_c

rxn11373_c: cpd16275_c <=> cpd00009_c + cpd16256_c

rxn11375_c: cpd16252_c + cpd16254_c <=> cpd00794_c + cpd16041_c

rxn11376_c: cpd16252_c + cpd16255_c <=> cpd00794_c + cpd16042_c

rxn11377_c: cpd16252_c + cpd16256_c <=> cpd00794_c + cpd16041_c

rxn11417_c: 2.0 cpd00001_c + cpd01281_c + cpd11609_c <=> 3.0 cpd00081_c + cpd11610_c

rxn11508_c: cpd00002_c + cpd11623_c <=> cpd00008_c + cpd00009_c + cpd14837_c

rxn11512_c: cpd01180_c --> cpd16279_c

rxn11515_c: cpd00398_c --> cpd16280_c

rxn11518_c: cpd00857_c --> cpd16297_c

rxn11519_c: cpd16281_c --> cpd16282_c

rxn11520_c: cpd16282_c --> cpd16283_c

rxn11521_c: cpd16283_c --> cpd00369_c

rxn11522_c: cpd16297_c --> cpd16286_c

rxn11524_c: cpd16286_c --> cpd16285_c

rxn11525_c: cpd16285_c --> cpd01652_c

rxn11528_c: cpd00369_c --> cpd16287_c

rxn11529_c: cpd01712_c --> cpd16288_c

rxn11532_c: cpd16289_c <=> cpd16290_c

rxn11533_c: cpd16290_c <-- cpd16291_c

rxn11541_c: cpd06588_c --> cpd16296_c

rxn11542_c: cpd16290_c --> cpd16292_c

rxn11548_c: cpd00017_c + cpd11985_c <=> cpd00019_c + cpd00067_c + cpd12483_c

rxn11558_c: cpd00163_c <=> cpd00014_c + cpd16320_c

rxn11564_c: cpd00017_c + cpd11985_c <=> cpd00019_c + cpd00067_c + cpd12571_c

rxn11566_c: cpd00691_c <=> cpd00014_c + cpd16320_c

rxn11582_c: cpd00644_c + 2.0 cpd11610_c <=> cpd00001_c + cpd03534_c + 2.0 cpd11609_c

rxn11593_c: cpd02083_c + 3.0 cpd11609_c <=> cpd03418_c + 3.0 cpd11610_c

rxn11597_c: 3.0 cpd00001_c + cpd02311_c + cpd11609_c <=> cpd00013_c + cpd00047_c + cpd03303_c + cpd11610_c

rxn11598_c: cpd02374_c + 2.0 cpd11463_c <=> 2.0 cpd00001_c + cpd12738_c

rxn11615_c: cpd00007_c + cpd03081_c + cpd11610_c <=> cpd00001_c + cpd03094_c + cpd11609_c

rxn11617_c: cpd00007_c + cpd03140_c + cpd11610_c <=> 2.0 cpd00001_c + cpd11609_c + cpd12693_c

rxn11619_c: cpd03205_c + cpd11609_c <=> cpd03206_c + cpd11610_c

rxn11620_c: cpd03206_c + cpd11609_c <=> cpd03215_c + cpd11610_c

rxn11624_c: 2.0 cpd00007_c + cpd03215_c + 2.0 cpd11610_c <=> 4.0 cpd00001_c + cpd03217_c + 2.0 cpd11609_c

rxn11626_c: cpd00007_c + cpd03219_c + cpd11610_c <=> 2.0 cpd00001_c + cpd03220_c + cpd11609_c

rxn11630_c: cpd00001_c + cpd16473_c <-- cpd16472_c

rxn11634_c: cpd03389_c <=> cpd16318_c

rxn11638_c: cpd11609_c + cpd12753_c <=> cpd11610_c + cpd12756_c

rxn11648_c: cpd00001_c + cpd03836_c + cpd11609_c <=> cpd03838_c + cpd11610_c

rxn11651_c: cpd01892_c + cpd11610_c <=> cpd00001_c + cpd00401_c + cpd11609_c

rxn11664_c: cpd00094_c + cpd00229_c + cpd01003_c <-- cpd00263_c

rxn11665_c: cpd00001_c + cpd02762_c + cpd11609_c <=> cpd08629_c + cpd11610_c

rxn11666_c: cpd08629_c + cpd11609_c <=> cpd08630_c + cpd11610_c

rxn11667_c: cpd08630_c + cpd11609_c <=> cpd08631_c + cpd11610_c

rxn11675_c: cpd00011_c + cpd03688_c <-- cpd03689_c

rxn11685_c: cpd14868_c + cpd16466_c <-- cpd14869_c

rxn11687_c: cpd14533_c --> cpd14536_c

rxn11688_c: cpd14967_c --> cpd14537_c

rxn11698_c: cpd00007_c + cpd11476_c + cpd11610_c <=> cpd00001_c + cpd11609_c + cpd16235_c

rxn11699_c: cpd00001_c + cpd16235_c <=> cpd00067_c + cpd05274_c + cpd11493_c

rxn11700_c: cpd00001_c + cpd12458_c <=> cpd00067_c + cpd01080_c + cpd11493_c

rxn11705_c: cpd03466_c <=> cpd16334_c

rxn11706_c: cpd16334_c --> cpd16333_c

rxn11707_c: cpd16339_c <-- cpd04580_c

rxn11708_c: cpd08698_c <-- cpd16338_c

rxn11709_c: cpd16338_c --> cpd16339_c

rxn11747_c: cpd00002_c + cpd00054_c + cpd16440_c <=> cpd00012_c + cpd00018_c + cpd15565_c

rxn11748_c: cpd09280_c <=> cpd16439_c

rxn11753_c: cpd09288_c --> cpd04810_c

rxn11769_c: cpd02832_c --> cpd16420_c

rxn11770_c: cpd16420_c --> cpd16421_c

rxn11783_c: cpd16445_c --> cpd08049_c

rxn11784_c: cpd16445_c --> cpd16356_c

rxn11785_c: cpd16445_c --> cpd16357_c

rxn11786_c: cpd16357_c <-- cpd08049_c

rxn11792_c: cpd03684_c <=> cpd16390_c

rxn11793_c: cpd01072_c <=> cpd08589_c

rxn11796_c: cpd04382_c <=> cpd16360_c

rxn11797_c: cpd03017_c <=> cpd16361_c

rxn11800_c: cpd16360_c --> cpd16362_c

rxn11804_c: cpd04950_c <=> cpd04134_c + cpd16364_c

rxn11812_c: cpd04807_c <=> cpd16366_c

rxn11814_c: cpd16325_c <=> cpd04134_c + cpd16364_c

rxn11815_c: cpd16325_c <=> cpd04134_c + cpd16369_c

rxn11820_c: cpd16370_c <=> cpd16371_c

rxn11821_c: cpd16326_c --> cpd16375_c

rxn11823_c: cpd16375_c --> cpd07889_c

rxn11824_c: cpd16375_c --> cpd16383_c

rxn11825_c: cpd16374_c --> cpd16386_c

rxn11826_c: cpd16375_c --> cpd16386_c

rxn11827_c: cpd16383_c <=> cpd07889_c

rxn11829_c: cpd07889_c --> cpd16384_c

rxn11830_c: cpd07889_c <=> cpd16385_c

rxn11833_c: cpd04672_c <-- cpd16395_c

rxn11836_c: cpd16396_c <-- cpd16400_c

rxn11839_c: cpd16402_c --> cpd16399_c

rxn11843_c: cpd16406_c --> cpd16407_c

rxn11845_c: cpd04219_c --> cpd16405_c

rxn11846_c: cpd16405_c <-- cpd16408_c

rxn11848_c: cpd16405_c --> cpd16407_c

rxn11850_c: cpd04664_c --> cpd04666_c

rxn11860_c: cpd16457_c --> cpd16459_c

rxn11861_c: cpd16452_c --> cpd16460_c

rxn11866_c: cpd04417_c <=> cpd10135_c

rxn11867_c: cpd10135_c <=> cpd16461_c

rxn11868_c: cpd05005_c <=> cpd16462_c

rxn11869_c: cpd16462_c --> 2.0 cpd00067_c + cpd16463_c

rxn11870_c: cpd04740_c <=> cpd16412_c

rxn11871_c: cpd04740_c <=> cpd16411_c

rxn11872_c: cpd16412_c <=> cpd16413_c

rxn11873_c: cpd04740_c --> cpd16416_c

rxn11874_c: cpd16412_c --> cpd16416_c

rxn11881_c: cpd00350_c <-- cpd06515_c

rxn11882_c: cpd03829_c --> cpd04828_c

rxn11884_c: cpd00350_c <-- cpd06578_c

rxn11886_c: 2.0 cpd00067_c + cpd12788_c <=> cpd11713_c

rxn11888_c: cpd05531_c <=> cpd05653_c

rxn11889_c: cpd00776_c --> cpd05702_c

rxn11903_c: cpd00283_c <-- cpd06761_c

rxn11904_c: cpd00283_c <-- cpd06738_c

rxn11905_c: cpd00283_c <-- cpd06730_c

rxn11906_c: cpd00283_c <-- cpd01062_c

rxn11907_c: cpd00283_c <-- cpd06740_c

rxn11908_c: cpd00283_c <-- cpd01061_c

rxn11909_c: cpd00283_c <-- cpd06739_c

rxn11910_c: cpd03630_c <=> cpd06085_c

rxn11911_c: cpd03629_c <=> cpd04765_c

rxn11920_c: 2.0 cpd02234_c + cpd12837_c <=> 2.0 cpd00010_c + cpd12836_c

rxn11921_c: 6.06e-05 cpd15529_c + 5.57e-05 cpd15531_c + 5.15e-05 cpd15533_c + 5.78e-05 cpd15536_c + 5.33e-05 cpd15538_c + 4.95e-05 cpd15540_c + 5.35e-05 cpd15695_c + 5.35e-05 cpd15696_c + 6.06e-05 cpd15697_c + 5.8e-05 cpd15698_c + 5.8e-05 cpd15699_c + 5.57e-05 cpd15700_c + 5.14e-05 cpd15722_c + 5.14e-05 cpd15723_c + 5.78e-05 cpd15724_c + 5.55e-05 cpd15725_c + 5.55e-05 cpd15726_c + 5.33e-05 cpd15727_c + 2.85e-05 cpd15791_c + 3.11e-05 cpd15792_c + 2.63e-05 cpd15793_c + 2.74e-05 cpd15794_c + 2.74e-05 cpd15795_c + 2.97e-05 cpd15797_c + 2.97e-05 cpd15798_c + 2.85e-05 cpd15799_c <=> cpd16488_c

rxn11922_c: 0.000129 cpd15652_c + 0.000505 cpd15665_c <=> 0.000505 cpd15666_c + cpd16489_c

rxn11950_c: cpd15563_c <=> cpd16579_c

rxn11955_c: cpd00213_c + cpd00269_c <=> cpd00011_c + cpd16497_c

rxn11960_c: cpd00413_c + cpd00449_c <=> cpd00010_c + cpd00067_c + cpd16497_c

rxn11979_c: cpd00173_c + cpd11794_c <=> cpd00012_c + cpd00067_c + cpd01125_c

rxn11983_c: cpd00074_c + cpd11610_c <=> cpd00239_c + cpd11609_c

rxn11986_c: 3.0 cpd00001_c + cpd01078_c + 3.0 cpd11609_c <=> 2.0 cpd00067_c + cpd03387_c + 3.0 cpd11610_c

rxn11989_c: cpd00001_c + cpd02214_c <=> cpd00067_c + cpd01120_c

rxn11993_c: cpd11949_c <=> cpd16495_c

rxn11994_c: cpd01401_c + cpd11610_c <=> cpd11609_c + cpd12844_c

rxn11996_c: cpd11463_c + cpd12036_c <=> cpd00067_c + cpd11710_c + cpd11907_c

rxn12006_c: cpd00001_c + cpd03837_c + cpd11609_c <=> cpd11610_c + cpd16521_c

rxn12007_c: cpd04008_c + 3.0 cpd11610_c <=> 2.0 cpd00001_c + cpd04009_c + 3.0 cpd11609_c

rxn12011_c: cpd05876_c --> cpd05879_c

rxn12012_c: cpd09143_c + cpd11623_c <=> cpd00011_c + cpd09191_c

rxn12014_c: cpd00007_c + cpd11610_c + cpd14913_c <=> cpd00081_c + cpd11609_c + cpd14915_c

rxn12017_c: cpd00007_c + cpd11476_c + cpd11610_c <=> 2.0 cpd00001_c + cpd11609_c + cpd16235_c

rxn12022_c: cpd00007_c + cpd11610_c + cpd16338_c <=> cpd00001_c + cpd11609_c + cpd16339_c

rxn12034_c: cpd07889_c --> cpd16502_c

rxn12055_c: 2.0 cpd03829_c --> cpd04828_c

rxn12061_c: cpd00107_c --> cpd05178_c

rxn12062_c: cpd00119_c + cpd05178_c <-- cpd16504_c

rxn12063_c: cpd00119_c <-- cpd04649_c

rxn12065_c: cpd00323_c <-- cpd16505_c

rxn12066_c: cpd16505_c --> cpd07062_c

rxn12067_c: cpd16505_c <-- cpd03695_c

rxn12068_c: cpd01155_c <=> cpd07659_c

rxn12069_c: cpd01155_c <=> cpd07669_c

rxn12070_c: cpd07669_c <=> cpd07649_c

rxn12071_c: cpd01155_c <=> cpd07658_c

rxn12072_c: cpd00333_c + cpd03691_c <-- cpd07060_c

rxn12073_c: cpd00333_c + cpd00401_c + cpd03691_c <-- cpd00010_c + cpd07046_c

rxn12077_c: cpd03688_c <-- cpd03931_c

rxn12078_c: cpd03688_c <=> cpd00580_c

rxn12079_c: cpd00544_c + cpd03331_c <-- cpd07746_c

rxn12082_c: cpd00066_c --> cpd16507_c

rxn12083_c: cpd00374_c + cpd16507_c <-- cpd16508_c

rxn12084_c: cpd16508_c <-- 2.0 cpd00067_c + cpd16509_c

rxn12089_c: cpd00291_c <-- cpd06536_c

rxn12090_c: cpd02357_c <-- cpd03955_c

rxn12091_c: cpd02357_c <=> cpd16498_c

rxn12093_c: cpd02357_c + cpd16513_c <-- cpd16514_c

rxn12096_c: cpd16516_c <=> cpd16517_c

rxn12097_c: cpd16517_c <-- cpd08113_c

rxn12098_c: cpd08113_c --> cpd16518_c

rxn12099_c: cpd16518_c --> cpd04757_c

rxn12102_c: cpd03676_c --> cpd03926_c

rxn12103_c: cpd03926_c <-- cpd03942_c

rxn12104_c: cpd03081_c + cpd03107_c <=> cpd04717_c

rxn12105_c: 2.0 cpd03081_c <=> cpd06548_c

rxn12107_c: cpd00561_c <-- cpd16467_c

rxn12108_c: cpd00066_c <-- cpd05216_c

rxn12115_c: cpd08877_c <=> cpd07608_c

rxn12116_c: cpd08877_c <-- cpd07518_c

rxn12117_c: cpd08877_c <-- cpd07624_c

rxn12118_c: cpd00093_c <-- cpd16522_c

rxn12119_c: cpd16522_c <-- cpd16525_c

rxn12120_c: cpd16525_c <-- cpd07546_c

rxn12121_c: cpd07546_c --> cpd07626_c

rxn12122_c: cpd00093_c + cpd11311_c <-- cpd07619_c

rxn12123_c: cpd00041_c + cpd03773_c <-- cpd07619_c

rxn12124_c: 2.0 cpd00017_c + cpd00318_c --> 2.0 cpd00019_c + cpd05227_c

rxn12125_c: cpd05227_c --> cpd04744_c

rxn12126_c: cpd00318_c --> cpd03944_c

rxn12127_c: cpd00318_c <=> cpd06104_c

rxn12128_c: cpd00318_c <=> cpd03945_c

rxn12131_c: cpd02193_c <-- cpd16527_c

rxn12132_c: cpd16527_c --> cpd16526_c

rxn12133_c: cpd16526_c <-- cpd16528_c

rxn12134_c: cpd16528_c <-- cpd01304_c

rxn12138_c: cpd02193_c <=> cpd03932_c

rxn12139_c: cpd02193_c <-- cpd16538_c

rxn12140_c: cpd16538_c <=> cpd16531_c

rxn12142_c: cpd01062_c --> cpd03616_c

rxn12143_c: cpd01275_c <-- cpd06674_c

rxn12144_c: cpd01062_c <-- cpd06877_c

rxn12145_c: cpd01062_c --> cpd06800_c

rxn12148_c: cpd00160_c --> cpd03781_c

rxn12149_c: cpd03781_c <=> cpd07708_c

rxn12150_c: cpd03781_c <=> cpd07712_c

rxn12151_c: cpd03781_c <-- cpd03952_c

rxn12152_c: cpd03846_c <-- cpd07027_c

rxn12156_c: cpd02357_c <-- cpd16511_c

rxn12160_c: cpd00007_c + cpd03058_c + cpd11610_c <=> cpd00001_c + cpd00029_c + cpd00904_c + cpd11609_c

rxn12161_c: cpd00007_c + cpd00866_c + cpd11610_c <=> cpd00001_c + cpd00029_c + cpd00237_c + cpd11609_c

rxn12162_c: cpd12194_c <=> cpd16536_c

rxn12167_c: cpd16533_c <=> cpd01621_c

rxn12168_c: cpd16532_c <=> cpd01259_c

rxn12176_c: cpd00559_c --> cpd05534_c

rxn12184_c: cpd00350_c --> cpd00012_c + cpd00178_c + cpd15002_c

rxn12192_c: 4.2 cpd00002_c + 0.05 cpd01326_c + 0.13 cpd11770_c + 0.07 cpd12003_c + 0.03 cpd12036_c + 0.04 cpd12060_c + 0.2 cpd12100_c + 0.02 cpd12105_c + 0.04 cpd12132_c + 0.08 cpd12133_c + 0.04 cpd12164_c + 0.02 cpd12194_c + 0.04 cpd12226_c + 0.04 cpd12227_c + 0.01 cpd12228_c + 0.05 cpd12229_c + 0.02 cpd12255_c + 0.05 cpd12256_c + 0.04 cpd12313_c + 0.02 cpd12335_c + 0.01 cpd12336_c <=> 4.2 cpd00008_c + 4.2 cpd00009_c + 0.0988 cpd11463_c + 0.02 cpd11751_c + 0.13 cpd11906_c + 0.03 cpd11907_c + 0.04 cpd11908_c + 0.04 cpd11909_c + 0.02 cpd11910_c + 0.04 cpd11911_c + 0.04 cpd11912_c + 0.2 cpd11913_c + 0.01 cpd11914_c + 0.05 cpd11915_c + 0.07 cpd11916_c + 0.05 cpd11917_c + 0.02 cpd11918_c + 0.02 cpd11919_c + 0.04 cpd11920_c + 0.04 cpd11921_c + 0.05 cpd11922_c + 0.01 cpd11923_c + 0.08 cpd11924_c

rxn12213_c: cpd00004_c + 5.0 cpd00067_e + cpd11669_e <=> cpd00003_c + 4.0 cpd00067_c + cpd15291_c

rxn12223_c: cpd00003_c + cpd03607_c <-- cpd08449_c

rxn12226_c: cpd00007_c + cpd03448_c --> cpd03449_c

rxn12229_c: cpd16666_c <=> cpd00001_c + cpd16665_c

rxn12240_c: cpd16655_c <=> cpd00022_c + cpd02500_c

rxn12242_c: cpd00001_c + cpd00003_c + cpd02500_c --> cpd00004_c + cpd00197_c

rxn12258_c: cpd00001_c + cpd16649_c <=> cpd16650_c

rxn12260_c: cpd00010_c + cpd16651_c <=> cpd00022_c + cpd09842_c

rxn12262_c: cpd00002_c + cpd00010_c + cpd05193_c <=> cpd00012_c + cpd00018_c + cpd16652_c

rxn12265_c: cpd00001_c + cpd11467_c <=> cpd03115_c

rxn12266_c: cpd00015_c + cpd01695_c <=> cpd00982_c + cpd11467_c

rxn12291_c: cpd00010_c + cpd16647_c <=> cpd00022_c + cpd00650_c

rxn12299_c: cpd00001_c + cpd01006_c <-- cpd00029_c + cpd01553_c

rxn12309_c: cpd00004_c + cpd03127_c <=> cpd00003_c + cpd11466_c

rxn12310_c: cpd11484_c <=> cpd00001_c + cpd03127_c

rxn12335_c: 1.67 cpd00026_c + 1.66 cpd00083_c + 1.67 cpd02113_c <=> 0.67 cpd00014_c + 1.66 cpd00031_c + 1.67 cpd00297_c + cpd16667_c

rxn12336_c: 2.0 cpd00017_c + cpd11470_c <=> 2.0 cpd00060_c + 2.0 cpd03091_c + cpd14956_c

rxn12360_c: 1.37 cpd00002_c + 0.298 cpd00115_c + 0.202 cpd00241_c + 0.202 cpd00356_c + 0.298 cpd00357_c <=> 1.37 cpd00008_c + 1.37 cpd00009_c + cpd00012_c + 0.3087 cpd11461_c

rxn12463_c: 0.0002371 cpd15794_c + 0.0002576 cpd15797_c + 0.0002469 cpd15799_c <=> cpd16671_c

rxn12467_c: 0.0006954 cpd15696_c + 0.0007541 cpd15699_c <=> cpd16675_c

rxn12468_c: 0.0005013 cpd15536_c + 0.0004623 cpd15538_c + 0.000429 cpd15540_c <=> cpd16676_c

rxn12469_c: 0.0003338 cpd15722_c + 0.0003759 cpd15724_c + 0.0003608 cpd15725_c + 0.0003467 cpd15727_c <=> cpd16677_c

rxn12470_c: 0.0006676 cpd15723_c + 0.0007215 cpd15726_c <=> cpd16678_c

rxn12471_c: 7.3e-05 cpd15746_c + 7.39e-05 cpd15747_c + 7.22e-05 cpd15748_c <=> cpd16679_c

rxn12472_c: 5.44e-05 cpd15749_c + 5.55e-05 cpd15751_c + 5.51e-05 cpd15752_c + 5.48e-05 cpd15754_c <=> cpd16680_c

rxn12473_c: 0.0001089 cpd15750_c + 0.0001102 cpd15753_c <=> cpd16681_c

rxn12474_c: 3.94e-05 cpd15755_c + 3.97e-05 cpd15756_c + 3.92e-05 cpd15757_c <=> cpd16682_c

rxn12475_c: 2.95e-05 cpd15758_c + 2.98e-05 cpd15760_c + 2.97e-05 cpd15761_c + 2.96e-05 cpd15763_c <=> cpd16683_c

rxn12476_c: 5.9e-05 cpd15759_c + 5.94e-05 cpd15762_c <=> cpd16684_c

rxn12477_c: 3.53e-05 cpd15764_c + 3.55e-05 cpd15765_c + 3.51e-05 cpd15766_c <=> cpd16685_c

rxn12478_c: 2.64e-05 cpd15767_c + 2.67e-05 cpd15769_c + 2.66e-05 cpd15770_c + 2.65e-05 cpd15772_c <=> cpd16686_c

rxn12479_c: 5.28e-05 cpd15768_c + 5.31e-05 cpd15771_c <=> cpd16687_c

rxn12480_c: 5.32e-05 cpd15773_c + 5.37e-05 cpd15774_c + 5.27e-05 cpd15775_c <=> cpd16688_c

rxn12481_c: 3.97e-05 cpd15776_c + 4.02e-05 cpd15778_c + 4.01e-05 cpd15779_c + 3.99e-05 cpd15781_c <=> cpd16689_c

rxn12482_c: 7.94e-05 cpd15777_c + 8.01e-05 cpd15780_c <=> cpd16690_c

rxn12492_c: cpd00002_c + cpd00558_e --> cpd00008_c + cpd00009_c + cpd00264_c

rxn12518_c: cpd00146_e + 2.0 cpd01908_e <-- 2.0 cpd00067_c + cpd01908_c

rxn12663_c: <=> cpd00038_c

rxn12665_c: cpd00004_c + 3.0 cpd00067_e + cpd11606_e <=> cpd00003_c + 2.0 cpd00067_c + cpd11451_c

rxn12667_c: cpd00026_c + cpd11423_c <=> cpd00014_c + cpd00067_c + cpd11450_c

rxn12668_c: cpd00026_c + cpd11450_c <=> cpd00014_c + cpd00067_c + cpd11428_c

rxn12669_c: cpd00026_c + cpd11428_c <=> cpd00014_c + cpd00067_c + cpd11458_c

rxn12823_c: cpd00007_c + cpd00009_c + cpd00094_c --> cpd00001_c + cpd00011_c + cpd01844_c

rxn12852_c: cpd00009_e + cpd00067_e <=> cpd00002_c + cpd00008_c + cpd00009_c + cpd00067_c

rxn12853_c: cpd00004_c + cpd01351_c --> cpd00003_c + cpd15291_c

rxn12866_c: cpd00006_c + 4.0 cpd00067_c + cpd12300_c <=> cpd00005_c + cpd00517_c

rxn12867_c: cpd00001_c + cpd11791_c <=> cpd00027_c + cpd15303_c

rxn12878_c: cpd00017_c + cpd02064_c --> cpd00019_c + cpd15291_c

rxn12880_c: cpd00007_c + cpd16764_c --> cpd02064_c

rxn12898_c: cpd00264_e + cpd01351_e --> cpd00726_c + cpd15291_c

rxn12899_c: cpd00035_c + cpd00042_c <=> cpd01017_c + cpd12762_c

rxn12907_c: cpd00005_c + cpd00007_c + cpd01188_c <=> cpd00006_c + cpd15209_c

rxn12912_c: 3.0 cpd00007_c + cpd15210_c <=> cpd15212_c

rxn12913_c: 3.0 cpd00007_c + cpd03035_c <=> cpd15299_c

rxn12929_c: cpd16857_c <=> cpd00001_c + cpd11471_c

rxn12940_c: cpd00005_c + cpd00067_c + cpd11490_c <=> cpd00006_c + cpd16857_c

rxn12948_c: cpd00026_c + cpd15303_c <=> cpd00014_c + cpd11791_c

rxn12973_c: cpd00002_c + cpd00066_e + cpd11919_e <=> cpd00012_c + cpd00018_c + cpd00067_c + cpd12335_c

rxn12978_c: cpd00033_c + cpd00213_c <=> cpd00011_c + cpd11830_c

rxn12982_c: cpd00002_c + cpd00053_c <=> cpd00012_c + cpd00018_c + cpd12060_c

rxn12988_c: cpd00007_c + cpd12153_c <=> 2.0 cpd00011_c + cpd00791_c

rxn12989_c: cpd00007_e + cpd00791_e <=> cpd01476_c

rxn12990_c: cpd01476_c <=> cpd00028_c

rxn13007_c: cpd02569_c <-- cpd00123_c

rxn13019_c: cpd00002_c + cpd00322_e + cpd11915_e <=> cpd00012_c + cpd00018_c + cpd00067_c + cpd12256_c

rxn13020_c: cpd00623_c + cpd12196_c <=> cpd00010_c + cpd00167_c

rxn13021_c: cpd08926_c + cpd12196_c <=> cpd00010_c + cpd08927_c

rxn13023_c: cpd13404_c <=> cpd12196_c

rxn13026_c: cpd00017_c + cpd00774_c <=> cpd00019_c + cpd03426_c

rxn13032_c: cpd00002_c + cpd00023_c <=> cpd00012_c + cpd00018_c + cpd12227_c

rxn13033_c: cpd00002_c + cpd00065_c + cpd11615_c <=> cpd00012_c + cpd00018_c + cpd12336_c

rxn13037_c: cpd00004_c + cpd01351_e --> cpd00003_c + cpd15291_c

rxn13075_c: cpd00282_e + cpd01351_e --> cpd00247_c + cpd15291_c

rxn13077_c: 1.5 cpd00067_c + 2.0 cpd00109_c + cpd15291_c <=> 2.0 cpd00110_c + cpd01351_c

rxn13083_c: cpd00982_c + cpd01351_c --> cpd00015_c + cpd15291_c

rxn13130_c: cpd00047_e + cpd01351_e --> cpd00011_c + 2.0 cpd00067_c + cpd15291_c

rxn13142_c: cpd00003_c + cpd27436_c --> cpd00001_c + cpd00004_c + cpd00072_c

rxn13146_c: cpd00001_c + cpd00007_c + cpd01758_c <-- cpd00025_c + cpd00434_c + cpd01504_c

rxn13170_c: cpd00002_c + cpd00065_e + cpd11923_e <=> cpd00012_c + cpd00018_c + cpd00067_c + cpd12336_c

rxn13174_c: cpd00001_c + cpd00167_c <=> cpd00623_c

rxn13175_c: cpd00001_c + cpd08927_c <=> cpd08926_c

rxn13179_c: cpd00002_c + cpd00129_e + cpd11920_e <=> cpd00012_c + cpd00018_c + 2.0 cpd00067_c + cpd12164_c

rxn13181_c: cpd00002_c + cpd00084_e + cpd11910_e <=> cpd00012_c + cpd00018_c + cpd00067_c + cpd12255_c

rxn13184_c: cpd00026_c <=> cpd00014_c + cpd00067_c + cpd12121_c

rxn13217_c: cpd00001_c + cpd11966_c <=> cpd00029_c + cpd00067_c + cpd00363_c

rxn13241_c: cpd00001_c + cpd11683_c <=> cpd00029_c + cpd00067_c + cpd11741_c + cpd15304_c

rxn13242_c: cpd00175_c + cpd15304_c <=> cpd00014_c + cpd11683_c

rxn13253_c: cpd00001_c + cpd11601_c <=> cpd00280_c

rxn13254_c: 0.02 cpd00134_c + 0.03 cpd00327_c + 0.015 cpd01695_c + 0.01 cpd14514_c + 0.655 cpd15238_c + 0.01 cpd15241_c + 0.27 cpd15274_c <=> cpd00010_c + 0.935 cpd00067_c + 0.01 cpd16880_c

rxn13255_c: cpd00001_c + 0.01 cpd16880_c <=> cpd00067_c + 0.02 cpd00214_c + 0.03 cpd01080_c + 0.015 cpd03847_c + 0.01 cpd14514_c + 0.655 cpd15237_c + 0.01 cpd15240_c + 0.27 cpd15269_c

rxn13287_c: 0.02 cpd00134_c + 0.03 cpd00327_c + 0.015 cpd01695_c + 0.01 cpd02755_c + 0.655 cpd15238_c + 0.01 cpd15241_c + 0.27 cpd15274_c <=> cpd00010_c + 0.935 cpd00067_c + 0.01 cpd16883_c

rxn13288_c: cpd00001_c + 0.01 cpd16883_c <=> cpd00067_c + 0.02 cpd00214_c + 0.03 cpd01080_c + 0.01 cpd02755_c + 0.015 cpd03847_c + 0.655 cpd15237_c + 0.01 cpd15240_c + 0.27 cpd15269_c

rxn13295_c: cpd11735_c <=> cpd00155_c

rxn13297_c: cpd00001_c + cpd00155_c <-- cpd00027_c

rxn13299_c: cpd00009_c + cpd00155_c <-- cpd00089_c

rxn13300_c: cpd00026_c --> cpd00014_c + cpd00067_c + cpd00155_c

rxn13307_c: cpd00067_e <=> cpd00031_c + cpd00038_c + cpd00067_c

rxn13326_c: cpd00001_c + 0.01 cpd15243_c <=> cpd00067_c + cpd00867_c + cpd15220_c

rxn13327_c: cpd00001_c + 0.01 cpd15244_c <=> cpd00067_c + cpd00867_c + cpd15221_c

rxn13328_c: cpd00001_c + 0.01 cpd15245_c <=> cpd00067_c + cpd00867_c + cpd15224_c

rxn13329_c: cpd00001_c + 0.01 cpd15246_c <=> cpd00067_c + cpd00867_c + cpd15225_c

rxn13330_c: cpd00001_c + 0.01 cpd15247_c <=> cpd00067_c + cpd00867_c + cpd15226_c

rxn13331_c: cpd00001_c + 0.01 cpd15248_c <=> cpd00067_c + cpd00867_c + cpd15227_c

rxn13335_c: 0.02 cpd00134_c + 0.03 cpd00327_c + 0.01 cpd01188_c + 0.015 cpd01695_c + 0.655 cpd15238_c + 0.01 cpd15241_c + 0.27 cpd15274_c <=> cpd00010_c + 0.935 cpd00067_c + 0.01 cpd16885_c

rxn13336_c: cpd00001_c + 0.01 cpd16885_c <=> cpd00067_c + 0.02 cpd00214_c + 0.03 cpd01080_c + 0.01 cpd01188_c + 0.015 cpd03847_c + 0.655 cpd15237_c + 0.01 cpd15240_c + 0.27 cpd15269_c

rxn13342_c: 0.27 cpd00134_c + 0.05 cpd00327_c + 0.06 cpd01260_c + 0.1 cpd01695_c + 0.02 cpd03128_c + 0.17 cpd15238_c + 0.09 cpd15272_c + 0.24 cpd15274_c + 0.01 cpd16871_c <=> cpd00010_c + 0.01 cpd11624_c

rxn13347_c: cpd00001_c + 0.01 cpd15253_c <=> cpd00067_c + cpd15220_c + cpd16886_c

rxn13348_c: cpd00001_c + 0.01 cpd15254_c <=> cpd00067_c + cpd15221_c + cpd16886_c

rxn13349_c: cpd00001_c + 0.01 cpd15255_c <=> cpd00067_c + cpd15224_c + cpd16886_c

rxn13350_c: cpd00001_c + 0.01 cpd15256_c <=> cpd00067_c + cpd15225_c + cpd16886_c

rxn13351_c: cpd00001_c + 0.01 cpd15257_c <=> cpd00067_c + cpd15226_c + cpd16886_c

rxn13352_c: cpd00001_c + 0.01 cpd15258_c <=> cpd00067_c + cpd15227_c + cpd16886_c

rxn13353_c: cpd00001_c + 0.01 cpd15259_c <=> cpd00067_c + cpd15220_c + cpd16887_c

rxn13354_c: cpd00001_c + 0.01 cpd15260_c <=> cpd00067_c + cpd15221_c + cpd16887_c

rxn13355_c: cpd00001_c + 0.01 cpd15261_c <=> cpd00067_c + cpd15224_c + cpd16887_c

rxn13356_c: cpd00001_c + 0.01 cpd15262_c <=> cpd00067_c + cpd15225_c + cpd16887_c

rxn13357_c: cpd00001_c + 0.01 cpd15263_c <=> cpd00067_c + cpd15226_c + cpd16887_c

rxn13358_c: cpd00001_c + 0.01 cpd15264_c <=> cpd00067_c + cpd15227_c + cpd16887_c

rxn13360_c: <=> cpd00006_c

rxn13361_c: <=> cpd00003_c

rxn13367_c: cpd00022_c + cpd00363_c <=> cpd00010_c + cpd11966_c

rxn13375_c: cpd11624_c + cpd15192_c <=> cpd11677_c + cpd16871_c

rxn13378_c: cpd00001_c + 0.01 cpd13384_c <=> cpd00009_c + 0.01 cpd15287_c

rxn13379_c: cpd00002_c + 0.01 cpd15287_c <=> cpd00008_c + 0.01 cpd13384_c

rxn13380_c: cpd00001_c + 0.01 cpd12582_c <=> cpd00009_c + 0.01 cpd15288_c

rxn13381_c: cpd00001_c + 0.005 cpd11822_c <=> 0.27 cpd00214_c + 0.5 cpd00902_c + 0.05 cpd01080_c + 0.02 cpd01107_c + 0.06 cpd01741_c + 0.1 cpd03847_c + 0.17 cpd15237_c + 0.24 cpd15269_c + 0.09 cpd16888_c

rxn13382_c: cpd00001_c + 0.005 cpd11624_c <=> 0.27 cpd00214_c + 0.5 cpd00507_c + 0.05 cpd01080_c + 0.02 cpd01107_c + 0.06 cpd01741_c + 0.1 cpd03847_c + 0.17 cpd15237_c + 0.24 cpd15269_c + 0.09 cpd16888_c

rxn13384_c: cpd00001_c + 0.01 cpd11624_c <=> cpd00098_c + 0.01 cpd15276_c

rxn13401_c: cpd00001_c + 0.01 cpd11677_c <=> 0.27 cpd00214_c + 0.05 cpd01080_c + 0.02 cpd01107_c + 0.06 cpd01741_c + 0.1 cpd03847_c + 0.01 cpd15192_c + 0.17 cpd15237_c + 0.24 cpd15269_c + 0.09 cpd16888_c

rxn13416_c: 0.02 cpd00134_c + 0.03 cpd00327_c + 0.015 cpd01695_c + 0.01 cpd03221_c + 0.655 cpd15238_c + 0.01 cpd15241_c + 0.27 cpd15274_c <=> cpd00010_c + 0.935 cpd00067_c + 0.01 cpd16896_c

rxn13417_c: cpd00001_c + 0.01 cpd16896_c <=> cpd00067_c + 0.02 cpd00214_c + 0.03 cpd01080_c + 0.01 cpd03221_c + 0.015 cpd03847_c + 0.655 cpd15237_c + 0.01 cpd15240_c + 0.27 cpd15269_c

rxn13439_c: cpd10516_e + cpd16945_e <=> cpd16948_c

rxn13440_c: cpd16948_c <=> cpd10516_c + cpd16945_c

rxn13497_c: cpd16899_c <=> cpd16031_c

rxn13498_c: cpd16953_c <=> cpd16031_c

rxn13499_c: cpd16903_c <=> cpd16031_c

rxn13500_c: cpd16941_c <=> cpd16031_c

rxn13501_c: cpd16988_c <=> cpd16031_c

rxn13502_c: cpd16961_c <=> cpd16031_c

rxn13503_c: cpd16963_c <=> cpd16031_c

rxn13504_c: cpd16935_c <=> cpd16031_c

rxn13505_c: cpd16982_c <=> cpd16031_c

rxn13506_c: cpd16951_c <=> cpd16031_c

rxn13543_c: cpd16992_c <=> cpd16976_c

rxn13544_c: cpd16993_c <=> cpd16976_c

rxn13545_c: cpd16994_c <=> cpd16976_c

rxn13546_c: cpd16995_c <=> cpd16976_c

rxn13547_c: cpd16996_c <=> cpd16976_c

rxn13548_c: cpd16997_c <=> cpd16976_c

rxn13643_c: cpd00387_c --> cpd00008_c + cpd00067_c + cpd00155_c

rxn13650_c: cpd00002_c + cpd11430_c + cpd11493_c <=> cpd00012_c + cpd00018_c + cpd17011_c

rxn13651_c: cpd00002_c + cpd11436_c + cpd11493_c <=> cpd00012_c + cpd00018_c + cpd17012_c

rxn13652_c: cpd00002_c + cpd11440_c + cpd11493_c <=> cpd00012_c + cpd00018_c + cpd17013_c

rxn13653_c: cpd00002_c + cpd11431_c + cpd11493_c <=> cpd00012_c + cpd00018_c + cpd17007_c

rxn13654_c: cpd00002_c + cpd11493_c + cpd17030_c <=> cpd00012_c + cpd00018_c + cpd17031_c

rxn13655_c: cpd00002_c + cpd11493_c + cpd17020_c <=> cpd00012_c + cpd00018_c + cpd17021_c

rxn13656_c: cpd00002_c + cpd11493_c + cpd17034_c <=> cpd00012_c + cpd00018_c + cpd17029_c

rxn13657_c: cpd00002_c + cpd11493_c + cpd17019_c <=> cpd00012_c + cpd00018_c + cpd17018_c

rxn13658_c: cpd00002_c + cpd11493_c + cpd17008_c <=> cpd00012_c + cpd00018_c + cpd17009_c

rxn13661_c: cpd00017_c + cpd11425_c <=> cpd00019_c + cpd00067_c + cpd11606_c

rxn13670_c: 11.0 cpd00005_c + 17.0 cpd00067_c + cpd00086_c + 6.0 cpd11492_c <=> 6.0 cpd00001_c + 11.0 cpd00006_c + cpd00010_c + 6.0 cpd00011_c + 5.0 cpd11493_c + cpd17031_c

rxn13674_c: 13.0 cpd00005_c + 20.0 cpd00067_c + cpd00086_c + 7.0 cpd11492_c <=> 7.0 cpd00001_c + 13.0 cpd00006_c + cpd00010_c + 7.0 cpd00011_c + 6.0 cpd11493_c + cpd17021_c

rxn13675_c: 2.0 cpd00005_c + 3.0 cpd00067_c + cpd00086_c + cpd11492_c <=> cpd00001_c + 2.0 cpd00006_c + cpd00010_c + cpd00011_c + cpd17010_c

rxn13676_c: 2.0 cpd00005_c + 3.0 cpd00067_c + cpd00481_c + cpd11492_c <=> cpd00001_c + 2.0 cpd00006_c + cpd00010_c + cpd00011_c + cpd17015_c

rxn13677_c: 2.0 cpd00005_c + 3.0 cpd00067_c + cpd01882_c + cpd11492_c <=> cpd00001_c + 2.0 cpd00006_c + cpd00010_c + cpd00011_c + cpd17014_c

rxn13678_c: cpd11451_e + 2.0 cpd17005_e <=> 2.0 cpd00067_c + 2.0 cpd00149_c + cpd11606_c

rxn13683_c: 1.5 cpd11451_e + cpd11595_e <=> cpd00001_c + 1.5 cpd11606_c + cpd17003_c

rxn13690_c: cpd00282_c + cpd11606_c <=> cpd00247_c + cpd11451_c

rxn13692_c: cpd08021_e + cpd11451_e <=> cpd00001_c + cpd00450_c + cpd11606_c

rxn13701_c: cpd00047_e + 2.0 cpd00067_e + cpd11606_e <=> cpd00011_c + cpd00067_c + cpd11451_c

rxn13702_c: 2.0 cpd10516_e + cpd11451_e <=> 2.0 cpd00067_c + 2.0 cpd10515_c + cpd11606_c

rxn13706_c: cpd00106_e + cpd11451_e <=> cpd00036_c + cpd11606_c

rxn13710_c: cpd00002_c + cpd00027_c --> cpd00008_c + cpd00009_c + cpd00067_c + cpd00155_c

rxn13714_c: cpd17001_c <=> cpd00001_c + cpd00231_c

rxn13715_c: cpd11606_e + cpd11640_e <=> cpd11451_c

rxn13717_c: cpd00221_c + cpd11606_c <=> cpd00020_c + cpd11451_c

rxn13719_c: cpd00159_c + cpd11606_c <=> cpd00020_c + cpd11451_c

rxn13723_c: 2.0 cpd00067_e + cpd11451_e + cpd17028_e <=> 2.0 cpd00001_c + cpd00030_c + cpd11606_c

rxn13725_c: cpd03002_c + cpd17004_c <=> cpd00046_c + cpd00067_c + cpd17023_c

rxn13727_c: cpd00004_c + 5.0 cpd00067_e + cpd11606_e <=> cpd00003_c + 4.0 cpd00067_c + cpd11451_c

rxn13732_c: cpd00067_e + cpd00209_e + cpd11451_e <=> cpd00001_c + cpd00075_c + cpd11606_c

rxn13735_c: cpd00067_e + cpd00075_e + 3.0 cpd11451_e <=> 2.0 cpd00001_c + cpd00013_c + 3.0 cpd11606_c

rxn13736_c: cpd00080_c + 0.25 cpd11825_c + 0.38 cpd15239_c + 0.02 cpd15268_c + 0.13 cpd15277_c + 0.13 cpd17007_c + 0.07 cpd17009_c + 0.12 cpd17011_c + 0.33 cpd17012_c + 0.15 cpd17013_c + 0.03 cpd17018_c + 0.25 cpd17021_c + 0.02 cpd17029_c + 0.12 cpd17031_c <=> cpd11422_c + 2.0 cpd11493_c

rxn13738_c: cpd00001_c + cpd11456_c <=> 0.065 cpd00214_c + 0.01 cpd01080_c + 0.06 cpd11430_c + 0.065 cpd11431_c + 0.165 cpd11436_c + 0.075 cpd11440_c + 0.19 cpd15237_c + 0.125 cpd15269_c + cpd15647_c + 0.035 cpd17008_c + 0.015 cpd17019_c + 0.125 cpd17020_c + 0.06 cpd17030_c + 0.01 cpd17034_c

rxn13739_c: cpd00001_c + cpd11652_c <=> 0.065 cpd00214_c + 0.01 cpd01080_c + 0.06 cpd11430_c + 0.065 cpd11431_c + 0.165 cpd11436_c + 0.075 cpd11440_c + 0.19 cpd15237_c + 0.125 cpd15269_c + cpd15648_c + 0.035 cpd17008_c + 0.015 cpd17019_c + 0.125 cpd17020_c + 0.06 cpd17030_c + 0.01 cpd17034_c

rxn13742_c: 19974.0 cpd00001_c + 19944.0 cpd00038_c + 1083.0 cpd11770_c + 777.0 cpd12003_c + 449.0 cpd12036_c + 524.0 cpd12060_c + 934.0 cpd12100_c + 257.0 cpd12105_c + 598.0 cpd12132_c + 596.0 cpd12133_c + 436.0 cpd12164_c + 253.0 cpd12194_c + 660.0 cpd12226_c + 612.0 cpd12227_c + 177.0 cpd12228_c + 566.0 cpd12229_c + 107.0 cpd12255_c + 506.0 cpd12256_c + 507.0 cpd12313_c + 330.0 cpd12335_c + 125.0 cpd12336_c + 505.0 cpd15249_c <=> 19944.0 cpd00009_c + 19944.0 cpd00031_c + 28346.0 cpd00067_c + 253.0 cpd11751_c + 1083.0 cpd11906_c + 449.0 cpd11907_c + 507.0 cpd11908_c + 660.0 cpd11909_c + 107.0 cpd11910_c + 524.0 cpd11911_c + 612.0 cpd11912_c + 934.0 cpd11913_c + 177.0 cpd11914_c + 506.0 cpd11915_c + 777.0 cpd11916_c + 505.0 cpd11917_c + 257.0 cpd11918_c + 330.0 cpd11919_c + 436.0 cpd11920_c + 598.0 cpd11921_c + 566.0 cpd11922_c + 125.0 cpd11923_c + 596.0 cpd11924_c + cpd17033_c

rxn13745_c: cpd00067_e + cpd00081_e + 3.0 cpd11451_e <=> 3.0 cpd00001_c + cpd00067_c + cpd00239_c + 3.0 cpd11606_c

rxn13749_c: cpd00268_e + cpd11451_e <=> cpd00067_c + cpd00081_c + cpd00239_c + cpd11606_c

rxn13751_c: 2.0 cpd00067_e + cpd01414_e + cpd11451_e <=> 2.0 cpd00067_c + 2.0 cpd00268_c + cpd11606_c

rxn13754_c: cpd11451_e + cpd17038_e <=> 2.0 cpd00067_c + cpd11606_c + cpd17037_c

rxn13760_c: cpd00041_c + cpd11606_c <=> cpd03470_c + cpd11451_c

rxn13772_c: cpd00812_c <-- cpd00011_c + cpd17039_c

rxn13785_c: 0.5 cpd00080_c + 0.13 cpd15239_c + 0.03 cpd15268_c + 0.33 cpd15277_c + 0.51 cpd15572_c <=> 0.01 cpd11422_c + cpd11493_c

rxn13792_c: 0.5 cpd00007_c + cpd11468_c <=> cpd17046_c

rxn13793_c: 0.5 cpd00007_c + cpd11466_c <=> cpd17047_c

rxn13804_c: cpd00363_c + cpd11669_c <=> cpd00071_c + cpd15561_c

rxn13805_c: 2.0 cpd00719_c <=> 2.0 cpd00031_c + cpd11949_c

rxn13807_c: cpd11949_c <=> cpd17075_c

rxn13810_c: cpd01048_c + cpd15481_c <=> cpd04098_c + cpd13050_c

rxn13815_c: cpd00080_c + 0.02 cpd11427_c <=> cpd00046_c + cpd00067_c + 0.02 cpd11454_c

rxn13816_c: cpd00054_c + 0.02 cpd11427_c <=> cpd00046_c + cpd00067_c + 0.02 cpd11455_c

rxn13817_c: 0.02 cpd11427_c + 0.02 cpd11652_c <=> cpd00046_c + cpd00067_c + 0.02 cpd12801_c

rxn13818_c: 0.04 cpd11652_c <=> cpd00100_c + 0.02 cpd12801_c

rxn13820_c: 2.0 cpd00109_e + cpd15561_e <=> 2.0 cpd00067_c + 2.0 cpd00110_c + cpd11669_c

rxn13821_c: 0.5 cpd00007_e + 2.5 cpd00067_e + cpd15561_e <=> cpd00001_c + 2.5 cpd00067_c + cpd11669_c

rxn13824_c: cpd00282_c + cpd11669_c <=> cpd00247_c + cpd15561_c

rxn13826_c: cpd00113_c + cpd04659_c <=> cpd00012_c + cpd00283_c

rxn13830_c: cpd00047_e + 3.0 cpd00067_e + cpd11669_e <=> cpd00011_c + 2.0 cpd00067_c + cpd15561_c

rxn13835_c: cpd00080_c + cpd11669_c <=> cpd00095_c + cpd15561_c

rxn13843_c: cpd17051_c <=> cpd00001_c + cpd15572_c

rxn13844_c: cpd00005_c + cpd00067_c + cpd14938_c <=> cpd00006_c + cpd17051_c

rxn13848_c: cpd00159_c + cpd11669_c <=> cpd00020_c + cpd15561_c

rxn13852_c: cpd00130_c + cpd11669_c <=> cpd00032_c + cpd15561_c

rxn13856_c: cpd00004_c + 3.0 cpd00067_e + cpd11669_e <=> cpd00003_c + 2.0 cpd00067_c + cpd15561_c

rxn13857_c: cpd00004_c + cpd00067_c + cpd11669_c <=> cpd00003_c + cpd15561_c

rxn13858_c: cpd00005_c + cpd00067_c + cpd11669_c <=> cpd00006_c + cpd15561_c

rxn13859_c: cpd00067_c + cpd00103_c + cpd08325_c --> cpd00012_c + cpd00873_c

rxn13861_c: cpd00873_c + cpd01997_c <-- cpd00067_c + cpd02904_c + cpd08325_c

rxn13862_c: 2.0 cpd00067_e + cpd00209_e + cpd15561_e <=> cpd00001_c + 2.0 cpd00067_c + cpd00075_c + cpd11669_c

rxn13863_c: 2.0 cpd00418_c + cpd15561_c <=> cpd00001_c + cpd00659_c + cpd11669_c

rxn13864_c: cpd00659_c + cpd15561_c <=> cpd00001_c + cpd00528_c + cpd11669_c

rxn13872_c: cpd11468_c + cpd17046_c + cpd17068_c <=> 2.0 cpd11493_c + cpd17066_c

rxn13887_c: cpd00001_c + 0.02 cpd11454_c <=> cpd00009_c + 0.02 cpd11652_c

rxn13889_c: cpd00052_c + cpd00067_c + 0.02 cpd11422_c <=> cpd00012_c + 0.02 cpd11427_c

rxn13891_c: cpd00001_c + cpd00020_c + cpd11669_c <=> cpd00011_c + cpd00029_c + cpd15561_c

rxn13894_c: 0.02 cpd11455_c <=> cpd00011_c + 0.02 cpd11456_c

rxn13897_c: cpd00027_c + cpd11669_c <=> cpd00170_c + cpd15561_c

rxn13898_c: cpd00005_c + cpd11669_c <=> cpd00006_c + cpd17097_c

rxn13907_c: cpd00982_c + cpd11669_c <=> cpd00015_c + cpd15561_c

rxn13908_c: cpd00005_c + cpd00067_c + cpd12566_c <=> cpd00006_c + cpd17089_c

rxn13929_c: 0.04 cpd15655_c <=> cpd00100_c + 0.02 cpd15650_c

rxn13938_c: 0.04 cpd15655_c <=> cpd00100_c + 0.02 cpd15650_c

rxn13951_c: cpd00001_c + cpd11624_c <=> cpd00188_c + cpd02601_c

rxn13964_c: cpd00001_c + cpd00187_c + cpd11609_c <=> cpd00013_c + cpd00055_c + cpd11610_c

rxn13987_c: cpd00005_c + cpd00007_c + cpd00067_c + cpd00542_c <=> cpd00001_c + cpd00006_c + cpd12754_c

rxn13992_c: cpd11610_c + cpd17480_c <=> cpd03240_c + cpd11609_c

rxn13996_c: cpd03491_c + cpd11770_c <=> cpd00067_c + cpd11906_c + cpd17675_c

rxn14006_c: cpd00007_c + cpd00752_c + cpd11610_c <=> cpd00001_c + cpd00011_c + cpd00778_c + cpd11609_c

rxn14015_c: cpd11609_c + cpd17483_c <=> cpd03230_c + cpd11610_c

rxn14018_c: cpd00001_c + cpd00336_c + cpd11609_c <=> cpd03545_c + cpd11610_c

rxn14021_c: 2.0 cpd00008_c + 2.0 cpd00009_c + cpd00013_c + cpd11609_c <=> cpd00001_c + 2.0 cpd00002_c + cpd00165_c + cpd11610_c

rxn14035_c: cpd09194_c <-- cpd08716_c

rxn14062_c: --> cpd03648_c

rxn14066_c: <=> cpd12626_c

rxn14073_c: cpd00202_c + cpd11615_c <=> cpd00012_c + cpd12546_c

rxn14113_c: cpd11612_c <=> cpd17455_c

rxn14128_c: 2.0 cpd00766_c + cpd11609_c <=> 2.0 cpd00335_c + cpd11610_c

rxn14141_c: cpd00017_c + cpd17470_c <=> cpd00019_c + cpd00067_c + cpd12481_c

rxn14147_c: 2.0 cpd12100_c + cpd17666_c <=> 2.0 cpd00067_c + 2.0 cpd11913_c + cpd17667_c

rxn14158_c: cpd00222_c + cpd11609_c <=> cpd03889_c + cpd11610_c

rxn14171_c: cpd00208_c + cpd11609_c <=> cpd03199_c + cpd11610_c

rxn14188_c: cpd00066_c <-- cpd17425_c

rxn14190_c: cpd00367_c --> cpd00084_c + cpd17424_c

rxn14195_c: 2.0 cpd00059_c + cpd11609_c <=> 2.0 cpd00766_c + cpd11610_c

rxn14198_c: 2.0 cpd12100_c + cpd17667_c <=> 2.0 cpd00067_c + cpd03493_c + 2.0 cpd11913_c

rxn14208_c: cpd00033_c + cpd02197_c + cpd11610_c + cpd17467_c <=> cpd00001_c + cpd00087_c + cpd11609_c + cpd17468_c

rxn14219_c: cpd11770_c + cpd17675_c <=> cpd00067_c + cpd11906_c + cpd17674_c

rxn14221_c: cpd00001_c <=> cpd17398_c

rxn14251_c: cpd04008_c <=> cpd04010_c

rxn14252_c: cpd03889_c + cpd11609_c <=> cpd01793_c + cpd11610_c

rxn14254_c: 2.0 cpd00001_c + cpd03428_c + 3.0 cpd11610_c <=> 2.0 cpd00164_c + cpd03431_c + 3.0 cpd11609_c

rxn14265_c: cpd00025_c + cpd11610_c <=> 2.0 cpd00001_c + cpd11609_c

rxn14272_c: cpd00003_c + cpd00057_c <=> cpd00004_c + cpd00067_c + cpd11872_c

rxn14277_c: cpd00481_c + cpd11609_c <=> cpd02187_c + cpd11610_c

rxn14297_c: cpd03492_c + cpd12100_c <=> cpd00067_c + cpd11913_c + cpd17666_c

rxn14309_c: cpd00002_c + cpd00738_c + cpd11910_c <=> cpd00012_c + cpd00018_c + cpd17286_c

rxn14315_c: cpd12662_c <=> cpd11949_c

rxn14326_c: cpd00006_c + cpd00012_c + cpd00018_c + cpd11631_c <=> cpd00002_c + cpd00005_c + cpd00067_c + cpd11694_c

rxn14329_c: cpd00001_c + cpd00217_c <-- cpd00027_c

rxn14343_c: cpd00017_c + cpd17469_c <=> cpd00019_c + cpd00067_c + cpd12482_c

rxn14349_c: cpd00085_c --> cpd01560_c

rxn14356_c: cpd11609_c + cpd11665_c <=> cpd11610_c + cpd11669_c

rxn14358_c: cpd00001_c + cpd00075_c + cpd11609_c <=> cpd00209_c + cpd11610_c

rxn14366_c: cpd08729_c <-- cpd09194_c

rxn14367_c: cpd11609_c + cpd17481_c <=> cpd03241_c + cpd11610_c

rxn14374_c: cpd00017_c + cpd11628_c <=> cpd00147_c + cpd11493_c + cpd18032_c

rxn14401_c: cpd00080_c + cpd11628_c <=> cpd00517_c + cpd11493_c

rxn14402_c: cpd00517_c + cpd11628_c <=> cpd11422_c + cpd11493_c

rxn15000_c: cpd00001_c + cpd11672_c <=> 2.0 cpd12039_c

rxn15016_c: cpd12036_c <=> cpd11710_c

rxn15031_c: cpd00190_c + cpd11609_c <=> cpd00170_c + cpd11610_c

rxn15032_c: cpd11609_c + cpd19001_c <=> cpd00170_c + cpd11610_c

rxn15041_c: cpd00514_c <=> cpd00012_c + cpd11461_c

rxn15042_c: cpd00002_c + cpd11703_c <=> cpd00012_c + cpd00018_c + cpd11461_c

rxn15043_c: cpd00003_c + cpd11703_c <=> cpd00018_c + cpd00355_c + cpd11461_c

rxn15054_c: cpd00001_c + cpd11609_c + cpd11616_c <=> cpd11610_c + cpd19000_c

rxn15089_c: cpd00083_c <=> cpd00031_c + cpd11685_c

rxn15160_c: cpd00001_c --> cpd02416_c

rxn15182_c: cpd00001_c + cpd12120_c <=> 2.0 cpd00138_c

rxn15183_c: cpd00001_c + cpd12120_c <=> 2.0 cpd19009_c

rxn15184_c: cpd00001_c + cpd12120_c <=> 2.0 cpd01490_c

rxn15193_c: cpd01487_c <-- cpd00001_c

rxn15194_c: cpd01422_c <-- cpd00001_c

rxn15199_c: cpd03844_c + cpd11609_c <=> cpd00801_c + cpd11610_c

rxn15200_c: cpd03845_c + cpd11609_c <=> cpd00801_c + cpd11610_c

rxn15232_c: cpd01354_c + cpd11609_c <=> cpd03199_c + cpd11610_c

rxn15246_c: cpd00001_c --> cpd01629_c

rxn15247_c: cpd00001_c --> cpd01698_c

rxn15251_c: cpd00001_c --> cpd19001_c

rxn15289_c: cpd05263_c <-- cpd00001_c

rxn15292_c: cpd00001_c --> cpd05262_c

rxn15299_c: cpd00001_c + cpd11657_c <=> cpd00665_c + cpd11976_c

rxn15300_c: cpd00001_c + cpd11657_c <=> cpd00179_c + cpd11976_c

rxn15304_c: cpd01808_c <-- cpd00776_c

rxn15314_c: cpd19001_c --> cpd19006_c

rxn15318_c: cpd00336_c + cpd11610_c <=> cpd00488_c + cpd11609_c

rxn15321_c: cpd00001_c + cpd11601_c <=> cpd00116_c + cpd11686_c

rxn15366_c: cpd00054_c <-- cpd00084_c

rxn15367_c: cpd00550_c <-- cpd00587_c

rxn15371_c: cpd00051_c + cpd00550_c --> cpd00064_c + cpd01190_c

rxn15372_c: cpd00550_c + cpd00586_c --> cpd00404_c + cpd01190_c

rxn15374_c: cpd00001_c + cpd00554_c + cpd11609_c <=> cpd00777_c + cpd11610_c

rxn15375_c: cpd00001_c + cpd11609_c + cpd16501_c <=> cpd00777_c + cpd11610_c

rxn15376_c: cpd00001_c --> cpd03844_c

rxn15377_c: cpd00001_c --> cpd03845_c

rxn15388_c: cpd00001_c + cpd11672_c <=> cpd00009_c

rxn15392_c: cpd00760_c + cpd11609_c <=> cpd02125_c + cpd11610_c

rxn15423_c: cpd01806_c + cpd11609_c <=> cpd01884_c + cpd11610_c

rxn15424_c: cpd01883_c + cpd11609_c <=> cpd01884_c + cpd11610_c

rxn15434_c: cpd00113_c <=> cpd00012_c + cpd00067_c + cpd12077_c

rxn15452_c: cpd00076_c <-- cpd00190_c

rxn15453_c: cpd00076_c <-- cpd19001_c

rxn15454_c: cpd00002_c + cpd00023_c <=> cpd00008_c + cpd00009_c + cpd06227_c

rxn15455_c: cpd02234_c <=> cpd00010_c + cpd12836_c

rxn15458_c: cpd00001_c + cpd02896_c <=> cpd00067_c + cpd02416_c + cpd02486_c

rxn15498_c: cpd03293_c + 2.0 cpd11608_c <=> 2.0 cpd00001_c + cpd12848_c

rxn15504_c: 2.0 cpd00001_c + cpd03428_c + 3.0 cpd11610_c <=> cpd03431_c + 2.0 cpd05264_c + 3.0 cpd11609_c

rxn15527_c: cpd00005_c + cpd00022_c + cpd00067_c + cpd00070_c <=> 2.0 cpd00001_c + 2.0 cpd00006_c + 2.0 cpd00010_c + 2.0 cpd00011_c + cpd00487_c

rxn15530_c: cpd00004_c + cpd00005_c + cpd00022_c + cpd00067_c + cpd00070_c <=> cpd00001_c + cpd00003_c + cpd00006_c + cpd00010_c + cpd00011_c + cpd12196_c

rxn15532_c: cpd00027_c + cpd11735_c <=> cpd00179_c

rxn15533_c: cpd11735_c + cpd19001_c <=> cpd00665_c

rxn15542_c: cpd00113_c + cpd00350_c <=> cpd00012_c + cpd12788_c

rxn15543_c: cpd00001_c <--

rxn15549_c: cpd00033_c + 2.0 cpd11609_c <=> cpd00011_c + cpd00150_c + 2.0 cpd11610_c

rxn15553_c: cpd00035_c + cpd00094_c + cpd00229_c <-- cpd00263_c

rxn15554_c: cpd00094_c + cpd00117_c + cpd00229_c <-- cpd00263_c

rxn15555_c: cpd00009_c + cpd11791_c <=> cpd00089_c

rxn15556_c: cpd00009_c + cpd11746_c <=> cpd00089_c

rxn15557_c: cpd00026_c <=> cpd00014_c + cpd11746_c

rxn15558_c: cpd00037_c <=> cpd00014_c + cpd11683_c

rxn15559_c: cpd00031_c + cpd11746_c <=> cpd00315_c

rxn15561_c: cpd00026_c <=> cpd00014_c + cpd12148_c

rxn15562_c: cpd00387_c <=> cpd00008_c + cpd11735_c

rxn15563_c: cpd00009_c + cpd11735_c <=> cpd00089_c

rxn15564_c: cpd00026_c <=> cpd00014_c + cpd11735_c

rxn15565_c: cpd00076_c <=> cpd00082_c + cpd11735_c

rxn15569_c: cpd00076_c <=> cpd00082_c + cpd11658_c

rxn15572_c: cpd11735_c + cpd19001_c <=> cpd00179_c

rxn15573_c: cpd00472_c <=> cpd00014_c + cpd11686_c

rxn15574_c: cpd02560_c <=> cpd01904_c + cpd12511_c

rxn15575_c: cpd00001_c + cpd11746_c <=> cpd00190_c

rxn15576_c: cpd00001_c + cpd11791_c <=> cpd19001_c

rxn15578_c: cpd00001_c + cpd11683_c <=> cpd00122_c

rxn15579_c: cpd00001_c + cpd11683_c <=> cpd02416_c

rxn15580_c: cpd00001_c + cpd11683_c <=> cpd00067_c + cpd02217_c

rxn15581_c: cpd00163_c <=> cpd00014_c + cpd12085_c

rxn15589_c: cpd00001_c + cpd03611_c <=> cpd00013_c + 2.0 cpd11636_c

rxn15591_c: cpd11594_c <=> cpd00179_c + cpd11735_c

rxn15592_c: cpd00001_c + cpd11792_c <=> cpd11735_c

rxn15593_c: cpd11735_c <=> cpd11658_c

rxn15595_c: cpd11735_c <=> cpd11594_c

rxn15596_c: cpd00001_c + cpd11594_c <=> cpd19001_c

rxn15597_c: cpd00001_c + 2.0 cpd11746_c <=> cpd00158_c

rxn15598_c: cpd00001_c + cpd11686_c <=> cpd00280_c

rxn15599_c: cpd00001_c + cpd11686_c <=> cpd05262_c

rxn15600_c: cpd01487_c <=> cpd00001_c + cpd12085_c

rxn15601_c: cpd01422_c <=> cpd00001_c + cpd12085_c

rxn15603_c: cpd00001_c + cpd11791_c <=> cpd00190_c

rxn15605_c: cpd00276_c <=> cpd00001_c + cpd11741_c

rxn15606_c: cpd00001_c + cpd11741_c <=> cpd05263_c

rxn15608_c: cpd03747_c + cpd11610_c <=> cpd00001_c + cpd08735_c + cpd11609_c

rxn15609_c: cpd03749_c + cpd11610_c <=> cpd00001_c + cpd08735_c + cpd11609_c

rxn15637_c: cpd00554_c --> cpd08215_c

rxn15638_c: cpd19105_c <-- cpd09205_c

rxn15663_c: cpd00067_c + cpd00534_c + cpd11609_c + cpd19082_c <=> cpd01259_c + cpd11610_c

rxn15671_c: cpd00026_c + cpd01424_c --> cpd00014_c + cpd02485_c

rxn15672_c: cpd01052_c --> cpd01424_c

rxn15678_c: cpd00053_c + cpd00102_c + cpd00171_c <-- cpd00016_c + cpd00023_c

rxn15683_c: cpd00007_c + cpd03217_c + cpd11610_c <=> cpd00001_c + cpd11609_c + cpd14598_c

rxn15684_c: cpd11609_c + cpd14601_c <=> cpd11610_c + cpd14605_c

rxn15690_c: cpd05498_c --> cpd19170_c

rxn15691_c: cpd19170_c --> cpd14691_c

rxn15705_c: cpd00007_c + cpd03218_c + cpd11610_c <=> cpd00001_c + cpd11609_c + cpd19175_c

rxn15708_c: cpd00001_c + cpd00554_c + cpd11609_c <=> cpd01946_c + cpd11610_c

rxn15709_c: cpd00001_c + cpd11609_c + cpd16501_c <=> cpd01946_c + cpd11610_c

rxn15734_c: cpd03688_c <-- cpd19106_c

rxn15735_c: cpd03688_c <=> cpd09199_c

rxn15737_c: cpd16509_c <=> cpd05439_c

rxn15739_c: cpd00318_c <-- cpd05985_c

rxn15752_c: cpd03148_c <=> cpd03149_c

rxn15753_c: cpd00216_c <-- cpd17270_c

rxn15755_c: cpd17281_c <-- cpd17282_c

rxn15757_c: cpd17282_c <=> cpd11451_c

rxn15763_c: cpd01880_c --> cpd00486_c

rxn15765_c: cpd00359_c --> cpd00703_c

rxn15781_c: cpd00007_c + cpd12785_c <=> cpd17679_c

rxn15787_c: cpd06735_c <=> cpd01984_c

rxn15788_c: cpd03617_c --> cpd08447_c

rxn15797_c: cpd17841_c --> cpd17842_c

rxn15798_c: cpd17844_c --> cpd17845_c

rxn15799_c: cpd17840_c --> cpd17839_c

rxn15800_c: cpd17839_c --> cpd07329_c

rxn15818_c: cpd07333_c --> cpd00887_c

rxn15828_c: cpd03870_c --> cpd03873_c

rxn15830_c: cpd17929_c --> cpd03874_c

rxn15832_c: cpd17934_c <=> cpd17947_c

rxn15836_c: cpd01807_c <-- cpd17927_c

rxn15837_c: cpd17927_c <-- cpd17928_c

rxn15838_c: cpd02024_c <-- cpd17926_c

rxn15839_c: cpd02024_c <-- cpd17930_c

rxn15840_c: cpd02024_c <-- cpd17945_c

rxn15841_c: cpd02024_c <-- cpd17946_c

rxn15843_c: cpd17933_c <=> cpd17934_c

rxn15859_c: cpd00002_c + cpd00023_c + cpd11493_c <=> cpd00008_c + cpd00009_c + cpd17990_c

rxn15863_c: cpd01214_c + cpd17993_c <=> 4.0 cpd00067_c + cpd11493_c + cpd17989_c

rxn15869_c: cpd18012_c <-- cpd18013_c

rxn15870_c: cpd18013_c <-- cpd18014_c

rxn15871_c: cpd04025_c <-- cpd18015_c

rxn15888_c: cpd18051_c --> cpd18052_c

rxn15889_c: cpd18052_c <-- cpd18053_c

rxn15890_c: cpd18053_c --> cpd18054_c

rxn15892_c: cpd01420_c --> cpd18019_c

rxn15893_c: cpd18019_c <=> cpd18020_c

rxn15894_c: cpd18020_c --> cpd06086_c

rxn15896_c: cpd00546_c <-- cpd03135_c

rxn15911_c: cpd19401_c <-- cpd07679_c

rxn15913_c: cpd19402_c --> cpd19403_c

rxn15914_c: cpd19403_c --> cpd17543_c

rxn15915_c: cpd07679_c --> cpd19405_c

rxn15920_c: cpd07658_c <=> cpd01704_c

rxn15921_c: cpd16470_c <-- cpd17210_c

rxn15931_c: cpd01449_c <=> cpd19427_c

rxn15932_c: cpd00001_c + cpd00017_c + cpd19427_c <=> cpd00019_c + cpd19430_c

rxn15936_c: cpd16354_c <=> cpd01449_c

rxn15951_c: cpd00007_c + cpd01270_c --> cpd00001_c + cpd00236_c + cpd01997_c

rxn15955_c: cpd00020_c + cpd00153_c <-- cpd00011_c + cpd17457_c

rxn15956_c: cpd17457_c <-- cpd05216_c

rxn15957_c: 3.0 cpd00070_c + cpd00481_c <-- cpd19471_c

rxn15965_c: cpd00290_c <-- cpd00169_c

rxn15966_c: cpd19036_c <-- cpd00169_c

rxn15967_c: cpd09997_c --> cpd19483_c

rxn15969_c: cpd09919_c --> cpd19484_c

rxn15973_c: cpd08679_c --> cpd19487_c

rxn15975_c: cpd19488_c --> cpd08863_c

rxn15977_c: cpd19479_c --> cpd19489_c

rxn15979_c: cpd19490_c --> cpd06014_c

rxn15983_c: cpd21382_c <=> cpd17999_c

rxn16001_c: cpd19510_c <-- cpd00011_c

rxn16004_c: cpd08026_c <=> cpd00204_c

rxn16005_c: cpd08026_c --> cpd08027_c

rxn16006_c: cpd08026_c --> cpd00040_c

rxn16007_c: cpd08026_c --> cpd00047_c

rxn16008_c: cpd04167_c --> cpd10563_c

rxn16009_c: cpd04167_c --> cpd08026_c

rxn16012_c: cpd10563_c --> cpd08028_c

rxn16013_c: cpd00001_c + cpd04167_c <=> cpd00040_c + 3.0 cpd00099_c

rxn16014_c: 2.0 cpd00001_c + cpd04167_c <=> cpd00047_c + 3.0 cpd00099_c

rxn16015_c: cpd00460_c <-- cpd02246_c

rxn16018_c: cpd19514_c --> cpd00071_c

rxn16020_c: cpd08028_c --> cpd00011_c

rxn16043_c: cpd10218_c <=> cpd19535_c

rxn16050_c: cpd19559_c <=> cpd19560_c

rxn16058_c: cpd19560_c <=> cpd19561_c

rxn16059_c: cpd04031_c <-- cpd04057_c

rxn16060_c: cpd02048_c --> cpd19562_c

rxn16061_c: cpd04030_c <-- cpd19565_c

rxn16064_c: cpd19537_c <=> cpd19539_c

rxn16065_c: cpd19538_c <=> cpd19540_c

rxn16072_c: cpd00007_c + cpd04705_c <=> cpd19552_c

rxn16080_c: cpd19517_c <=> cpd19518_c

rxn16118_c: 8.0 cpd00067_c + 8.0 cpd00070_c + cpd11493_c <=> 8.0 cpd00010_c + 8.0 cpd00011_c + cpd13411_c

rxn16152_c: cpd11609_c + cpd19589_c <=> cpd03589_c + cpd11610_c

rxn16154_c: cpd19594_c <=> cpd00001_c + cpd19618_c

rxn16155_c: cpd00005_c + cpd00067_c + cpd19618_c <=> cpd00006_c + cpd19619_c

rxn16158_c: cpd00005_c + cpd00067_c + cpd19618_c <=> cpd00006_c + cpd19620_c

rxn16160_c: cpd19622_c <=> cpd00001_c + cpd20096_c

rxn16166_c: cpd19597_c <=> cpd19892_c

rxn16167_c: cpd19600_c <=> cpd09172_c

rxn16171_c: cpd19894_c --> cpd19895_c

rxn16174_c: cpd19898_c <-- cpd19900_c

rxn16177_c: cpd09177_c --> cpd19903_c

rxn16181_c: cpd19597_c <=> cpd19941_c

rxn16185_c: cpd00322_c + cpd19942_c --> cpd00001_c + cpd00011_c + cpd19943_c

rxn16187_c: cpd09155_c <-- cpd19945_c

rxn16188_c: cpd19945_c <-- cpd19946_c

rxn16189_c: cpd20040_c <-- cpd19947_c

rxn16190_c: cpd19947_c <-- cpd19948_c

rxn16191_c: cpd19601_c <=> cpd19972_c

rxn16196_c: cpd19602_c <=> cpd00067_c + cpd09119_c + cpd11493_c

rxn16197_c: cpd09132_c --> cpd09134_c

rxn16198_c: cpd19946_c --> cpd20040_c

rxn16200_c: cpd00067_c + cpd20059_c <=> cpd00150_c + cpd11616_c

rxn16201_c: cpd19948_c <-- cpd20085_c

rxn16202_c: cpd20085_c <-- cpd20084_c

rxn16203_c: cpd20084_c <-- cpd20083_c

rxn16219_c: cpd00002_c + 2.0 cpd00052_c + cpd12048_c <=> 3.0 cpd00012_c + cpd20345_c

rxn16220_c: cpd00052_c + cpd12048_c <=> cpd00012_c + cpd20338_c

rxn16266_c: cpd15157_c <=> cpd20855_c

rxn16267_c: cpd20828_c <=> cpd20856_c

rxn16274_c: cpd20821_c --> cpd20823_c

rxn16275_c: cpd20819_c --> cpd20823_c

rxn16276_c: cpd20832_c --> cpd20833_c

rxn16277_c: cpd20836_c --> cpd20833_c

rxn16287_c: cpd00005_c + 2.0 cpd00067_c + cpd11722_c <=> cpd00006_c + cpd12196_c

rxn16288_c: cpd00001_c + cpd12196_c <=> cpd00010_c + 2.0 cpd00067_c + cpd00487_c

rxn16295_c: cpd20867_c --> cpd20868_c

rxn16296_c: cpd20870_c --> cpd20871_c

rxn16297_c: cpd20877_c --> cpd20878_c

rxn16308_c: cpd11709_c <=> cpd00204_c + cpd11855_c

rxn16309_c: cpd11855_c <=> cpd11679_c

rxn16312_c: 2.0 cpd00005_c + 2.0 cpd00067_c + cpd12196_c <=> 2.0 cpd00006_c + cpd00010_c + cpd00195_c

rxn16325_c: cpd00047_c + cpd11609_c <=> cpd00011_c + cpd11610_c

rxn16326_c: cpd00003_c + cpd11493_c + cpd11709_c <=> cpd00004_c + cpd00067_c + cpd11628_c

rxn16327_c: cpd00006_c + cpd11493_c + cpd11709_c <=> cpd00005_c + cpd00067_c + cpd11628_c

rxn16338_c: cpd00567_c + cpd11609_c <=> cpd02235_c + cpd11610_c

rxn16361_c: cpd00007_c + cpd00013_c + cpd11610_c <=> cpd00001_c + cpd00165_c + cpd11609_c

rxn16395_c: cpd00212_c + cpd11609_c <=> cpd11610_c + cpd20927_c

rxn16439_c: cpd00002_c + cpd00039_c + cpd20969_c <=> cpd00001_c + cpd00012_c + cpd00018_c + cpd20970_c

rxn16446_c: cpd00007_c + cpd05523_c + cpd11610_c <=> cpd00001_c + cpd11609_c + cpd20979_c

rxn16447_c: cpd00007_c + cpd11610_c + cpd20980_c <=> cpd00001_c + cpd05876_c + cpd11609_c

rxn16502_c: cpd03215_c + 2.0 cpd11609_c <=> cpd03217_c + 2.0 cpd11610_c

rxn16514_c: 4.0 cpd00007_c + 4.0 cpd11610_c + cpd21041_c <=> 6.0 cpd00001_c + 4.0 cpd11609_c + cpd21042_c

rxn16537_c: cpd03211_c + 3.0 cpd11609_c <=> cpd03216_c + 3.0 cpd11610_c

rxn16538_c: cpd03211_c + cpd11609_c <=> cpd03206_c + cpd11610_c

rxn16539_c: cpd03211_c + 2.0 cpd11609_c <=> cpd03215_c + 2.0 cpd11610_c

rxn16549_c: cpd03211_c + 5.0 cpd11609_c <=> 5.0 cpd11610_c + cpd14598_c

rxn16562_c: cpd03211_c + 4.0 cpd11609_c <=> cpd03217_c + 4.0 cpd11610_c

rxn16568_c: cpd00001_c + 2.0 cpd20961_c <=> cpd20957_c + cpd21085_c

rxn16575_c: 2.0 cpd00007_c + 2.0 cpd11610_c + cpd14866_c <=> 3.0 cpd00001_c + 2.0 cpd11609_c + cpd21081_c

rxn16576_c: cpd00007_c + cpd11610_c + cpd21081_c <=> cpd00001_c + cpd11609_c + cpd14867_c

rxn16605_c: cpd14530_c --> cpd21113_c

rxn16606_c: cpd05719_c --> cpd21114_c

rxn16650_c: cpd00013_c + cpd00418_c <=> cpd00001_c + cpd00659_c

rxn16686_c: cpd00001_c + cpd21353_c <=> cpd11608_c + cpd12562_c

rxn16688_c: cpd12603_c <=> cpd20939_c

rxn16692_c: cpd08957_c <=> cpd21367_c

rxn16693_c: cpd21367_c <=> cpd21368_c

rxn16696_c: cpd21370_c <=> cpd21371_c

rxn16722_c: cpd00237_c + cpd11609_c <=> cpd11610_c + cpd21377_c

rxn16757_c: cpd21376_c --> cpd00237_c

rxn16828_c: cpd21482_c <-- cpd21486_c

rxn16840_c: cpd21470_c <=> cpd00009_c + 2.0 cpd11735_c

rxn16841_c: cpd00001_c + cpd11657_c <=> cpd00794_c

rxn16846_c: cpd11628_c + cpd22235_c <=> cpd11493_c + cpd27368_c

rxn16847_c: cpd11628_c + cpd22235_c <=> cpd11493_c + cpd27368_c

rxn16901_c: cpd00248_c + cpd28018_c <=> cpd00699_c + cpd28074_c

rxn16947_c: cpd00248_c + cpd28018_c <=> cpd00699_c + cpd28074_c

rxn17001_c: cpd00067_c + cpd11180_c + cpd28018_c <=> cpd02016_c + cpd28074_c

rxn17048_c: cpd22337_c + 2.0 cpd27814_c <=> cpd00001_c + cpd22318_c + cpd27930_c

rxn17065_c: cpd00007_c + cpd00024_c + cpd27033_c <=> cpd00011_c + cpd00036_c + cpd26968_c

rxn17115_c: cpd00007_c + cpd00160_c + cpd28078_c <=> cpd00001_c + cpd01342_c + cpd01585_c + cpd27753_c

rxn17148_c: cpd00001_c + cpd00452_c + 2.0 cpd28018_c <=> cpd11203_c + 2.0 cpd28074_c

rxn17224_c: cpd27072_c + 2.0 cpd27638_c <=> cpd00067_c + cpd22375_c + 2.0 cpd27640_c

rxn17227_c: cpd08441_c <=> cpd00389_c + cpd28082_c

rxn17277_c: 2.0 cpd00268_c + cpd28018_c <=> cpd01414_c + cpd28074_c

rxn17322_c: 2.0 cpd00268_c + cpd28018_c <=> cpd01414_c + cpd28074_c

rxn17379_c: cpd28293_c <-- cpd00014_c

rxn17380_c: cpd00001_c + cpd25349_c <=> 2.0 cpd00067_c + cpd25350_c

rxn17424_c: cpd00017_c + cpd28567_c <=> cpd00019_c + cpd28548_c

rxn17457_c: cpd11628_c + cpd28154_c <=> cpd11493_c + cpd26819_c

rxn17463_c: 3.0 cpd00070_c + cpd08876_c --> 4.0 cpd00010_c + 3.0 cpd00011_c + cpd08877_c

rxn17470_c: cpd00017_c + cpd11628_c <=> cpd00147_c + cpd11493_c + cpd22307_c

rxn17472_c: cpd00095_c + cpd22234_c <=> cpd00010_c + cpd24471_c

rxn17481_c: cpd27059_c + cpd27426_c <=> cpd00010_c + cpd27423_c

rxn17491_c: cpd00042_c <-- cpd00033_c

rxn17495_c: cpd24353_c + cpd27149_c <=> cpd26919_c + cpd27692_c

rxn17498_c: cpd00387_c + cpd27149_c <=> cpd00008_c + cpd25341_c

rxn17500_c: cpd22375_c + cpd28293_c <=> cpd00014_c + cpd00067_c + cpd22277_c

rxn17507_c: cpd00076_c <-- cpd19102_c

rxn17510_c: cpd24838_c --> cpd02229_c

rxn17526_c: cpd00037_c + cpd27097_c <=> cpd00014_c + cpd00067_c + cpd27564_c

rxn17529_c: cpd00037_c + cpd22406_c <=> cpd00014_c + cpd00067_c + cpd27555_c

rxn17530_c: cpd00037_c + cpd22406_c <=> cpd00014_c + cpd00067_c + cpd27556_c

rxn17531_c: cpd00043_c + cpd22406_c <=> cpd00014_c + cpd00067_c + cpd22256_c

rxn17532_c: cpd00037_c + cpd00067_c + cpd26919_c <=> cpd00014_c + cpd12587_c

rxn17542_c: cpd00175_c + cpd25353_c <=> cpd00014_c + cpd00067_c + cpd27133_c

rxn17543_c: cpd00175_c + cpd25353_c <=> cpd00014_c + cpd00067_c + cpd27133_c

rxn17547_c: cpd27033_c + cpd28293_c <=> cpd00014_c + cpd27032_c

rxn17557_c: cpd02560_c <-- cpd26842_c

rxn17565_c: cpd00144_c + cpd27262_c <=> cpd00014_c + cpd27263_c

rxn17566_c: cpd23744_c + cpd27627_c <=> cpd00031_c + 2.0 cpd00067_c + cpd27628_c

rxn17573_c: cpd00037_c + cpd27236_c <=> cpd00014_c + cpd00067_c + cpd27235_c

rxn17574_c: cpd00037_c + cpd27236_c <=> cpd00014_c + cpd00067_c + cpd27235_c

rxn17575_c: cpd00144_c + cpd27235_c <=> cpd00014_c + cpd27236_c

rxn17576_c: cpd00144_c + cpd27235_c <=> cpd00014_c + cpd27236_c

rxn17577_c: cpd00144_c + cpd27133_c <=> cpd00014_c + cpd25353_c

rxn17578_c: cpd00144_c + cpd27133_c <=> cpd00014_c + cpd25353_c

rxn17582_c: cpd27086_c + cpd28293_c <=> cpd00014_c + cpd27082_c

rxn17583_c: cpd22377_c + cpd28293_c --> cpd00014_c + cpd22376_c

rxn17587_c: cpd27653_c <=> cpd27685_c

rxn17588_c: cpd26833_c <=> cpd26834_c

rxn17592_c: cpd27191_c + cpd28293_c <=> cpd00014_c + cpd27162_c

rxn17594_c: cpd00043_c + cpd25355_c <=> cpd00014_c + cpd00067_c + cpd22406_c

rxn17597_c: cpd00472_c <-- cpd00014_c

rxn17598_c: cpd00043_c + cpd27415_c <=> cpd00014_c + cpd12553_c

rxn17602_c: cpd00043_c + cpd26685_c <=> cpd00014_c + cpd00067_c + cpd22221_c

rxn17604_c: cpd12183_c + cpd28293_c <=> cpd00014_c + cpd12563_c

rxn17605_c: cpd11720_c + cpd28293_c <=> cpd00014_c + cpd00067_c + cpd25083_c

rxn17606_c: cpd00037_c + cpd27415_c <=> cpd00014_c + cpd27565_c

rxn17607_c: cpd00037_c + cpd27415_c <=> cpd00014_c + cpd27565_c

rxn17611_c: cpd23744_c + cpd27627_c <=> cpd00031_c + 2.0 cpd00067_c + cpd27629_c

rxn17614_c: cpd00067_c + cpd11804_c + cpd28293_c <=> cpd00014_c + cpd12391_c

rxn17621_c: cpd00051_c + cpd27638_c <=> cpd00133_c + cpd24580_c

rxn17623_c: cpd27149_c + cpd37298_c <=> cpd12092_c + cpd26919_c

rxn17626_c: cpd00003_c + cpd27674_c <=> cpd00133_c + cpd22243_c

rxn17627_c: cpd00163_c + cpd27627_c <=> cpd00014_c + 2.0 cpd00067_c + cpd27630_c

rxn17628_c: cpd00163_c + cpd27627_c <=> cpd00014_c + 2.0 cpd00067_c + cpd27630_c

rxn17633_c: cpd00112_c + cpd22425_c <=> cpd00046_c + cpd00067_c + cpd02982_c

rxn17635_c: cpd00112_c + cpd22406_c <=> cpd00046_c + cpd02987_c

rxn17638_c: cpd00113_c --> cpd00012_c

rxn17647_c: 2.0 cpd00289_c + cpd27814_c <=> 2.0 cpd00012_c + 2.0 cpd28134_c

rxn17673_c: cpd00003_c + cpd21917_c <=> cpd00001_c + cpd00133_c + cpd21093_c + cpd28596_c

rxn17682_c: cpd00002_c + cpd27149_c <=> cpd00008_c + cpd27148_c

rxn17702_c: cpd11713_c <-- cpd26919_c

rxn17708_c: cpd00008_c + cpd22358_c <=> cpd00009_c + cpd22241_c

rxn17709_c: cpd00067_c + cpd22358_c + cpd27685_c <=> cpd00009_c + cpd11898_c

rxn17714_c: cpd00009_c <=> cpd27685_c

rxn17721_c: cpd02086_c + cpd28293_c <=> cpd00067_c + cpd00091_c + cpd02831_c

rxn17732_c: 2.0 cpd00017_e + cpd00084_e + cpd01311_e + cpd28207_e <=> cpd00035_c + 2.0 cpd00060_c + cpd00104_c + 2.0 cpd03091_c + cpd28307_c

rxn17757_c: cpd11836_c <=> cpd00001_c + cpd11728_c

rxn17770_c: cpd11492_c + cpd28167_c <=> cpd00011_c + cpd11493_c + cpd11726_c

rxn17771_c: cpd11492_c + cpd28167_c <=> cpd00011_c + cpd11493_c + cpd11726_c

rxn17776_c: cpd00001_c + cpd25348_c <=> cpd00029_c + cpd00067_c + cpd25347_c

rxn17781_c: cpd00001_c --> cpd00797_c

rxn17783_c: cpd21994_c <=> cpd22016_c + cpd27058_c

rxn17791_c: cpd00001_c <=> cpd27686_c

rxn17792_c: <=> cpd27686_c

rxn17797_c: cpd00001_c + cpd27721_c <=> cpd27686_c

rxn17799_c: cpd28576_c <=> cpd27686_c + cpd28567_c

rxn17800_c: cpd00001_c + cpd28041_c <=> cpd22057_c

rxn17802_c: cpd00001_c <=> cpd22057_c

rxn17816_c: cpd00002_c + cpd26896_c <=> cpd00008_c + cpd00009_c + cpd26982_c

rxn17817_c: cpd00001_c + cpd26896_c <=> cpd26982_c

rxn17825_c: cpd00001_c + cpd28540_c <=> cpd28108_c

rxn17836_c: cpd00001_c + cpd27889_c <=> cpd27721_c

rxn17837_c: cpd28576_c <=> cpd28111_c + cpd28567_c

rxn17839_c: cpd00001_c + cpd28041_c <=> cpd22057_c + cpd22082_c

rxn17842_c: cpd00001_c + cpd28041_c <=> cpd22053_c + cpd22082_c

rxn17846_c: cpd00001_c + cpd27889_c <=> cpd00988_c

rxn17849_c: cpd00001_c + cpd27148_c <=> cpd00009_c + cpd27149_c

rxn17864_c: cpd00001_c + cpd26763_c <=> cpd00067_c + cpd27686_c

rxn17889_c: cpd23691_c <=> cpd00048_c + cpd22518_c

rxn17892_c: cpd00001_c + cpd27491_c <=> cpd24024_c

rxn17893_c: cpd00001_c + cpd21810_c <=> cpd19009_c + cpd24095_c

rxn17902_c: 2.0 cpd00001_c + cpd27493_c <=> 2.0 cpd19009_c + cpd27492_c

rxn17904_c: cpd00001_c --> cpd01262_c

rxn17906_c: cpd00001_c + cpd27897_c <=> cpd01384_c

rxn17910_c: cpd00001_c + cpd27153_c <=> cpd23933_c + cpd27152_c

rxn17912_c: cpd26712_c <=> 2.0 cpd26713_c

rxn17914_c: cpd00001_c + cpd27994_c <=> cpd00537_c

rxn17915_c: cpd00001_c + cpd27173_c <=> cpd27172_c

rxn17916_c: cpd00001_c + cpd27173_c <=> cpd27172_c

rxn17917_c: cpd00001_c --> cpd27437_c

rxn17919_c: cpd00001_c + cpd22519_c <=> cpd26711_c

rxn17920_c: cpd00001_c + cpd22519_c <=> cpd26711_c

rxn17921_c: cpd00001_c + cpd22519_c <=> cpd26711_c

rxn17922_c: cpd27433_c <=> cpd00794_c + cpd27490_c

rxn17925_c: cpd00001_c + cpd22010_c <=> cpd00875_c + cpd27415_c

rxn17926_c: cpd00001_c + cpd22454_c <=> cpd00709_c + cpd28035_c

rxn17930_c: cpd00001_c + cpd28355_c <=> cpd28356_c

rxn17932_c: --> cpd19102_c

rxn17933_c: cpd00001_c --> cpd19102_c

rxn17935_c: cpd00001_c + cpd28500_c <=> cpd23882_c + cpd23883_c

rxn17936_c: cpd00001_c + cpd22315_c <=> cpd25500_c

rxn17937_c: cpd00001_c + cpd22315_c <=> cpd25500_c

rxn17938_c: cpd00001_c + cpd27668_c <=> cpd23867_c + cpd23868_c + cpd26818_c

rxn17940_c: cpd00001_c + cpd27397_c <=> cpd23886_c + cpd23887_c

rxn17941_c: cpd00001_c + cpd27397_c <=> cpd23886_c + cpd23887_c

rxn17942_c: --> cpd27437_c

rxn17944_c: --> cpd00276_c

rxn17945_c: cpd00001_c + cpd27843_c <=> cpd00122_c + cpd27842_c

rxn17951_c: cpd00001_c + cpd22357_c <=> cpd19009_c + cpd28035_c

rxn17952_c: cpd00001_c + cpd22450_c <=> cpd01490_c + cpd28035_c

rxn17956_c: cpd00001_c + cpd28281_c <-- cpd00190_c + cpd25936_c

rxn17957_c: cpd00001_c + cpd27261_c <=> cpd11691_c

rxn17958_c: cpd00001_c + cpd27261_c <=> cpd11691_c

rxn17959_c: cpd00001_c + cpd27261_c <=> cpd23482_c

rxn17960_c: cpd00001_c + cpd21753_c <=> cpd01422_c

rxn17961_c: cpd00001_c + cpd22451_c <=> cpd01421_c + cpd28035_c

rxn17962_c: cpd00001_c + cpd22361_c <=> cpd01627_c + cpd28035_c

rxn17963_c: cpd00001_c + cpd22456_c <=> cpd01562_c + cpd28035_c

rxn17968_c: cpd00001_c + cpd22268_c <=> cpd23469_c + cpd28035_c

rxn17969_c: cpd00001_c + cpd27577_c <=> cpd27613_c + cpd28035_c

rxn17970_c: cpd00001_c + cpd27577_c <=> cpd27613_c + cpd28035_c

rxn17971_c: cpd00001_c + cpd28380_c <=> cpd27607_c + cpd28035_c

rxn17972_c: cpd00001_c + cpd21759_c <=> cpd01698_c

rxn17973_c: cpd00001_c + cpd21759_c <=> cpd01698_c

rxn17976_c: cpd00001_c + cpd27994_c <=> cpd23936_c

rxn17977_c: cpd00001_c --> cpd19001_c

rxn17979_c: cpd00001_c --> cpd01399_c

rxn17980_c: cpd27669_c <=> cpd01074_c + cpd24014_c

rxn17981_c: cpd00001_c + cpd27401_c <=> cpd01189_c

rxn17983_c: cpd00001_c --> cpd00179_c

rxn17987_c: cpd00001_c + cpd21749_c <=> cpd01422_c

rxn17989_c: --> cpd00190_c

rxn17991_c: cpd00001_c + cpd26849_c <=> cpd23457_c + cpd26949_c

rxn17994_c: cpd21757_c <=> cpd21753_c

rxn17995_c: cpd21757_c <=> cpd21753_c

rxn17996_c: --> cpd30321_c

rxn17997_c: cpd00001_c + cpd22315_c <=> cpd27668_c

rxn17998_c: cpd00001_c + cpd22315_c <=> cpd27668_c

rxn17999_c: cpd00001_c --> cpd01532_c

rxn18000_c: cpd00001_c + cpd28502_c <=> cpd23880_c + cpd23881_c

rxn18001_c: --> cpd19001_c

rxn18002_c: cpd00001_c --> cpd24020_c

rxn18004_c: cpd00001_c + cpd26683_c <=> cpd03845_c

rxn18011_c: cpd00001_c --> cpd01329_c

rxn18012_c: cpd00001_c + cpd27354_c <=> cpd21759_c

rxn18013_c: cpd00001_c + cpd27354_c <=> cpd21759_c

rxn18015_c: cpd00001_c + cpd26902_c <=> cpd00226_c + cpd22278_c

rxn18016_c: cpd00001_c + cpd26904_c <=> cpd26908_c

rxn18017_c: cpd00001_c + cpd26911_c <=> cpd00677_c + cpd26896_c

rxn18019_c: cpd00001_c + cpd26909_c <=> cpd23830_c + cpd26896_c

rxn18020_c: cpd22243_c <=> cpd00251_c + cpd27674_c

rxn18022_c: cpd00001_c + cpd02749_c <=> cpd00507_c + cpd22319_c

rxn18023_c: cpd00001_c + cpd21769_c <=> cpd00908_c + cpd22319_c

rxn18026_c: cpd00001_c <=> cpd22369_c

rxn18027_c: <=> cpd22369_c

rxn18028_c: cpd00001_c + cpd27149_c <=> cpd22369_c + cpd27839_c

rxn18029_c: cpd27149_c <=> 2.0 cpd27839_c

rxn18030_c: cpd00001_c + cpd27960_c <=> cpd00065_c + cpd00067_c + cpd27839_c

rxn18031_c: cpd00001_c + cpd27959_c <=> cpd00060_c + cpd00067_c + cpd27839_c

rxn18032_c: cpd00001_c + cpd27958_c <=> cpd00067_c + cpd26810_c + cpd27839_c

rxn18034_c: <=> cpd22353_c

rxn18050_c: cpd27839_c <=> cpd26871_c

rxn18052_c: cpd00001_c + cpd27149_c <=> cpd26871_c + cpd27839_c

rxn18053_c: cpd27841_c <=> cpd27839_c + cpd27922_c

rxn18058_c: cpd00001_c <-- cpd22632_c

rxn18062_c: cpd00001_c <=> cpd22440_c

rxn18063_c: cpd00001_c <=> cpd22440_c

rxn18065_c: cpd00001_c <=> cpd22353_c

rxn18068_c: cpd00001_c <=> cpd22393_c

rxn18069_c: cpd00001_c + cpd27927_c <=> cpd27344_c + cpd27839_c

rxn18071_c: cpd00001_c --> cpd00023_c

rxn18074_c: cpd00001_c --> cpd19181_c

rxn18076_c: cpd00001_c <-- cpd02012_c

rxn18077_c: cpd27966_c <=> cpd00023_c + cpd27839_c

rxn18078_c: cpd00001_c + cpd26680_c <=> cpd27839_c

rxn18079_c: cpd27149_c <=> 2.0 cpd27839_c

rxn18092_c: cpd00001_c + cpd26748_c <=> cpd27839_c

rxn18103_c: cpd27049_c <=> cpd27839_c

rxn18104_c: cpd27068_c <=> cpd27839_c

rxn18113_c: cpd27149_c <=> 2.0 cpd27839_c

rxn18121_c: cpd27149_c <=> 2.0 cpd27839_c

rxn18129_c: cpd00067_c <=> cpd27839_c

rxn18134_c: cpd27149_c <=> cpd27839_c

rxn18139_c: cpd00001_c + cpd26680_c <=> 2.0 cpd27839_c

rxn18146_c: cpd26748_c <=> cpd27839_c

rxn18147_c: cpd00001_c + cpd27843_c <=> cpd01757_c + cpd27839_c

rxn18148_c: cpd00001_c + cpd27149_c <=> cpd27839_c

rxn18150_c: cpd00001_c + cpd27695_c <=> cpd27839_c

rxn18155_c: cpd26748_c <=> 2.0 cpd27839_c

rxn18158_c: cpd00001_c + cpd27149_c <=> 2.0 cpd27839_c

rxn18161_c: cpd00001_c + cpd27843_c <=> cpd01757_c + cpd27839_c

rxn18163_c: cpd00001_c + cpd24362_c <=> cpd00023_c + cpd27422_c

rxn18164_c: cpd00001_c + cpd24588_c <=> cpd00162_c + cpd27422_c

rxn18186_c: cpd00001_c + cpd27706_c <=> 2.0 cpd00067_c + cpd12357_c + cpd26919_c

rxn18672_c: cpd00001_e + cpd00002_c + cpd24099_e <=> cpd00008_c + cpd00009_c + cpd00067_c + cpd24099_c

rxn18938_c: <=> cpd23672_c

rxn18940_c: cpd28348_c <=> cpd23674_c + cpd23675_c

rxn18941_c: cpd22355_c <=> cpd00190_c + cpd03896_c

rxn18942_c: <=> cpd23677_c

rxn18944_c: <=> cpd26852_c

rxn18946_c: --> cpd22255_c

rxn18947_c: --> cpd26851_c

rxn18948_c: cpd26950_c <=> cpd02956_c

rxn18949_c: <=> cpd22911_c

rxn18950_c: cpd26719_c <=> cpd02956_c

rxn18951_c: --> cpd02956_c

rxn18952_c: --> cpd23426_c

rxn18954_c: cpd27209_c <=> cpd27722_c

rxn18958_c: cpd26908_c <=> 2.0 cpd00067_c + cpd22083_c + cpd22161_c

rxn18998_c: cpd00002_c + cpd26675_c + cpd27149_c <=> cpd00012_c + cpd00018_c + cpd22309_c

rxn19006_c: cpd00002_c + cpd00041_c + cpd26760_c <=> cpd00008_c + cpd00009_c + cpd26761_c

rxn19007_c: cpd00002_c + cpd00051_c + cpd26761_c <=> cpd00008_c + cpd00009_c + cpd26760_c

rxn19014_c: cpd00002_c + cpd00053_c + cpd27376_c <=> cpd00008_c + cpd00009_c + cpd00023_c + cpd26693_c

rxn19022_c: cpd01233_c + cpd02879_c <=> cpd00067_c + cpd00096_c + cpd22227_c

rxn19023_c: cpd00029_c + cpd28168_c <=> cpd00022_c + cpd27058_c

rxn19043_c: cpd00006_c + cpd22234_c <=> cpd00005_c + cpd00067_c + cpd26839_c

rxn19049_c: cpd00007_c + cpd22234_c <=> cpd00025_c + cpd28232_c

rxn19051_c: cpd00007_c + cpd22234_c <=> cpd00025_c + cpd28232_c

rxn19053_c: cpd00002_c + cpd11493_c + cpd27424_c <=> cpd00012_c + cpd00018_c + cpd27419_c

rxn19066_c: cpd11628_c + cpd21851_c <=> cpd11493_c + cpd27339_c

rxn19067_c: cpd11628_c + cpd21851_c <=> cpd11493_c + cpd27339_c

rxn19078_c: cpd00001_c + cpd22245_c <=> cpd00018_c + cpd22358_c

rxn19083_c: cpd00001_c + cpd27924_c <=> cpd00035_c + cpd27839_c

rxn19088_c: cpd22318_c + cpd27638_c <=> cpd00067_c + cpd22319_c + cpd27640_c

rxn19096_c: cpd00006_c + cpd22318_c <=> cpd00005_c + cpd00067_c + cpd22319_c

rxn19125_c: cpd00003_c + cpd26809_c <=> cpd00004_c + cpd00067_c + cpd26808_c

rxn19138_c: cpd21766_c + cpd27426_c <=> cpd00067_c + cpd21768_c + cpd27422_c

rxn19146_c: cpd00001_c --> cpd15495_c

rxn19153_c: cpd00017_c + cpd22346_c <=> cpd00019_c + cpd12107_c

rxn19162_c: cpd00001_c + cpd27606_c <=> cpd27605_c + cpd28567_c

rxn19172_c: cpd00006_c + 2.0 cpd00423_c <=> cpd00005_c + 2.0 cpd00730_c

rxn19173_c: cpd00003_c + 2.0 cpd00423_c <=> cpd00004_c + 2.0 cpd00730_c

rxn19183_c: cpd26689_c + cpd27149_c <=> cpd22281_c + cpd27935_c

rxn19185_c: cpd00006_c + cpd22398_c <=> cpd00005_c + cpd00067_c + cpd22399_c

rxn19186_c: cpd00003_c + cpd22398_c <=> cpd00004_c + cpd00067_c + cpd22399_c

rxn19194_c: cpd00044_c + cpd22398_c <=> cpd00045_c + cpd22404_c

rxn19195_c: 5.0 cpd00067_c + cpd22400_c + cpd28293_c <=> cpd00014_c + cpd02011_c

rxn19196_c: cpd00022_c + 5.0 cpd00067_c + cpd22400_c <=> cpd00010_c + cpd01676_c

rxn19197_c: cpd00044_c + cpd22346_c <=> cpd00045_c + cpd28201_c

rxn19200_c: cpd00001_c + cpd22401_c <=> cpd22398_c + cpd26962_c

rxn19201_c: cpd00001_c + cpd27853_c <=> cpd00029_c + cpd00067_c + cpd22398_c

rxn19255_c: cpd00001_c <=> cpd27344_c

rxn19279_c: cpd00009_c + cpd26683_c <=> cpd00089_c + cpd21756_c

rxn19280_c: cpd00315_c + cpd22518_c <=> cpd00031_c + cpd21756_c

rxn19281_c: cpd00001_c + cpd26685_c <=> cpd00267_c + cpd26675_c

rxn19284_c: cpd00067_c + cpd26685_c + cpd28293_c <=> cpd00014_c + cpd27168_c

rxn19285_c: cpd00002_c + cpd26685_c <=> cpd00008_c + cpd00067_c + cpd01899_c

rxn19287_c: cpd26687_c <=> cpd27033_c

rxn19293_c: cpd00001_c + cpd22519_c <=> cpd00029_c + cpd26712_c

rxn19295_c: cpd00001_c + cpd22519_c <=> cpd00029_c + cpd26712_c

rxn19302_c: cpd00001_c + cpd01236_c <=> cpd00999_c + cpd01449_c

rxn19311_c: cpd00006_c + cpd22234_c <=> cpd00005_c + cpd00067_c + cpd22523_c

rxn19354_c: cpd00007_c + 2.0 cpd00067_c + cpd26793_c <=> cpd00001_c + cpd26792_c

rxn19446_c: cpd00115_c + cpd26946_c <=> cpd09118_c + cpd26896_c

rxn19460_c: cpd26891_c <=> cpd26888_c

rxn19632_c: cpd26941_c <=> cpd00012_c

rxn19633_c: cpd27687_c <=> cpd00012_c

rxn19634_c: cpd00002_c + cpd26848_c <=> cpd00012_c + cpd00018_c

rxn19635_c: cpd00003_c + cpd26896_c <=> cpd00018_c + cpd00355_c

rxn19638_c: cpd00134_c + cpd26918_c <=> cpd00010_c + cpd12128_c

rxn19649_c: cpd00001_c + cpd12434_c <=> cpd12234_c + cpd26685_c

rxn19650_c: cpd00001_c + cpd27899_c <=> cpd27705_c

rxn19651_c: cpd00003_c + cpd28167_c <=> cpd00004_c + cpd00067_c + cpd11728_c

rxn19652_c: cpd00003_c + cpd28167_c <=> cpd00004_c + cpd00067_c + cpd11728_c

rxn19653_c: cpd00006_c + cpd28167_c <=> cpd00005_c + cpd00067_c + cpd11728_c

rxn19692_c: cpd00017_c + cpd27058_c <=> cpd00019_c + cpd27057_c

rxn19693_c: 2.0 cpd00005_c + cpd00022_c + 2.0 cpd00067_c + cpd00070_c <=> 2.0 cpd00006_c + 2.0 cpd00010_c + cpd00011_c + cpd27424_c

rxn19694_c: 2.0 cpd00004_c + 2.0 cpd00005_c + cpd00022_c + 4.0 cpd00067_c + cpd00070_c <=> 2.0 cpd00003_c + 2.0 cpd00006_c + cpd00010_c + cpd00011_c + cpd27059_c

rxn19698_c: cpd00131_c + 9.0 cpd00239_c + cpd00528_c + cpd00919_c + 7.0 cpd10515_c <=> cpd00013_c + 6.0 cpd00067_c + cpd24467_c

rxn19712_c: cpd00002_c + cpd00023_c <-- cpd00008_c + cpd00009_c

rxn19713_c: cpd00002_c + cpd00023_c <-- cpd00008_c + cpd00009_c

rxn19749_c: cpd22406_c + cpd27127_c <=> cpd00031_c + cpd00067_c + cpd25354_c

rxn19750_c: cpd00001_c + cpd22221_c <=> cpd00709_c + cpd26685_c

rxn19758_c: cpd22140_c + cpd22369_c <=> cpd22139_c + cpd27839_c

rxn19759_c: cpd22140_c + cpd22369_c <=> cpd22139_c + cpd27839_c

rxn19817_c: cpd00001_c + cpd27169_c <=> cpd00190_c + cpd26685_c

rxn19847_c: cpd00001_c + cpd27837_c <=> cpd00033_c + cpd27839_c

rxn19852_c: cpd00007_c + cpd12184_c + cpd28214_c <=> cpd00001_c + cpd12365_c + cpd22433_c

rxn19853_c: cpd00007_c + cpd12184_c + cpd28214_c <=> cpd00001_c + cpd12365_c + cpd22433_c

rxn19859_c: cpd21754_c <=> cpd27186_c

rxn19862_c: cpd00387_c <-- cpd00008_c

rxn19864_c: cpd00009_c + cpd27186_c <=> cpd00089_c + cpd26551_c

rxn19865_c: cpd00134_c + cpd27188_c <=> cpd00001_c + cpd00010_c + cpd27617_c

rxn19869_c: cpd00009_c + cpd27186_c <=> cpd00089_c + cpd01399_c

rxn19906_c: cpd27261_c <=> cpd00958_c

rxn19938_c: cpd27490_c <=> cpd01399_c

rxn19947_c: cpd27317_c + cpd28293_c <=> cpd00014_c + cpd27316_c

rxn19960_c: cpd00044_c + cpd27329_c <=> cpd00045_c + cpd27330_c

rxn19961_c: cpd00022_c + cpd28168_c <=> cpd00010_c + cpd22026_c

rxn19966_c: cpd00022_c + cpd28168_c <=> cpd00010_c + cpd22026_c

rxn19978_c: cpd00022_c + cpd28168_c <=> cpd00010_c + cpd22026_c

rxn20030_c: cpd26698_c + cpd27149_c <=> cpd27377_c + cpd28567_c

rxn20033_c: cpd00076_c <-- cpd00190_c

rxn20050_c: cpd22234_c + cpd27005_c <=> cpd26839_c + cpd27006_c

rxn20051_c: cpd00001_c + 2.0 cpd00003_c + cpd27426_c <=> 2.0 cpd00004_c + 3.0 cpd00067_c + cpd27422_c

rxn20053_c: cpd00001_c + cpd00003_c + cpd27421_c <=> cpd00004_c + 2.0 cpd00067_c + cpd27422_c

rxn20054_c: cpd00006_c + cpd00010_c + cpd27421_c <=> cpd00005_c + cpd00067_c + cpd27059_c

rxn20061_c: cpd00001_c + cpd27927_c <=> cpd22440_c + cpd27839_c

rxn20069_c: cpd00130_c + cpd28018_c <=> cpd00032_c + cpd28074_c

rxn20112_c: cpd00130_c + cpd28018_c <=> cpd00032_c + cpd28074_c

rxn20213_c: cpd00038_c + cpd25368_c <=> cpd00012_c + cpd00067_c + cpd25369_c

rxn20218_c: 2.0 cpd00005_c + 2.0 cpd00067_c + cpd00891_c + cpd22234_c <=> 2.0 cpd00006_c + cpd00010_c + cpd00011_c + cpd24481_c

rxn20264_c: cpd00001_c + cpd27638_c <=> cpd00133_c + cpd00251_c

rxn20277_c: cpd00209_c + cpd28079_c <=> cpd00075_c + cpd27754_c

rxn20278_c: cpd00209_c + cpd28079_c <=> cpd00075_c + cpd27754_c

rxn20284_c: cpd00067_c + cpd22326_c <=> cpd00001_c + cpd27670_c

rxn20291_c: cpd00067_c + cpd27640_c + cpd28018_c <=> cpd27638_c + cpd28074_c

rxn20367_c: cpd00067_c + cpd27640_c + cpd28018_c <=> cpd27638_c + cpd28074_c

rxn20464_c: cpd26942_c + cpd27689_c <=> cpd26940_c + cpd27688_c

rxn20466_c: cpd00001_c + cpd27689_c <=> cpd00009_c + cpd27688_c

rxn20467_c: cpd00001_c + cpd26971_c <=> 2.0 cpd00067_c + 2.0 cpd27686_c

rxn20468_c: cpd00002_c + cpd27686_c <=> cpd00018_c + cpd00067_c + cpd02634_c

rxn20505_c: cpd00001_c + cpd27835_c <=> cpd00116_c + cpd27777_c

rxn20513_c: cpd00001_c + cpd27939_c <=> cpd00013_c + cpd28372_c

rxn20514_c: cpd00001_c + cpd27925_c <=> cpd27120_c + cpd27839_c

rxn20515_c: cpd24458_c <=> cpd00040_c + cpd00067_c + cpd12041_c

rxn20516_c: cpd00007_c + cpd00059_c + cpd27933_c <=> cpd00001_c + cpd00335_c + cpd12288_c

rxn20520_c: cpd00025_c + cpd27851_c <=> 2.0 cpd00001_c + 2.0 cpd27852_c

rxn20521_c: cpd00025_c + cpd27851_c <=> 2.0 cpd00001_c + 2.0 cpd27852_c

rxn20524_c: cpd27340_c + cpd27442_c <=> cpd26855_c + cpd27441_c

rxn20525_c: cpd27340_c + cpd27442_c <=> cpd26855_c + cpd27441_c

rxn20535_c: cpd22398_c + cpd28293_c <=> cpd00014_c + cpd22403_c

rxn20610_c: cpd27809_c + cpd28293_c <=> cpd00014_c + cpd00067_c + cpd12607_c

rxn20642_c: cpd00009_c + cpd28004_c <=> cpd00475_c + cpd28002_c

rxn20657_c: cpd00005_c + cpd28018_c <=> cpd00006_c + cpd28170_c

rxn20667_c: cpd27114_c + cpd28082_c <=> cpd17456_c + cpd27757_c

rxn20705_c: cpd00102_c + cpd27757_c <=> cpd00169_c + cpd28082_c

rxn20773_c: cpd00001_c + cpd00003_c + cpd26664_c <=> cpd00004_c + 4.0 cpd00067_c + cpd00577_c

rxn20786_c: cpd00001_c + cpd28004_c <=> cpd28002_c + cpd28037_c

rxn20790_c: cpd00002_c + cpd28111_c <=> cpd00012_c + cpd00018_c

rxn20801_c: 3.0 cpd00067_c + cpd22805_c <-- cpd22806_c

rxn20805_c: cpd02041_c + cpd22809_c <-- cpd00012_c + cpd22810_c

rxn20883_c: cpd22334_c <=> cpd28169_c

rxn20884_c: cpd22334_c <=> cpd28169_c

rxn20889_c: cpd28326_c <=> cpd00204_c + cpd22334_c

rxn20890_c: cpd00070_c + cpd27059_c <=> cpd00010_c + cpd00011_c + cpd28320_c

rxn20934_c: cpd00080_c + cpd11628_c <=> cpd11493_c + cpd22235_c

rxn20935_c: cpd00080_c + cpd11628_c <=> cpd11493_c + cpd22235_c

rxn20969_c: cpd27373_c --> cpd22933_c

rxn20970_c: cpd01621_c --> cpd22934_c

rxn20971_c: cpd27373_c <-- cpd22935_c

rxn20972_c: cpd01621_c <-- cpd22936_c

rxn20996_c: 2.0 cpd00007_c + cpd21002_c <=> cpd06516_c

rxn21037_c: cpd22084_c + cpd22969_c <=> cpd00001_c + cpd25926_c

rxn21041_c: cpd00001_c + cpd03477_c + cpd22973_c <=> cpd00007_c + cpd00011_c + cpd22974_c + cpd27932_c

rxn21046_c: cpd22650_c + cpd22673_c <=> 2.0 cpd00067_c + cpd27774_c

rxn21050_c: cpd22978_c <=> cpd22650_c

rxn21053_c: 3.0 cpd00067_c + cpd22375_c <=> cpd22673_c

rxn21084_c: cpd22994_c + cpd26821_c <-- cpd22998_c

rxn21091_c: cpd01048_c + cpd28060_c <=> cpd04098_c + cpd27735_c

rxn21098_c: cpd22996_c <-- cpd07203_c

rxn21100_c: cpd22997_c <-- cpd07203_c

rxn21101_c: cpd07203_c --> cpd07194_c

rxn21106_c: cpd00043_c + cpd27596_c <=> cpd00014_c + cpd27137_c

rxn21126_c: cpd27047_c <=> cpd27839_c

rxn21130_c: cpd09115_c --> cpd07276_c

rxn21155_c: cpd01870_c <-- cpd23081_c

rxn21164_c: cpd02193_c --> 2.0 cpd00001_c + cpd00011_c + cpd16527_c

rxn21166_c: cpd16527_c <-- cpd01304_c

rxn21167_c: cpd00044_c + cpd26627_c <=> cpd00045_c + cpd21985_c

rxn21171_c: cpd00044_c + cpd23096_c <=> cpd00045_c + cpd21986_c

rxn21173_c: cpd00044_c + cpd05719_c <=> cpd00045_c + cpd22473_c

rxn21174_c: cpd00044_c + cpd14530_c <=> cpd00045_c + cpd26681_c

rxn21175_c: cpd23098_c <-- cpd03312_c

rxn21176_c: cpd00044_c + cpd07933_c <=> cpd00045_c + cpd21977_c

rxn21177_c: cpd00044_c + cpd23100_c <=> cpd00045_c + cpd21978_c

rxn21288_c: cpd00161_c + cpd23154_c + cpd28331_c <=> 2.0 cpd00001_c + cpd23152_c + cpd28330_c

rxn21289_c: cpd02142_c + cpd23154_c <=> cpd00001_c + cpd23153_c + cpd28330_c

rxn21290_c: cpd02494_c + cpd28330_c <=> cpd00018_c + cpd23154_c

rxn21304_c: cpd23161_c --> cpd23162_c

rxn21305_c: cpd23162_c --> cpd23163_c

rxn21338_c: cpd03389_c <=> cpd23188_c

rxn21339_c: cpd03163_c <=> cpd01667_c

rxn21340_c: cpd03389_c <=> cpd03163_c

rxn21363_c: cpd03390_c <=> cpd23188_c

rxn21364_c: cpd03391_c <=> cpd01667_c

rxn21365_c: 3.0 cpd00001_c + 2.0 cpd23316_c --> 4.0 cpd00117_c + cpd02229_c + cpd23307_c

rxn21366_c: 3.0 cpd00001_c + 2.0 cpd23316_c --> 4.0 cpd00117_c + cpd02229_c + cpd23307_c

rxn21376_c: cpd02338_c + cpd21758_c <-- cpd00014_c + cpd21760_c

rxn21394_c: 3.0 cpd15942_c + cpd23216_c <-- 3.0 cpd01904_c + cpd23214_c

rxn21395_c: 3.0 cpd15942_c + cpd23216_c <-- 3.0 cpd01904_c + cpd23214_c

rxn21396_c: 6.0 cpd15942_c + cpd23219_c <-- 6.0 cpd01904_c + cpd23218_c

rxn21397_c: 31.0 cpd15942_c + cpd23214_c <-- 31.0 cpd01904_c + cpd23219_c

rxn21399_c: cpd00007_c + cpd28523_c <=> 2.0 cpd00048_c + 2.0 cpd00067_c + cpd28502_c

rxn21403_c: cpd00005_c + 3.0 cpd00017_c + 8.0 cpd00067_c + cpd00070_c + 3.0 cpd00084_c + cpd23224_c --> 8.0 cpd00001_c + cpd00006_c + cpd00010_c + cpd00018_c + 3.0 cpd00019_c + cpd08829_c

rxn21420_c: cpd00007_c + cpd23235_c <=> cpd00001_c + cpd00067_c + cpd11359_c

rxn21426_c: cpd00001_c + cpd00002_c + cpd27149_c <=> cpd00008_c + cpd00009_c + 2.0 cpd27839_c

rxn21436_c: cpd00472_c <-- cpd02497_c

rxn21476_c: cpd00013_c + cpd02364_c + cpd27640_c <=> cpd00051_c + cpd27638_c

rxn21487_c: cpd23056_c <=> cpd23055_c

rxn21495_c: cpd07306_c --> cpd07421_c

rxn21496_c: cpd04758_c --> cpd07350_c

rxn21502_c: cpd00536_c <-- cpd16341_c

rxn21505_c: cpd23279_c <-- cpd23285_c

rxn21508_c: cpd23286_c <-- cpd23285_c

rxn21566_c: cpd00422_c --> cpd00192_c

rxn21570_c: cpd00192_c --> cpd00321_c

rxn21575_c: cpd23291_c <-- cpd23289_c

rxn21577_c: cpd23292_c <=> cpd23289_c

rxn21578_c: cpd23293_c --> cpd23290_c

rxn21580_c: cpd17837_c --> cpd00010_c + cpd23295_c

rxn21582_c: cpd23289_c <-- cpd23296_c

rxn21597_c: cpd01565_c <=> cpd23303_c

rxn21618_c: 2.0 cpd00001_c + cpd23308_c --> 2.0 cpd00117_c + cpd02229_c + cpd23318_c

rxn21619_c: 2.0 cpd00001_c + cpd23308_c --> 2.0 cpd00117_c + cpd02229_c + cpd23318_c

rxn21624_c: cpd00001_c + 3.0 cpd00536_c <-- cpd00011_c + cpd00067_c + cpd23319_c

rxn21630_c: cpd00001_c --> cpd00029_c

rxn21660_c: 3.0 cpd00001_c + 2.0 cpd23343_c --> 4.0 cpd00117_c + cpd02229_c + cpd23344_c

rxn21661_c: 3.0 cpd00001_c + 2.0 cpd23343_c --> 4.0 cpd00117_c + cpd02229_c + cpd23344_c

rxn21663_c: 3.0 cpd00001_c + 2.0 cpd23345_c --> 4.0 cpd00117_c + cpd02229_c + cpd23346_c

rxn21735_c: cpd00017_c + cpd02710_c <=> cpd00019_c + cpd00067_c + cpd21849_c

rxn21736_c: cpd00017_c + cpd28486_c <=> cpd00067_c + cpd00147_c + cpd02710_c

rxn21772_c: cpd23367_c <=> cpd23371_c

rxn21792_c: cpd05919_c --> cpd03615_c

rxn21856_c: cpd11492_c + cpd20921_c <=> cpd00010_c + cpd00011_c + cpd22027_c

rxn21863_c: cpd00005_c + cpd00067_c + cpd27021_c <=> cpd00006_c + cpd21088_c

rxn21864_c: cpd00035_c + cpd00067_c + cpd21087_c <=> cpd00011_c + cpd00800_c + cpd11493_c

rxn21930_c: cpd04654_c --> cpd15077_c

rxn21937_c: cpd27155_c <=> cpd28173_c

rxn21938_c: cpd27156_c <=> cpd28173_c

rxn21962_c: cpd26720_c <=> cpd02957_c

rxn21993_c: --> cpd02957_c

rxn21994_c: cpd00001_c + cpd26950_c <=> cpd26952_c

rxn21995_c: cpd00001_c + cpd26950_c <=> cpd26952_c

rxn21996_c: cpd00001_c + cpd26723_c <=> cpd26722_c

rxn21997_c: cpd00001_c + cpd26723_c <=> cpd26722_c

rxn22002_c: cpd00037_c + cpd27263_c <=> cpd00014_c + cpd27262_c

rxn22025_c: cpd00007_c + 2.0 cpd23496_c --> 2.0 cpd00067_c + cpd25746_c

rxn22032_c: cpd00007_c + 2.0 cpd23496_c --> cpd00001_c + cpd23503_c

rxn22034_c: cpd00001_c + cpd23504_c <-- cpd00033_c + cpd23505_c

rxn22035_c: cpd00001_c + cpd23505_c --> cpd00023_c + cpd23506_c

rxn22050_c: cpd00001_c + cpd09262_c <-- 2.0 cpd00067_c + cpd23516_c

rxn22052_c: cpd23517_c --> cpd00001_c + cpd00067_c + cpd01254_c

rxn22058_c: cpd27954_c <=> cpd27815_c + cpd27839_c

rxn22061_c: cpd23500_c --> cpd23518_c

rxn22064_c: cpd11493_c + cpd27421_c + cpd27638_c <=> cpd00067_c + cpd27419_c + cpd27640_c

rxn22065_c: cpd01264_c + cpd11493_c + cpd27638_c <=> cpd00067_c + cpd12458_c + cpd27640_c

rxn22122_c: 2.0 cpd11836_c <=> 2.0 cpd11493_c + cpd21992_c

rxn22134_c: cpd00010_c + cpd11836_c <=> cpd11493_c + cpd26802_c

rxn22145_c: cpd23572_c --> cpd00010_c

rxn22148_c: cpd23577_c <=> cpd02229_c + cpd28348_c

rxn22153_c: cpd23576_c <=> cpd02229_c + cpd28348_c

rxn22162_c: cpd23582_c <=> cpd02229_c + cpd22292_c

rxn22170_c: cpd19496_c --> cpd23588_c

rxn22184_c: cpd23588_c --> cpd23587_c

rxn22186_c: cpd00005_c + cpd00007_c + cpd00067_c + cpd27317_c <=> cpd00001_c + cpd00006_c + cpd21877_c

rxn22214_c: cpd00004_c + cpd00007_c + cpd03222_c + cpd27029_c <=> cpd00001_c + cpd00003_c + cpd03037_c + cpd27031_c

rxn22251_c: cpd28262_c <=> cpd27848_c

rxn22301_c: cpd14598_c --> cpd23649_c

rxn22302_c: cpd00001_c + cpd23649_c + cpd27638_c <=> cpd00005_c + cpd00067_c + cpd23650_c

rxn22318_c: cpd00007_c + cpd00067_c + cpd23680_c --> cpd00011_c + cpd00025_c + cpd23679_c

rxn22366_c: cpd00002_c + cpd27911_c + cpd27912_c <=> cpd00012_c + cpd00018_c + cpd21917_c

rxn22382_c: cpd00001_c + cpd22354_c <=> cpd00724_c + cpd28035_c

rxn22383_c: cpd00001_c + cpd22354_c <=> cpd00724_c + cpd28035_c

rxn22387_c: cpd00001_c + cpd21896_c <=> cpd00029_c + cpd28101_c

rxn22388_c: cpd00001_c + cpd22038_c <=> cpd00029_c + cpd28101_c

rxn22390_c: cpd28599_c <=> cpd28602_c

rxn22396_c: cpd23721_c --> cpd00666_c

rxn22399_c: cpd00001_c + cpd27581_c <=> cpd26856_c + cpd27580_c

rxn22485_c: cpd23741_c <-- cpd04091_c

rxn22498_c: cpd00001_c + cpd28101_c <=> cpd27370_c + cpd28099_c

rxn22499_c: cpd00001_c + cpd28101_c <=> cpd26931_c + cpd28100_c

rxn22500_c: cpd00001_c + cpd28101_c <=> cpd26931_c + cpd28100_c

rxn22502_c: cpd00001_c + cpd26835_c <=> cpd05262_c + cpd27370_c

rxn22503_c: cpd00001_c + cpd27370_c <=> cpd01562_c + cpd26835_c

rxn22511_c: cpd00002_c + cpd00010_c + cpd27708_c <=> cpd00012_c + cpd00018_c + cpd27221_c

rxn22513_c: cpd00001_c + cpd00067_c + cpd27490_c <=> cpd00179_c + cpd19001_c

rxn22517_c: cpd00179_c <-- cpd19001_c

rxn22520_c: cpd00001_c + cpd00002_c + cpd27490_c <=> cpd00009_c + cpd00018_c + cpd22191_c

rxn22521_c: cpd00001_c + cpd00002_c + cpd27490_c <=> cpd00009_c + cpd00018_c + cpd22191_c

rxn22522_c: cpd00001_c + cpd00002_c + cpd22191_c <=> cpd00009_c + cpd00018_c + cpd27888_c

rxn22523_c: cpd00001_c + cpd00002_c + cpd22191_c <=> cpd00009_c + cpd00018_c + cpd27888_c

rxn22528_c: cpd05401_c --> cpd21079_c

rxn22529_c: cpd00703_c --> cpd21079_c

rxn22536_c: 2.0 cpd00001_c --> 2.0 cpd00009_c

rxn22537_c: cpd00007_c + cpd01024_c + cpd28074_c <=> cpd00001_c + cpd00116_c + cpd28018_c

rxn22583_c: cpd00007_c + cpd01024_c + cpd28074_c <=> cpd00001_c + cpd00116_c + cpd28018_c

rxn22629_c: cpd00001_c + cpd27401_c <=> cpd00076_c + cpd19102_c

rxn22714_c: cpd19117_c --> cpd23768_c

rxn22715_c: cpd00435_c --> cpd01553_c

rxn22720_c: cpd23752_c <=> cpd23753_c

rxn22721_c: cpd00225_c --> cpd03709_c

rxn22729_c: cpd00005_c + cpd00067_c + cpd27019_c <=> cpd00006_c + cpd27387_c

rxn22730_c: cpd26931_c <=> cpd23745_c + cpd26835_c

rxn22731_c: cpd27145_c <=> cpd21204_c

rxn22747_c: cpd00001_c + cpd01376_c <-- cpd03845_c

rxn22748_c: cpd00001_c + cpd22518_c <=> cpd01376_c

rxn22754_c: cpd00001_c + cpd28355_c <=> cpd28357_c

rxn22763_c: cpd00001_c + cpd27994_c <=> cpd01262_c

rxn22768_c: cpd00001_c --> cpd01157_c

rxn22769_c: cpd00001_c --> cpd01157_c

rxn22785_c: cpd00001_c + cpd28189_c <=> cpd02284_c + cpd27058_c

rxn22788_c: cpd00001_c + cpd27176_c <=> cpd27175_c

rxn22789_c: cpd00001_c + cpd27176_c <=> cpd27175_c

rxn22843_c: cpd00001_c --> cpd00179_c

rxn22859_c: cpd01262_c <-- cpd26821_c

rxn22860_c: cpd01262_c <-- cpd26821_c

rxn22861_c: cpd00009_c --> cpd00089_c

rxn22866_c: cpd00001_c + cpd28355_c <=> cpd23848_c + cpd23849_c + cpd23850_c + cpd23851_c

rxn22882_c: cpd00043_c + cpd28361_c <=> cpd00014_c + cpd28358_c

rxn22883_c: cpd00043_c + cpd28358_c <=> cpd00014_c + cpd00067_c + cpd28359_c

rxn22884_c: cpd00043_c + cpd28358_c <=> cpd00014_c + cpd00067_c + cpd28359_c

rxn22885_c: cpd00043_c + cpd28360_c <=> cpd00014_c + cpd28359_c

rxn22886_c: cpd28293_c <-- cpd00014_c

rxn22888_c: cpd00001_c + cpd27905_c <=> cpd23877_c + cpd27668_c

rxn22889_c: cpd00001_c + cpd22315_c <=> cpd23870_c + cpd23871_c

rxn22890_c: cpd00001_c + cpd22315_c <=> cpd23870_c + cpd23871_c

rxn22891_c: cpd00001_c + cpd22315_c <=> cpd23871_c + cpd23873_c

rxn22899_c: cpd00064_c + cpd11836_c <=> cpd11493_c + cpd27389_c

rxn22900_c: cpd11628_c + cpd27389_c <=> cpd11493_c + cpd27709_c

rxn22906_c: cpd00001_c + cpd21856_c <=> cpd11624_c + cpd27058_c

rxn22968_c: cpd00005_c + cpd28215_c <=> cpd00006_c + cpd28272_c

rxn22996_c: cpd00001_c + cpd22519_c <=> cpd01157_c

rxn22997_c: cpd00001_c + cpd22519_c <=> cpd01157_c

rxn23012_c: cpd00001_c + cpd22351_c <=> cpd00067_c + cpd00365_c + cpd27424_c

rxn23016_c: cpd00001_c + cpd26964_c <=> cpd00067_c + cpd00365_c + cpd27424_c

rxn23025_c: cpd25485_c --> cpd07522_c

rxn23034_c: cpd00005_c + cpd00007_c + cpd00067_c + cpd03630_c <=> cpd00001_c + cpd00006_c + cpd23922_c

rxn23037_c: cpd00005_c + cpd00007_c + cpd00067_c + cpd21378_c <=> cpd00001_c + cpd00006_c + cpd23925_c

rxn23039_c: cpd23941_c --> cpd23938_c

rxn23040_c: cpd23941_c --> cpd23939_c

rxn23051_c: cpd00001_c + cpd26711_c <=> cpd01157_c

rxn23052_c: cpd00001_c + cpd26711_c <=> cpd01157_c

rxn23053_c: cpd00001_c + cpd26711_c <=> cpd23788_c

rxn23054_c: cpd00001_c + cpd26711_c <=> cpd23788_c

rxn23093_c: cpd00064_c --> cpd23977_c

rxn23096_c: cpd00001_c + cpd18034_c --> cpd00012_c + cpd06733_c

rxn23097_c: cpd00903_c --> cpd23983_c

rxn23139_c: cpd24010_c <=> cpd24036_c

rxn23172_c: cpd00070_c + cpd11492_c + cpd28494_c <=> cpd00011_c + cpd11493_c + cpd22078_c

rxn23173_c: cpd00070_c + cpd11492_c + cpd28494_c <=> cpd00011_c + cpd11493_c + cpd22078_c

rxn23193_c: cpd00001_c + cpd28511_c <=> 2.0 cpd00067_c + cpd21416_c + cpd28517_c

rxn23194_c: cpd00001_c + cpd28511_c <=> 2.0 cpd00067_c + cpd02481_c + cpd28516_c

rxn23202_c: cpd02252_c --> cpd07134_c

rxn23208_c: cpd24084_c <-- cpd24086_c

rxn23209_c: cpd24086_c <-- cpd24079_c

rxn23214_c: cpd00001_c + cpd27310_c <=> cpd27839_c

rxn23224_c: cpd01138_c --> cpd00666_c

rxn23283_c: cpd00001_c + cpd28485_c <=> cpd00067_c + cpd02077_c + cpd11493_c

rxn23284_c: cpd01942_c + cpd11493_c <=> cpd00010_c + cpd28494_c

rxn23308_c: cpd11493_c + cpd24122_c <=> cpd00010_c + cpd26994_c

rxn23315_c: cpd00001_c + cpd26916_c <=> cpd11493_c + cpd16301_c

rxn23328_c: cpd21470_c <-- cpd00009_c

rxn23332_c: 2.0 cpd00067_c + cpd22251_c <=> cpd00001_c + cpd27722_c

rxn23333_c: cpd00001_c + cpd27722_c <=> cpd02508_c

rxn23352_c: cpd00007_c + cpd00291_c --> cpd00001_c + cpd00612_c

rxn23395_c: cpd04399_c --> cpd24189_c

rxn23396_c: cpd15073_c --> cpd23446_c

rxn23397_c: cpd15074_c --> cpd15075_c

rxn23450_c: 2.0 cpd00007_c + 2.0 cpd00024_c + cpd12737_c <=> 2.0 cpd00011_c + 2.0 cpd00036_c + 2.0 cpd00055_c + cpd27940_c

rxn23457_c: 2.0 cpd00005_c + 2.0 cpd00007_c + 2.0 cpd00067_c + cpd27033_c <=> 2.0 cpd00001_c + 2.0 cpd00006_c + cpd21993_c

rxn23497_c: cpd00025_c + cpd00067_c + cpd27424_c <=> 2.0 cpd00001_c + cpd00011_c + cpd28249_c

rxn23513_c: 3.0 cpd00067_c + cpd00070_c + cpd27247_c <=> cpd00010_c + 2.0 cpd00011_c + cpd11493_c + cpd24230_c

rxn23514_c: cpd00070_c + cpd27419_c <=> cpd00010_c + 2.0 cpd00011_c + cpd11493_c + cpd28249_c

rxn23515_c: 3.0 cpd00067_c + cpd00070_c + cpd12458_c <=> cpd00010_c + 2.0 cpd00011_c + cpd11493_c + cpd24224_c

rxn23516_c: 2.0 cpd00005_c + cpd00007_c + cpd00067_c + cpd27421_c <=> cpd00001_c + 2.0 cpd00006_c + cpd00047_c + cpd22334_c

rxn23520_c: cpd00003_c + cpd00010_c + cpd27421_c <=> cpd00004_c + cpd00067_c + cpd27059_c

rxn23522_c: cpd00003_c + cpd00010_c + cpd27421_c <=> cpd00004_c + cpd00067_c + cpd27059_c

rxn23528_c: cpd00001_c + cpd27421_c <=> cpd00047_c + cpd00067_c + cpd22334_c

rxn23538_c: cpd27421_c <=> cpd00204_c + cpd22334_c

rxn23539_c: cpd27421_c <=> cpd00204_c + cpd22334_c

rxn23600_c: cpd00001_c + cpd00007_c + cpd27582_c <=> cpd00025_c + cpd00040_c + cpd22343_c

rxn23708_c: cpd00001_c + cpd11624_c <=> cpd03850_c + cpd21879_c

rxn23709_c: cpd00001_c + cpd11624_c <=> cpd03850_c + cpd21879_c

rxn23711_c: cpd00001_c + cpd26958_c <=> cpd24466_c + cpd27058_c

rxn23745_c: cpd00001_c --> cpd00665_c

rxn23746_c: cpd00001_c --> cpd00665_c

rxn23770_c: cpd00001_c <=> cpd21755_c

rxn23771_c: cpd00001_c <=> cpd21755_c

rxn23774_c: cpd03467_c --> cpd05335_c

rxn23775_c: cpd03467_c --> cpd05333_c

rxn23776_c: cpd05333_c <-- cpd05334_c

rxn23784_c: cpd05324_c <=> cpd05323_c

rxn23786_c: cpd05323_c <=> cpd22018_c

rxn23807_c: cpd00102_c --> cpd00171_c

rxn23841_c: cpd21899_c --> cpd22205_c

rxn23843_c: cpd27275_c --> cpd21900_c

rxn23845_c: cpd21900_c --> cpd22014_c

rxn23846_c: cpd22014_c --> cpd22037_c

rxn23847_c: cpd21899_c --> cpd21901_c

rxn23883_c: cpd22254_c <-- cpd00093_c

rxn23884_c: cpd00001_c + cpd24459_c <=> cpd00921_c

rxn23890_c: cpd00067_c + cpd21989_c <=> cpd00001_c + cpd00011_c + cpd27060_c

rxn23891_c: cpd21989_c <=> cpd21990_c

rxn23917_c: cpd24472_c <=> cpd24473_c

rxn23920_c: cpd27186_c <=> cpd27490_c

rxn23933_c: cpd22334_c <=> cpd27426_c

rxn23934_c: cpd00007_c + cpd27426_c <=> cpd00025_c + cpd27060_c

rxn23935_c: cpd00007_c + cpd27426_c <=> cpd00025_c + cpd27060_c

rxn23936_c: cpd00007_c + cpd27426_c <=> cpd00025_c + cpd27060_c

rxn23937_c: 2.0 cpd00005_c + cpd00007_c + cpd27058_c <=> cpd00001_c + 2.0 cpd00006_c + cpd27708_c

rxn23958_c: cpd28549_c <=> cpd26728_c

rxn23959_c: cpd26728_c <=> cpd15145_c

rxn24011_c: cpd00035_c + cpd21853_c --> cpd00001_c + cpd00011_c + cpd05205_c

rxn24019_c: cpd24536_c <-- cpd06607_c

rxn24029_c: cpd00092_c --> cpd21871_c

rxn24099_c: cpd24351_c + cpd24563_c <=> cpd00067_c + cpd26919_c + cpd27706_c

rxn24100_c: cpd24351_c + cpd24563_c <=> cpd00067_c + cpd26919_c + cpd27706_c

rxn24116_c: <=> cpd00003_c

rxn24117_c: cpd00374_c --> cpd02361_c

rxn24119_c: cpd00007_c + cpd00069_c --> cpd00291_c

rxn24125_c: cpd21871_c <-- cpd21853_c

rxn24147_c: cpd00001_c + cpd24592_c <=> cpd27039_c

rxn24211_c: cpd00007_c + cpd28068_c <=> cpd00001_c + cpd27743_c

rxn24309_c: cpd00013_c + cpd00196_c + cpd27735_c <=> cpd00009_c + cpd00033_c + cpd28060_c

rxn24310_c: 2.0 cpd00067_c + cpd02295_c + cpd03214_c --> cpd00011_c + cpd00012_c + cpd23602_c

rxn24312_c: cpd00017_c + cpd23602_c --> cpd00019_c + 3.0 cpd00067_c + cpd12844_c

rxn24365_c: cpd27800_c <=> cpd27186_c

rxn24385_c: cpd00070_c + cpd28329_c <=> cpd00010_c + cpd00011_c + cpd28328_c

rxn24386_c: cpd00070_c + cpd28329_c <=> cpd00010_c + cpd00011_c + cpd28328_c

rxn24432_c: cpd00011_c + cpd19585_c <-- cpd00508_c

rxn24439_c: cpd00005_c + cpd00007_c + cpd00067_c + cpd27033_c <=> cpd00001_c + cpd00006_c + cpd22019_c

rxn24448_c: cpd27640_c + cpd27879_c <=> cpd00001_c + cpd27638_c + cpd27881_c

rxn24449_c: cpd27640_c + cpd27879_c <=> cpd00001_c + cpd27638_c + cpd27881_c

rxn24470_c: cpd00005_c + cpd21918_c <=> cpd00006_c + cpd00067_c + cpd28272_c

rxn24506_c: cpd00001_c + cpd11628_c <=> cpd11493_c + cpd27058_c

rxn24507_c: cpd00001_c + cpd11628_c <=> cpd11493_c + cpd27058_c

rxn24508_c: cpd00007_c + cpd12458_c + cpd28082_c <=> 2.0 cpd00001_c + cpd27716_c + cpd27757_c

rxn24509_c: cpd00007_c + cpd12458_c + cpd28082_c <=> 2.0 cpd00001_c + cpd27716_c + cpd27757_c

rxn24510_c: cpd00002_c + cpd00010_c + cpd27424_c <=> cpd00012_c + cpd00018_c + cpd00067_c + cpd27059_c

rxn24511_c: cpd00002_c + cpd00010_c + cpd27424_c <=> cpd00012_c + cpd00018_c + cpd00067_c + cpd27059_c

rxn24512_c: cpd00002_c + cpd00010_c + cpd27424_c <=> cpd00012_c + cpd00018_c + cpd00067_c + cpd27059_c

rxn24513_c: cpd00002_c + cpd00010_c + cpd27424_c <=> cpd00012_c + cpd00018_c + cpd00067_c + cpd27059_c

rxn24514_c: cpd00002_c + cpd00010_c + cpd27424_c <=> cpd00012_c + cpd00018_c + cpd00067_c + cpd27059_c

rxn24517_c: cpd00241_c + cpd26946_c <=> cpd09118_c + cpd26896_c

rxn24518_c: cpd00006_c + cpd28232_c <=> cpd00005_c + cpd00067_c + cpd28215_c

rxn24526_c: cpd00001_c + cpd28278_c <=> cpd00067_c + cpd26675_c + cpd26958_c

rxn24587_c: 2.0 cpd00013_c + cpd00244_c + cpd01620_c <=> 2.0 cpd00001_c + 3.0 cpd00067_c + cpd24795_c

rxn24616_c: cpd03503_c <-- cpd00001_c

rxn24634_c: cpd24816_c + cpd24821_c <=> cpd00001_c + cpd27506_c

rxn24644_c: cpd28223_c <=> cpd27301_c

rxn24645_c: cpd27675_c <=> cpd00048_c + cpd27670_c

rxn24658_c: cpd00549_c --> cpd00922_c

rxn24659_c: cpd00269_c <-- cpd00379_c

rxn24701_c: cpd08613_c <=> cpd24991_c

rxn24702_c: cpd08613_c <=> cpd08614_c

rxn24703_c: cpd08614_c <=> cpd03951_c

rxn24704_c: cpd24991_c <=> cpd03951_c

rxn24719_c: cpd00554_c --> cpd03934_c

rxn24835_c: cpd27143_c <=> cpd02077_c

rxn24836_c: cpd27143_c <=> cpd02077_c

rxn24840_c: cpd15016_c <-- cpd25049_c

rxn24841_c: cpd15016_c <-- cpd25049_c

rxn24890_c: cpd25090_c --> cpd25091_c

rxn24922_c: cpd25111_c + cpd27138_c <=> cpd25112_c

rxn24923_c: cpd25112_c + cpd27138_c <=> cpd25113_c

rxn24925_c: cpd25115_c + cpd27138_c <=> cpd25114_c

rxn24926_c: cpd25114_c + cpd27138_c <=> cpd25116_c

rxn25057_c: cpd00007_c + cpd00024_c + cpd12736_c <=> cpd00011_c + cpd00036_c + cpd00055_c + cpd27940_c

rxn25061_c: cpd00005_c + cpd00007_c + 2.0 cpd00067_c + cpd22019_c <=> cpd00001_c + cpd00006_c + cpd21993_c

rxn25070_c: cpd00065_c <-- cpd25487_c

rxn25105_c: cpd00007_c + 5.0 cpd00067_c + 2.0 cpd07113_c <=> 3.0 cpd00001_c + cpd25506_c

rxn25131_c: cpd00017_c + cpd14961_c <=> cpd00011_c + cpd00019_c + cpd00067_c + cpd25541_c

rxn25198_c: cpd00007_c + cpd00024_c + 2.0 cpd00067_c + cpd09080_c <=> cpd00011_c + cpd00036_c + cpd25461_c

rxn25199_c: cpd08695_c --> cpd04580_c

rxn25200_c: cpd08696_c --> cpd08697_c

rxn25212_c: cpd21072_c --> cpd25616_c

rxn25217_c: cpd04063_c --> cpd25619_c

rxn25234_c: cpd19401_c --> cpd00011_c + cpd07679_c

rxn25263_c: cpd00001_c + cpd00023_c + cpd00900_c <-- cpd00024_c + 2.0 cpd00067_c + cpd25666_c

rxn25313_c: cpd26551_c <-- cpd28281_c

rxn25314_c: cpd00001_c + cpd25936_c <-- cpd00190_c

rxn25315_c: cpd00001_c + cpd25936_c <-- cpd00190_c

rxn25316_c: cpd00009_c + cpd25936_c <-- cpd00089_c

rxn25318_c: cpd25701_c + cpd28293_c <=> cpd00014_c + cpd27499_c

rxn25319_c: cpd25700_c + cpd28293_c <=> cpd00014_c + cpd27229_c

rxn25320_c: cpd05876_c + cpd28293_c <=> cpd00014_c + cpd28185_c

rxn25321_c: cpd17551_c + cpd28293_c <=> cpd00014_c + cpd28186_c

rxn25324_c: cpd25716_c <=> cpd25717_c

rxn25332_c: cpd25721_c --> cpd25722_c

rxn25333_c: cpd00017_c + cpd01100_c --> cpd00019_c + cpd25722_c

rxn25335_c: cpd14908_c <-- cpd25723_c

rxn25336_c: cpd25723_c --> cpd25724_c

rxn25337_c: cpd25723_c --> cpd25725_c

rxn25339_c: cpd04758_c --> cpd25726_c

rxn25357_c: cpd25738_c --> cpd25740_c

rxn25358_c: cpd25739_c --> cpd25741_c

rxn25366_c: cpd00144_c + cpd21757_c <=> cpd00014_c + cpd27176_c

rxn25369_c: cpd01703_c --> cpd02395_c

rxn25374_c: cpd02295_c <-- cpd07257_c

rxn25375_c: cpd00163_c <-- cpd00014_c

rxn25376_c: cpd00202_c + cpd02295_c <-- cpd01045_c

rxn25377_c: cpd00070_c <-- cpd25772_c

rxn25380_c: cpd21899_c --> cpd26870_c

rxn25392_c: cpd25781_c + cpd25782_c <-- cpd25783_c

rxn25397_c: cpd08027_c --> cpd00180_c

rxn25401_c: cpd00001_c + cpd25787_c <=> cpd22209_c + cpd25522_c

rxn25411_c: cpd00094_c --> cpd25800_c

rxn25414_c: cpd25806_c <=> cpd25807_c

rxn25420_c: cpd06574_c --> cpd25819_c

rxn25425_c: cpd25720_c --> cpd25820_c

rxn25430_c: cpd03829_c --> cpd25821_c

rxn25431_c: cpd25821_c --> cpd25822_c

rxn25432_c: cpd25807_c <-- cpd05207_c

rxn25437_c: cpd25831_c --> cpd00010_c + cpd25832_c

rxn25438_c: cpd25832_c --> cpd25833_c

rxn25451_c: cpd00005_c + cpd16516_c <=> cpd00006_c + cpd08113_c

rxn25452_c: cpd00005_c + cpd08113_c --> cpd00006_c + cpd16518_c

rxn25496_c: cpd07316_c --> cpd07355_c

rxn25498_c: cpd00318_c --> cpd03946_c

rxn25507_c: 2.0 cpd00005_c + 2.0 cpd00067_c + cpd27059_c <=> 2.0 cpd00006_c + cpd00010_c + cpd27426_c

rxn25523_c: cpd17741_c --> cpd25924_c

rxn25574_c: cpd04322_c <-- cpd22559_c

rxn25585_c: cpd00163_c + cpd21756_c <=> cpd00014_c + cpd27130_c

rxn25586_c: cpd00163_c + cpd21756_c <=> cpd00014_c + cpd27130_c

rxn25587_c: cpd00043_c + cpd28361_c <=> cpd00014_c + cpd28360_c

rxn25588_c: cpd00043_c + cpd28361_c <=> cpd00014_c + cpd28360_c

rxn25592_c: cpd00390_c <-- cpd22571_c

rxn25601_c: cpd00022_c <-- cpd23483_c

rxn25624_c: cpd22595_c <-- cpd02314_c

rxn25627_c: cpd02317_c --> cpd16552_c

rxn25628_c: cpd22594_c <-- cpd24509_c

rxn25635_c: cpd24511_c --> cpd16551_c

rxn25671_c: cpd19036_c <-- cpd15486_c

rxn25676_c: cpd00190_c + cpd22598_c <-- 2.0 cpd00067_c + cpd22609_c

rxn25681_c: cpd00163_c + cpd21755_c <=> cpd00014_c + cpd28354_c

rxn25682_c: cpd00163_c + cpd21755_c <=> cpd00014_c + cpd28354_c

rxn25683_c: cpd00009_c + cpd11628_c <=> cpd11493_c + cpd22306_c

rxn25688_c: cpd28117_c <=> cpd28116_c

rxn25689_c: cpd00001_c + cpd00007_c + cpd27915_c <=> cpd00013_c + cpd00025_c + cpd22319_c

rxn25690_c: cpd00001_c + cpd00007_c + cpd27915_c <=> cpd00013_c + cpd00025_c + cpd22319_c

rxn25700_c: cpd22612_c --> cpd09026_c

rxn25707_c: cpd22618_c --> cpd22619_c

rxn25708_c: cpd22619_c --> 2.0 cpd00067_c + cpd22620_c

rxn25714_c: cpd00007_c + cpd21074_c + cpd27640_c <=> cpd00001_c + cpd27638_c + cpd28252_c

rxn25715_c: cpd00007_c + cpd21074_c + cpd27640_c <=> cpd00001_c + cpd27638_c + cpd28252_c

rxn25720_c: cpd00003_c + cpd12458_c <=> cpd00004_c + cpd00067_c + cpd14940_c

rxn25767_c: cpd18061_c --> cpd06105_c

rxn25770_c: cpd05956_c --> cpd18062_c

rxn25771_c: cpd05956_c --> cpd06180_c

rxn25772_c: cpd18062_c --> cpd06180_c

rxn25790_c: cpd00005_c + cpd03502_c <=> cpd00001_c + cpd00006_c + cpd06619_c

rxn25878_c: cpd00719_c + cpd22724_c <=> cpd22251_c

rxn25939_c: cpd05523_c <=> cpd05822_c

rxn25942_c: 5.0 cpd00070_c <-- 5.0 cpd00010_c + cpd00011_c + cpd02495_c

rxn25956_c: cpd00374_c --> cpd02357_c

rxn25960_c: cpd00001_c --> cpd30321_c

rxn25966_c: cpd00061_c + 5.0 cpd00067_c + cpd00235_c --> cpd00009_c + cpd22793_c

rxn25992_c: cpd03587_c + cpd27865_c <=> cpd15483_c + cpd22223_c

rxn25998_c: cpd03587_c + 2.0 cpd14878_c <-- 2.0 cpd19245_c + cpd27350_c

rxn25999_c: cpd03587_c + 2.0 cpd14878_c <-- 2.0 cpd19245_c + cpd27350_c

rxn26015_c: cpd26913_c <=> cpd00092_c + cpd22278_c

rxn26042_c: cpd00001_c + cpd27149_c <=> 2.0 cpd27839_c

rxn26044_c: cpd27149_c <=> cpd00051_c + cpd27839_c

rxn26045_c: cpd27149_c <=> cpd00051_c + cpd27839_c

rxn26075_c: cpd28041_c <=> cpd27686_c

rxn26086_c: cpd26896_c <=> cpd27686_c

rxn26088_c: cpd28179_c <=> cpd28175_c

rxn26095_c: 2.0 cpd00017_c + cpd27554_c + cpd28207_c <=> cpd00019_c + cpd00060_c + cpd00067_c + cpd03091_c + cpd21884_c + cpd28307_c

rxn26117_c: cpd03831_c + cpd26544_c --> cpd00008_c + cpd26545_c

rxn26118_c: cpd03831_c + cpd26544_c --> cpd00008_c + cpd26545_c

rxn26121_c: cpd02113_c + cpd27415_c <=> cpd00297_c + cpd26547_c

rxn26124_c: cpd00017_c + cpd28573_c <=> cpd00019_c + cpd28548_c

rxn26125_c: cpd00001_c + cpd26551_c <-- cpd01399_c + cpd25936_c

rxn26126_c: cpd00001_c --> cpd01329_c

rxn26132_c: cpd26006_c <=> cpd26007_c + cpd26008_c

rxn26133_c: cpd26006_c <=> cpd26007_c + cpd26008_c

rxn26134_c: cpd26006_c <=> cpd26007_c + cpd26008_c

rxn26311_c: cpd28041_c <=> cpd21928_c + cpd22082_c

rxn26339_c: cpd00037_c <=> cpd00014_c + cpd26014_c

rxn26340_c: cpd00037_c <=> cpd00014_c + cpd26014_c

rxn26353_c: cpd00008_c + cpd00009_c + cpd00070_c --> cpd00002_c + 2.0 cpd00067_c + cpd00242_c + cpd00782_c

rxn26355_c: cpd00002_c + cpd11493_c + cpd27058_c <=> cpd00012_c + cpd00018_c + cpd28167_c

rxn26356_c: cpd00002_c + cpd11493_c + cpd27058_c <=> cpd00012_c + cpd00018_c + cpd28167_c

rxn26357_c: cpd28166_c + cpd28167_c <=> cpd11493_c + cpd21848_c

rxn26358_c: cpd28166_c + cpd28167_c <=> cpd11493_c + cpd21848_c

rxn26375_c: cpd00202_c + cpd28567_c <=> cpd00012_c + cpd28549_c

rxn26449_c: cpd00004_c + cpd28018_c <=> cpd00003_c + cpd28170_c

rxn26488_c: cpd03043_c <-- cpd00010_c

rxn26493_c: cpd22299_c <=> cpd00001_c + cpd26552_c + cpd27896_c

rxn26579_c: cpd00067_c + cpd26566_c + cpd26582_c <=> cpd00010_c + cpd00018_c + cpd21913_c + cpd28373_c

rxn26597_c: cpd28418_c <=> cpd28386_c

rxn26598_c: cpd28386_c <=> cpd28383_c

rxn26608_c: cpd22497_c + cpd26585_c <=> cpd26591_c + cpd27896_c

rxn26609_c: cpd22499_c + cpd26585_c <=> cpd26562_c + cpd27896_c

rxn26610_c: cpd22501_c + cpd26585_c <=> cpd26563_c + cpd27896_c

rxn26611_c: cpd22500_c + cpd26585_c <=> cpd26564_c + cpd27896_c

rxn26612_c: cpd22502_c + cpd26585_c <=> cpd26565_c + cpd27896_c

rxn26615_c: cpd00067_c + cpd26582_c + cpd26583_c <=> cpd00010_c + cpd00018_c + cpd21913_c + cpd28469_c

rxn26624_c: cpd28386_c <=> cpd28607_c

rxn26626_c: cpd28607_c <=> cpd28603_c

rxn26633_c: cpd28421_c <=> cpd28387_c

rxn26636_c: cpd28387_c <=> cpd28384_c

rxn26637_c: cpd28387_c <=> cpd28634_c

rxn26639_c: cpd00070_c + cpd12458_c <=> cpd00010_c + cpd00011_c + cpd22072_c

rxn26646_c: cpd00002_c + cpd28388_c <=> cpd00008_c + cpd00009_c + cpd11493_c + cpd26566_c

rxn26649_c: cpd00002_c + cpd28383_c <=> cpd00008_c + cpd00009_c + cpd11493_c + cpd26579_c

rxn26650_c: cpd00002_c + cpd28603_c <=> cpd00008_c + cpd00009_c + cpd11493_c + cpd26580_c

rxn26651_c: cpd00002_c + cpd28384_c <=> cpd00008_c + cpd00009_c + cpd11493_c + cpd26583_c

rxn26652_c: cpd00002_c + cpd28605_c <=> cpd00008_c + cpd00009_c + cpd11493_c + cpd26584_c

rxn26659_c: cpd28415_c <=> cpd28388_c

rxn26660_c: cpd01393_c + cpd11492_c <=> cpd00010_c + cpd00011_c + cpd22073_c

rxn26676_c: cpd00010_c + cpd26686_c <=> cpd11493_c + cpd26581_c

rxn26679_c: cpd00010_c + cpd22388_c <=> cpd01393_c + cpd11493_c

rxn26725_c: cpd00067_c + cpd26579_c + cpd26582_c <=> cpd00010_c + cpd00018_c + cpd21913_c + cpd28471_c

rxn26726_c: cpd26597_c <=> 2.0 cpd00067_c + cpd26685_c

rxn26740_c: cpd27876_c <=> cpd22362_c

rxn26748_c: cpd22362_c + cpd27308_c <=> cpd27295_c

rxn26780_c: cpd24389_c --> cpd24401_c

rxn26781_c: cpd24401_c --> cpd20823_c

rxn26793_c: cpd22104_c --> cpd22052_c

rxn26821_c: cpd24402_c --> cpd20823_c

rxn26822_c: cpd24385_c <=> cpd24397_c

rxn26823_c: cpd24385_c <-- cpd24396_c

rxn26836_c: cpd00007_c + cpd24400_c + cpd28070_c <=> cpd00001_c + cpd24389_c + cpd27744_c

rxn26844_c: cpd04214_c --> cpd24414_c

rxn27027_c: cpd27059_c + cpd28325_c <=> cpd00010_c + cpd28335_c

rxn27028_c: cpd27059_c + cpd28325_c <=> cpd00010_c + cpd28335_c

rxn27061_c: cpd05313_c <=> cpd05312_c

rxn27062_c: cpd17439_c <=> cpd26625_c

rxn27063_c: cpd17441_c <=> cpd26626_c

rxn27064_c: cpd17443_c <=> cpd17458_c

rxn27065_c: cpd05331_c <=> cpd26623_c

rxn27068_c: cpd05312_c <=> cpd05315_c

rxn27075_c: cpd24359_c <-- cpd01881_c

rxn27077_c: 2.0 cpd26650_c <-- cpd26646_c

rxn27078_c: cpd26650_c --> cpd26645_c

rxn27111_c: cpd00383_c + cpd28018_c <=> cpd01716_c + cpd28074_c

rxn27157_c: cpd00383_c + cpd28018_c <=> cpd01716_c + cpd28074_c

rxn27210_c: cpd00001_c + cpd28189_c <=> cpd00067_c + cpd00098_c + cpd01899_c

rxn27212_c: cpd00267_c + cpd22234_c <=> cpd00010_c + cpd00067_c + cpd26685_c

rxn27253_c: 2.0 cpd00076_c <=> cpd00190_c + cpd27312_c

rxn27281_c: cpd00017_c + cpd27089_c + cpd28083_c <=> cpd00060_c + cpd00067_c + cpd03091_c + cpd27073_c + cpd27088_c

rxn27338_c: cpd00003_c + cpd22234_c <=> cpd00004_c + cpd00067_c + cpd28232_c

rxn27339_c: cpd00003_c + cpd22234_c <=> cpd00004_c + cpd00067_c + cpd28232_c

rxn27360_c: <=> cpd00061_c

rxn27384_c: cpd00001_c + cpd28278_c <=> cpd00067_c + cpd26675_c + cpd26958_c

rxn27387_c: cpd00017_c + cpd28567_c <=> cpd00019_c + cpd28598_c

rxn27388_c: cpd00002_c + 2.0 cpd00052_c + cpd28576_c <=> 3.0 cpd00012_c + cpd28600_c

rxn27416_c: cpd00001_c + cpd28296_c <=> 2.0 cpd00067_c + cpd00091_c + cpd22358_c

rxn27417_c: cpd00001_c + cpd28296_c <=> 2.0 cpd00067_c + cpd00091_c + cpd22358_c

rxn27444_c: cpd00001_c + cpd28335_c <=> cpd00067_c + cpd27422_c + cpd27426_c

rxn27463_c: cpd22337_c + 2.0 cpd27814_c <=> cpd00001_c + cpd22318_c + cpd27930_c

rxn27464_c: cpd22337_c + 2.0 cpd27814_c <=> cpd00001_c + cpd22318_c + cpd27930_c

rxn27501_c: cpd00005_c + cpd00017_c <=> cpd00006_c + cpd00019_c + cpd27445_c + cpd27507_c

rxn27509_c: cpd00042_c <-- cpd00033_c

rxn27510_c: cpd28293_c <-- cpd00014_c

rxn27511_c: cpd11463_c + cpd28293_c <=> cpd00014_c + cpd25341_c

rxn27512_c: cpd27149_c + cpd28293_c <=> cpd00014_c + cpd25341_c

rxn27514_c: cpd27127_c + cpd27627_c <=> cpd00031_c + 2.0 cpd00067_c + cpd27628_c

rxn27516_c: cpd00037_c + cpd14490_c + cpd27236_c <=> cpd00014_c + cpd14491_c + cpd27235_c

rxn27517_c: cpd27653_c <=> cpd27685_c

rxn27518_c: cpd00003_c + 2.0 cpd11953_c <=> cpd00133_c + 2.0 cpd12522_c

rxn27526_c: cpd00002_c + cpd27149_c <=> cpd00008_c + cpd27148_c

rxn27530_c: cpd00009_c <=> cpd27685_c

rxn27544_c: cpd00006_c + cpd11836_c <=> cpd00005_c + cpd00067_c + cpd11726_c

rxn27546_c: cpd11492_c + cpd11628_c + cpd28167_c <=> cpd00011_c + cpd11493_c + cpd11726_c

rxn27552_c: cpd00001_c + cpd27148_c <=> cpd00009_c + cpd27149_c

rxn27553_c: 2.0 cpd00001_c + cpd27493_c <=> 2.0 cpd19009_c + cpd27492_c

rxn27556_c: cpd00001_c + cpd22357_c <=> cpd19009_c + cpd28035_c

rxn27561_c: cpd27149_c <=> 2.0 cpd27839_c

rxn27674_c: cpd00007_c + cpd22234_c <=> cpd00025_c + cpd28232_c

rxn27676_c: cpd00015_c + cpd22234_c + cpd28168_c <=> cpd00982_c + cpd26839_c

rxn27677_c: cpd00002_c + cpd00010_c + cpd27058_c <=> cpd00012_c + cpd00018_c + cpd22234_c + cpd28168_c

rxn27704_c: cpd00001_c + cpd21754_c <=> cpd15495_c

rxn27750_c: cpd26687_c <=> cpd27033_c

rxn27764_c: cpd00001_c + cpd01236_c <=> cpd00999_c + cpd28718_c

rxn27784_c: cpd26891_c <=> cpd26888_c

rxn27832_c: cpd00017_c + cpd11461_c <=> cpd00019_c + cpd22153_c

rxn27833_c: cpd26941_c <=> cpd00012_c

rxn27834_c: cpd27687_c <=> cpd00012_c

rxn27835_c: cpd27687_c <=> cpd00012_c

rxn27836_c: cpd00002_c + cpd26848_c <=> cpd00012_c + cpd00018_c

rxn27870_c: cpd22140_c + cpd22369_c <=> cpd22139_c + cpd27839_c

rxn27901_c: cpd00080_c + cpd11628_c <=> cpd11493_c + cpd22235_c

rxn27902_c: cpd00080_c + cpd11628_c <=> cpd11493_c + cpd22235_c

rxn27903_c: cpd00080_c + cpd11628_c <=> cpd11493_c + cpd22235_c

rxn27910_c: 2.0 cpd00067_c + cpd21754_c <=> cpd22197_c

rxn27912_c: cpd00009_c + cpd27186_c <=> cpd00089_c + cpd26551_c

rxn27913_c: cpd00009_c + cpd27186_c <=> cpd00089_c + cpd01399_c

rxn28047_c: cpd00113_c --> cpd00012_c

rxn28049_c: cpd00113_c --> cpd00012_c

rxn28094_c: cpd00002_c + cpd27149_c <=> cpd00008_c + cpd27148_c

rxn28095_c: cpd00002_c + cpd27149_c <=> cpd00008_c + cpd27148_c

rxn28170_c: cpd00002_c + cpd00010_c + cpd27058_c <=> cpd00012_c + cpd00018_c + cpd28168_c

rxn28171_c: cpd01078_c <=> cpd03389_c

rxn28172_c: cpd01078_c <=> cpd03163_c

rxn28196_c: cpd00075_c + 6.0 cpd28082_c <=> cpd00001_c + cpd00013_c + 6.0 cpd27757_c

rxn28197_c: 2.0 cpd00001_c + cpd00013_c + 5.0 cpd27757_c <=> 7.0 cpd00067_c + cpd00075_c + 6.0 cpd28082_c

rxn28263_c: cpd03467_c --> cpd05333_c

rxn28274_c: cpd00001_c --> cpd00023_c

rxn28343_c: cpd00007_c + cpd00300_c --> cpd00011_c + cpd00025_c + cpd01092_c

rxn28348_c: cpd27800_c <=> cpd22197_c

rxn28354_c: cpd00070_c + cpd27059_c <=> cpd00010_c + cpd00011_c + cpd22026_c

rxn28358_c: cpd00005_c + cpd28232_c <=> cpd00006_c + cpd27059_c

rxn28370_c: cpd00005_c + cpd21918_c <=> cpd00006_c + cpd28272_c

rxn28375_c: cpd00006_c + cpd28232_c <=> cpd00005_c + cpd00067_c + cpd28215_c

rxn28399_c: cpd00218_c + cpd03688_c <-- cpd00554_c

rxn28416_c: cpd00061_c <-- cpd00236_c

rxn28417_c: cpd00236_c <-- cpd01716_c

rxn28418_c: cpd25046_c --> cpd01020_c

rxn28420_c: cpd25046_c <-- cpd00197_c

rxn28421_c: cpd22503_c --> cpd25046_c

rxn28424_c: 2.0 cpd07725_c <-- 2.0 cpd11640_c + 2.0 cpd25746_c

rxn28425_c: cpd25763_c --> cpd25764_c

rxn28426_c: cpd25764_c <-- cpd25765_c

rxn28427_c: cpd25765_c --> cpd25762_c

rxn28428_c: cpd25762_c --> cpd25758_c

rxn28429_c: cpd25758_c <-- cpd25768_c

rxn28430_c: cpd25768_c <-- cpd25769_c

rxn28431_c: cpd25769_c <-- cpd25760_c

rxn28432_c: cpd25763_c <-- cpd25755_c

rxn28433_c: cpd25755_c --> cpd25766_c

rxn28434_c: cpd25766_c <-- cpd25767_c

rxn28435_c: cpd25767_c --> cpd25752_c

rxn28436_c: cpd25752_c <-- cpd25770_c

rxn28437_c: cpd25770_c --> cpd25771_c

rxn28439_c: cpd25755_c --> cpd25753_c

rxn28440_c: cpd25755_c --> cpd25761_c

rxn28441_c: cpd25767_c --> cpd25754_c

rxn28442_c: cpd25754_c --> cpd25768_c

rxn28443_c: cpd25754_c --> cpd25756_c

rxn28444_c: cpd25752_c --> cpd25756_c

rxn28446_c: cpd00005_c + 3.0 cpd00067_c + cpd11640_c + 4.0 cpd17156_c <=> cpd00006_c + cpd03938_c + cpd08233_c

rxn28448_c: cpd20995_c <=> cpd28803_c

rxn28450_c: cpd28804_c <=> cpd28805_c

rxn28451_c: cpd28804_c <=> cpd28802_c

rxn28452_c: cpd28802_c <=> cpd28809_c

rxn28454_c: cpd20995_c <=> cpd28807_c

rxn28456_c: cpd28806_c <=> cpd28810_c

rxn28457_c: cpd28804_c <=> cpd28811_c

rxn28458_c: cpd28804_c <=> cpd28812_c

rxn28459_c: cpd28804_c <=> cpd28813_c

rxn28461_c: cpd00604_c <=> cpd28799_c

rxn28463_c: cpd06210_c --> cpd06158_c

rxn28464_c: cpd06158_c <=> cpd01209_c + cpd26936_c

rxn28465_c: cpd06210_c <=> cpd06163_c + cpd26936_c

rxn28466_c: cpd06163_c --> cpd18060_c

rxn28467_c: cpd22504_c <-- cpd22503_c

rxn28468_c: cpd22503_c <=> cpd18067_c + cpd26936_c

rxn28469_c: cpd18067_c <=> cpd06158_c

rxn28470_c: cpd01059_c <=> cpd18068_c

rxn28471_c: cpd18068_c <=> cpd01209_c

rxn28472_c: cpd18060_c <=> cpd06206_c + cpd26936_c

rxn28473_c: cpd01209_c <=> cpd06206_c + cpd26936_c

rxn28476_c: cpd06158_c <=> cpd18060_c + cpd26936_c

rxn28477_c: cpd06200_c <=> cpd29622_c

rxn28478_c: cpd29622_c <=> cpd28800_c

rxn28479_c: cpd28800_c <=> cpd01893_c + cpd26936_c

rxn28480_c: cpd28678_c <=> cpd01893_c

rxn28481_c: cpd00564_c <=> cpd28800_c

rxn28482_c: cpd28800_c <=> cpd26936_c + cpd28801_c

rxn28484_c: cpd01097_c <=> cpd28801_c

rxn28491_c: cpd06210_c <-- cpd18061_c

rxn28492_c: cpd03502_c <=> cpd05631_c

rxn28493_c: cpd05631_c --> cpd08923_c

rxn28494_c: cpd08923_c <=> cpd28729_c

rxn28495_c: cpd28729_c <=> cpd15009_c

rxn28503_c: cpd00080_c + cpd28018_c <=> cpd00095_c + cpd28074_c

rxn28511_c: cpd00080_c + cpd28018_c <=> cpd00095_c + cpd28074_c

rxn28519_c: cpd00080_c + cpd28018_c <=> cpd00095_c + cpd28074_c

rxn28538_c: cpd00001_c --> cpd30321_c

rxn28667_c: cpd02113_c + cpd27415_c <=> cpd00297_c + cpd26547_c

rxn28669_c: cpd00001_c + cpd21754_c <=> cpd01329_c

rxn28735_c: cpd00202_c + cpd28567_c <=> cpd00012_c + cpd28549_c

rxn28736_c: cpd00202_c + cpd28567_c <=> cpd00012_c + cpd28549_c

rxn28816_c: cpd28853_c <=> cpd28842_c

rxn28822_c: cpd28838_c <=> cpd28839_c

rxn28825_c: cpd28853_c <=> cpd28843_c

rxn28826_c: cpd28853_c <=> cpd28841_c

rxn28827_c: cpd28853_c <=> cpd25700_c

rxn28828_c: cpd28841_c <=> cpd28849_c

rxn28830_c: cpd28844_c <=> cpd28852_c

rxn28831_c: cpd28853_c <=> cpd28844_c

rxn28832_c: cpd28854_c <=> cpd28851_c

rxn28833_c: cpd25700_c <=> cpd28850_c

rxn28834_c: cpd28853_c <=> cpd28854_c

rxn28835_c: cpd28841_c <=> cpd28845_c

rxn28836_c: cpd28841_c <=> cpd28846_c

rxn28837_c: cpd00002_c + cpd00070_c + cpd28846_c <=> cpd00010_c + cpd00012_c + cpd00018_c + cpd28847_c

rxn28838_c: cpd28841_c <=> cpd28848_c

rxn28839_c: cpd03038_c --> cpd03035_c

rxn28842_c: cpd00002_c + cpd00053_c + cpd27374_c <=> cpd00008_c + cpd00009_c + cpd00023_c + cpd26690_c

rxn28845_c: cpd29075_c <=> cpd07680_c

rxn28846_c: cpd07680_c <=> cpd00011_c + cpd29074_c

rxn28848_c: cpd00321_c <=> cpd28856_c

rxn28850_c: cpd28877_c <=> cpd28880_c

rxn28851_c: cpd28880_c <=> cpd28860_c

rxn28853_c: cpd28875_c <=> cpd28874_c

rxn28857_c: cpd28866_c <=> cpd28874_c

rxn28858_c: cpd28879_c <=> cpd28861_c

rxn28859_c: cpd28865_c <=> cpd28866_c

rxn28860_c: cpd28864_c <=> cpd28865_c

rxn28863_c: cpd28857_c <=> cpd28864_c

rxn28864_c: cpd28865_c <=> cpd28875_c

rxn28866_c: cpd28877_c <=> cpd28881_c

rxn28867_c: cpd28881_c <=> cpd28871_c

rxn28869_c: cpd28882_c <=> cpd28883_c

rxn28870_c: cpd28879_c <=> cpd28870_c

rxn28871_c: cpd08675_c <=> cpd28879_c

rxn28872_c: cpd28879_c <=> cpd28862_c

rxn28878_c: cpd28760_c <=> cpd28761_c

rxn28891_c: cpd00007_c + cpd00291_c + cpd10515_c --> cpd02914_c

rxn28900_c: cpd00001_c + cpd27638_c + cpd29656_c <=> cpd00023_c + cpd00067_c + cpd27640_c

rxn28905_c: cpd29097_c <=> cpd02054_c

rxn28937_c: cpd05296_c <=> cpd29023_c

rxn28938_c: cpd05296_c <=> cpd29024_c

rxn28939_c: cpd24985_c <=> cpd29021_c

rxn28940_c: cpd24985_c <=> cpd29022_c

rxn28941_c: cpd05288_c <=> cpd29025_c

rxn28942_c: cpd05288_c <=> cpd29026_c

rxn28949_c: cpd00506_c <-- cpd01293_c

rxn28950_c: cpd00042_c <=> cpd00506_c

rxn28964_c: cpd29047_c <=> cpd29048_c

rxn28965_c: cpd29049_c <=> cpd05335_c

rxn28975_c: cpd00080_c + cpd29077_c <=> cpd00046_c + cpd00067_c + cpd29080_c

rxn28976_c: cpd00080_c + cpd29077_c <=> cpd00046_c + cpd00067_c + cpd29080_c

rxn29240_c: <=> cpd00002_c + cpd00008_c

rxn29318_c: <=> cpd00002_c

rxn29320_c: <=> cpd00008_c

rxn29335_c: <=> cpd00031_c

rxn29736_c: cpd00067_e <=> cpd00038_c + cpd00067_c

rxn29784_c: cpd00067_e <=> cpd00002_c + cpd00067_c

rxn29785_c: <=> cpd00004_c

rxn29791_c: cpd00067_e <=> cpd00002_c + cpd00067_c

rxn29852_c: cpd00067_e <=> cpd00008_c + cpd00067_c

rxn29867_c: cpd00067_e <=> cpd00031_c + cpd00067_c

rxn29868_c: cpd00067_e <=> cpd00038_c + cpd00067_c

rxn29920_c: cpd00006_c + cpd22234_c + cpd28168_c <=> cpd00005_c + cpd28232_c

rxn29925_c: cpd00001_c + cpd28278_c <=> cpd00067_c + cpd26675_c + cpd26958_c

rxn29927_c: cpd00017_c + cpd28567_c <=> cpd00019_c + cpd12482_c

rxn29928_c: cpd00002_c <=> cpd00012_c + cpd28600_c

rxn29929_c: cpd00052_c <=> cpd00012_c + cpd28600_c

rxn29930_c: cpd00017_c + cpd28567_c <=> cpd00019_c + cpd12481_c

rxn29931_c: cpd00017_c + cpd28567_c <=> cpd00019_c + cpd12483_c

rxn29932_c: cpd00017_c + cpd28567_c <=> cpd00019_c + cpd12484_c

rxn30110_c: cpd00113_c + cpd00202_c <-- cpd00012_c + cpd00289_c

rxn30111_c: cpd00113_c + cpd00289_c --> cpd00012_c + cpd00350_c

rxn30127_c: cpd00290_c --> cpd00095_c + cpd00902_c

rxn30129_c: cpd00002_c + cpd00023_c + cpd00087_c <=> cpd00008_c + cpd00009_c + cpd00067_c + cpd06227_c

rxn30131_c: cpd00001_c --> cpd00080_c + cpd00098_c

rxn30136_c: cpd16880_c <=> cpd00001_c + cpd00930_c

rxn30137_c: cpd16857_c <=> cpd00001_c + cpd11471_c

rxn30140_c: cpd14939_c <=> cpd00001_c + cpd14940_c

rxn30161_c: cpd00002_c + cpd00041_c + cpd11909_c <=> cpd00012_c + cpd00018_c + cpd00067_c + cpd12226_c

rxn30163_c: cpd00002_c + cpd00066_c + cpd11919_c <=> cpd00012_c + cpd00018_c + cpd00067_c + cpd12335_c

rxn30164_c: cpd00002_c + cpd00039_c + cpd11917_c <=> cpd00012_c + cpd00018_c + cpd01326_c

rxn30165_c: cpd00002_c + cpd00060_c + cpd11918_c <=> cpd00012_c + cpd00018_c + cpd00067_c + cpd12105_c

rxn30166_c: cpd00002_c + cpd00161_c + cpd11922_c <=> cpd00012_c + cpd00018_c + 2.0 cpd00067_c + cpd12229_c

rxn30167_c: cpd00002_c + cpd00069_c + cpd11751_c <=> cpd00012_c + cpd00018_c + cpd00067_c + cpd12194_c

rxn30168_c: cpd00002_c + cpd00107_c + cpd11916_c <=> cpd00012_c + cpd00018_c + cpd00067_c + cpd12003_c

rxn30171_c: cpd00005_c + cpd00067_c + cpd11490_c <=> cpd00006_c + cpd16857_c

rxn30175_c: cpd00005_c + cpd00067_c + cpd14938_c <=> cpd00006_c + cpd14939_c

rxn30178_c: cpd02611_c <-- cpd00288_c

rxn30195_c: cpd00005_c + cpd00007_c + cpd00067_c <=> cpd00001_c + cpd00006_c + cpd08927_c

rxn30198_c: cpd00238_c + cpd00902_c <-- cpd00072_c + cpd00236_c

rxn30199_c: cpd00003_c + cpd00009_c + cpd00902_c <-- cpd00004_c + cpd00203_c

rxn30200_c: cpd00238_c + cpd00902_c <-- cpd00198_c + cpd19028_c

rxn30201_c: cpd00902_c <-- cpd00095_c

rxn30202_c: cpd00054_c + cpd29667_c <=> cpd00001_c + cpd00065_c + cpd00902_c

rxn30205_c: cpd00001_c + cpd00046_c --> cpd00367_c + cpd19028_c

rxn30216_c: <=> cpd00003_c

rxn30238_c: cpd00001_c + cpd29694_c <=> cpd00033_c + cpd00035_c

rxn30282_c: cpd15378_c -->

rxn30283_c: cpd15380_c <--

rxn30284_c: cpd04122_c -->

rxn30285_c: cpd15486_c <--

rxn30286_c: cpd01027_c <--

rxn30295_c: cpd00067_e <=> cpd00061_c + cpd00067_c

rxn30296_c: cpd00067_e <=> cpd00061_c + cpd00067_c

rxn30352_c: cpd00002_c + cpd00051_c + cpd11907_c <=> cpd00012_c + cpd00018_c + cpd00067_c + cpd12036_c

rxn30354_c: cpd00002_c + cpd00132_c + cpd11908_c <=> cpd00012_c + cpd00018_c + cpd00067_c + cpd12313_c

rxn30387_c: cpd00002_c + cpd00023_c + cpd11912_c <=> cpd00012_c + cpd00018_c + cpd00067_c + cpd12227_c

rxn30394_c: cpd00002_c + cpd00119_c + cpd11914_c <=> cpd00012_c + cpd00018_c + cpd00067_c + cpd12228_c

rxn30401_c: cpd00002_c + cpd00322_c + cpd11915_c <=> cpd00012_c + cpd00018_c + cpd00067_c + cpd12256_c

rxn30404_c: cpd00001_c + 0.01 cpd16885_c <=> cpd00067_c + 0.02 cpd00214_c + 0.03 cpd01080_c + 0.01 cpd01188_c + 0.015 cpd03847_c + 0.655 cpd15237_c + 0.01 cpd15240_c + 0.27 cpd15269_c

rxn30421_c: cpd00001_c + 0.005 cpd11624_c <=> cpd00067_c + 0.27 cpd00214_c + 0.5 cpd00507_c + 0.05 cpd01080_c + 0.02 cpd01107_c + 0.06 cpd01741_c + 0.1 cpd03847_c + 0.17 cpd15237_c + 0.24 cpd15269_c + 0.09 cpd15270_c

rxn30443_c: cpd00002_c + cpd00065_c + cpd11923_c <=> cpd00012_c + cpd00018_c + cpd00067_c + cpd12336_c

rxn30448_c: cpd00002_c + cpd00156_c + cpd11924_c <=> cpd00012_c + cpd00018_c + cpd00067_c + cpd12133_c

rxn30449_c: cpd00001_c + 0.01 cpd16896_c <=> cpd00067_c + 0.02 cpd00214_c + 0.03 cpd01080_c + 0.01 cpd03221_c + 0.015 cpd03847_c + 0.655 cpd15237_c + 0.01 cpd15240_c + 0.27 cpd15269_c

rxn30513_c: cpd00009_e <=> cpd00009_c + cpd00061_c

rxn30545_c: cpd00002_c + cpd01352_c --> cpd00008_c + cpd00067_c + cpd19245_c

rxn30557_c: 3.0 cpd00005_c + 3.0 cpd00007_c + cpd01334_c --> 3.0 cpd00006_c + cpd00047_c + cpd08347_c

rxn30558_c: cpd00005_c + cpd00007_c + cpd03630_c <=> cpd00006_c + cpd08670_c

rxn30559_c: cpd00005_c + cpd00007_c + cpd08672_c <=> cpd00006_c + cpd08673_c

rxn30560_c: cpd00005_c + cpd00007_c + cpd01835_c <=> cpd00006_c + cpd00872_c

rxn30561_c: cpd00005_c + cpd00007_c + cpd03624_c --> cpd00006_c + cpd11194_c

rxn30563_c: cpd00006_c + cpd00102_c --> cpd00005_c + cpd00169_c

rxn30564_c: cpd00007_c + cpd09441_c --> cpd00025_c + cpd03624_c

rxn30566_c: cpd00003_c + cpd00351_c --> cpd00004_c + cpd00024_c + cpd00039_c

rxn30567_c: cpd00006_c + cpd00351_c --> cpd00005_c + cpd00024_c + cpd00039_c

rxn30568_c: cpd00003_c + cpd00351_c --> cpd00004_c + cpd00023_c + cpd01046_c

rxn30571_c: cpd00022_c + cpd00123_c --> cpd00010_c + cpd01646_c

rxn30575_c: cpd27127_c + cpd27627_c <=> cpd00031_c + 2.0 cpd00067_c + cpd27628_c

rxn30581_c: cpd02503_c --> cpd00010_c + cpd00876_c

rxn30585_c: cpd00001_c + 2.0 cpd11658_c <=> cpd00179_c

rxn30586_c: cpd08625_c --> cpd09027_c

rxn30587_c: cpd01651_c --> cpd00013_c + cpd00041_c

rxn30588_c: cpd00925_c --> cpd00038_c + cpd00126_c

rxn30593_c: cpd00911_c --> cpd00284_c

rxn30597_c: cpd00438_c --> cpd00013_c + cpd03279_c

rxn30598_c: cpd00182_c --> cpd00013_c + cpd00246_c

rxn30605_c: cpd00388_c <=> cpd00011_c + cpd00013_c + cpd28773_c

rxn30608_c: cpd00003_c + cpd01046_c --> cpd00004_c + cpd00705_c

rxn30610_c: cpd00018_c --> cpd00013_c + cpd00114_c

rxn30614_c: cpd00002_c --> 2.0 cpd00009_c + cpd00018_c

rxn30622_c: cpd01720_c --> cpd00011_c + cpd00013_c + cpd00085_c

rxn30624_c: cpd03705_c --> cpd00008_c + cpd00018_c

rxn30625_c: cpd00242_c <-- cpd00011_c

rxn30626_c: cpd00616_c <-- cpd00011_c + cpd00066_c

rxn30629_c: cpd01236_c <=> cpd00999_c + cpd01449_c

rxn30630_c: cpd00274_c --> cpd00011_c + cpd00013_c + cpd00064_c

rxn30631_c: cpd00022_c + cpd00032_c --> cpd00010_c + cpd00137_c

rxn30636_c: cpd00654_c --> cpd00013_c + cpd00412_c

rxn30637_c: cpd00367_c --> cpd00013_c + cpd00249_c

rxn30642_c: cpd00004_c + 5.0 cpd00067_e + cpd11669_e <=> cpd00003_c + 4.0 cpd00067_c + cpd15291_c

rxn30644_c: 2.0 cpd00067_e + cpd15291_e + 2.0 cpd26784_e <=> 4.0 cpd00067_c + cpd11669_c + 2.0 cpd26785_c

rxn30650_c: cpd00356_c --> cpd00013_c + cpd00358_c

rxn30651_c: cpd00587_c --> cpd00013_c + cpd00020_c + cpd00239_c

rxn30653_c: cpd00020_c + cpd00346_c <-- cpd02120_c

rxn30655_c: cpd00282_c --> cpd00343_c

rxn30656_c: cpd00337_c --> cpd01720_c

rxn30662_c: cpd00002_c + cpd00056_c --> cpd00008_c + cpd00357_c

rxn30663_c: cpd00358_c --> cpd00012_c + cpd00299_c

rxn30664_c: cpd00007_c + cpd00059_c + cpd00909_c <=> cpd00011_c + cpd00150_c + cpd00335_c + cpd03956_c

rxn30668_c: cpd00003_c + cpd00055_c --> cpd00004_c + cpd00047_c

rxn30670_c: cpd00201_c --> cpd00047_c + cpd00087_c

rxn30671_c: cpd15454_c + cpd15581_c <-- cpd00056_c + cpd15582_c

rxn30672_c: cpd00780_c --> cpd00106_c + cpd00142_c

rxn30678_c: cpd00003_c + cpd00023_c --> cpd00004_c + cpd00013_c + cpd00024_c

rxn30679_c: cpd00006_c + cpd00023_c --> cpd00005_c + cpd00013_c + cpd00024_c

rxn30681_c: cpd00025_c + 2.0 cpd00042_c <-- cpd00111_c

rxn30685_c: cpd00003_c + cpd00229_c --> cpd00004_c + cpd00139_c

rxn30687_c: cpd02182_c --> cpd00042_c + cpd00221_c

rxn30688_c: cpd00002_c + cpd00053_c + cpd00497_c --> cpd00012_c + cpd00018_c + cpd00023_c + cpd00126_c

rxn30690_c: cpd00038_c --> cpd00047_c + cpd02978_c

rxn30691_c: cpd00038_c --> cpd00012_c + cpd00047_c + cpd00957_c

rxn30692_c: cpd00207_c --> cpd00013_c + cpd00309_c

rxn30693_c: cpd00311_c --> cpd00013_c + cpd01217_c

rxn30694_c: cpd02978_c --> cpd00012_c + cpd03521_c

rxn30695_c: cpd00028_c + cpd00350_c <=> cpd00012_c + cpd11313_c

rxn30696_c: cpd00003_c + cpd01324_c --> cpd00004_c + cpd00119_c

rxn30699_c: cpd01775_c --> cpd00012_c + cpd01777_c

rxn30700_c: cpd24599_c <-- cpd02800_c

rxn30701_c: cpd00022_c + cpd00279_c --> cpd00010_c + cpd00292_c

rxn30703_c: cpd00003_c + cpd02625_c --> cpd00004_c + cpd19042_c

rxn30704_c: 2.0 cpd00004_c + cpd00070_c + cpd00327_c <=> 2.0 cpd00003_c + cpd00010_c + cpd00011_c + cpd01393_c

rxn30705_c: 2.0 cpd00005_c + cpd00070_c + cpd00327_c <=> 2.0 cpd00006_c + cpd00010_c + cpd00011_c + cpd01393_c

rxn30707_c: cpd00956_c <-- cpd00011_c + cpd02210_c

rxn30709_c: cpd00003_c + cpd00114_c --> cpd00004_c + cpd00497_c

rxn30711_c: cpd00007_c + 2.0 cpd00486_c --> cpd00025_c + 2.0 cpd00703_c

rxn30714_c: cpd00004_c + cpd08615_c <-- cpd00003_c + cpd00113_c

rxn30715_c: cpd00005_c + cpd08615_c <-- cpd00006_c + cpd00113_c

rxn30720_c: cpd00007_c + 2.0 cpd00059_c <=> 2.0 cpd00335_c

rxn30721_c: cpd00025_c + cpd00059_c <-- cpd00335_c

rxn30724_c: cpd00003_c + cpd00334_c --> cpd00004_c + cpd00159_c

rxn30725_c: cpd00007_c + cpd00024_c + cpd02289_c <=> cpd00011_c + cpd00036_c + cpd03501_c

rxn30726_c: cpd02835_c --> cpd00091_c + cpd02930_c

rxn30728_c: cpd00022_c + cpd00040_c --> cpd00010_c + cpd00130_c

rxn30729_c: cpd00001_c + cpd11735_c <=> cpd19001_c

rxn30730_c: cpd00347_c --> cpd00201_c

rxn30735_c: cpd00007_c + cpd00121_c --> cpd00164_c

rxn30736_c: cpd00341_c --> cpd00011_c + cpd00013_c + cpd00118_c

rxn30739_c: cpd00004_c + cpd00209_c <=> cpd00003_c + cpd00075_c

rxn30740_c: cpd00355_c --> cpd00101_c + cpd00133_c

rxn30745_c: cpd00009_c + cpd00032_c <-- cpd00011_c + cpd00061_c

rxn30749_c: 2.0 cpd00338_c <-- cpd00689_c

rxn30750_c: cpd00007_c + cpd00791_c <=> cpd01476_c

rxn30751_c: cpd00012_c + cpd00023_c + cpd01982_c <-- cpd00053_c + cpd00103_c

rxn30754_c: cpd00095_c + cpd03470_c <-- cpd00009_c + cpd02333_c

rxn30755_c: cpd00007_c + cpd00041_c + cpd00095_c <=> cpd00009_c + cpd00025_c + cpd02333_c

rxn30756_c: cpd02791_c <=> cpd28787_c

rxn30757_c: cpd00003_c + cpd01088_c --> cpd00004_c + cpd03846_c

rxn30758_c: cpd01368_c --> cpd00013_c + cpd00400_c

rxn30764_c: cpd00957_c --> cpd00013_c + cpd00931_c

rxn30767_c: cpd00011_c + cpd00871_c --> 2.0 cpd00169_c

rxn30769_c: cpd00005_c + cpd00007_c + cpd00214_c --> cpd00006_c + cpd19486_c

rxn30770_c: cpd00005_c + cpd00007_c + cpd19486_c --> cpd00006_c + cpd05200_c

rxn30771_c: 2.0 cpd00005_c + cpd00007_c + cpd00536_c --> 2.0 cpd00006_c + cpd20869_c

rxn30772_c: cpd00005_c + cpd00007_c + cpd20869_c --> cpd00006_c + cpd20873_c

rxn30774_c: cpd00005_c + cpd00007_c + cpd00604_c --> cpd00006_c + cpd00881_c

rxn30775_c: cpd00005_c + cpd00007_c + cpd01059_c --> cpd00006_c + cpd03337_c

rxn30776_c: cpd00007_c + cpd00024_c + cpd03636_c <=> cpd00011_c + cpd00036_c + cpd08653_c

rxn30777_c: cpd00005_c + cpd00007_c + cpd01733_c --> cpd00006_c + cpd08984_c

rxn30778_c: cpd00075_c + 6.0 cpd28082_c <=> cpd00013_c + 6.0 cpd27757_c

rxn30779_c: cpd00007_c + cpd00024_c + cpd02249_c <=> cpd00011_c + cpd00036_c + cpd08668_c

rxn30781_c: cpd00003_c + cpd01733_c --> cpd00004_c + cpd01059_c

rxn30782_c: cpd00006_c + cpd01733_c --> cpd00005_c + cpd01059_c

rxn30786_c: cpd00005_c + cpd00007_c + cpd00065_c --> cpd00006_c + cpd00011_c + cpd01880_c

rxn30788_c: cpd01881_c --> cpd00013_c + cpd00703_c

rxn30791_c: cpd03467_c --> cpd00190_c + cpd24359_c

rxn30793_c: cpd00005_c + cpd02289_c <-- cpd00006_c + cpd06215_c

rxn30794_c: cpd00005_c + cpd03503_c <-- cpd00006_c + cpd03970_c

rxn30796_c: cpd00007_c + cpd00024_c + cpd08663_c <=> cpd00011_c + cpd00036_c + cpd08652_c

rxn30797_c: cpd00001_c + 2.0 cpd11735_c <=> cpd00665_c

rxn30798_c: cpd00010_c + cpd00333_c <-- cpd00422_c

rxn30800_c: cpd00401_c --> cpd00010_c + cpd00153_c

rxn30801_c: cpd00005_c + cpd00007_c + cpd00153_c --> cpd00006_c + cpd00599_c

rxn30802_c: cpd00401_c --> cpd23245_c

rxn30803_c: cpd23245_c --> cpd00010_c + cpd00599_c

rxn30804_c: cpd00006_c + cpd20870_c --> cpd00005_c + cpd20871_c

rxn30806_c: cpd00022_c + cpd00869_c --> cpd00010_c + cpd17400_c

rxn30807_c: cpd00005_c + cpd00007_c + cpd17403_c --> cpd00006_c + cpd00011_c + cpd17431_c

rxn30808_c: cpd00005_c + cpd00007_c + cpd01890_c --> cpd00006_c + cpd07322_c

rxn30809_c: cpd00005_c + cpd00007_c + cpd02595_c --> cpd00006_c + cpd00634_c

rxn30810_c: cpd00007_c + cpd00024_c + cpd08666_c <=> cpd00011_c + cpd00036_c + cpd08667_c

rxn30816_c: cpd00006_c + cpd05521_c <-- cpd00005_c + cpd00007_c + cpd05486_c

rxn30817_c: cpd00005_c + 3.0 cpd00070_c + cpd00192_c --> cpd00006_c + 4.0 cpd00010_c + 3.0 cpd00011_c + cpd05556_c

rxn30818_c: cpd00006_c + cpd05486_c <-- cpd00005_c + cpd00007_c + cpd03637_c

rxn30819_c: cpd00025_c + 2.0 cpd00059_c <=> 2.0 cpd00766_c

rxn30820_c: cpd00007_c + cpd00059_c <=> cpd00766_c

rxn30823_c: cpd00005_c + cpd00007_c + cpd01234_c --> cpd00006_c + cpd14531_c

rxn30824_c: cpd00005_c + cpd00007_c + cpd14522_c --> cpd00006_c + cpd14533_c

rxn30825_c: cpd00005_c + cpd00007_c + cpd14521_c --> cpd00006_c + cpd14532_c

rxn30828_c: cpd00005_c + cpd15130_c --> cpd00006_c + cpd15133_c

rxn30829_c: cpd00005_c + cpd15131_c --> cpd00006_c + cpd15134_c

rxn30830_c: cpd00005_c + cpd02870_c --> cpd00006_c + cpd16331_c

rxn30831_c: cpd15130_c --> cpd00009_c + cpd15131_c

rxn30832_c: cpd15133_c --> cpd00009_c + cpd15134_c

rxn30833_c: cpd15131_c --> cpd00009_c + cpd02870_c

rxn30834_c: cpd15134_c --> cpd00009_c + cpd16331_c

rxn30835_c: cpd02870_c --> cpd00009_c + cpd00278_c

rxn30837_c: cpd00278_c <-- cpd02526_c + cpd05267_c

rxn30838_c: cpd00278_c <-- cpd15136_c

rxn30839_c: cpd02526_c --> cpd00302_c

rxn30843_c: cpd00005_c + cpd00007_c + cpd00066_c --> cpd00006_c + cpd00011_c + cpd14796_c

rxn30845_c: cpd00015_c + cpd00278_c <-- cpd00182_c + cpd00982_c + cpd04535_c

rxn30849_c: cpd23795_c --> cpd00012_c + cpd08247_c

rxn30850_c: cpd00005_c + cpd00007_c + cpd08670_c <=> cpd00006_c + cpd08671_c

rxn30851_c: cpd00005_c + cpd00007_c + cpd00717_c --> cpd00006_c + cpd01135_c

rxn30853_c: cpd00005_c + cpd00007_c + cpd02762_c <=> cpd00006_c + cpd08629_c

rxn30854_c: cpd00005_c + cpd00007_c + cpd08629_c <=> cpd00006_c + cpd08630_c

rxn30855_c: cpd00005_c + cpd00007_c + cpd08630_c <=> cpd00006_c + cpd08631_c

rxn30857_c: cpd24351_c + cpd24563_c <=> cpd12494_c + cpd26919_c

rxn30858_c: cpd00005_c + cpd00026_c <-- cpd00006_c + cpd01481_c

rxn30861_c: cpd00005_c + cpd00007_c + cpd03218_c --> cpd00006_c + cpd05497_c

rxn30862_c: cpd00005_c + cpd00007_c + cpd05497_c --> cpd00006_c + cpd05508_c

rxn30864_c: cpd00007_c + cpd00024_c + cpd03503_c <=> cpd00011_c + cpd00036_c + cpd03502_c

rxn30866_c: cpd00347_c --> cpd02197_c

rxn30868_c: cpd00003_c + cpd01504_c --> cpd00004_c + cpd00085_c

rxn30869_c: cpd00006_c + cpd01504_c --> cpd00005_c + cpd00085_c

rxn30871_c: cpd03375_c --> cpd00010_c + cpd00745_c

rxn30876_c: cpd00005_c + cpd00007_c + cpd03633_c <=> cpd00006_c + cpd08651_c

rxn30877_c: cpd00007_c + cpd00024_c + cpd23358_c <=> cpd00011_c + cpd00036_c + cpd08666_c

rxn30885_c: cpd00005_c + cpd00007_c + cpd14525_c --> cpd00006_c + cpd14526_c

rxn30886_c: cpd00005_c + cpd00007_c + cpd14530_c --> cpd00006_c + cpd05719_c

rxn30905_c: cpd00007_c + cpd00024_c + cpd08657_c <=> cpd00011_c + cpd00036_c + cpd00067_c + cpd08660_c

rxn30906_c: cpd00007_c + cpd00024_c + cpd08660_c <=> 2.0 cpd00011_c + cpd00036_c + cpd23358_c

rxn30909_c: cpd00007_c + cpd00300_c --> cpd00011_c + cpd00025_c + cpd01092_c

rxn30910_c: cpd00005_c + cpd00007_c + cpd08656_c <=> cpd00006_c + cpd00011_c + cpd23358_c

rxn30911_c: cpd00005_c + cpd00007_c + cpd00398_c <=> cpd00006_c + cpd01047_c

rxn30912_c: cpd00005_c + cpd00007_c + cpd01047_c <=> cpd00006_c + cpd01071_c

rxn30913_c: cpd00005_c + cpd00007_c + cpd00398_c --> cpd00006_c + cpd03344_c

rxn30914_c: cpd00005_c + cpd00007_c + cpd03344_c <=> cpd00006_c + cpd01071_c

rxn30915_c: cpd00005_c + cpd00007_c + cpd01449_c <=> cpd00006_c + cpd16354_c

rxn30916_c: cpd00005_c + cpd00007_c + cpd16354_c <=> cpd00006_c + cpd16355_c

rxn30917_c: cpd00006_c + cpd03149_c <=> cpd00005_c + cpd00007_c + cpd19421_c

rxn30919_c: cpd00003_c + cpd00226_c --> cpd00004_c + cpd00309_c

rxn30923_c: cpd00005_c + cpd00007_c + cpd14523_c --> cpd00006_c + cpd14534_c

rxn30924_c: cpd00023_c + cpd02465_c --> cpd00024_c + cpd00504_c

rxn30925_c: cpd03148_c <=> cpd00999_c + cpd01449_c

rxn30926_c: cpd00007_c + cpd14534_c --> cpd14535_c

rxn30927_c: 2.0 cpd00007_c + cpd18004_c <=> cpd18005_c

rxn30929_c: cpd00007_c + cpd14537_c --> cpd14538_c

rxn30930_c: cpd00007_c + cpd14538_c --> cpd14539_c

rxn30931_c: cpd00005_c + cpd00007_c + cpd00160_c --> cpd00006_c + cpd03276_c

rxn30932_c: cpd00005_c + cpd00007_c + cpd01210_c --> cpd00006_c + cpd24744_c

rxn30933_c: cpd00005_c + cpd00007_c + cpd05717_c --> cpd00006_c + cpd24745_c

rxn30934_c: cpd00005_c + cpd00007_c + cpd24747_c --> cpd00006_c + cpd24746_c

rxn30935_c: cpd00004_c + cpd00007_c + cpd03637_c --> cpd00003_c + cpd05486_c

rxn30936_c: cpd00004_c + cpd00007_c + cpd05486_c --> cpd00003_c + cpd05521_c

rxn30939_c: cpd00006_c + cpd03333_c --> cpd00005_c + cpd00373_c

rxn30941_c: cpd03468_c <-- cpd03479_c

rxn30942_c: cpd00007_c + cpd00225_c --> cpd00025_c + cpd00153_c

rxn30943_c: cpd00007_c + cpd00670_c --> cpd00025_c + cpd00333_c

rxn30944_c: cpd00007_c + cpd09793_c --> cpd00025_c + cpd09794_c

rxn30946_c: cpd00007_c + cpd01061_c --> cpd00025_c + cpd15165_c

rxn30947_c: cpd00007_c + cpd10089_c --> cpd00025_c + cpd15608_c

rxn30952_c: cpd00007_c + 2.0 cpd25047_c <=> 2.0 cpd25044_c

rxn30954_c: cpd25046_c <-- cpd06158_c

rxn30957_c: cpd11574_c + cpd21090_c <=> cpd00018_c + cpd19503_c

rxn30958_c: cpd00084_c + cpd19503_c <=> cpd00035_c + cpd25050_c

rxn30960_c: cpd03435_c <=> cpd00999_c + cpd18004_c

rxn30962_c: cpd25102_c <=> 2.0 cpd00099_c + cpd00204_c

rxn30963_c: cpd25102_c <=> cpd00047_c + 2.0 cpd00099_c

rxn30964_c: cpd02016_c --> cpd00133_c + cpd05267_c

rxn30965_c: cpd00873_c --> cpd00101_c + cpd00218_c

rxn30969_c: cpd25529_c --> cpd00300_c

rxn30970_c: cpd00360_c --> cpd25530_c

rxn30975_c: cpd00038_c --> cpd00009_c + cpd00031_c

rxn30976_c: cpd00251_c --> cpd00018_c + cpd00101_c

rxn30977_c: cpd00007_c + cpd02083_c --> 2.0 cpd00011_c + cpd00791_c

rxn30980_c: 2.0 cpd00042_c + cpd01048_c <=> cpd00111_c + cpd04098_c

rxn30984_c: cpd00001_c + 6.0 cpd11735_c <=> cpd01329_c

rxn30989_c: cpd00052_c <-- cpd00356_c

rxn30990_c: cpd00062_c <-- cpd00358_c

rxn30991_c: cpd00002_c <-- cpd00115_c

rxn30992_c: cpd00038_c <-- cpd00241_c

rxn30993_c: cpd00004_c + cpd08615_c <-- cpd00003_c + cpd00202_c

rxn30994_c: cpd00005_c + cpd08615_c <-- cpd00006_c + cpd00202_c

rxn30995_c: cpd00003_c + cpd00309_c --> cpd00004_c + cpd00300_c

rxn30997_c: cpd00007_c + cpd08655_c <=> cpd03634_c

rxn30998_c: cpd01420_c --> cpd03637_c

rxn30999_c: cpd00005_c + cpd00007_c + cpd08673_c <=> cpd00006_c + cpd03633_c

rxn31001_c: cpd00007_c + cpd00024_c + cpd08658_c <=> cpd00011_c + cpd00036_c + cpd00067_c + cpd08659_c

rxn31002_c: cpd00007_c + cpd00024_c + cpd08661_c <=> cpd00011_c + cpd00036_c + cpd23358_c

rxn31003_c: cpd00007_c + cpd00024_c + cpd03634_c <=> cpd00011_c + cpd00036_c + cpd09080_c

rxn31013_c: cpd00007_c + cpd00024_c + cpd00717_c --> cpd00011_c + cpd00036_c + cpd19040_c

rxn31014_c: cpd00005_c + cpd00007_c + cpd00623_c --> cpd00006_c + cpd08926_c

rxn31015_c: cpd00005_c + cpd00007_c + cpd14514_c <=> cpd00006_c + cpd14515_c

rxn31016_c: cpd00025_c + cpd00363_c <=> cpd00071_c

rxn31017_c: cpd00004_c + cpd00007_c + cpd14644_c --> cpd00003_c + cpd25359_c

rxn31018_c: cpd00005_c + cpd00007_c + cpd14644_c --> cpd00006_c + cpd25359_c

rxn31019_c: cpd00004_c + cpd00007_c + cpd25359_c --> cpd00003_c + cpd25360_c

rxn31020_c: cpd00005_c + cpd00007_c + cpd25359_c --> cpd00006_c + cpd25360_c

rxn31023_c: cpd00004_c + cpd00007_c + cpd24475_c --> cpd00003_c + cpd25363_c

rxn31024_c: cpd00005_c + cpd00007_c + cpd24475_c --> cpd00006_c + cpd25363_c

rxn31025_c: cpd00004_c + cpd00007_c + cpd25363_c --> cpd00003_c + cpd25364_c

rxn31026_c: cpd00005_c + cpd00007_c + cpd25363_c --> cpd00006_c + cpd25364_c

rxn31029_c: cpd00005_c + cpd00007_c + cpd00559_c --> cpd00006_c + cpd00776_c

rxn31030_c: cpd00003_c + cpd00071_c --> cpd00004_c + cpd00029_c

rxn31031_c: cpd00005_c + cpd00007_c + cpd01188_c --> cpd00006_c + cpd24498_c

rxn31032_c: cpd00005_c + cpd00007_c + cpd24498_c <=> cpd00006_c + cpd24501_c

rxn31034_c: cpd00004_c + cpd00007_c + cpd03038_c --> cpd00003_c + cpd24503_c

rxn31035_c: cpd00005_c + cpd00007_c + cpd03038_c --> cpd00006_c + cpd24503_c

rxn31036_c: cpd00004_c + cpd00007_c + cpd24503_c --> cpd00003_c + cpd24504_c

rxn31037_c: cpd00005_c + cpd00007_c + cpd24503_c --> cpd00006_c + cpd24504_c

rxn31040_c: cpd00004_c + cpd00007_c + cpd03035_c --> cpd00003_c + cpd24505_c

rxn31041_c: cpd00005_c + cpd00007_c + cpd03035_c --> cpd00006_c + cpd24505_c

rxn31042_c: cpd00004_c + cpd00007_c + cpd24505_c <=> cpd00003_c + cpd24506_c

rxn31043_c: cpd00005_c + cpd00007_c + cpd24505_c <=> cpd00006_c + cpd24506_c

rxn31047_c: cpd00004_c + cpd00007_c + cpd02398_c --> cpd00003_c + cpd00857_c

rxn31048_c: cpd00005_c + cpd00007_c + cpd02398_c --> cpd00006_c + cpd00857_c

rxn31050_c: cpd00003_c + cpd02431_c --> cpd00004_c + cpd00023_c

rxn31051_c: cpd00006_c + cpd02431_c --> cpd00005_c + cpd00023_c

rxn31052_c: cpd01536_c --> cpd00126_c + cpd14655_c

rxn31055_c: cpd19036_c <-- cpd01642_c

rxn31056_c: cpd01642_c --> 2.0 cpd00009_c + cpd02054_c

rxn31068_c: cpd00022_c + cpd17401_c --> cpd00010_c + cpd17404_c

rxn31069_c: cpd00022_c + cpd17406_c --> cpd00010_c + cpd17408_c

rxn31070_c: cpd00022_c + cpd17410_c --> cpd00010_c + cpd17412_c

rxn31071_c: cpd00022_c + cpd17414_c --> cpd00010_c + cpd17416_c

rxn31072_c: cpd00022_c + cpd17418_c --> cpd00010_c + cpd17420_c

rxn31073_c: cpd00002_c + cpd00060_c --> cpd00009_c + cpd00012_c + cpd00017_c

rxn31074_c: cpd00759_c --> cpd00042_c + cpd00047_c

rxn31077_c: cpd00690_c --> cpd00098_c + cpd00373_c

rxn31080_c: cpd00003_c + cpd03526_c --> cpd00004_c + cpd03525_c

rxn31081_c: cpd00003_c + cpd00199_c --> cpd00004_c + cpd00036_c

rxn31082_c: cpd00006_c + cpd00199_c --> cpd00005_c + cpd00036_c

rxn31085_c: cpd00239_c + 6.0 cpd27757_c <=> cpd00081_c + 6.0 cpd28082_c

rxn31089_c: cpd00002_c + cpd00184_c <-- cpd00008_c + cpd00793_c

rxn31090_c: cpd00125_c + cpd00299_c <-- cpd00330_c + cpd00793_c

rxn31092_c: cpd00005_c + cpd00007_c + cpd00333_c --> cpd00006_c + cpd01224_c

rxn31093_c: cpd00005_c + cpd00007_c + cpd00333_c --> cpd00006_c + cpd00604_c

rxn31099_c: 2.0 cpd00003_c + cpd00026_c --> 2.0 cpd00004_c + cpd00144_c

rxn31101_c: cpd00073_c --> cpd00011_c + 2.0 cpd00013_c

rxn31102_c: cpd00465_c --> cpd00011_c + 2.0 cpd00013_c + cpd00040_c

rxn31158_c: <=> cpd00002_c

rxn31159_c: <=> cpd00008_c

rxn31176_c: <=> cpd00002_c

rxn31177_c: <=> cpd00008_c

rxn31193_c: <=> cpd00002_c

rxn31227_c: cpd00213_c + cpd00269_c <=> cpd00011_c + cpd16497_c

rxn31229_c: 2.0 cpd11735_c <=> cpd11657_c

rxn31230_c: cpd00009_c + cpd11735_c <=> cpd00089_c

rxn31231_c: cpd00001_c + cpd11657_c <=> cpd00665_c

rxn31233_c: cpd00001_c --> cpd02883_c

rxn31234_c: cpd00001_c --> cpd03648_c

rxn31236_c: cpd00413_c + cpd00449_c <=> cpd00010_c + cpd00067_c + cpd16497_c

rxn31244_c: cpd00002_c + cpd00023_c + cpd11911_c <=> cpd00012_c + cpd00018_c + cpd12828_c

rxn31246_c: cpd00067_c + cpd03220_c + cpd11610_c <=> cpd01420_c + cpd11609_c

rxn31248_c: cpd01625_c <-- cpd03461_c

rxn31251_c: cpd00067_c + cpd06227_c <=> cpd00087_c + cpd12759_c

rxn31280_c: cpd00665_c <=> cpd11735_c + cpd19001_c

rxn31314_c: cpd00387_c <=> cpd00008_c + cpd11735_c

rxn31315_c: 2.0 cpd00001_c + cpd11594_c <=> 2.0 cpd19001_c

rxn31316_c: cpd02234_c <=> cpd00010_c + cpd12836_c

rxn31317_c: cpd02234_c <=> cpd00010_c + cpd12836_c

rxn31318_c: cpd02234_c <=> cpd00010_c + cpd12836_c

rxn31330_c: cpd00004_c + 0.5 cpd00007_c + 2.4 cpd00008_c + 2.4 cpd00009_c + cpd00067_c <-- cpd00001_c + 2.4 cpd00002_c + cpd00003_c

rxn31331_c: 0.5 cpd00007_c + 1.5 cpd00008_c + 1.5 cpd00009_c + cpd00982_c <-- cpd00001_c + 1.5 cpd00002_c + cpd00015_c

rxn31334_c: cpd00028_c <=> cpd11313_c

rxn31337_c: cpd03218_c --> cpd05497_c

rxn31338_c: cpd05497_c --> cpd05508_c

rxn31339_c: cpd01420_c --> cpd05498_c

rxn31340_c: cpd05498_c --> cpd03637_c

rxn31341_c: cpd05499_c --> cpd14689_c

rxn31342_c: cpd05490_c --> cpd14691_c

rxn31343_c: cpd14689_c --> cpd14692_c

rxn31344_c: cpd14691_c --> cpd05487_c

rxn31345_c: cpd00007_c + cpd14919_c <=> cpd00001_c + cpd00127_c

rxn31347_c: 2.0 cpd00017_c + 2.0 cpd00074_c + cpd11470_c <=> 2.0 cpd00060_c + 2.0 cpd03091_c + cpd14956_c

rxn31352_c: cpd03218_c --> cpd19175_c

rxn31353_c: cpd19175_c --> cpd05508_c

rxn31355_c: cpd00289_c <-- cpd03205_c

rxn31388_c: 2.0 cpd00001_c + 2.0 cpd00073_c --> 2.0 cpd00011_c + 3.0 cpd00013_c

rxn31390_c: 2.0 cpd00067_c + cpd02469_c <-- cpd00020_c + cpd00204_c

rxn31393_c: cpd00004_c + cpd00067_c + cpd11609_c <=> cpd00003_c + cpd11610_c

rxn31399_c: cpd00025_c + cpd00059_c --> cpd00001_c + 2.0 cpd00067_c + cpd00335_c

rxn31402_c: cpd00075_c + cpd11620_c <=> cpd00001_c + cpd00013_c + cpd11621_c

rxn31403_c: cpd00075_c + cpd11620_c <=> cpd00001_c + cpd00013_c + cpd11621_c

rxn31405_c: 2.0 cpd00007_c + cpd00080_c --> cpd00025_c + cpd00095_c

rxn31409_c: cpd00081_c + cpd11620_c <=> cpd00001_c + cpd00239_c + cpd11621_c

rxn31413_c: cpd00120_c + cpd11609_c <=> cpd00650_c + cpd11610_c

rxn31415_c: 2.0 cpd00007_c + cpd00024_c + cpd00129_c --> cpd00011_c + cpd00036_c + cpd00851_c

rxn31418_c: 16.0 cpd00100_c + 33.0 cpd19000_c <=> 33.0 cpd00067_c + 19.0 cpd11677_c

rxn31421_c: 2.0 cpd00067_c + cpd02605_c <-- cpd00200_c + cpd00204_c

rxn31425_c: 2.0 cpd00005_c + 27.0 cpd00067_c + 9.0 cpd00292_c <-- 2.0 cpd00006_c + 9.0 cpd00010_c + 2.0 cpd00332_c

rxn31427_c: cpd00001_c + cpd11657_c <=> cpd00665_c + cpd11976_c

rxn31429_c: cpd00109_c + cpd15291_c <=> 2.0 cpd00067_c + cpd00110_c + cpd11669_c

rxn31430_c: 9.0 cpd00002_c + 2.0 cpd00332_c --> 9.0 cpd00008_c + 11.0 cpd00067_c + 9.0 cpd00812_c

rxn31432_c: 2.0 cpd00001_c + 2.0 cpd00002_c + cpd00401_c + cpd11610_c <=> 2.0 cpd00009_c + cpd03763_c + cpd11609_c

rxn31438_c: cpd00001_c + cpd01078_c + cpd11621_c <=> cpd03387_c + cpd11620_c

rxn31440_c: 65.0 cpd00007_c + 84.0 cpd00024_c + 65.0 cpd01390_c <=> 65.0 cpd00011_c + 65.0 cpd00036_c + 70.0 cpd03636_c

rxn31442_c: 2.0 cpd00025_c + cpd01468_c <-- cpd00001_c + cpd00007_c + cpd00067_c + cpd19044_c

rxn31446_c: cpd01882_c + cpd11609_c <=> cpd01966_c + cpd11610_c

rxn31447_c: 2.0 cpd00067_c + 3.0 cpd06227_c <=> 3.0 cpd00087_c + cpd12797_c

rxn31448_c: cpd00007_c + cpd00024_c + cpd02289_c <=> cpd00001_c + cpd00011_c + cpd00036_c + 3.0 cpd00067_c + cpd03501_c

rxn31449_c: cpd03281_c <-- cpd00204_c + cpd03106_c

rxn31455_c: 3.0 cpd00001_c + cpd12848_c <=> cpd03293_c + cpd11608_c

rxn31456_c: cpd03330_c <-- cpd00204_c + cpd03308_c

rxn31457_c: 7.0 cpd00067_c + 6.0 cpd03346_c <=> 5.0 cpd03463_c

rxn31460_c: 18.0 cpd00067_c + 15.0 cpd11464_c + 4.0 cpd11492_c <=> 4.0 cpd00011_c + 4.0 cpd11486_c + 15.0 cpd11493_c

rxn31461_c: 18.0 cpd00067_c + 15.0 cpd11464_c + 4.0 cpd11492_c <=> 4.0 cpd00011_c + 4.0 cpd11486_c + 15.0 cpd11493_c

rxn31465_c: cpd00007_c + cpd00024_c + cpd03503_c <=> cpd00001_c + cpd00011_c + cpd00036_c + 3.0 cpd00067_c + cpd03502_c

rxn31467_c: 28.0 cpd15070_c <=> 25.0 cpd05548_c

rxn31468_c: cpd00665_c <=> cpd00027_c + cpd11735_c

rxn31469_c: cpd00665_c <=> cpd00027_c + cpd11735_c

rxn31472_c: cpd04044_c <-- cpd00204_c + cpd04045_c

rxn31485_c: cpd00037_c + cpd12559_c <=> cpd00014_c + cpd11675_c

rxn31486_c: cpd00037_c + cpd12559_c <=> cpd00014_c + cpd11675_c

rxn31505_c: 70.0 cpd00007_c + 89.0 cpd00024_c + 70.0 cpd08661_c <=> 70.0 cpd00011_c + 70.0 cpd00036_c + 75.0 cpd08662_c

rxn31506_c: 70.0 cpd00007_c + 89.0 cpd00024_c + 70.0 cpd08661_c <=> 70.0 cpd00011_c + 70.0 cpd00036_c + 75.0 cpd08663_c

rxn31507_c: 65.0 cpd00007_c + 84.0 cpd00024_c + 65.0 cpd08662_c <=> 65.0 cpd00011_c + 65.0 cpd00036_c + 70.0 cpd08666_c

rxn31516_c: cpd09805_c --> cpd09806_c

rxn31519_c: 3.0 cpd00007_c + 2.0 cpd11255_c --> 4.0 cpd00047_c + 2.0 cpd00067_c + 3.0 cpd00204_c + 2.0 cpd05191_c

rxn31521_c: cpd00007_c + cpd00098_c + cpd11620_c <=> cpd00001_c + cpd00447_c + cpd11621_c

rxn31525_c: 8.0 cpd07395_c <-- 11.0 cpd00067_c + 11.0 cpd00639_c

rxn31526_c: 8.0 cpd07395_c <-- 11.0 cpd00067_c + 11.0 cpd00639_c

rxn31527_c: 8.0 cpd15070_c <=> 11.0 cpd05548_c

rxn31529_c: cpd00017_c + 2.0 cpd00074_c + cpd11470_c <=> cpd00060_c + cpd00067_c + cpd03091_c + cpd14956_c

rxn31531_c: cpd14533_c --> cpd14536_c

rxn31537_c: 2.0 cpd00289_c <-- 2.0 cpd00067_c + cpd03205_c

rxn31543_c: 6.0 cpd00067_c + 6.0 cpd15186_c <=> 7.0 cpd00077_c

rxn31546_c: 12.0 cpd17281_c <-- 25.0 cpd00067_c + 14.0 cpd17282_c

rxn31586_c: 0.378 cpd00023_c + 0.74 cpd00033_c + 0.624 cpd00035_c + 0.38 cpd00039_c + 0.417 cpd00041_c + 0.319 cpd00051_c + 0.529 cpd00054_c + 0.372 cpd00060_c + 0.272 cpd00065_c + 0.336 cpd00066_c + 0.307 cpd00069_c + 0.423 cpd00107_c + 0.358 cpd00119_c + 0.483 cpd00129_c + 0.474 cpd00156_c + 0.467 cpd00161_c + 0.423 cpd00322_c + 0.231 cpd00381_c <=> cpd11612_c

rxn31590_c: cpd00100_c + cpd00536_c <-- 3.0 cpd00001_c + cpd23060_c

rxn31591_c: cpd00100_c + cpd00214_c <-- 3.0 cpd00001_c + cpd26606_c

rxn31601_c: 1.527 cpd00023_c + 0.044 cpd00033_c + 1.153 cpd00035_c + 5.7e-05 cpd00039_c + 0.414 cpd00041_c + 0.096 cpd00051_c + 0.612 cpd00054_c + 0.123 cpd00060_c + 0.004 cpd00065_c + 0.313 cpd00066_c + 0.244 cpd00069_c + 1.509 cpd00107_c + 0.091 cpd00119_c + 0.762 cpd00129_c + 0.249 cpd00156_c + 0.175 cpd00161_c + 0.464 cpd00322_c + 0.031 cpd00381_c <=> cpd11463_c

rxn31607_c: cpd00013_c + cpd00047_c + cpd03303_c + cpd11610_c <=> 3.0 cpd00001_c + cpd02311_c + cpd11609_c

rxn31646_c: cpd00008_c + cpd00020_c <-- cpd00011_c + cpd00071_c

rxn31647_c: cpd00001_c + cpd00003_c + cpd11616_c <=> cpd00004_c + 2.0 cpd00008_c + cpd00049_c

rxn31648_c: cpd00008_c + cpd11625_c <=> cpd00011_c + cpd11616_c

rxn31649_c: cpd00002_c + cpd00072_c --> 3.0 cpd00008_c + cpd00290_c

rxn31650_c: cpd00002_c + cpd00082_c --> 3.0 cpd00008_c + cpd00072_c

rxn31651_c: cpd00012_c + cpd00072_c --> cpd00008_c + cpd00009_c + cpd00290_c

rxn31652_c: cpd00062_c + cpd00072_c --> cpd00008_c + cpd00014_c + cpd00290_c

rxn31653_c: cpd00068_c + cpd00072_c --> cpd00008_c + cpd00090_c + cpd00290_c

rxn31654_c: cpd00068_c + cpd00082_c --> cpd00008_c + cpd00072_c + cpd00090_c

rxn31655_c: cpd00001_c + cpd11624_c <=> cpd00008_c + cpd00457_c + cpd19004_c

rxn31656_c: cpd00008_c + cpd00143_c <-- cpd00011_c + cpd00464_c

rxn31657_c: cpd00003_c + cpd00449_c --> cpd00004_c + cpd00008_c + cpd00213_c

rxn31658_c: cpd00002_c + cpd00238_c --> 3.0 cpd00008_c + cpd00349_c

rxn31661_c: cpd00004_c + cpd00007_c + cpd00008_c + cpd00374_c <-- cpd00001_c + cpd00003_c + cpd02357_c

rxn31664_c: cpd00008_c + cpd00483_c <-- cpd00011_c + cpd01360_c

rxn31665_c: cpd00001_c + cpd00007_c + cpd00486_c --> cpd00008_c + cpd00025_c + cpd00703_c

rxn31666_c: cpd00002_c + cpd00010_c + cpd00489_c --> cpd00008_c + cpd00012_c + cpd00018_c + cpd03165_c

rxn31669_c: cpd00005_c + cpd00007_c + 2.0 cpd00008_c + cpd00542_c <=> cpd00001_c + cpd00006_c + cpd12754_c

rxn31670_c: cpd00150_c + cpd00547_c <=> cpd00008_c + cpd00239_c + cpd01651_c

rxn31671_c: cpd00001_c + cpd11715_c <=> cpd00008_c + cpd00867_c + cpd19004_c

rxn31672_c: cpd00043_c + cpd00878_c <=> cpd00008_c + cpd00014_c + cpd00947_c

rxn31673_c: cpd00006_c + cpd02905_c --> cpd00005_c + cpd00008_c + cpd00903_c

rxn31677_c: cpd00003_c + cpd02691_c --> cpd00004_c + cpd00008_c + cpd02124_c

rxn31678_c: cpd00001_c + cpd12414_c <=> cpd00008_c + cpd11615_c + cpd12338_c

rxn31680_c: cpd00006_c + 3.0 cpd00008_c + cpd11722_c <=> cpd00005_c + cpd12566_c

rxn31681_c: cpd00002_c + cpd00242_c + cpd12848_c <=> 3.0 cpd00008_c + cpd00009_c + cpd12543_c

rxn31682_c: 2.0 cpd00001_c + cpd00003_c + cpd02625_c --> cpd00004_c + cpd00008_c + cpd19042_c

rxn31683_c: 2.0 cpd00001_c + cpd00006_c + cpd02625_c --> cpd00005_c + cpd00008_c + cpd19042_c

rxn31684_c: cpd00003_c + cpd03231_c --> cpd00004_c + cpd00008_c + cpd03232_c

rxn31690_c: cpd00005_c + cpd00007_c + cpd00008_c + cpd03441_c <-- cpd00001_c + cpd00006_c + cpd03442_c

rxn31691_c: cpd00008_c + cpd03443_c <-- cpd00011_c + cpd03444_c

rxn31692_c: cpd00005_c + cpd00007_c + cpd03446_c --> cpd00001_c + cpd00006_c + cpd00008_c + cpd03447_c

rxn31698_c: cpd00003_c + cpd04003_c --> cpd00004_c + cpd00008_c + cpd04004_c

rxn31699_c: cpd00001_c + cpd00006_c + cpd00075_c + cpd00364_c <=> cpd00005_c + cpd00007_c + cpd00008_c + cpd00646_c

rxn31700_c: cpd00003_c + cpd00010_c + cpd03957_c --> cpd00004_c + cpd00008_c + cpd00022_c

rxn31701_c: cpd00003_c + cpd04368_c --> cpd00004_c + cpd00008_c + cpd04369_c

rxn31702_c: cpd00004_c + cpd00007_c + cpd00008_c + cpd03480_c <-- cpd00001_c + cpd00003_c + cpd00426_c

rxn31703_c: cpd00002_c + cpd00010_c + cpd04386_c --> cpd00008_c + cpd00012_c + cpd00018_c + cpd04390_c

rxn31704_c: cpd00004_c + cpd00007_c + cpd00008_c + cpd19069_c <-- cpd00001_c + cpd00003_c + cpd03480_c

rxn31705_c: cpd00005_c + cpd00007_c + 3.0 cpd00008_c + cpd00015_c + cpd04358_c <-- cpd00001_c + cpd00006_c + cpd00982_c + cpd01413_c

rxn31706_c: cpd00001_c + cpd02864_c --> cpd00008_c + cpd03770_c

rxn31708_c: cpd00008_c + cpd00052_c + cpd08286_c <-- cpd00012_c + cpd08287_c

rxn31709_c: cpd00006_c + cpd03038_c --> cpd00005_c + cpd00008_c + cpd08302_c

rxn31710_c: cpd00006_c + cpd00877_c --> cpd00005_c + cpd00008_c + cpd03222_c

rxn31716_c: cpd00001_c + cpd09317_c --> cpd00008_c + cpd01498_c

rxn31717_c: cpd00005_c + cpd00007_c + cpd00008_c + cpd00415_c <-- cpd00001_c + cpd00006_c + cpd01822_c

rxn31718_c: cpd00004_c + cpd00007_c + cpd00008_c + cpd09785_c <-- cpd00001_c + cpd00003_c + cpd09792_c

rxn31719_c: cpd00004_c + cpd00007_c + cpd00008_c + cpd09801_c <-- cpd00001_c + cpd00003_c + cpd01859_c

rxn31720_c: cpd00003_c + cpd09844_c --> cpd00004_c + cpd00008_c + cpd01507_c

rxn31721_c: cpd00002_c + cpd00010_c + cpd03642_c --> cpd00008_c + cpd00012_c + cpd00018_c + cpd09842_c

rxn31722_c: cpd00008_c + cpd00191_c <-- cpd00011_c + cpd00071_c

rxn31723_c: cpd00006_c + cpd09879_c --> cpd00005_c + cpd00008_c + cpd00759_c

rxn31724_c: cpd05499_c --> cpd19170_c

rxn31725_c: cpd19170_c --> cpd14692_c

rxn31726_c: 2.0 cpd00002_c + cpd00013_c + cpd00242_c --> 3.0 cpd00008_c + cpd00009_c + cpd00146_c

rxn31728_c: cpd15185_c <=> cpd00008_c + cpd15176_c

rxn31729_c: cpd00005_c + cpd00007_c + cpd00065_c --> cpd00001_c + cpd00006_c + cpd00008_c + cpd00011_c + cpd01880_c

rxn31730_c: cpd00002_c + cpd00054_c + cpd16440_c <=> 2.0 cpd00008_c + cpd00012_c + cpd00018_c + cpd15565_c

rxn31731_c: cpd00006_c + cpd00098_c --> cpd00005_c + cpd00008_c + cpd00447_c

rxn31732_c: cpd00008_c + cpd00359_c <-- cpd00703_c

rxn31733_c: cpd00001_c + cpd12531_c <=> cpd00008_c + cpd00912_c + cpd12208_c

rxn31734_c: cpd00008_c + cpd00426_c + cpd02172_c <-- cpd00011_c + cpd00012_c + cpd17691_c

rxn31774_c: cpd00001_c + cpd23795_c --> cpd00012_c + cpd01062_c

rxn31782_c: cpd03148_c <=> cpd03435_c

rxn31835_c: cpd00026_c <=> cpd00014_c + cpd11746_c

rxn31840_c: cpd00026_c <=> cpd00014_c + cpd11746_c

rxn32016_c: <=> cpd00008_c

rxn32017_c: <=> cpd00008_c

rxn32128_c: cpd00005_c + cpd00067_c + cpd12227_c <=> cpd00006_c + cpd02345_c + cpd11912_c

rxn32129_c: cpd00820_c + 2.0 cpd26784_c <=> cpd00059_c + 2.0 cpd00067_c + 2.0 cpd00110_c

rxn32254_c: cpd00027_c + cpd11735_c <=> cpd00179_c

rxn32256_c: cpd00002_c + cpd00023_c + cpd11912_c <=> cpd00012_c + cpd00018_c + cpd00067_c + cpd12227_c

rxn32331_c: cpd25076_c --> cpd00001_c + cpd25077_c

rxn32332_c: cpd25076_c --> cpd00001_c + cpd25077_c

rxn32348_c: cpd00044_c + cpd16333_c <-- cpd00045_c + cpd24359_c

rxn32368_c: cpd00221_c + 2.0 cpd26784_c <=> cpd00020_c + 2.0 cpd00067_c + 2.0 cpd00110_c

rxn32391_c: cpd00001_c + cpd01059_c <=> 2.0 cpd00067_c + cpd18068_c

rxn32393_c: cpd00001_c + cpd01059_c <=> 2.0 cpd00067_c + cpd18068_c

rxn32394_c: cpd00001_c + cpd01059_c <=> 2.0 cpd00067_c + cpd18068_c

rxn32442_c: cpd00113_c + cpd23795_c --> cpd00012_c + cpd00350_c

rxn32448_c: cpd00083_c + cpd11619_c <=> cpd00031_c + cpd24353_c

rxn32453_c: cpd00083_c + cpd11619_c <=> cpd00031_c + cpd24353_c

rxn32457_c: cpd00083_c + cpd11619_c <=> cpd00031_c + cpd24353_c

rxn32461_c: cpd00083_c + cpd11619_c <=> cpd00031_c + cpd24353_c

rxn32574_c: <=> cpd00004_c

rxn32575_c: <=> cpd00004_c

rxn32576_c: <=> cpd00004_c

rxn32577_c: <=> cpd00004_c

rxn32582_c: <=> cpd00004_c

rxn32584_c: <=> cpd00004_c

rxn32597_c: cpd25075_c --> cpd00001_c + cpd25077_c

rxn32601_c: cpd25075_c --> cpd00001_c + cpd25077_c

rxn32746_c: <=> cpd00002_c

rxn32858_c: cpd18068_c <=> cpd00001_c + cpd01209_c

rxn32864_c: cpd18068_c <=> cpd00001_c + cpd01209_c

rxn32866_c: cpd18068_c <=> cpd00001_c + cpd01209_c

rxn32870_c: cpd18068_c <=> cpd00001_c + cpd01209_c

rxn32927_c: cpd00001_c + cpd24359_c --> cpd00027_c + cpd00048_c + cpd00239_c + cpd01881_c

rxn32937_c: cpd00007_c + 4.0 cpd00067_c + 4.0 cpd00110_c <=> 2.0 cpd00001_c + 4.0 cpd26784_c

rxn33179_c: <=> cpd00061_c

rxn33183_c: <=> cpd00061_c

rxn33185_c: cpd00001_c + cpd11746_c <=> cpd00190_c

rxn33186_c: <=> cpd00005_c

rxn33188_c: cpd00001_c + cpd11746_c <=> cpd00190_c

rxn33189_c: <=> cpd00005_c

rxn33190_c: <=> cpd00005_c

rxn33192_c: <=> cpd00005_c

rxn33196_c: <=> cpd00005_c

rxn33197_c: <=> cpd00005_c

rxn33261_c: cpd00001_c + cpd00081_c + 2.0 cpd26784_c <=> cpd00048_c + 2.0 cpd00067_c + 2.0 cpd00110_c

rxn33263_c: cpd00001_c + cpd00081_c + 2.0 cpd26784_c <=> cpd00048_c + 2.0 cpd00067_c + 2.0 cpd00110_c

rxn33289_c: cpd00001_c + cpd24353_c <=> cpd00138_c + cpd11619_c

rxn33312_c: cpd00001_c + cpd24353_c <=> cpd00138_c + cpd11619_c

rxn33317_c: cpd00001_c + cpd24353_c <=> cpd00138_c + cpd11619_c

rxn33348_c: cpd00059_c + 2.0 cpd26784_c <=> 2.0 cpd00067_c + 2.0 cpd00110_c + cpd00335_c

rxn33773_c: <=> cpd00006_c

rxn33781_c: <=> cpd00006_c

rxn33783_c: <=> cpd00006_c

rxn33784_c: <=> cpd00006_c

rxn33786_c: <=> cpd00006_c

rxn33788_c: <=> cpd00006_c

rxn33812_c: <=> cpd00003_c

rxn33813_c: <=> cpd00003_c

rxn33817_c: <=> cpd00003_c

rxn33824_c: <=> cpd00003_c

rxn33825_c: <=> cpd00003_c

rxn33850_c: cpd00002_c + cpd00020_c + cpd00242_c <-- cpd00008_c + cpd00032_c

rxn33853_c: cpd00022_c + cpd00032_c --> cpd00010_c + cpd00137_c

rxn33854_c: cpd00002_c + cpd00010_c + cpd00036_c <-- cpd00008_c + cpd00078_c

rxn33855_c: cpd00010_c + cpd00036_c + cpd00038_c <-- cpd00031_c + cpd00078_c

rxn33871_c: cpd00002_c + cpd00009_c + cpd00020_c --> cpd00008_c + cpd00012_c + cpd00061_c

rxn33872_c: cpd00002_c + cpd00009_c + cpd00020_c --> cpd00008_c + cpd00012_c + cpd00061_c

rxn33875_c: cpd00011_c + cpd00871_c --> 2.0 cpd00169_c

rxn33888_c: cpd00008_c + 4.67 cpd00067_c --> cpd00002_c

rxn33892_c: 2.0 cpd00001_c + cpd00002_c --> 2.0 cpd00012_c + cpd00018_c

rxn33894_c: 50.0 cpd00002_c + 50.0 cpd00089_c <=> 50.0 cpd00008_c + 50.0 cpd00012_c + cpd11657_c

rxn33898_c: 7.0 cpd00002_c + 14.0 cpd00005_c + 8.0 cpd00022_c + 14.0 cpd00067_c --> 6.0 cpd00001_c + 14.0 cpd00006_c + 7.0 cpd00008_c + 7.0 cpd00009_c + 8.0 cpd00010_c + cpd00214_c

rxn33905_c: cpd00002_c + 2.0 cpd00005_c + cpd00022_c + 3.0 cpd00067_c + cpd00242_c --> cpd00001_c + 2.0 cpd00006_c + cpd00008_c + cpd00009_c + cpd00010_c + cpd00011_c

rxn33906_c: cpd00002_c + 2.0 cpd00005_c + cpd00022_c + 3.0 cpd00067_c + cpd00242_c --> cpd00001_c + 2.0 cpd00006_c + cpd00008_c + cpd00009_c + cpd00010_c + cpd00011_c

rxn33912_c: cpd00002_c + cpd00010_c <-- cpd00012_c + cpd00018_c

rxn33951_c: cpd00002_c + cpd00253_c + cpd19181_c --> cpd00012_c + cpd00018_c + cpd00023_c + cpd15142_c

rxn33969_c: cpd00001_c + cpd00038_c <-- cpd00047_c + cpd03521_c

rxn34005_c: 2.0 cpd00042_c + cpd00044_c <-- cpd00018_c + cpd00081_c + cpd00111_c

rxn34007_c: cpd00239_c + 3.0 cpd11621_c <=> cpd00081_c + 3.0 cpd11620_c

rxn34008_c: 3.0 cpd00005_c + 3.0 cpd00067_c + cpd00081_c <-- 3.0 cpd00006_c + cpd00239_c

rxn34012_c: 3.5 cpd00007_c + 2.0 cpd00067_c + cpd00755_c <=> 6.0 cpd00001_c + 6.0 cpd00011_c + cpd01476_c

rxn34064_c: cpd00001_c + cpd00002_c + cpd00253_c + cpd00497_c <-- cpd00012_c + cpd00018_c + cpd00126_c

rxn34085_c: cpd00009_e <=> cpd00009_c + cpd00061_c

rxn34104_c: cpd00002_c + cpd15141_e <-- cpd00008_c + cpd15141_c

rxn34105_c: cpd00002_c + cpd15141_e <-- cpd00008_c + cpd15141_c

rxn34107_c: cpd00002_c + cpd01003_e <-- cpd00008_c + cpd01003_c

rxn34108_c: cpd00002_c + cpd00033_e <-- cpd00008_c + cpd00033_c

rxn34109_c: cpd00002_c + cpd00033_e <-- cpd00008_c + cpd00033_c

rxn34112_c: cpd00002_c + cpd19181_e <-- cpd00008_c + cpd19181_c

rxn34113_c: cpd00002_c + cpd19181_e <-- cpd00008_c + cpd19181_c

rxn34114_c: cpd00002_c + cpd00253_e <-- cpd00008_c + cpd00253_c

rxn34115_c: cpd00002_c + cpd00253_e <-- cpd00008_c + cpd00253_c

rxn34116_c: cpd00002_c + cpd00253_e <-- cpd00008_c + cpd00253_c

rxn34117_c: cpd00002_c + cpd00253_e <-- cpd00008_c + cpd00253_c

rxn34119_c: cpd00002_c + cpd00065_e <-- cpd00008_c + cpd00065_c

rxn34120_c: cpd00002_c + cpd15143_e <-- cpd00008_c + cpd15143_c

rxn34121_c: cpd00002_c + cpd15143_e <-- cpd00008_c + cpd15143_c

rxn34122_c: cpd00002_c + cpd00161_e <-- cpd00008_c + cpd00161_c

rxn34123_c: cpd00002_c + cpd15139_e <-- cpd00008_c + cpd15139_c

rxn34124_c: cpd00002_c + cpd15139_e <-- cpd00008_c + cpd15139_c

rxn34125_c: cpd00002_c + cpd15140_e <-- cpd00008_c + cpd15140_c

rxn34126_c: cpd00002_c + cpd15140_e <-- cpd00008_c + cpd15140_c

rxn34128_c: cpd00002_c + cpd01400_e <-- cpd00008_c + cpd01400_c

rxn34129_c: cpd00002_c + cpd01400_e <-- cpd00008_c + cpd01400_c

rxn34130_c: cpd00002_c + cpd00069_e <-- cpd00008_c + cpd00069_c

rxn34131_c: cpd00002_c + cpd00069_e <-- cpd00008_c + cpd00069_c

rxn34132_c: cpd00002_c + cpd19182_e <-- cpd00008_c + cpd19182_c

rxn34133_c: cpd00002_c + cpd19182_e <-- cpd00008_c + cpd19182_c

rxn34134_c: cpd00002_c + cpd00572_e <-- cpd00008_c + cpd00572_c

rxn34135_c: cpd00002_c + cpd00572_e <-- cpd00008_c + cpd00572_c

rxn34136_c: cpd00002_c + cpd19021_e <-- cpd00008_c + cpd19021_c

rxn34137_c: cpd00002_c + cpd19021_e <-- cpd00008_c + cpd19021_c

rxn34160_c: <=> cpd00002_c + cpd00008_c

rxn34161_c: <=> cpd00008_c + cpd00038_c

rxn34162_c: <=> cpd00008_c + cpd00038_c

rxn34165_c: cpd00052_e <=> cpd00008_c + cpd00052_c

rxn34166_c: cpd00052_e <=> cpd00008_c + cpd00052_c

rxn34167_c: cpd00115_e <=> cpd00008_c + cpd00115_c

rxn34168_c: cpd00115_e <=> cpd00008_c + cpd00115_c

rxn34169_c: cpd00241_e <=> cpd00008_c + cpd00241_c

rxn34170_c: cpd00241_e <=> cpd00008_c + cpd00241_c

rxn34171_c: cpd00358_e <=> cpd00008_c + cpd00358_c

rxn34172_c: cpd00358_e <=> cpd00008_c + cpd00358_c

rxn34173_c: cpd00356_e <=> cpd00008_c + cpd00356_c

rxn34174_c: cpd00356_e <=> cpd00008_c + cpd00356_c

rxn34197_c: 3.0 cpd00067_e <=> cpd00002_c + cpd00008_c + 3.0 cpd00067_c

rxn34198_c: 3.0 cpd00067_e <=> cpd00002_c + cpd00008_c + 3.0 cpd00067_c

rxn34199_c: 3.0 cpd00067_e <=> cpd00002_c + cpd00008_c + 3.0 cpd00067_c

rxn34221_c: cpd00009_c + cpd00155_c <-- cpd00089_c

rxn34247_c: cpd00067_e <=> cpd00004_c + cpd00067_c

rxn34256_c: cpd00067_e <=> cpd00061_c + cpd00067_c

rxn34301_c: cpd00004_c + cpd00007_c + cpd19000_c <=> cpd00003_c + cpd12347_c

rxn34302_c: cpd00005_c + cpd00007_c + cpd19000_c <=> cpd00006_c + cpd12347_c

rxn34303_c: 2.0 cpd00001_c + cpd11594_c <=> 2.0 cpd19001_c

rxn34305_c: cpd00001_c + cpd11657_c <=> cpd00179_c

rxn34306_c: cpd03389_c + cpd11610_c <=> cpd00035_c + cpd01078_c + cpd11609_c

rxn34307_c: cpd00179_c <=> cpd00027_c + cpd11735_c

rxn34460_c: cpd00067_e <=> cpd00004_c + cpd00067_c

rxn34556_c: cpd00002_c + cpd00035_c + cpd11906_c <=> cpd00012_c + cpd00018_c + cpd00067_c + cpd11770_c

rxn34713_c: cpd00004_c + 2.0 cpd00067_c + cpd15572_c <=> cpd00003_c + cpd12458_c

rxn34724_c: cpd00001_c + cpd12458_c <=> cpd00067_c + cpd01080_c + cpd11493_c

rxn34745_c: cpd00002_c + cpd01080_c + cpd11493_c <=> cpd00012_c + cpd00018_c + cpd00067_c + cpd12458_c

rxn34959_c: cpd00080_c + cpd12458_c <=> cpd11493_c + cpd15329_c

rxn35165_c: cpd00001_c + cpd00067_c + cpd12547_c <=> cpd00908_c + cpd01080_c

rxn35213_c: cpd00002_c + cpd00033_c + cpd11913_c <=> cpd00012_c + cpd00018_c + cpd00067_c + cpd12100_c

rxn35461_c: cpd00001_c + cpd03512_c <=> 2.0 cpd00067_c + cpd00304_c

rxn35467_c: 2.0 cpd00067_c + cpd01430_c <=> cpd00001_c + cpd11750_c

rxn35491_c: cpd00002_c + cpd00054_c + cpd15573_c <=> cpd00012_c + cpd00018_c + cpd15565_c

rxn35520_c: 7.0 cpd00113_c + cpd08211_c <=> 7.0 cpd00012_c + cpd12788_c

rxn35521_c: 2.0 cpd00004_c + cpd12788_c <=> 2.0 cpd00003_c + cpd11713_c

rxn35534_c: cpd00002_c + cpd00156_c + cpd11924_c <=> cpd00012_c + cpd00018_c + cpd00067_c + cpd12133_c

rxn35562_c: cpd00001_c --> cpd19009_c

rxn35563_c: cpd00001_c --> cpd01490_c

rxn35564_c: cpd00001_c + cpd11683_c <=> 2.0 cpd02416_c

rxn35568_c: cpd23795_c --> cpd00012_c + cpd24545_c

rxn35569_c: cpd23795_c --> cpd00012_c + cpd06763_c

rxn35570_c: cpd23795_c --> cpd00012_c + cpd24548_c

rxn35573_c: cpd00113_c + cpd23795_c --> cpd00012_c + cpd00350_c

rxn35574_c: cpd00113_c + cpd00202_c <-- cpd00012_c + cpd23795_c

rxn35581_c: cpd00001_c + cpd11683_c <=> 2.0 cpd00122_c

rxn36849_c: cpd00005_c + cpd00067_c + cpd15290_c <=> cpd00006_c + cpd11665_c

rxn36931_c: cpd00005_c + cpd00067_c + cpd15290_c <=> cpd00006_c + cpd11665_c

rxn36933_c: cpd00004_c + cpd00067_c + cpd15290_c <=> cpd00003_c + cpd11665_c

rxn37647_c: cpd11628_c + cpd22235_c <=> cpd11493_c + cpd27368_c

rxn37648_c: cpd11628_c + cpd22235_c <=> cpd11493_c + cpd27368_c

rxn37649_c: cpd11628_c + cpd22235_c <=> cpd11493_c + cpd27368_c

rxn37682_c: cpd22140_c + cpd22369_c <=> cpd22139_c + cpd27839_c

rxn37683_c: cpd00080_c + cpd11628_c <=> cpd11493_c + cpd22235_c

rxn37708_c: cpd00005_c + cpd00007_c + cpd00067_c + cpd27317_c <=> cpd00001_c + cpd00006_c + cpd21877_c

rxn37723_c: cpd00001_c + cpd28485_c <=> cpd00067_c + cpd02077_c + cpd11493_c

rxn37726_c: cpd00001_c + cpd26916_c <=> cpd11493_c + cpd16301_c

rxn37822_c: cpd00007_c + cpd24400_c + cpd28070_c <=> cpd00001_c + cpd24389_c + cpd27744_c

rxn37832_c: cpd28898_c <=> cpd24922_c

rxn37833_c: cpd28899_c <=> cpd24932_c

rxn37834_c: cpd05296_c --> cpd25331_c

rxn37835_c: cpd28905_c <=> cpd24933_c

rxn37836_c: cpd28907_c <=> cpd25330_c

rxn37837_c: cpd28909_c <=> cpd24935_c

rxn37838_c: cpd25328_c --> cpd05304_c

rxn37839_c: cpd05305_c --> cpd25326_c

rxn37841_c: cpd24918_c <=> cpd28902_c

rxn37842_c: cpd15240_c <=> cpd29018_c

rxn37843_c: cpd24919_c <=> cpd29019_c

rxn37845_c: cpd05196_c --> cpd20876_c

rxn37859_c: cpd00080_c + cpd29077_c <=> cpd00046_c + cpd00067_c + cpd29080_c

rxn37864_c: cpd11628_c + cpd22235_c <=> cpd11493_c + cpd27368_c

rxn37905_c: cpd28293_c <-- cpd00014_c

rxn37908_c: cpd00017_c + cpd11763_c <=> cpd00019_c + cpd26897_c

rxn37913_c: cpd11492_c + cpd11628_c <=> cpd00011_c + cpd11493_c + cpd11726_c

rxn37925_c: cpd00043_c + cpd22406_c <=> cpd00014_c + cpd00067_c + cpd22256_c

rxn37926_c: cpd00043_c + cpd22406_c <=> cpd00014_c + cpd00067_c + cpd22256_c

rxn37928_c: cpd27086_c + cpd28293_c <=> cpd00014_c + cpd27082_c

rxn37942_c: cpd00002_c + cpd27149_c <=> cpd00008_c + cpd27148_c

rxn37951_c: 2.0 cpd00017_e + cpd00084_e + cpd01311_e + cpd28207_e <=> cpd00035_c + 2.0 cpd00060_c + cpd00104_c + 2.0 cpd03091_c + cpd28307_c

rxn37959_c: cpd00006_c + cpd11836_c <=> cpd00005_c + cpd00067_c + cpd11726_c

rxn37962_c: cpd00006_c + cpd11836_c <=> cpd00005_c + cpd00067_c + cpd11726_c

rxn37966_c: cpd11492_c + cpd28167_c <=> cpd00011_c + cpd11493_c + cpd11726_c

rxn37967_c: <=> cpd27686_c

rxn37969_c: cpd00001_c + cpd27148_c <=> cpd00009_c + cpd27149_c

rxn37973_c: cpd00001_c --> cpd00190_c

rxn37974_c: cpd00001_c + cpd27261_c <=> cpd11691_c

rxn37975_c: cpd00001_c + cpd27577_c <=> cpd27613_c + cpd28035_c

rxn37976_c: --> cpd30321_c

rxn37977_c: cpd00001_c <=> cpd22369_c

rxn37979_c: cpd27149_c <=> 2.0 cpd27839_c

rxn37996_c: <=> cpd22911_c

rxn38001_c: cpd00002_c + cpd00053_c + cpd27376_c <=> cpd00008_c + cpd00009_c + cpd00023_c + cpd26693_c

rxn38014_c: cpd00007_c + cpd22234_c <=> cpd00025_c + cpd28232_c

rxn38040_c: cpd00017_c + cpd22346_c <=> cpd00019_c + cpd12107_c

rxn38041_c: cpd00017_c + cpd22346_c <=> cpd00019_c + cpd12107_c

rxn38061_c: cpd00315_c + cpd22518_c <=> cpd00031_c + cpd21756_c

rxn38062_c: cpd28293_c <-- cpd00014_c

rxn38071_c: cpd26891_c <=> cpd26888_c

rxn38089_c: cpd27687_c <=> cpd00012_c

rxn38093_c: cpd00006_c + cpd28167_c <=> cpd00005_c + cpd11728_c

rxn38100_c: 2.0 cpd00005_c + cpd00022_c + 2.0 cpd00067_c + cpd00070_c <=> 2.0 cpd00006_c + 2.0 cpd00010_c + cpd00011_c + cpd27424_c

rxn38109_c: cpd22140_c + cpd22369_c <=> cpd22139_c + cpd27839_c

rxn38110_c: cpd22140_c + cpd22369_c <=> cpd22139_c + cpd27839_c

rxn38111_c: cpd22140_c + cpd22369_c <=> cpd22139_c + cpd27839_c

rxn38112_c: cpd22140_c + cpd22369_c <=> cpd22139_c + cpd27839_c

rxn38161_c: cpd00022_c + cpd28168_c <=> cpd00010_c + cpd22026_c

rxn38166_c: cpd00006_c + cpd00010_c + cpd27421_c <=> cpd00005_c + cpd00067_c + cpd27059_c

rxn38167_c: cpd00006_c + cpd00010_c + cpd27421_c <=> cpd00005_c + cpd00067_c + cpd27059_c

rxn38168_c: cpd00006_c + cpd00010_c + cpd27421_c <=> cpd00005_c + cpd00067_c + cpd27059_c

rxn38172_c: cpd00038_c + cpd25368_c <=> cpd00012_c + cpd00067_c + cpd25369_c

rxn38175_c: cpd00003_c <-- cpd00067_c + cpd00133_c

rxn38245_c: cpd00001_c + cpd27835_c <=> cpd00116_c + cpd27777_c

rxn38246_c: cpd00001_c + cpd27835_c <=> cpd00116_c + cpd27777_c

rxn38247_c: cpd00001_c + cpd27835_c <=> cpd00116_c + cpd27777_c

rxn38250_c: cpd00025_c + cpd27851_c <=> 2.0 cpd00001_c + 2.0 cpd27852_c

rxn38251_c: cpd00025_c + cpd27851_c <=> 2.0 cpd00001_c + 2.0 cpd27852_c

rxn38252_c: cpd00025_c + cpd27851_c <=> 2.0 cpd00001_c + 2.0 cpd27852_c

rxn38266_c: cpd00002_c <-- cpd00012_c

rxn38271_c: cpd00002_c + cpd27149_c <=> cpd00008_c + cpd27148_c

rxn38287_c: cpd28326_c <=> cpd00204_c + cpd22334_c

rxn38288_c: cpd00080_c + cpd11628_c <=> cpd11493_c + cpd22235_c

rxn38339_c: cpd00005_c + cpd00067_c + cpd27021_c <=> cpd00006_c + cpd21088_c

rxn38346_c: cpd00001_c + cpd26950_c <=> cpd26952_c

rxn38347_c: cpd00001_c + cpd26723_c <=> cpd26722_c

rxn38361_c: cpd00001_c --> cpd00665_c

rxn38368_c: cpd00001_c <=> cpd21755_c

rxn38369_c: cpd00001_c <=> cpd21755_c

rxn38440_c: cpd00001_c + cpd11628_c <=> cpd11493_c + cpd27058_c

rxn38468_c: cpd26551_c <-- cpd28281_c

rxn38469_c: cpd00001_c + cpd25936_c <-- cpd00190_c

rxn38470_c: cpd00009_c + cpd25936_c <-- cpd00089_c

rxn38471_c: cpd00163_c <-- cpd00014_c

rxn38511_c: cpd00001_c --> cpd30321_c

rxn38532_c: cpd28166_c + cpd28167_c <=> cpd11493_c + cpd21848_c

rxn38608_c: cpd28607_c <=> cpd28603_c

rxn38609_c: cpd28607_c <=> cpd28603_c

rxn38629_c: cpd11492_c + cpd28832_c <=> cpd00010_c + cpd00011_c + cpd22073_c

rxn38690_c: cpd00053_c + cpd00095_c + cpd00171_c <-- cpd00016_c + cpd00023_c

rxn38695_c: cpd00002_c + cpd00003_c <-- cpd00006_c

rxn38698_c: cpd00042_c + cpd09283_c --> cpd00111_c + cpd00620_c

rxn38700_c: cpd00038_c + cpd00305_c --> cpd00031_c + cpd00056_c

rxn38703_c: cpd00003_c + cpd00033_c + cpd00084_c <-- cpd00133_c + cpd26630_c

rxn38717_c: 2.0 cpd00076_c <=> cpd00190_c + cpd27312_c

rxn38813_c: cpd00001_c + cpd28278_c <=> cpd26675_c + cpd26958_c

rxn38814_c: cpd00017_c + cpd28567_c <=> cpd00019_c + cpd12484_c

rxn38874_c: cpd00282_c + cpd15290_c <=> cpd00247_c + cpd11665_c

rxn38877_c: cpd00190_c + cpd15290_c <=> cpd00170_c + cpd11665_c

rxn38897_c: cpd00159_c + cpd15290_c <=> cpd00020_c + cpd11665_c

rxn38901_c: cpd00004_c + cpd00067_c + cpd15290_c <=> cpd00003_c + cpd11665_c

rxn38986_c: 2.0 cpd00067_c + cpd15290_c <=> cpd11665_c

rxn39021_c: cpd00002_c <=> cpd00012_c + cpd28600_c

rxn39044_c: cpd00001_c + cpd15398_c <=> cpd15387_c + cpd15396_c

rxn39045_c: cpd00001_c + cpd15398_c <=> cpd00117_c + cpd15397_c

rxn39060_c: cpd00005_c + cpd00067_c + cpd14940_c <=> cpd00006_c + cpd12458_c

rxn39066_c: cpd00858_c <-- cpd02431_c

rxn39068_c: cpd00002_c + cpd00023_c <=> cpd00012_c + cpd00018_c + cpd12227_c

rxn39070_c: cpd00001_c + cpd11791_c <=> cpd00027_c

rxn39074_c: cpd12407_c <=> cpd11619_c + cpd11685_c

rxn39075_c: cpd00001_c + 0.01 cpd16880_c <=> cpd00067_c + 0.02 cpd00214_c + 0.03 cpd01080_c + 0.015 cpd03847_c + 0.01 cpd14514_c + 0.655 cpd15237_c + 0.01 cpd15240_c + 0.27 cpd15269_c

rxn39082_c: cpd00001_c + 0.01 cpd16883_c <=> cpd00067_c + 0.02 cpd00214_c + 0.03 cpd01080_c + 0.01 cpd02755_c + 0.015 cpd03847_c + 0.655 cpd15237_c + 0.01 cpd15240_c + 0.27 cpd15269_c

rxn39087_c: cpd00002_c + 0.01 cpd11715_c <=> cpd00008_c + cpd00067_c + 0.01 cpd15288_c

rxn39093_c: cpd23795_c --> cpd00012_c + cpd00407_c

rxn39095_c: cpd00113_c + cpd23795_c --> cpd00012_c + cpd00350_c

rxn39098_c: cpd23795_c <=> cpd00012_c + cpd03749_c

rxn39099_c: cpd23795_c <=> cpd00012_c + cpd03748_c

rxn39100_c: cpd23795_c --> cpd00012_c + cpd03620_c

rxn39101_c: cpd23795_c --> cpd00012_c + cpd03619_c

rxn39103_c: cpd00822_c + cpd30740_c <=> cpd00036_c + cpd00135_c

rxn39104_c: cpd00001_c + cpd23795_c --> cpd00012_c + cpd01062_c

rxn39277_c: cpd00085_c <=> cpd01655_c

rxn39292_c: cpd00100_c + cpd19000_c <=> cpd11677_c

rxn39324_c: cpd11657_c <=> cpd00179_c + cpd11976_c

rxn39341_c: cpd00048_c + cpd00381_c <=> cpd00084_c + cpd01608_c

rxn39358_c: cpd11707_c + cpd19004_c <=> cpd00010_c + cpd11677_c

rxn39366_c: cpd30743_c <-- cpd00547_c

rxn39367_c: cpd00001_c + cpd11737_c <=> cpd00992_c

rxn39369_c: cpd00550_c + cpd19021_c --> cpd01190_c + cpd19016_c

rxn39375_c: cpd11652_c + cpd11770_c <=> cpd02676_c + cpd11906_c

rxn39382_c: cpd11609_c + cpd19174_c <=> cpd02125_c + cpd11610_c

rxn39419_c: cpd01297_c + cpd11707_c <=> cpd00010_c + cpd19004_c

rxn39422_c: cpd01326_c + cpd11652_c <=> cpd11917_c + cpd12556_c

rxn39425_c: cpd11463_c + cpd12003_c <=> cpd11916_c + cpd12104_c

rxn39427_c: cpd01431_c + cpd11707_c <=> cpd00010_c + cpd19004_c

rxn39429_c: cpd11463_c + cpd12036_c <=> cpd11907_c + cpd19194_c

rxn39431_c: cpd01468_c <-- cpd19044_c

rxn39454_c: cpd00001_c + cpd06227_c <=> cpd00023_c + cpd00087_c

rxn39455_c: cpd00001_c + cpd02311_c --> cpd00013_c + cpd00047_c + cpd03303_c

rxn39471_c: cpd02862_c + cpd11770_c <=> cpd02977_c + cpd11906_c

rxn39473_c: cpd00048_c + cpd12732_c <=> cpd00042_c + cpd01608_c

rxn39482_c: cpd03614_c <=> cpd03615_c

rxn39483_c: cpd00001_c + cpd12836_c <=> cpd02767_c + cpd13381_c

rxn39486_c: cpd00001_c + cpd03836_c + cpd11609_c <=> cpd03779_c + cpd11610_c

rxn39489_c: 2.0 cpd00005_c + cpd00022_c + 2.0 cpd00067_c + cpd00070_c <=> cpd00001_c + 2.0 cpd00006_c + 2.0 cpd00010_c + cpd00011_c + cpd00487_c

rxn39491_c: 2.0 cpd00005_c + cpd00022_c + 4.0 cpd00067_c + cpd00070_c <=> cpd00001_c + 2.0 cpd00006_c + cpd00010_c + cpd00011_c + cpd12196_c

rxn39496_c: cpd00026_c + cpd02214_c <=> cpd00014_c + cpd12897_c

rxn39613_c: cpd00001_c + cpd00007_c + cpd03811_c <=> cpd00013_c + cpd00025_c + cpd11616_c

rxn39687_c: cpd21384_c --> cpd08697_c

rxn39691_c: cpd00002_c + cpd00010_c + cpd08721_c --> cpd00001_c + cpd00012_c + cpd00018_c + cpd08726_c

rxn39692_c: cpd11610_c + cpd19107_c <=> cpd08266_c + cpd11609_c

rxn39711_c: cpd00007_c + cpd11610_c + cpd19099_c <=> cpd00001_c + cpd08735_c + cpd11609_c

rxn39728_c: cpd00002_c + cpd00010_c + cpd08259_c --> cpd00001_c + cpd00012_c + cpd00018_c + cpd08261_c

rxn39739_c: cpd00022_c + 7.0 cpd00070_c + cpd11493_c <=> 7.0 cpd00010_c + 7.0 cpd00011_c + cpd13411_c

rxn39740_c: 7.0 cpd00070_c + cpd00120_c + cpd11493_c <=> 7.0 cpd00010_c + 7.0 cpd00011_c + cpd13413_c

rxn39741_c: 8.0 cpd00070_c + cpd04029_c + cpd11493_c <=> 9.0 cpd00010_c + 8.0 cpd00011_c + cpd13430_c

rxn39743_c: 10.0 cpd00070_c + cpd11493_c <=> 10.0 cpd00010_c + 10.0 cpd00011_c + cpd13417_c

rxn39744_c: 9.0 cpd00070_c + cpd00086_c + cpd11493_c <=> 9.0 cpd00010_c + 9.0 cpd00011_c + cpd13418_c

rxn39745_c: 9.0 cpd00070_c + cpd00120_c + cpd11493_c <=> 9.0 cpd00010_c + 9.0 cpd00011_c + cpd13419_c

rxn39746_c: cpd19943_c --> cpd09148_c

rxn39766_c: cpd00066_c --> cpd01035_c

rxn39767_c: cpd09223_c + 2.0 cpd13440_c <=> cpd03029_c

rxn39768_c: cpd09216_c + cpd13440_c <=> cpd08823_c

rxn39769_c: cpd09209_c + cpd09213_c <-- cpd09214_c

rxn39772_c: cpd19069_c --> cpd00489_c

rxn39773_c: cpd19069_c --> cpd03320_c

rxn39777_c: 2.0 cpd09476_c --> cpd09477_c

rxn39778_c: cpd09482_c --> cpd09484_c

rxn39779_c: cpd09485_c --> cpd00136_c + cpd19150_c

rxn39793_c: cpd11609_c + cpd12118_c <=> cpd11610_c + cpd11625_c

rxn39799_c: cpd00070_c + cpd19166_c <=> cpd00010_c + cpd14483_c

rxn39814_c: cpd00001_c + cpd00952_c + cpd11609_c <=> cpd11610_c + cpd14710_c

rxn39818_c: cpd00007_c + cpd05497_c + cpd11610_c <=> cpd00001_c + cpd05508_c + cpd11609_c

rxn39848_c: 3.0 cpd00007_c + 3.0 cpd11610_c + cpd14866_c <=> 4.0 cpd00001_c + 3.0 cpd11609_c + cpd14867_c

rxn39852_c: cpd14950_c <-- cpd10013_c

rxn39893_c: cpd04417_c <-- cpd19184_c

rxn39894_c: cpd19184_c --> cpd16461_c

rxn39899_c: cpd19111_c <=> cpd04765_c

rxn39910_c: cpd00239_c + cpd17286_c <=> cpd00009_c + cpd12255_c

rxn39913_c: cpd08729_c <-- cpd19363_c

rxn39914_c: cpd19363_c <-- cpd08716_c

rxn39920_c: cpd11609_c + 2.0 cpd17626_c <=> cpd01462_c + cpd11610_c

rxn39928_c: cpd00001_c + cpd00002_c + cpd01449_c + cpd11610_c <=> cpd00008_c + cpd00009_c + cpd11609_c + cpd19422_c

rxn39931_c: cpd00007_c + cpd11610_c + cpd19514_c <=> cpd00001_c + cpd00071_c + cpd00099_c + cpd11609_c

rxn39955_c: cpd00017_c + cpd11492_c <=> cpd00019_c + cpd20921_c

rxn39960_c: cpd11657_c <=> cpd00794_c

rxn39996_c: cpd00007_c + cpd01420_c + cpd11610_c <=> cpd00001_c + cpd11609_c + cpd24336_c

rxn39997_c: cpd11609_c + cpd24337_c <=> 2.0 cpd00304_c + cpd11610_c

rxn40006_c: 2.0 cpd00005_c + 2.0 cpd00067_c + cpd12196_c <=> 2.0 cpd00006_c + cpd00010_c + cpd11650_c

rxn40014_c: cpd11492_c + cpd20921_c <=> cpd00011_c + cpd11493_c + cpd31062_c

rxn40070_c: cpd00007_c + cpd11610_c + cpd31099_c <=> cpd00001_c + cpd11609_c + cpd31100_c

rxn40101_c: cpd00017_c + cpd00069_c + cpd11610_c <=> cpd00060_c + cpd01042_c + cpd03091_c + cpd11609_c + cpd14545_c

rxn40156_c: cpd00001_c + cpd00448_c + cpd11609_c <=> cpd00223_c + cpd11610_c

rxn40162_c: cpd11609_c + cpd31165_c <=> cpd00581_c + cpd11610_c

rxn40163_c: cpd11609_c + cpd31161_c <=> cpd02824_c + cpd11610_c

rxn40165_c: cpd12003_c + cpd12335_c <=> cpd11916_c + cpd11919_c + cpd31162_c

rxn40166_c: 2.0 cpd12194_c <=> 2.0 cpd11751_c + cpd31159_c

rxn40167_c: 2.0 cpd12003_c <=> 2.0 cpd11916_c + cpd31157_c

rxn40191_c: cpd31206_c --> cpd31205_c

rxn40201_c: cpd00001_c + cpd00007_c + cpd03610_c <=> cpd00025_c + cpd00075_c + cpd11616_c

rxn40202_c: cpd00001_c + cpd00007_c + cpd03610_c <=> cpd00025_c + cpd00075_c + cpd11733_c

rxn40203_c: cpd11640_c + cpd20940_c <=> cpd00239_c

rxn40206_c: cpd15829_c <-- cpd15827_c

rxn40212_c: cpd31177_c --> cpd31205_c

rxn40213_c: cpd31205_c <=> cpd31169_c

rxn40216_c: cpd01608_c + cpd13049_c <=> cpd00081_c + cpd00084_c + cpd13050_c

rxn40242_c: 2.0 cpd00007_c + cpd00024_c + cpd11610_c + cpd31241_c <=> cpd00001_c + cpd00011_c + cpd00036_c + cpd11609_c + cpd21284_c

rxn40261_c: 4.0 cpd00007_c + cpd00028_c + 4.0 cpd11610_c <=> 4.0 cpd00001_c + cpd00204_c + cpd10515_c + 4.0 cpd11609_c + cpd31266_c

rxn40271_c: cpd02449_c --> cpd16551_c

rxn40288_c: cpd11609_c + cpd19047_c <=> cpd00011_c + cpd00551_c + cpd11610_c

rxn40290_c: 4.0 cpd00007_c + cpd00028_c + 4.0 cpd11610_c <=> 4.0 cpd00001_c + cpd00204_c + cpd10515_c + 4.0 cpd11609_c + cpd31267_c

rxn40292_c: cpd00006_c + cpd00239_c <=> cpd00005_c + cpd00067_c + cpd20940_c

rxn40326_c: cpd31212_c <=> cpd31215_c

rxn40327_c: cpd31212_c <-- cpd31220_c

rxn40328_c: cpd31175_c <=> cpd31169_c

rxn40329_c: cpd31169_c <=> cpd31178_c

rxn40333_c: cpd00795_c + cpd11609_c <=> cpd00024_c + cpd11610_c

rxn40428_c: cpd00016_c + cpd00119_c <-- cpd02775_c

rxn40445_c: cpd12370_c + cpd15364_c <=> cpd00012_c + cpd11493_c

rxn40450_c: cpd12489_c <=> cpd31353_c

rxn40470_c: cpd31376_c --> cpd31377_c

rxn40475_c: cpd17502_c --> cpd04066_c

rxn40479_c: cpd00620_c + cpd31378_c <-- cpd31370_c

rxn40486_c: cpd31379_c --> cpd17503_c

rxn40496_c: 28.0 cpd00005_c + 47.0 cpd00067_c + 15.0 cpd00070_c + cpd23741_c --> 16.0 cpd00001_c + 28.0 cpd00006_c + 20.0 cpd00010_c + 15.0 cpd00011_c + cpd33169_c

rxn40520_c: cpd11493_c + cpd33585_c <=> cpd00018_c + cpd00067_c + cpd22441_c

rxn40523_c: cpd00584_c + cpd31664_c <=> cpd00046_c + cpd00067_c + cpd36278_c

rxn40546_c: cpd00112_c + cpd22447_c <=> cpd00046_c + cpd00067_c + cpd02971_c

rxn40563_c: cpd11493_c + cpd23224_c <=> cpd00018_c + cpd00067_c

rxn40584_c: cpd36928_c <=> cpd00001_c + cpd11493_c + cpd32896_c

rxn40586_c: cpd00001_c <=> cpd26940_c

rxn40598_c: cpd02246_c + cpd27514_c <=> cpd00067_c + cpd27539_c

rxn40601_c: cpd27173_c <=> cpd00154_c + cpd03647_c + cpd27109_c

rxn40608_c: cpd00002_c + cpd00119_c + cpd27218_c <=> cpd00012_c + cpd00018_c

rxn40609_c: cpd00293_c + cpd12396_c <=> cpd11799_c + cpd37247_c

rxn40612_c: cpd00098_c + cpd22517_c <=> cpd00046_c + cpd00067_c

rxn40614_c: cpd00042_c + cpd33845_c <-- cpd33560_c

rxn40626_c: cpd22495_c <-- cpd02229_c

rxn40634_c: cpd00002_c + cpd00033_c + cpd11493_c <=> cpd00012_c + cpd00018_c

rxn40648_c: cpd00057_c + cpd00256_c <=> cpd00046_c + cpd00067_c

rxn40702_c: cpd00001_c --> cpd34090_c

rxn40703_c: cpd00002_c + cpd00132_c + cpd22284_c <=> cpd00012_c + cpd00018_c

rxn40707_c: cpd00007_c + 2.0 cpd00067_c + cpd12458_c + 2.0 cpd28082_c <=> 2.0 cpd00001_c + cpd27716_c + 2.0 cpd27757_c

rxn40712_c: cpd00022_c + cpd37257_c <=> cpd00010_c + cpd00067_c

rxn40718_c: cpd00022_c + cpd11493_c <=> cpd00010_c

rxn40740_c: cpd32130_c + 2.0 cpd36154_c <=> 2.0 cpd11493_c + cpd35315_c

rxn40778_c: cpd00001_c + cpd27994_c <=> 2.0 cpd23936_c

rxn40779_c: cpd00007_c + cpd00639_c + cpd21035_c <=> cpd00001_c + cpd11630_c

rxn40782_c: cpd00010_c + cpd11799_c <=> cpd00045_c + cpd00067_c + cpd11493_c

rxn40792_c: cpd33866_c <=> 2.0 cpd00067_c + 2.0 cpd02229_c + cpd37168_c

rxn40794_c: cpd27171_c + cpd27279_c <=> cpd25737_c

rxn40795_c: cpd03587_c + cpd14878_c <-- cpd19245_c

rxn40816_c: cpd00020_c + cpd00038_c <-- cpd00031_c + cpd00067_c

rxn40825_c: cpd00001_c + cpd00002_c + cpd22191_c <=> cpd00009_c + cpd00018_c

rxn40831_c: cpd00006_c + cpd01159_c --> cpd00005_c + 2.0 cpd00067_c

rxn40845_c: cpd33968_c <=> cpd32508_c

rxn40849_c: cpd00010_c + cpd12458_c <=> cpd00327_c + cpd11493_c

rxn40861_c: cpd00080_c + cpd22306_c <=> cpd00009_c

rxn40868_c: 2.0 cpd00005_c + 2.0 cpd00007_c + 2.0 cpd00017_c + cpd00067_c + cpd00906_c + cpd25439_c --> 2.0 cpd00001_c + 2.0 cpd00006_c + cpd00011_c + cpd00012_c + 2.0 cpd00019_c + cpd21868_c

rxn40870_c: cpd00002_c + cpd00084_c + cpd27834_c <=> cpd00012_c + cpd00018_c

rxn40880_c: cpd00002_c + cpd00023_c + cpd11493_c <=> cpd00012_c + cpd00018_c + cpd36986_c

rxn40885_c: 2.0 cpd00042_c + cpd21980_c <=> cpd00067_c + cpd00099_c + cpd04005_c

rxn40893_c: cpd00059_c + cpd00067_c + cpd05486_c <-- cpd00001_c + cpd03637_c

rxn40896_c: cpd03586_c + cpd03831_c <=> cpd00008_c + cpd00067_c

rxn40899_c: cpd00002_c + cpd11799_c <=> cpd00008_c + cpd00067_c + cpd12396_c

rxn40901_c: cpd03335_c + cpd28218_c <=> cpd01020_c

rxn40911_c: cpd00009_c --> cpd00485_c

rxn40913_c: 11.0 cpd00005_c + 22.0 cpd00067_c + 9.0 cpd00070_c --> 5.0 cpd00001_c + 11.0 cpd00006_c + 11.0 cpd00010_c + 11.0 cpd00011_c + cpd32300_c

rxn40916_c: cpd00006_c + cpd22234_c <=> cpd00005_c + cpd00067_c + cpd26725_c

rxn40919_c: cpd00006_c + cpd00007_c + cpd23235_c <=> cpd00001_c + cpd00005_c + cpd00067_c + cpd11359_c

rxn40929_c: 4.0 cpd00002_c + 4.0 cpd00017_c + 2.0 cpd00084_c + 2.0 cpd00156_c + 2.0 cpd36199_c <=> 4.0 cpd00012_c + 4.0 cpd00018_c + 8.0 cpd00067_c + 2.0 cpd11493_c + cpd34565_c

rxn40930_c: cpd00001_c + cpd21994_c <=> cpd00067_c + cpd22016_c + cpd27058_c

rxn40941_c: cpd00002_c + cpd00023_c + cpd00312_c --> cpd00008_c + cpd00009_c + cpd00067_c

rxn40949_c: cpd26689_c + cpd27149_c <=> cpd22281_c + cpd36088_c

rxn40954_c: cpd00017_c + cpd00121_c <-- cpd00019_c + cpd00067_c

rxn40962_c: 2.0 cpd00008_c + 2.0 cpd00009_c + cpd01449_c + 2.0 cpd27757_c <=> 2.0 cpd00001_c + 2.0 cpd00002_c + 2.0 cpd28082_c

rxn40986_c: cpd00002_c + cpd00067_c + cpd14548_c + cpd21836_c + 2.0 cpd28082_c <=> cpd00012_c + cpd00018_c + 2.0 cpd27757_c + cpd28205_c + cpd36957_c

rxn40987_c: cpd00001_c + cpd36287_c <=> cpd00067_c + cpd11493_c + cpd32533_c

rxn40988_c: cpd00001_c + cpd36864_c + cpd36873_c <=> cpd00067_c + 2.0 cpd11493_c + cpd31629_c

rxn40991_c: cpd07568_c <-- cpd31540_c

rxn41003_c: cpd00002_c + cpd00035_c + cpd11493_c <=> cpd00012_c + cpd00018_c + cpd36668_c

rxn41005_c: cpd00387_c + cpd21754_c <=> cpd00008_c + cpd36779_c

rxn41010_c: cpd00002_c + cpd00023_c + cpd00118_c --> cpd00008_c + cpd00009_c + cpd00067_c

rxn41016_c: cpd32777_c --> cpd34106_c

rxn41040_c: cpd00067_c + cpd11492_c + cpd11628_c <=> cpd00011_c + cpd11493_c

rxn41051_c: cpd16528_c --> cpd00027_c + cpd01304_c

rxn41056_c: cpd00006_c + cpd00383_c <-- cpd00005_c + cpd00067_c

rxn41057_c: cpd36406_c + cpd36979_c <=> cpd00067_c + cpd11493_c

rxn41061_c: cpd00033_c + cpd00502_c <=> cpd01762_c + cpd28218_c

rxn41072_c: cpd11493_c + cpd32461_c <=> cpd00018_c + cpd00067_c + cpd36887_c

rxn41089_c: cpd33965_c --> cpd32360_c

rxn41099_c: cpd02246_c + cpd27448_c <=> cpd00067_c + cpd27485_c

rxn41111_c: cpd00022_c + cpd22447_c <=> cpd00010_c

rxn41112_c: cpd00001_c + 2.0 cpd22234_c <=> 2.0 cpd00010_c + cpd00067_c + cpd36939_c

rxn41116_c: cpd00070_c + cpd29330_c <=> cpd00010_c + cpd00011_c + cpd28320_c

rxn41145_c: cpd02108_c <=> cpd06003_c

rxn41172_c: 2.0 cpd00042_c + cpd27930_c <=> cpd27931_c

rxn41180_c: cpd00038_c + cpd00296_c <-- cpd00031_c

rxn41183_c: cpd00002_c + cpd00067_c + cpd28253_c <=> cpd00012_c

rxn41196_c: cpd00002_c + cpd00129_c + cpd27808_c <=> cpd00012_c + cpd00018_c

rxn41200_c: cpd00001_c + cpd27669_c <=> cpd01074_c + cpd24014_c

rxn41206_c: cpd11628_c + cpd15329_c <=> cpd11493_c + cpd36728_c

rxn41218_c: cpd01262_c <-- cpd00027_c

rxn41230_c: cpd00709_c <--

rxn41241_c: cpd00007_c + cpd00059_c + cpd00067_c + cpd04947_c <-- cpd00001_c + cpd19191_c

rxn41260_c: cpd00001_c + 8.0 cpd00067_c + 9.0 cpd00070_c + cpd36137_c <=> 9.0 cpd00010_c + 9.0 cpd00011_c + cpd11493_c + cpd31580_c

rxn41280_c: cpd00022_c + cpd36486_c <=> cpd00010_c + cpd00067_c

rxn41294_c: cpd22271_c + cpd28218_c <=> cpd00013_c + cpd26863_c

rxn41296_c: cpd00026_c + cpd36665_c <=> cpd00014_c + cpd36076_c

rxn41300_c: cpd00001_c + cpd00067_c + cpd28004_c <=> cpd00105_c + cpd28002_c

rxn41318_c: cpd00007_c + 2.0 cpd00067_c + 2.0 cpd27031_c + cpd27876_c <=> cpd00001_c + cpd22362_c + 2.0 cpd27029_c

rxn41329_c: cpd01449_c + 2.0 cpd27757_c <=> 2.0 cpd00067_c + 2.0 cpd28082_c

rxn41336_c: cpd00001_c + cpd27897_c <=> 2.0 cpd01384_c

rxn41337_c: cpd00001_c + cpd37060_c <=> cpd00067_c + cpd05274_c + cpd11799_c

rxn41344_c: cpd11493_c + cpd36972_c <=> cpd00018_c + cpd00067_c

rxn41350_c: cpd11493_c + cpd33594_c <=> cpd00018_c + cpd00067_c

rxn41352_c: cpd11493_c + cpd33085_c <=> cpd00018_c + cpd00067_c + cpd37055_c

rxn41366_c: 22.0 cpd00005_c + 32.0 cpd00067_c + 5.0 cpd00070_c + cpd11611_c <=> 11.0 cpd00001_c + 22.0 cpd00006_c + 10.0 cpd00010_c + 10.0 cpd00011_c + cpd37255_c

rxn41374_c: cpd00002_c + cpd00084_c + cpd27833_c <=> cpd00012_c + cpd00018_c

rxn41379_c: 2.0 cpd00042_c + cpd01018_c <-- 2.0 cpd00084_c

rxn41380_c: cpd00007_c + cpd00458_c + cpd21035_c <=> cpd00001_c + cpd11630_c

rxn41381_c: cpd01233_c + cpd02879_c --> cpd00067_c + cpd00096_c

rxn41400_c: cpd00001_c <=> cpd27687_c

rxn41408_c: cpd00001_c + cpd00002_c + cpd27149_c <=> cpd00008_c + cpd00009_c + 2.0 cpd36163_c

rxn41412_c: cpd11628_c + cpd36668_c + cpd37059_c <=> 2.0 cpd00067_c + 2.0 cpd11493_c + cpd36274_c

rxn41422_c: 19.0 cpd00005_c + cpd00022_c + 37.0 cpd00067_c + 15.0 cpd00070_c --> 8.0 cpd00001_c + 19.0 cpd00006_c + 19.0 cpd00010_c + 18.0 cpd00011_c + cpd34050_c

rxn41429_c: cpd22251_c <=> cpd31973_c

rxn41430_c: cpd27340_c + cpd36269_c <=> cpd26855_c

rxn41433_c: cpd27275_c <--

rxn41448_c: cpd00026_c + cpd27124_c <=> cpd00014_c + cpd00067_c

rxn41452_c: cpd00017_c + cpd11492_c <=> cpd00019_c + cpd36635_c

rxn41477_c: cpd35544_c + cpd37082_c <=> cpd00067_c + cpd19245_c

rxn41481_c: cpd00001_c + cpd00002_c + cpd00020_c <-- cpd00009_c + cpd00018_c + 2.0 cpd00067_c

rxn41490_c: cpd36404_c + cpd36906_c <=> cpd36418_c + cpd36439_c

rxn41502_c: cpd00042_c + cpd34345_c <-- cpd00067_c + cpd04374_c

rxn41509_c: cpd00001_c + cpd36052_c <=> cpd00067_c + cpd11493_c + cpd33876_c

rxn41549_c: cpd00100_c + cpd27339_c <=> cpd00162_c

rxn41572_c: cpd00002_c + cpd00059_c <-- cpd00008_c + cpd00067_c

rxn41574_c: cpd00001_c + cpd26711_c <=> 2.0 cpd01157_c

rxn41586_c: cpd00007_c + cpd00420_c + cpd21035_c <=> cpd00001_c + cpd11630_c

rxn41591_c: cpd00387_c + cpd27124_c <=> cpd00008_c + cpd00067_c

rxn41604_c: cpd26723_c <=> cpd00048_c + cpd00164_c + cpd03019_c

rxn41635_c: cpd12458_c + cpd15329_c <=> cpd11493_c + cpd26148_c

rxn41636_c: cpd00059_c + 2.0 cpd26784_c <=> cpd00067_c + 2.0 cpd26785_c

rxn41637_c: cpd00001_c + cpd28281_c <-- cpd00027_c + cpd25936_c

rxn41640_c: cpd00026_c + cpd04125_c <-- cpd00014_c + cpd00067_c

rxn41643_c: 2.0 cpd00042_c + cpd22225_c <=> cpd00001_c + cpd00057_c

rxn41655_c: cpd00022_c + cpd36120_c <=> cpd00010_c + cpd00067_c

rxn41672_c: cpd00324_c + cpd02246_c <-- cpd00067_c + cpd00239_c

rxn41697_c: cpd00002_c + cpd02302_c + cpd11493_c <=> cpd00012_c + cpd00018_c + cpd36713_c

rxn41701_c: cpd00017_c + cpd14613_c <=> cpd00019_c + cpd00067_c

rxn41716_c: 7.0 cpd00005_c + 15.0 cpd00067_c + 2.0 cpd00070_c + cpd18009_c --> 3.0 cpd00001_c + 7.0 cpd00006_c + 8.0 cpd00010_c + 8.0 cpd00011_c + cpd08797_c

rxn41728_c: cpd00002_c + cpd02674_c + cpd11493_c <=> cpd00012_c + cpd00018_c + cpd36476_c

rxn41730_c: cpd00006_c + 2.0 cpd00042_c <-- cpd00005_c + cpd00067_c

rxn41732_c: cpd27209_c <=> cpd34084_c

rxn41757_c: cpd00144_c + cpd37138_c <=> cpd00014_c + cpd00067_c

rxn41784_c: cpd11493_c + cpd23224_c <=> cpd00018_c + cpd00067_c + cpd36295_c

rxn41793_c: cpd00001_c + cpd27668_c <=> cpd00108_c + cpd23867_c + cpd23868_c

rxn41794_c: cpd22084_c + cpd22968_c <=> cpd00067_c + cpd11493_c + cpd22969_c

rxn41800_c: cpd01185_c <-- cpd00001_c

rxn41807_c: 5.0 cpd00005_c + 2.0 cpd00017_c + cpd00022_c + 8.0 cpd00067_c + 5.0 cpd00070_c + cpd11493_c <=> 4.0 cpd00001_c + 5.0 cpd00006_c + 6.0 cpd00010_c + 5.0 cpd00011_c + 2.0 cpd00019_c + cpd36805_c

rxn41814_c: cpd00001_c + cpd22518_c <=> 2.0 cpd26683_c

rxn41824_c: cpd00022_c + cpd00411_c --> cpd00010_c + cpd00067_c

rxn41840_c: cpd00001_c --> cpd00251_c

rxn41846_c: cpd00001_c + cpd21757_c <=> cpd21753_c

rxn41848_c: cpd33241_c --> cpd31223_c

rxn41850_c: cpd00026_c + cpd27033_c <=> cpd00014_c + cpd27032_c

rxn41853_c: cpd32428_c <=> cpd31753_c

rxn41859_c: cpd00149_c + cpd03426_c <=> 2.0 cpd00067_c

rxn41860_c: cpd00002_c + cpd00161_c + cpd11493_c <=> cpd00012_c + cpd00018_c + cpd37268_c

rxn41870_c: cpd36289_c <=>

rxn41896_c: cpd00001_c + cpd19505_c + 2.0 cpd28260_c <=> 4.0 cpd00067_c + cpd03520_c + 2.0 cpd28253_c

rxn41898_c: cpd07035_c <-- cpd32719_c

rxn41899_c: cpd00017_c + cpd21755_c <=> cpd00019_c + cpd00067_c + cpd27835_c

rxn41902_c: cpd00002_c + cpd00053_c + cpd27100_c <=> cpd00012_c + cpd00018_c

rxn41912_c: cpd00007_c + cpd36434_c <=> cpd33315_c

rxn41916_c: cpd00076_c <-- cpd27165_c

rxn41924_c: cpd00002_c + cpd00053_c + cpd00703_c --> cpd00012_c + cpd00018_c + cpd00067_c

rxn41927_c: cpd00002_c + cpd00051_c + cpd22281_c <=> cpd00012_c + cpd00018_c

rxn41933_c: 2.0 cpd00001_c + cpd28218_c <=> 2.0 cpd00023_c + cpd00087_c

rxn41935_c: cpd00002_c + cpd00069_c + cpd28243_c <=> cpd00012_c + cpd00018_c

rxn41936_c: 4.0 cpd00083_c + cpd32798_c <=> 4.0 cpd00031_c + 4.0 cpd00067_c + cpd36597_c

rxn41975_c: cpd11493_c + cpd25932_c <=> cpd00018_c + cpd00067_c

rxn41986_c: cpd00002_c + cpd00129_c + cpd11493_c <=> cpd00012_c + cpd00018_c + cpd36315_c

rxn41993_c: cpd00067_c + cpd11180_c + cpd28018_c <=> cpd02016_c

rxn42007_c: 2.0 cpd27194_c <=> 2.0 cpd00067_c + cpd08085_c + 2.0 cpd11493_c

rxn42014_c: cpd00007_c + cpd00325_c + cpd21035_c <=> cpd00001_c + cpd11630_c

rxn42024_c: cpd00192_c + cpd00248_c <-- cpd00010_c

rxn42025_c: cpd00017_c + cpd01217_c <-- cpd00019_c

rxn42032_c: 8.0 cpd00067_c + 8.0 cpd00070_c + cpd04029_c + cpd11493_c <=> 2.0 cpd00001_c + 9.0 cpd00010_c + 8.0 cpd00011_c + cpd36673_c

rxn42036_c: cpd00134_c + cpd11799_c <=> cpd00010_c + cpd02165_c

rxn42052_c: cpd00017_c + cpd00309_c <=> cpd00019_c + cpd00067_c

rxn42056_c: cpd11493_c + cpd32435_c <=> cpd00018_c + cpd00067_c + cpd36332_c

rxn42057_c: cpd28358_c + cpd30770_c <=> cpd00014_c + cpd00067_c + cpd28359_c

rxn42060_c: 2.0 cpd00005_c + 3.0 cpd00067_c + cpd00519_c + cpd27419_c <=> cpd00001_c + 2.0 cpd00006_c + cpd00010_c + cpd00011_c + cpd36435_c

rxn42066_c: cpd11640_c --> cpd00239_c

rxn42071_c: cpd35508_c + cpd37168_c <=> cpd00014_c + cpd00067_c + cpd36677_c

rxn42076_c: cpd00002_c + cpd00644_c <-- cpd00008_c + cpd00067_c

rxn42080_c: 2.0 cpd00001_c + 2.0 cpd00002_c + cpd27307_c + cpd28083_c <=> 2.0 cpd00008_c + 2.0 cpd00009_c + 3.0 cpd00067_c + cpd27073_c + cpd27392_c

rxn42088_c: cpd00363_c + cpd01014_c <-- cpd24339_c

rxn42106_c: cpd00001_c + cpd03959_c <=> cpd00067_c + cpd00099_c

rxn42129_c: cpd34764_c --> cpd31550_c

rxn42131_c: cpd00004_c + cpd00067_c + 2.0 cpd28018_c <=> cpd00003_c + 2.0 cpd28170_c

rxn42140_c: cpd00007_c + cpd21035_c + cpd27074_c <=> cpd00001_c + cpd11630_c

rxn42142_c: cpd00007_c + cpd01069_c + cpd21035_c <=> 2.0 cpd00001_c + cpd11630_c

rxn42143_c: cpd00026_c + cpd27809_c <=> cpd00014_c + cpd00067_c + cpd12607_c

rxn42146_c: 4.0 cpd00005_c + 2.0 cpd00017_c + cpd00022_c + 6.0 cpd00067_c + 4.0 cpd00070_c + cpd11493_c <=> 3.0 cpd00001_c + 4.0 cpd00006_c + 5.0 cpd00010_c + 4.0 cpd00011_c + 2.0 cpd00019_c + cpd37043_c

rxn42156_c: cpd00023_c <--

rxn42164_c: cpd34995_c <-- cpd31696_c

rxn42170_c: cpd00002_c + cpd00009_c + cpd00020_c <-- cpd00012_c + cpd00018_c + cpd00067_c

rxn42176_c: cpd00002_c + cpd03848_c + cpd11493_c <=> cpd00012_c + cpd00018_c + cpd22388_c

rxn42177_c: cpd00001_c + cpd26760_c <=> cpd21085_c

rxn42178_c: 5.0 cpd00005_c + 12.0 cpd00067_c + cpd00070_c --> 2.0 cpd00001_c + 5.0 cpd00006_c + 7.0 cpd00010_c + 7.0 cpd00011_c + cpd08794_c

rxn42186_c: cpd00083_c + cpd36233_c <=> cpd00031_c + cpd00067_c

rxn42197_c: cpd00004_c + cpd00007_c + cpd00067_c + cpd00300_c <=> cpd00001_c + cpd00003_c

rxn42198_c: cpd11493_c + cpd32461_c <=> cpd00018_c + cpd00067_c + cpd36315_c

rxn42232_c: cpd00001_c + cpd37007_c <=> cpd00067_c + cpd11493_c + cpd34567_c

rxn42256_c: cpd00022_c + cpd00098_c <-- cpd00010_c

rxn42265_c: cpd23154_c + cpd37000_c <=> cpd00067_c + cpd11493_c

rxn42266_c: cpd00002_c + cpd11493_c + cpd34985_c <=> cpd00012_c + cpd00018_c + cpd36686_c

rxn42278_c: cpd00007_c + cpd00024_c + cpd02374_c --> cpd00011_c + cpd00036_c

rxn42297_c: cpd34670_c + cpd37242_c <=> cpd11493_c + cpd31988_c

rxn42304_c: cpd00003_c + cpd00222_c <-- cpd00004_c + cpd00067_c

rxn42323_c: cpd00163_c + cpd36126_c <=> cpd00014_c + cpd00067_c

rxn42325_c: cpd36694_c <=> cpd00067_c + cpd09212_c + cpd11493_c

rxn42326_c: cpd33530_c <-- cpd31393_c

rxn42337_c: cpd00001_c + cpd37186_c <=> 2.0 cpd36405_c

rxn42339_c: cpd00007_c + cpd00233_c + cpd12184_c <=> cpd00001_c + cpd01895_c + cpd12365_c

rxn42360_c: cpd00007_c + cpd21035_c + cpd27424_c <=> cpd00001_c + cpd11630_c + cpd27708_c

rxn42370_c: cpd00017_c + cpd27096_c <=> cpd00019_c + cpd00067_c

rxn42374_c: 3.0 cpd00005_c + 3.0 cpd00022_c + 3.0 cpd00067_c + cpd11493_c <=> cpd00001_c + 3.0 cpd00006_c + 3.0 cpd00010_c + cpd00011_c + cpd36180_c

rxn42376_c: cpd00017_c + cpd27620_c <=> cpd00019_c + cpd00067_c

rxn42380_c: cpd00001_c + cpd00002_c + cpd28388_c <=> cpd00012_c + cpd11493_c + cpd26566_c

rxn42405_c: cpd00022_c + cpd36347_c <=> cpd00010_c + cpd00067_c

rxn42413_c: cpd00084_c + cpd27355_c <=> cpd00035_c + cpd14548_c

rxn42421_c: cpd00006_c + cpd01456_c --> cpd00005_c

rxn42451_c: cpd00002_c + cpd00599_c + cpd27833_c <=> cpd00012_c + cpd00018_c

rxn42453_c: cpd03452_c + cpd11493_c <=> cpd00018_c + cpd00067_c + cpd37100_c

rxn42458_c: cpd00001_c + cpd27994_c <=> 2.0 cpd00537_c

rxn42474_c: cpd30770_c <-- cpd00014_c

rxn42478_c: cpd36951_c <=> cpd34898_c

rxn42504_c: cpd00017_c + cpd22422_c <=> cpd00019_c + cpd00067_c + cpd12372_c

rxn42511_c: cpd36289_c + cpd36860_c <=> cpd00067_c + cpd11493_c

rxn42523_c: cpd00002_c + cpd00705_c + cpd11493_c <=> cpd00012_c + cpd00018_c + cpd36532_c

rxn42534_c: cpd00002_c + cpd00599_c + cpd11493_c <=> cpd00012_c + cpd00018_c

rxn42543_c: cpd27645_c <=> cpd02229_c

rxn42545_c: cpd26950_c <=> cpd00048_c + cpd03019_c + cpd23457_c

rxn42547_c: cpd00042_c + cpd34944_c <-- cpd00067_c + cpd04372_c

rxn42555_c: cpd11493_c + cpd25931_c <=> cpd00018_c

rxn42558_c: cpd33835_c <-- cpd33750_c

rxn42575_c: cpd36709_c <=> cpd11493_c + cpd31504_c

rxn42576_c: cpd00007_c + cpd03936_c + cpd21035_c <=> cpd00001_c + cpd11630_c

rxn42579_c: cpd23154_c + cpd36372_c <=> cpd00067_c + cpd11493_c + cpd36647_c

rxn42580_c: cpd00001_c + cpd27156_c <=> cpd28173_c

rxn42581_c: cpd00001_c + cpd11493_c <=> cpd00067_c + cpd00834_c + cpd11799_c

rxn42592_c: cpd31965_c --> cpd00011_c + cpd34717_c

rxn42599_c: cpd00001_c + cpd36651_c <=> cpd00067_c + cpd11493_c + cpd32181_c

rxn42634_c: cpd00033_c + cpd02882_c <-- cpd31670_c

rxn42635_c: cpd00002_c + cpd01107_c + cpd11493_c <=> cpd00012_c + cpd00018_c

rxn42657_c: cpd00020_c + cpd00241_c <-- cpd00067_c + cpd00295_c

rxn42658_c: cpd25491_c + cpd28218_c <=> cpd32090_c

rxn42679_c: cpd00005_c + cpd00007_c + cpd00067_c + cpd36077_c <=> cpd00001_c + cpd00006_c

rxn42697_c: cpd00002_c + cpd11493_c + cpd26607_c <=> cpd00012_c + cpd00018_c + cpd28167_c

rxn42711_c: cpd05191_c + cpd28218_c <=> cpd24831_c

rxn42718_c: cpd00001_c <=> cpd37014_c

rxn42724_c: cpd00037_c + cpd00122_c <=> cpd00014_c + cpd26014_c

rxn42734_c: cpd00002_c + cpd05196_c + cpd11493_c <=> cpd00012_c + cpd00018_c + cpd22441_c

rxn42746_c: cpd25747_c + cpd27171_c <=> cpd25748_c

rxn42758_c: cpd00003_c + cpd00266_c <-- cpd00004_c + cpd00067_c

rxn42784_c: cpd28326_c <=> cpd00204_c + cpd36984_c

rxn42801_c: cpd00002_c + cpd00035_c + cpd36998_c <=> cpd00012_c + cpd00018_c + cpd00067_c

rxn42809_c: 8.0 cpd00005_c + 2.0 cpd00017_c + 14.0 cpd00067_c + 8.0 cpd00070_c + cpd11493_c <=> 6.0 cpd00001_c + 8.0 cpd00006_c + 8.0 cpd00010_c + 8.0 cpd00011_c + 2.0 cpd00019_c + cpd37122_c

rxn42810_c: cpd00005_c + cpd00067_c + 2.0 cpd28018_c <=> cpd00006_c + 2.0 cpd28170_c

rxn42821_c: cpd00001_c + cpd36869_c <=> cpd00048_c + cpd00067_c + cpd14548_c

rxn42841_c: cpd00027_c --> cpd00179_c

rxn42856_c: cpd00415_c --> cpd01822_c

rxn42860_c: cpd00001_c + cpd11726_c <=> cpd00067_c + cpd11493_c + cpd23507_c

rxn42862_c: cpd14548_c + cpd28307_c <=> cpd27355_c + cpd28207_c

rxn42865_c: cpd00002_c + cpd00069_c + cpd36063_c <=> cpd00012_c + cpd00018_c + cpd00067_c + cpd11493_c + cpd32083_c

rxn42885_c: cpd00038_c + cpd00047_c + cpd00067_c <-- cpd00001_c + cpd00011_c

rxn42889_c: cpd00604_c --> cpd18070_c

rxn42894_c: cpd27517_c + cpd28218_c <=> cpd00067_c + cpd27485_c

rxn42898_c: cpd00025_c + cpd00059_c + cpd00067_c <-- 2.0 cpd00001_c

rxn42912_c: cpd00001_c + cpd36117_c <=> cpd00067_c + cpd11493_c + cpd31563_c

rxn42917_c: cpd00042_c + cpd10096_c <=> cpd00067_c + cpd00099_c

rxn42921_c: cpd00003_c + cpd00383_c <-- cpd00004_c + cpd00067_c

rxn42924_c: cpd11493_c + cpd35943_c <=> cpd00018_c + cpd00067_c + cpd36737_c

rxn42930_c: cpd00001_c + cpd11677_c <=> cpd00067_c + cpd26958_c + cpd27058_c

rxn42935_c: cpd00002_c + cpd00039_c + cpd27388_c <=> cpd00012_c + cpd00018_c

rxn42944_c: cpd27340_c + cpd37055_c <=> cpd11493_c + cpd36304_c

rxn42949_c: cpd00059_c + cpd00067_c + cpd21160_c <-- cpd00001_c + cpd21159_c

rxn42952_c: cpd00067_c + cpd03846_c + cpd23347_c <-- cpd00001_c + cpd22604_c

rxn42956_c: cpd00248_c + cpd28018_c <=>

rxn42982_c: cpd14548_c + cpd28060_c <=> cpd00239_c + cpd27355_c + cpd27735_c

rxn43003_c: cpd00007_c + cpd00059_c + cpd00067_c + cpd00374_c <-- cpd00001_c + cpd02599_c

rxn43018_c: cpd00002_c + cpd00069_c + cpd37043_c <=> cpd00012_c + cpd00018_c + 2.0 cpd00067_c + cpd11493_c + cpd34602_c

rxn43029_c: cpd00001_c <=> cpd36896_c

rxn43044_c: cpd00005_c + cpd00017_c + 2.0 cpd00084_c + cpd00599_c <-- 4.0 cpd00001_c + cpd00006_c + cpd08828_c

rxn43045_c: cpd00666_c <--

rxn43051_c: cpd00002_c + cpd12109_c <=> cpd00008_c + cpd00067_c + cpd21768_c

rxn43052_c: cpd00703_c + cpd27171_c <=> cpd25734_c

rxn43054_c: 3.0 cpd00017_c + cpd00022_c + 12.0 cpd00067_c + 7.0 cpd00070_c + cpd11493_c + 8.0 cpd27640_c <=> 6.0 cpd00001_c + 8.0 cpd00010_c + 7.0 cpd00011_c + 3.0 cpd00019_c + 8.0 cpd27638_c + cpd37045_c

rxn43064_c: cpd00047_c + cpd00067_c + cpd28109_c <=> cpd00001_c + cpd00011_c

rxn43074_c: cpd31089_c --> cpd32823_c

rxn43078_c: cpd00007_c + cpd01059_c + cpd21035_c <=> cpd00001_c + cpd11630_c

rxn43090_c: cpd36666_c <=> cpd36165_c

rxn43094_c: cpd00022_c + cpd36831_c <=> cpd00010_c + cpd00067_c

rxn43099_c: 4.0 cpd00042_c + 2.0 cpd00067_c + cpd03387_c <=> 3.0 cpd00001_c + cpd20131_c

rxn43107_c: cpd00022_c + cpd37184_c <=> cpd00010_c + cpd00067_c

rxn43112_c: cpd00001_c + cpd27190_c <=> cpd27058_c

rxn43115_c: cpd36925_c <=> cpd00067_c + cpd11493_c + cpd19561_c

rxn43123_c: cpd00001_c + cpd19505_c + 2.0 cpd36362_c <=> 4.0 cpd00067_c + cpd03520_c + 2.0 cpd27582_c

rxn43140_c: cpd00001_c + cpd12396_c <=> cpd00009_c + cpd11799_c

rxn43150_c: cpd00002_c + cpd00599_c + cpd11493_c <=> cpd00012_c + cpd00018_c + cpd36295_c

rxn43154_c: cpd00003_c + cpd00033_c + cpd28218_c <=> cpd00004_c + cpd00011_c + cpd00013_c

rxn43170_c: cpd00526_c + cpd11228_c <-- cpd01237_c

rxn43179_c: cpd00067_c + cpd00450_c + cpd27539_c <=> cpd00324_c

rxn43188_c: cpd00002_c + cpd11493_c + cpd33359_c <=> cpd00012_c + cpd00018_c + cpd36913_c

rxn43192_c: cpd00001_c + cpd27391_c <=> cpd00067_c + cpd02416_c + cpd02486_c

rxn43197_c: cpd01982_c <-- cpd34589_c

rxn43203_c: cpd11493_c + cpd32198_c <=> cpd00018_c + cpd00067_c + cpd36986_c

rxn43204_c: cpd00002_c + cpd00161_c + cpd11493_c <=> cpd00012_c + cpd00018_c

rxn43239_c: cpd00067_c + cpd11492_c + cpd11628_c <=> cpd00011_c + cpd11493_c + cpd36173_c

rxn43263_c: 5.0 cpd00005_c + 10.0 cpd00067_c + 3.0 cpd00070_c + cpd36332_c <=> 2.0 cpd00001_c + 5.0 cpd00006_c + 5.0 cpd00010_c + 5.0 cpd00011_c + cpd36903_c

rxn43269_c: cpd00067_c + cpd00081_c <--

rxn43271_c: cpd11493_c + cpd34368_c <=> cpd00018_c + cpd00067_c + cpd37139_c

rxn43310_c: cpd00007_c + 2.0 cpd00042_c <-- cpd00025_c

rxn43316_c: cpd00042_c + cpd17456_c <-- cpd00239_c

rxn43319_c: cpd00001_c + cpd00203_c + cpd11493_c <=> 2.0 cpd00009_c

rxn43328_c: cpd11493_c + cpd32347_c <=> cpd00018_c + cpd00067_c + cpd36497_c

rxn43338_c: 2.0 cpd00022_c <-- cpd00010_c

rxn43354_c: cpd35046_c --> cpd33704_c

rxn43367_c: cpd00002_c + cpd00323_c + cpd11493_c <=> cpd00012_c + cpd00018_c + cpd37169_c

rxn43379_c: cpd00001_c + cpd00002_c + cpd28383_c <=> cpd00012_c + cpd11493_c + cpd26579_c

rxn43380_c: cpd00002_c + cpd00054_c + cpd28152_c <=> cpd00012_c + cpd00018_c

rxn43383_c: cpd11493_c + cpd34368_c <=> cpd00018_c + cpd00067_c + cpd37162_c

rxn43385_c: cpd36198_c <=> cpd00108_c + cpd00138_c + cpd00751_c + cpd03647_c + cpd27109_c

rxn43386_c: cpd00017_c + cpd36452_c <=> cpd00019_c + cpd00067_c + cpd22117_c

rxn43391_c: cpd00001_c + cpd27490_c <=> cpd00027_c + cpd00179_c

rxn43396_c: cpd00002_c + cpd11493_c + cpd31629_c <=> cpd00012_c + cpd00018_c

rxn43402_c: cpd00026_c + cpd22650_c <=> cpd00014_c + cpd00067_c + cpd36546_c

rxn43403_c: cpd00571_c <-- cpd00001_c

rxn43404_c: cpd00022_c + 6.0 cpd00067_c + 6.0 cpd00070_c + cpd11493_c <=> cpd00001_c + 7.0 cpd00010_c + 6.0 cpd00011_c + cpd36534_c

rxn43423_c: cpd00002_c + cpd00214_c + cpd11493_c <=> cpd00012_c + cpd00018_c

rxn43424_c: cpd00001_c + cpd00002_c + cpd28603_c <=> cpd00012_c + cpd11493_c + cpd26580_c

rxn43430_c: cpd00001_c + cpd36402_c <=> cpd00067_c + cpd11493_c + cpd32470_c

rxn43454_c: cpd14548_c <=> cpd27355_c

rxn43461_c: cpd00007_c + cpd22518_c + cpd26781_c <=> cpd00001_c + cpd26683_c + cpd27029_c + cpd36871_c

rxn43485_c: cpd00067_c + cpd05191_c + cpd27539_c <=> cpd24831_c

rxn43494_c: cpd00001_c + cpd12607_c <=> cpd00027_c + cpd27809_c

rxn43501_c: 9.0 cpd00067_c + 9.0 cpd00070_c + cpd11493_c <=> 9.0 cpd00010_c + 9.0 cpd00011_c + cpd36414_c

rxn43505_c: cpd00047_c + cpd00052_c + cpd00067_c <-- cpd00001_c + cpd00011_c

rxn43508_c: 6.0 cpd00005_c + 9.0 cpd00067_c + 3.0 cpd00519_c + cpd01393_c + cpd11493_c <=> 3.0 cpd00001_c + 6.0 cpd00006_c + 4.0 cpd00010_c + 3.0 cpd00011_c

rxn43511_c: cpd00042_c + cpd31409_c <-- cpd00165_c

rxn43513_c: cpd00067_c + cpd00425_c + cpd27536_c <=> cpd00187_c

rxn43546_c: cpd35508_c + cpd37032_c <=> cpd00014_c + cpd00067_c

rxn43561_c: 2.0 cpd00042_c + cpd01608_c <=> cpd00067_c + cpd00081_c + cpd00084_c

rxn43564_c: cpd00002_c + cpd00035_c + cpd11493_c <=> cpd00012_c + cpd00018_c + cpd37096_c

rxn43572_c: cpd00584_c + cpd31431_c <=> cpd00046_c + cpd00067_c + cpd37039_c

rxn43578_c: cpd00350_c + cpd36121_c <=> cpd00012_c

rxn43579_c: cpd28101_c <=> cpd00108_c + cpd00280_c + cpd00396_c + cpd03647_c

rxn43593_c: cpd34533_c + cpd36432_c <=> cpd11493_c + cpd34905_c

rxn43607_c: cpd00007_c + cpd00059_c + cpd00067_c + cpd05472_c <-- cpd00001_c + cpd05450_c

rxn43630_c: <=> cpd25964_c

rxn43652_c: 2.0 cpd00042_c + cpd01251_c <-- 2.0 cpd00135_c

rxn43656_c: cpd00002_c + cpd00069_c + cpd11493_c <=> cpd00012_c + cpd00018_c + cpd36077_c

rxn43660_c: cpd00002_c + cpd00069_c + cpd36185_c <=> cpd00012_c + cpd00018_c + 2.0 cpd00067_c + cpd11493_c + cpd34607_c

rxn43662_c: cpd00004_c + cpd00007_c + cpd00067_c + cpd04851_c --> cpd00003_c

rxn43667_c: cpd36313_c + cpd36662_c <=> cpd00067_c + cpd11493_c + cpd36438_c

rxn43683_c: cpd23797_c <=> cpd06433_c

rxn43685_c: cpd00001_c + cpd26711_c <=> cpd01157_c + cpd23788_c

rxn43687_c: cpd27312_c <=> cpd26834_c

rxn43689_c: cpd00002_c + cpd00129_c + cpd11493_c <=> cpd00012_c + cpd00018_c + cpd36887_c

rxn43695_c: cpd00002_c + cpd00103_c + cpd01831_c --> cpd00008_c + cpd00009_c + cpd00012_c + cpd02709_c

rxn43698_c: cpd14956_c + cpd27144_c <=> cpd00067_c + cpd11493_c + cpd27820_c

rxn43701_c: cpd00001_c + cpd27155_c <=> cpd28173_c

rxn43704_c: cpd28355_c <=> cpd00027_c + cpd00709_c + cpd00751_c + cpd01487_c

rxn43705_c: 15.0 cpd00005_c + 23.0 cpd00067_c + 8.0 cpd00519_c + cpd15277_c <=> 7.0 cpd00001_c + 15.0 cpd00006_c + 8.0 cpd00010_c + 8.0 cpd00011_c + cpd36949_c

rxn43711_c: cpd00095_c + cpd36197_c <=> cpd11493_c + cpd36004_c

rxn43726_c: cpd00001_c + cpd28355_c <=> cpd23850_c + cpd23864_c + cpd23865_c

rxn43728_c: cpd11799_c + cpd37247_c <=> cpd12396_c + cpd19031_c

rxn43736_c: cpd00002_c + cpd00084_c + cpd26668_c <=> cpd00012_c + cpd00018_c

rxn43745_c: cpd00007_c + cpd07097_c + cpd21035_c <=> cpd00001_c + cpd11630_c

rxn43750_c: cpd00144_c + cpd27157_c <=> cpd00014_c + cpd00067_c

rxn43752_c: 2.0 cpd00083_c + cpd36597_c <=> 2.0 cpd00031_c + 2.0 cpd00067_c + cpd32462_c

rxn43759_c: cpd11628_c + cpd35485_c <=> cpd00067_c + cpd11493_c + cpd13403_c

rxn43762_c: cpd00002_c + cpd00084_c + cpd11493_c <=> cpd00012_c + cpd00018_c + cpd23227_c

rxn43776_c: cpd00001_c + 2.0 cpd11836_c <=> cpd00067_c + 2.0 cpd11493_c + cpd21992_c

rxn43784_c: 3.0 cpd36284_c <=> cpd03453_c + 3.0 cpd11493_c

rxn43789_c: cpd00001_c + cpd37064_c <=> cpd00067_c + cpd11493_c + cpd33939_c

rxn43791_c: cpd00042_c + cpd27518_c <=> cpd26936_c

rxn43804_c: cpd00044_c + cpd36474_c <=> cpd00045_c + cpd00067_c + cpd12830_c

rxn43814_c: cpd07568_c <-- cpd23893_c

rxn43823_c: cpd00007_c + cpd36434_c <=> cpd31879_c + cpd33571_c + cpd34484_c + cpd34692_c

rxn43837_c: cpd14548_c + cpd22312_c + 2.0 cpd28082_c <=> cpd00018_c + 2.0 cpd27757_c + cpd28205_c + cpd28259_c

rxn43848_c: cpd00007_c + cpd21035_c + cpd22019_c <=> cpd00001_c + cpd11630_c + cpd21993_c

rxn43893_c: 13.0 cpd00005_c + 20.0 cpd00067_c + 7.0 cpd00519_c + cpd15277_c <=> 6.0 cpd00001_c + 13.0 cpd00006_c + 7.0 cpd00010_c + 7.0 cpd00011_c + cpd36432_c

rxn43895_c: cpd02246_c + cpd27511_c <=> cpd00067_c + cpd27536_c

rxn43907_c: cpd00098_c + cpd00865_c <-- cpd00027_c

rxn43918_c: cpd00013_c + cpd00032_c + cpd36180_c <=> 3.0 cpd00001_c + cpd07035_c + cpd11493_c

rxn43925_c: cpd00022_c + 3.0 cpd00067_c + 3.0 cpd00070_c + cpd11493_c <=> cpd00001_c + 4.0 cpd00010_c + 3.0 cpd00011_c

rxn43927_c: cpd00002_c + cpd01190_c <-- cpd00008_c + cpd00067_c

rxn43956_c: cpd00144_c + cpd27131_c <=> cpd00014_c + cpd00067_c

rxn43960_c: cpd00083_c + cpd27295_c <=> cpd00031_c + cpd00067_c

rxn43961_c: cpd00002_c + cpd00010_c + cpd27708_c <=> cpd00012_c + cpd00018_c + cpd36526_c

rxn43986_c: cpd00052_c + 2.0 cpd17154_c <=> cpd00001_c

rxn44008_c: cpd00002_c + cpd11493_c + cpd33351_c <=> cpd00012_c + cpd00018_c + cpd36631_c

rxn44012_c: cpd00001_c + cpd27889_c <=> 2.0 cpd00988_c

rxn44020_c: cpd00001_c + cpd25936_c <-- cpd00027_c
